# Insecticidal metabolites from *Serratia marcescens* associated with the fall armyworm (*Spodoptera frugiperda*): molecular identification, metabolomic profiling and molecular docking insights

**DOI:** 10.1038/s41598-026-66375-7

**Published:** 2026-08-19

**Authors:** Kreema A. El-Lebody, Ramy E. El-Ansary, Shaimaa A. Nour, Rasha G. Salim, Mohamed M. Elsebaie, Ghada M. El-Sayed

**Affiliations:** 1 Plant Protection Research Institute, Agriculture Research Centre, Giza, 12627 Dokki Egypt; 2 Zoology and Entomology Department, Faculty of Science, Al-Azhar University, Cairo, Egypt; 3 Chemistry of Natural and Microbial Products Department, Pharmaceutical and Drug Industries Research Institute, National Research Centre, 33 El‑Bohouth St., Dokki, P.O. 12622, Giza, Egypt; 4 Microbial Genetics Department, Biotechnology Research Institute, National Research Centre, 33 El‑Bohouth St., (Former El‑Tahrir St.) Dokki, P.O. 12622, Giza, Egypt; 5 Pharmaceutical Organic Chemistry Department, Faculty of Pharmacy, Al-Azhar University, Nasr City, 11884 Cairo Egypt

**Keywords:** *Spodoptera frugiperda*, *Serratia marcescens*, Acetylcholinesterase, Cardenolide, Diketopiperazines, Squalene, Molecular docking, Bioinsecticides, Sustainable pest management, Biochemistry, Biological techniques, Biotechnology, Chemical biology, Microbiology

## Abstract

The fall armyworm (*S. frugiperda*) has developed resistance to numerous insecticides and is currently considered one of the most destructive pests threatening global crop production. Consequently, the development of environmentally sustainable pest management strategies has become increasingly important. Insect-associated pathogenic bacteria represent a promising source of bioactive metabolites with potential insecticidal properties. In the present study, pathogenic bacteria associated with *S. frugiperda* were isolated and molecularly identified as *Serratia marcescens* strain INS420 based on 16 S rRNA gene sequencing. The secondary metabolites produced by this bacterium demonstrated significant insecticidal activity under both laboratory and field conditions. Metabolic profiling of the extracted compounds was performed using liquid chromatography–mass spectrometry (LC–MS) and gas chromatography–mass spectrometry (GC–MS), revealing the presence of several bioactive compounds, including diketopiperazines, fatty acids and their esters, squalene, phthalate derivatives, and a cardenolide. To gain insights into the potential mechanism of action, molecular docking simulations were conducted to evaluate the binding affinity of the identified metabolites with *S. frugiperda* acetylcholinesterase (AChE). Among the detected compounds, squalene and several fatty acid derivatives exhibited stable interactions within the active site of the enzyme, suggesting a possible inhibitory effect on AChE activity. Collectively, these findings demonstrate that *S. marcescens* associated with *S. frugiperda* produces metabolites with notable insecticidal potential and highlight insect-associated pathogenic bacteria as a valuable source of bioactive compounds for the sustainable management of fall armyworm.

**Supplementary Information:**

The online version contains supplementary material available at 10.1038/s41598-026-66375-7.

## Introduction

The management of fall armyworm (*S. frugiperda*), a major pest of maize and other staple crops, is increasingly shifting toward biotechnological control strategies. Building on prior studies^1–3^, we investigate *S. marcescens* as a promising biocontrol agent. This entomopathogenic bacterium demonstrates potent larvicidal activity with reduced environmental impact compared to chemical insecticides. Enzymatic extracts of *S. marcescens* induce up to ~ 93% larval mortality, accompanied by growth inhibition and developmental delay^1^. Following ingestion, the bacterium disrupts the midgut epithelium, enabling systemic invasion and septicemia^2^. This pathogenicity is driven by secreted virulence factors—particularly chitinases and proteases—that degrade host structural barriers^4^. A 50 kDa serralysin-like metalloprotease has been identified as a key determinant of insecticidal activity^3^. Nevertheless, translating *S. marcescens* and its secondary metabolites from a laboratory model into a field-ready biocontrol agent is limited by significant gaps in our molecular understanding. Although its general bioactivity is well documented, the specific insecticidal metabolites and toxins responsible for larval mortality remain incompletely characterized. Previous studies have primarily examined either broad virulence gene expression^2^ or individual compounds such as prodigiosin^5^. Consequently, the contribution of other bioactive metabolites to insecticidal activity has received comparatively little attention. As a result, the precise mode of action—particularly the interaction between specific bacterial metabolites and host immune responses—remains only partially understood.

Previous studies on Serratia species as biocontrol agents against *S. frugiperda* have largely focused on the pyrrolic secondary metabolite prodigiosin. This red pigment is one of the most studied metabolites in Serratia, noted for its antimicrobial^6,7^ and insecticidal activities^8,9^. Another research has emphasized enzymatic bioactivity, particularly chitinases^10,11^. In contrast, the present study targets secondary metabolites beyond prodigiosin and enzymes, aiming to assess their potential to disrupt *S. frugiperda* vitality. Its novelty lies in an integrated approach that combines chemical profiling (LC–MS), in silico molecular docking, and larvicidal assays to evaluate these previously underexplored insecticidal compounds.

Molecular docking has become a supportive computational tool in modern research, enabling the accurate prediction of interactions between bioactive compounds and their molecular targets. It accelerates the identification of novel compounds with potential affinity for proteins critical to organismal survival, while significantly reducing experimental time and cost^12^. By simulating ligand–receptor binding at atomic resolution, molecular docking facilitates the identification of compounds capable of modulating key proteins within specific biological systems. In this study, molecular docking simulation was used to assess the binding of LC–MS–identified *S. marcescens* metabolites to acetylcholinesterase. The results provide strong justification for further computational optimization, advanced chemical profiling, and experimental validation, which may ultimately support the development of new molecular scaffolds for insecticidal bioagents^10^.

This study aimed to isolate entomopathogenic bacteria and extract their secondary metabolites for potential use as biocontrol agents against *S. frugiperda* in both laboratory and field settings. Metabolic profiles were characterized using LC–MS and GC, and the bioactivity of identified compounds was evaluated computationally to predict their ability to disrupt insect vitality by targeting the key nervous system protein acetylcholinesterase.

## Materials and methods

### Sampling and isolation of insect-associated bacterial isolates

During the study conducted by Lebody et al.^13^ between 2022 and 2023, dead larvae and pupae of *S. frugiperda* observed during laboratory rearing were collected for bacterial isolation. The specimens were placed individually in sterile plastic bags and transported to the microbiology laboratory under aseptic conditions to prevent external contamination. Upon arrival, the dead larvae were surface-sterilized with 75% ethanol for 1 min, followed by two rinses with sterile distilled water to remove residual disinfectant. The sterilized samples were then air-dried in a laminar airflow cabinet for approximately 10 min. Each larva was aseptically dissected in a sterile Petri dish using flame-sterilized dissecting tools. The internal tissues were streaked onto nutrient agar (NA) plates, a non-selective general-purpose medium that supports the growth of a broad range of culturable heterotrophic bacteria. Since the objective of this study was to isolate bacteria with insecticidal activity rather than comprehensively assess microbial diversity, NA was considered appropriate for the initial isolation and screening. The inoculated plates were incubated at 30 °C for 72 h. Bacterial colonies exhibiting distinct morphological characteristics or dominance were selected and purified by repeated streaking on fresh NA plates until single, homogeneous colonies were obtained. Pure cultures were maintained on nutrient agar slants at 4 °C for subsequent molecular identification and characterization^14,15^.

### Experimental insect and rearing conditions

A laboratory colony of *S. frugiperda* was established from larvae collected from 60-day-old maize (hybrid type 324) field in Beheira Governorate, Egypt (31.0728° N, 30.3063° E). The colony was maintained for six successive generations without exposure to insecticides under controlled conditions of 27 ± 1 °C, 65 ± 5% relative humidity, and a 16:8 h (light: dark) photoperiod. Newly hatched larvae were transferred to clean 1-L glass jars and provided with fresh maize leaves daily until they reached the fourth instar. To minimize cannibalism, fourth-instar larvae were transferred to 2-L glass jars containing a thick layer of fine sawdust at the bottom. Pupae were collected and maintained in separate containers until adult emergence. Emerged adults (seven males and five females) were placed in 3-L glass jars and supplied with a cotton wick soaked in 10% sucrose solution, which was replaced daily to reduce the risk of fungal contamination, following the method described by Kandil et al.^16^.

### Preparation of bacterial cell-free filtrate for secondary metabolite extraction and LC–MS/MS and GC–MS/MS analysis

A pure bacterial colony was precultured in 100 mL of nutrient broth and incubated at 30 °C for 16 h with constant agitation at 140 rpm. This preculture served as the seed inoculum for large-scale fermentation. A 50 mL aliquot of the seed culture (≈ 1 × 10⁶ cells/mL, 10% v/v of total volume) was transferred into 500 mL of sterile Peptone Glycerol (PG) medium (pH 7.2 ± 0.2), containing 4 g/L casein peptone, 0.6% (v/v) glycerol, and 1.5 g/L ammonium sulfate [(NH₄)₂SO₄]. The inoculated flasks were incubated at 30 °C with shaking at 140 rpm for 15 days to promote growth and metabolite production. After incubation, the fermentation broth was allowed to stand at 25 °C for 24 h to facilitate sedimentation of residual solids. The culture was then centrifuged at 10,000 rpm for 15 min to separate the biomass from the liquid phase. The resulting supernatant, or cell-free filtrate, was extracted with an equal volume of ethyl acetate. The biphasic mixture was stirred for 4 h, after which the organic phase was collected and evaporated using a rotary evaporator to yield the crude extract. The crude extract was subsequently lyophilized, pulverized, and stored in sterile glass vials at 4 °C until further chemical and biological assays^17–19^.

LC–MS/MS and GC–MS/MS analysis was performed in ESI positive mode, with separation over 30 min. A solvent blank was analyzed for comparison. HS-GC–MS analysis was conducted using a hexane–dichloromethane method, and compound identification was based on NIST library spectra.

### Laboratory bioassay – leaf dipping

The insecticidal activity of the crude extract obtained from the isolated bacteria was evaluated against fourth-instar *S. frugiperda*larvae using a leaf-dip bioassay with slight modifications to the method described by Moustafa et al.^20^,. Fresh maize leaves were thoroughly washed with distilled water, immersed in the crude extract for 30 s, and subsequently air-dried at room temperature. Treated leaves were then placed in 0.25-L glass jars, after which ten fourth-instar *S. frugiperda*larvae were introduced into each jar, with three replications per treatment. Two exposure regimes were evaluated: larvae were allowed to feed on treated leaves for 24 h (Group I) or 72 h (Group II). After the exposure period, larvae were transferred to untreated maize leaves and maintained under the same rearing conditions. Larval mortality was recorded at 24, 48, and 72 h post-treatment. Control groups consisted of larvae fed on untreated maize leaves following the procedure described by Hamada et al.^21^,. Each bioassay was independently repeated twice to ensure reproducibility of the results.

### Evaluation of insecticidal activity under field conditions

As an extension of the study by Lebody et al.^13^, the insecticidal efficacy of the crude extract produced by the isolated bacterium was evaluated against *S. frugiperda* under natural field conditions during the 2023 summer maize growing season. Two field trials were conducted using white maize hybrid 2031 at two locations in Egypt: Kafr El-Akram (Quesna, Monufia Governorate) and Ezbet Abu Garf (Banha, Qalyubia Governorate). High pest pressure in both locations provided suitable conditions for evaluating the efficacy of the *S. marcescens* INS420 crude extract under natural field conditions. At the time of treatment, the maize plants at Kafr El-Akram were approximately 35 days old, whereas those at Ezbet Abu Garf were approximately 28 days old. The experiments were arranged in a completely randomized design with five replicates per treatment (crude extract treatment and untreated control), and each replicate plot measured 35 m². The crude extract was applied using the whorl application method at an application rate of 240 L ha⁻¹. Prior to treatment, 50 plants per plot (10 plants per replicate) were randomly selected and visually inspected to record the number of infested plants and larvae. Post-treatment assessments were conducted at 2, 5, and 7 days after application, during which the number of infested plants and larval counts were recorded. Treatment efficacy was calculated using the Henderson–Tilton formula^22^.

### Molecular identification of isolated bacteria and phylogenetic analysis

Purified bacterial isolates were cultured in nutrient broth at 30 °C for 16 h. Genomic DNA was extracted from the most promising isolate using the Thermo Scientific GeneJET Genomic DNA Purification Kit (K0721; Waltham, MA, USA) following the manufacturer’s protocol. The extracted DNA was used as a template for amplification of the 16 S rRNA gene using the universal bacterial primers 8 F (5′-AGAGTTTGATCCTGGCTCAG-3′) and 1492R (5′-GGTTACCTTGTTACGACTT-3′) (dos Santos et al., 2019). PCR amplification was performed in a 25 µL reaction mixture containing EmeraldAmp GT PCR Master Mix (2× Premix; Takara, Japan), 1 µL of each primer, and 2 µL of DNA template. The PCR cycling conditions were as follows: initial denaturation at 94 °C for 3 min; followed by 30 cycles of denaturation at 94 °C for 30 s, annealing at 50 °C for 1 min, and extension at 72 °C for 2 min; with a final extension at 72 °C for 10 min. The amplified 16 S rRNA gene product was purified and sequenced using the Sanger sequencing method^23^ with a BigDye X Terminator Kit (Thermo Fisher Scientific, USA) on an Applied Biosystems 3500 Genetic Analyzer. Using BioEdit software^24^, the 16 S rRNA gene sequence was edited. The obtained nucleotide sequences were analyzed using the NCBI BLASTN program (https://blast.ncbi.nlm.nih.gov/Blast.cgi) and aligned with available reference sequences for taxonomic identification. Homologous sequences found within the GenBank database were used for phylogenetic analysis and construction. There were twelve other GenBank sequences of the 16 S rRNA gene from bacteria that were initially assessed to be closely related. Using the MUSCLE algorithm^25^ in MEGA version 11^26^, a multiple sequence was done. Using the neighbour-joining (NJ) technique^27^, the phylogenetic relationships were determined with the support of 1,000 bootstrap replications^28^ to evaluate branch robustness. For the Jukes–Cantor substitution model^29^, the evolutionary distances were determined.

### In silico molecular docking simulation of identified metabolites with acetylcholine esterase in FAW wild-type

#### Molecular modeling of acetylcholinesterase (AChE)

The amino acid sequence of the *S. frugiperda* (FAW) wild-type acetylcholinesterase (AChE) enzyme (accession number: XTR48140.1) was retrieved from the National Center for Biotechnology Information (NCBI) database (https://www.ncbi.nlm.nih.gov/). The three-dimensional (3D) structure of AChE, both experimentally validated and computationally predicted, was obtained from the AlphaFold database. The corresponding PDB file was downloaded from UniProt (https://www.uniprot.org/uniprotkb/A0A9R0CUI4/entry) for subsequent molecular analyses. The finalized and energy-minimized protein model was employed for molecular docking studies to investigate its interactions with metabolites of *S. marcescens* identified through LC–MS and GC–MS analyses. The accuracy and reliability of the protein model were further validated using computational tools, including VERIFY3D^30^ and PROCHECK^31^.

### In silico molecular docking of identified metabolites with acetylcholinesterase

For molecular docking analysis, the two-dimensional (2D) structures of the identified metabolites were retrieved from the PubChem database in SDF format. The ligand structures were first converted to MOL2 format using Open Babel^32^, and subsequently converted to PDBQT format using AutoDock Tools to ensure compatibility with docking simulations. Hydrogen atoms were added to the three-dimensional (3D) structure of the target protein using the MGL Tools package in AutoDock Vina^33^. The active-site residues of the target protein were predicted using the COACH server^34^. Docking simulations were carried out using AutoDock Vina v1.0, which generated ten potential binding conformations for each ligand. The optimal docking pose was selected based on the lowest binding energy (most negative value), representing the most stable and energetically favorable interaction. Protein–ligand interactions were visualized and analyzed using BIOVIA Discovery Studio Visualizer (version 2021, Dassault Systèmes, San Diego, CA, USA). For each ligand, all ten docking poses were examined, and the conformation exhibiting the highest binding affinity was selected for detailed analysis. Two-dimensional interaction diagrams were generated using the “Show 2D Diagram” feature, while the “Show Distance” function was used to display hydrogen bond lengths and other interaction parameters. Receptor surface representations were also employed to clearly illustrate the ligand-binding regions within the acetylcholinesterase structure.

### Statistical analysis

In the laboratory bioassay, all treatments were conducted in three biological replicates and the average results were compared to negative control (untreated maize leaves). no mortality was observed in the untreated control group; therefore, the mortality percentage of treated larvae was calculated directly without correction using Abbott’s formula^35^. All data collected were directly subjected to analysis of variance (ANOVA) and significant means separated with Duncan’s Multiple Range Test (DMRT) at *P* < 0.05 level using the Computer Software Statistical Package (CO-STATE) User Manual Version 3.03, Barkley Co., USA. All results are presented as mean ± standard error (SE).

For the field experiments, treatment efficacy against both infested plants and larval density was calculated using the Henderson–Tilton formula^22^at 2, 5, and 7 days after treatment. The resulting efficacy percentages were analyzed without transformation. Statistical analyses were performed using one-way analysis of variance (ANOVA) in SPSS software (version 27; IBM Corp., 2023). The experimental data were analyzed according to a completely randomized design, following the procedures described by Steel et al.^36^,. When significant differences were detected, Duncan’s multiple range test was applied for mean separation at a significance level of *P* < 0.05. To ensure the robustness of the statistical results, the data were additionally analyzed following arcsine square-root transformation and repeated-measures ANOVA, which yielded results consistent with those obtained from the analysis of the original percentage data. All results are presented as mean ± standard error (SE).

## Results

### Isolation, molecular identification and phylogenetic analysis of the bacterial isolate

**Fig. 1:**
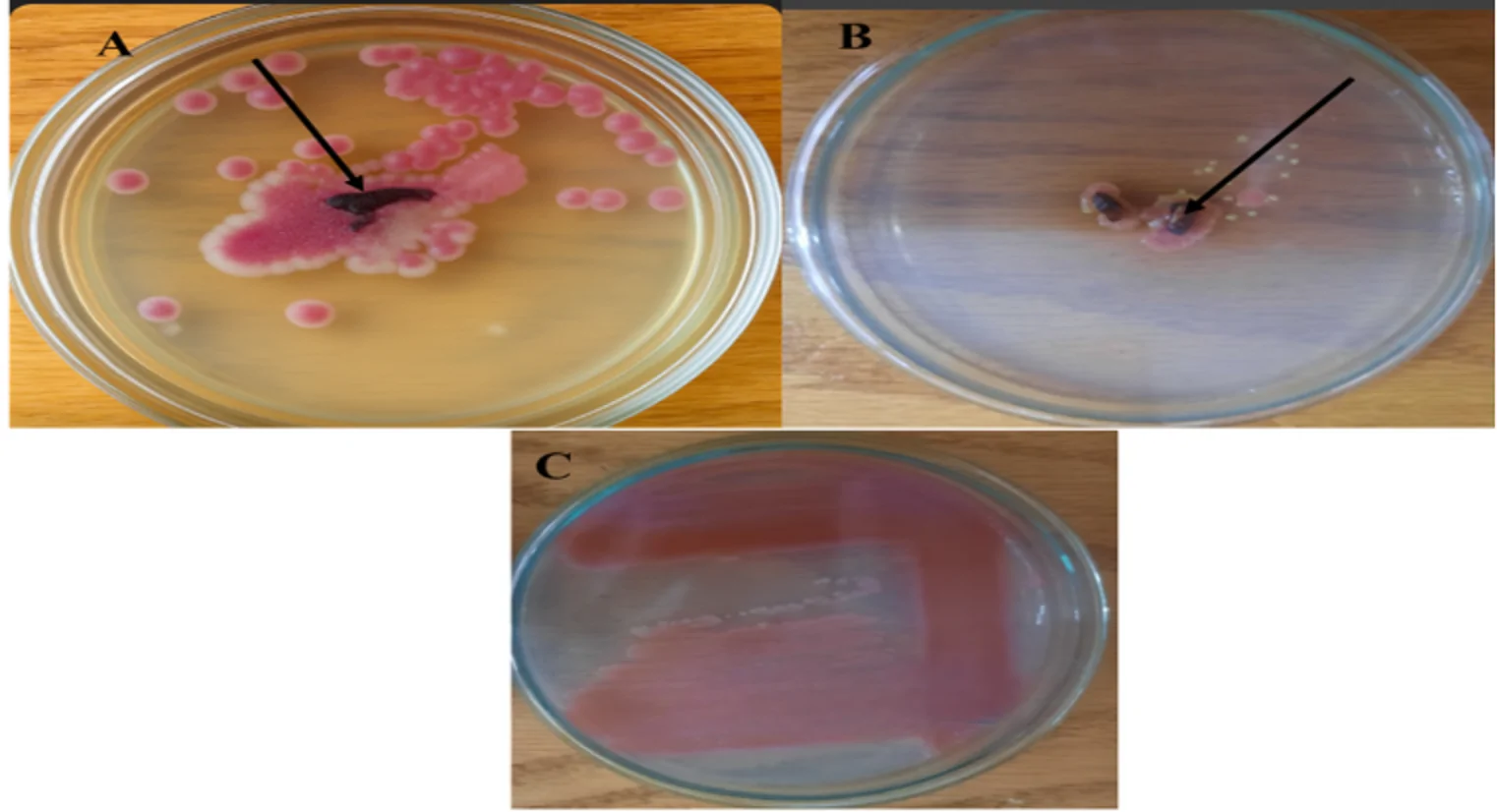
Isolation and purification of bacterial colonies from dead *S. frugiperda* larvae. (**A**,**B**) Mixed bacterial growth on nutrient agar plates showing distinct colony morphologies surrounding the dead larvae (black arrows). (**C**) Pure bacterial isolate obtained after successive streak plating.

Following the dissection of dead *S. frugiperda* larvae and incubation of the tissue on nutrient agar plates for 72 h at 30 °C, one morphologically distinct bacterial colony was observed. A red, smooth, and mucoid colony type predominated (Fig. 1). This isolate was purified through repeated sub-culturing and preserved on nutrient agar slants at 4 °C for further biochemical and molecular analyses related to insect biocontrol potential. Genomic DNA from the purified isolate was extracted, and the 16 S rRNA gene was amplified and sequenced. The obtained sequence was compared with sequences available in the NCBI GenBank database using the BLASTn algorithm (https://www.ncbi.nlm.nih.gov/genbank/). Alignment analysis revealed 100% sequence similarity with *S. marcescens*. The sequence was submitted to GenBank under accession number PP757784.

**Fig. 2:**
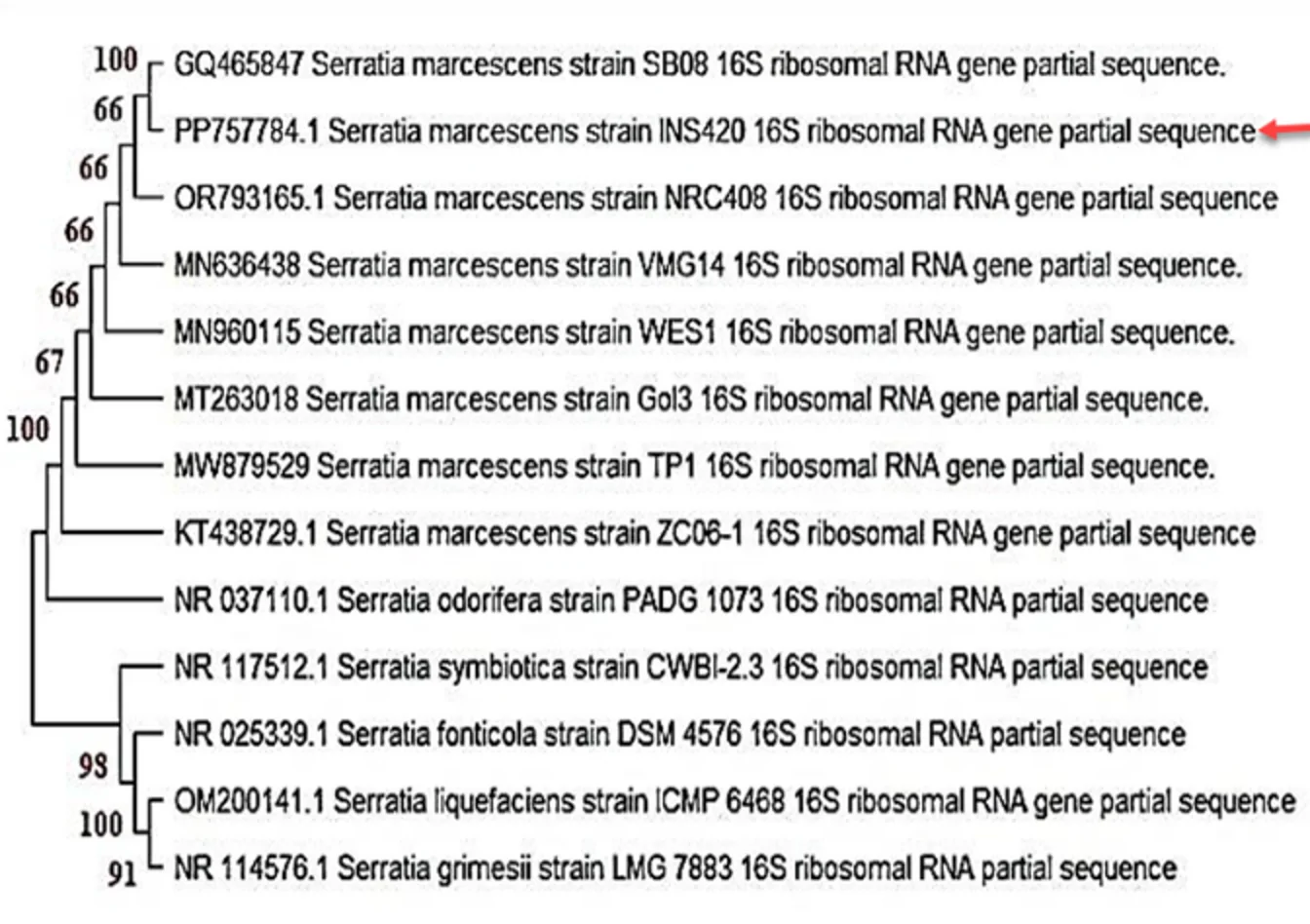
Phylogenetic relationships between the bacterial isolate INS420 and closely related bacterial species retrieved from the GenBank database, constructed using the neighbor-joining method. Numerical values at the nodes represent bootstrap percentages based on 1,000 replications. The red arrow indicates the position of *S. marcescens* strain INS420.

Phylogenetic analysis of the 16 S rRNA gene using the neighbour-joining method demonstrated that the isolate, designated *S. marcescens* strain INS420, clustered tightly with other *S. marcescens* strains (Fig. 2), confirming its taxonomic identity as determined by BLAST.

### Laboratory bioassay of *S. marcescens* strain INS420 crude extract against *S. frugiperda*

The insecticidal activity of the crude extract increased significantly with both exposure duration and post-treatment time, demonstrating a clear time- and exposure-dependent effect on the mortality of fourth-instar *S. frugiperda* larvae (Table 1). No mortality was observed in the untreated control throughout the experimental period. Following 24 h of exposure (Group I), larval mortality increased progressively from 6.7 ± 5.8% on day 1 to 13.3 ± 5.8% on day 2 and 33.3 ± 5.8% on day 3. Extending the exposure period to 72 h (Group II) significantly enhanced mortality, reaching 26.7 ± 5.7%, 50.0 ± 0.0%, and 83.3 ± 5.7% on days 1, 2, and 3, respectively.

Statistical analysis revealed significant differences among treatments, as indicated by the different superscript letters in Table 1 (*P* < 0.05). Mortality in the 72-h exposure treatment was significantly higher than that observed after 24 h of exposure on each assessment day, whereas the control remained significantly lower than all treated groups. Furthermore, within each treatment, mortality increased significantly over time, indicating that both prolonged exposure and longer post-treatment intervals contributed to enhanced insecticidal activity. Overall, these findings demonstrate that the crude extract of *S. marcescens* strain INS420 possesses potent insecticidal activity against fourth-instar *S. frugiperda* larvae in a time- and exposure-dependent manner.

**Table 1:** Time- and exposure-dependent mortality of fourth-instar *S. frugiperda* larvae following treatment with *S. marcescens* strain INS420 crude extract.

Treatment	Days post-treatment	Mortality (%) (mean ± SD)
Control group	Day 1	0.00 ± 0.00e
	Day 2	0.00 ± 0.00e
	Day 3	0.00 ± 0.00e
Group I	Day 1	6.7 ± 5.8de
	Day 2	13.3 ± 5.8d
	Day 3	33.3 ± 5.8c
Group II	Day 1	26.7 ± 5.7c
	Day 2	50.0 ± 0.0b
	Day 3	83.0 ± 5.7a

Values are presented as mean ± SD (*n* = 3). Means followed by different lowercase letters are significantly different according to Duncan’s Multiple Range Test (DMRT) at *P* < 0.05.

### Field efficacy of *S. marcescens* strain INS420 crude extract against *S. frugiperda* in maize fields

The efficacy of *S. marcescens* strain INS420 varied according to the assessment time and field location (Table 2). At Kafr El Akram, the reduction of infested plants (IP) increased from 35.00 ± 10.92% at 2 days after treatment to a maximum of 67.97 ± 7.16% at 5 days, before declining slightly to 58.18 ± 11.83% at 7 days. A comparable trend was observed for larval reduction (LC), which increased from 46.06 ± 16.30% at 2 days to 75.00 ± 5.88% at 5 days and then decreased slightly to 64.29 ± 12.79% at 7 days. Although these numerical variations suggest a peak response at 5 days after treatment, the differences in larval reduction among the assessment times were not statistically significant (*P* > 0.05), indicating that the observed fluctuations may reflect natural biological variability or uncontrolled environmental factors rather than a true change in treatment efficacy. Importantly, the absence of significant differences over the 7-day evaluation period also demonstrates that their insecticidal activity was sustained, indicating prolonged residual efficacy following application. Moreover, the similar larval reduction patterns observed at both Kafr El Akram and Ezbet Abu Garaf suggest comparable susceptibility of the target pest populations to the bacterial metabolites, highlighting the consistency of their field performance across different locations.

At Ezbet Abu Garaf, treatment efficacy against infested plants remained relatively stable throughout the evaluation period, recording 49.36 ± 15.19%, 51.95 ± 6.57%, and 58.61 ± 7.30% at 2, 5, and 7 days after treatment, respectively. Likewise, larval reduction ranged from 57.56 ± 11.20% to 60.10 ± 6.22% and 58.69 ± 6.52% over the same sampling intervals, indicating only minor temporal variation.

Statistical analysis using Duncan’s Multiple Range Test (*P* < 0.05) revealed no significant differences among values sharing the same lowercase superscript letter within each column. At Kafr El Akram, efficacy against infested plants at 5 days was significantly higher than that at 2 days, whereas the efficacy at 7 days did not differ significantly from either sampling time. In contrast, no significant temporal differences were detected for larval reduction at this location or for either assessment parameter at Ezbet Abu Garaf, where all observations shared the same superscript letter. Comparison between the two field sites revealed distinct response patterns. At Kafr El Akram, treatment efficacy peaked five days after application, suggesting a delayed but enhanced insecticidal effect under the environmental conditions of this location. Conversely, Ezbet Abu Garaf exhibited a more uniform level of efficacy across all assessment dates, indicating a stable response with no significant temporal variation. Overall, these findings demonstrate that while *S. marcescens* strain INS420 effectively reduced both plant infestation and larval populations under field conditions, the magnitude and temporal dynamics of its efficacy were influenced by field location.

**Table 2:** Field Efficacy of *S. marcescens* Strain INS420 Crude Extract against *S. frugiperda* Infestation and Larval Populations.

Days after treatment	Kafr El Akram		Ezbet Abu Garaf	
	IP	LC	IP	LC
2	35.00 ± 10.92^b^	46.06 ± 16.30^a^	49.36 ± 15.19^a^	57.56 ± 11.20^a^
5	67.97 ± 7.16^a^	75.00 ± 5.88^a^	51.95 ± 6.57^a^	60.10 ± 6.22^a^
7	58.18 ± 11.83^ab^	64.29 ± 12.79^a^	58.61 ± 7.30^a^	58.69 ± 6.52^a^
LSD (*P* = 0.05)	31.35	35.15	32.18	25.57

Means followed by different lowercase letters within the same column differ significantly according to Duncan’s Multiple Range Test (*P* ≤ 0.05). Means sharing at least one common letter are not significantly different. The least significant difference (LSD) at *P* = 0.05 is presented for each treatment at each location. IP = infested plants; LC = larval counts.

### GC–MS and LC–MS-based characterization of bioactive metabolites from *S. marcescens* crude extract

**Fig. 3:**
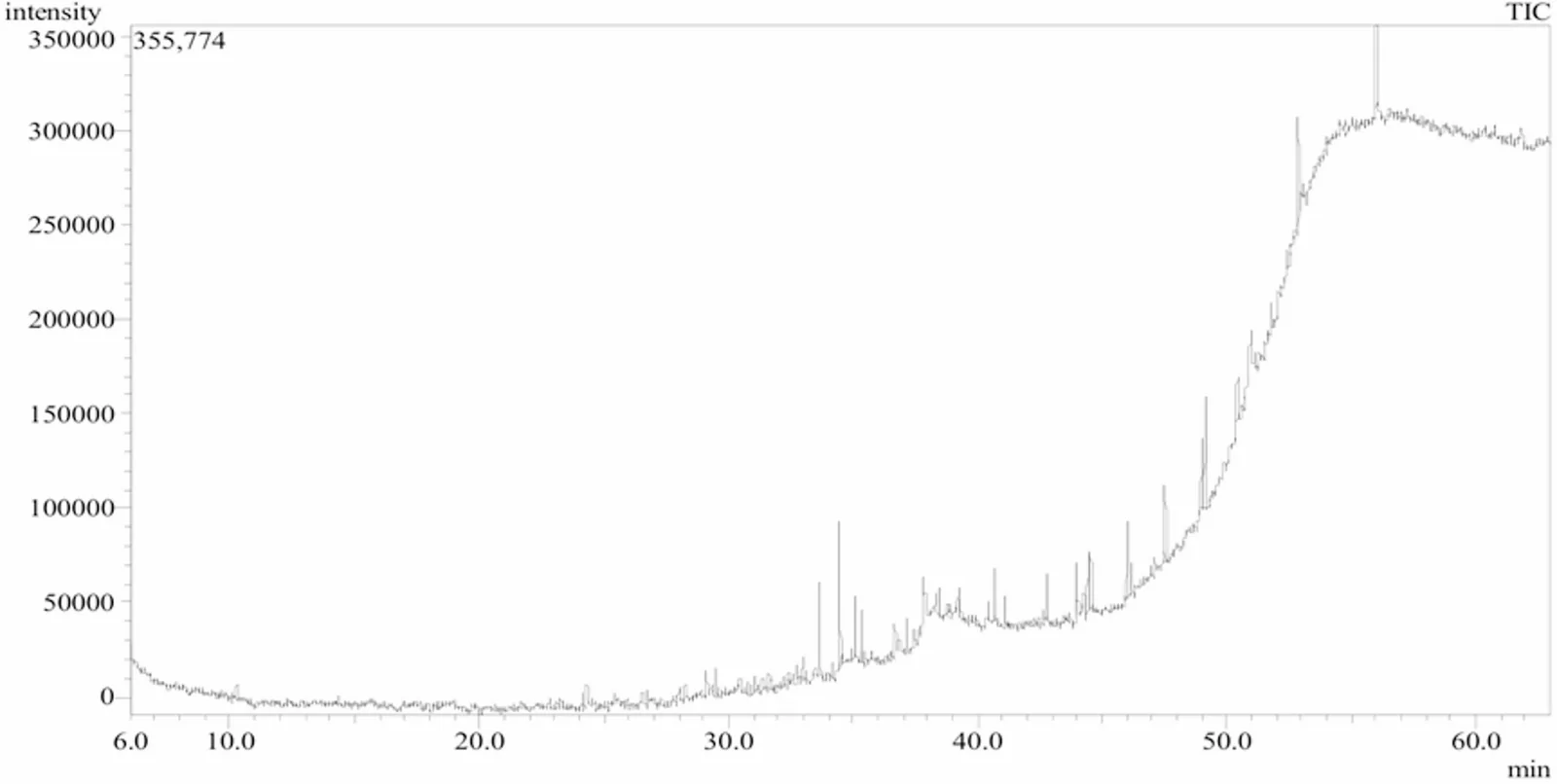
GC–MS chromatogram of bacterial extract.

**Fig. 4:**
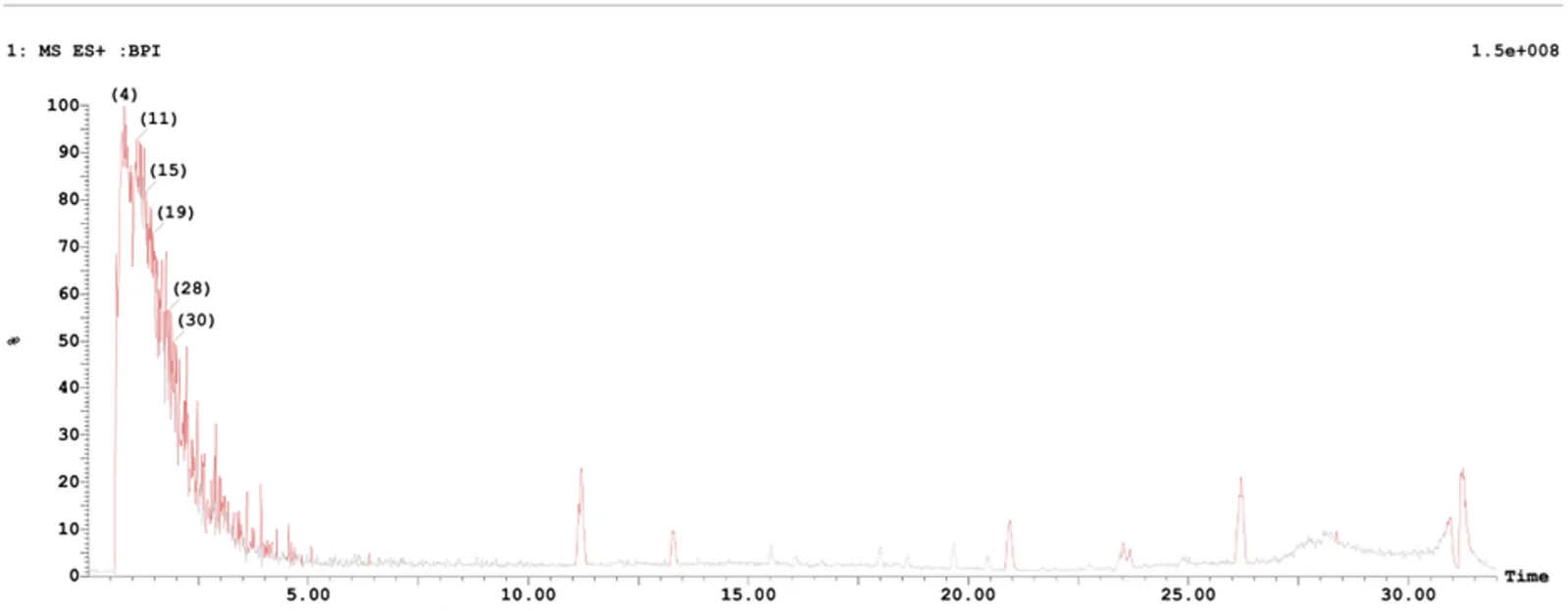
LC–MS chromatogram of bacterial extract.

As shown in Fig. 3, HS–GC–MS analysis reveals complementary data on primary metabolites, including fatty acids, esters, hydrocarbons, and sterols. Identified compounds included pentadecanoic acid derivatives, squalene, and cardenolide-like structures. The selected positive ion peaks detected by GC–MS are summarized in Table 3. In contrast, the LC–MS chromatogram (Fig. 4) exhibited more than 90 peaks. The most prominent peaks within the retention time range of 0.64–1.20 min are summarized in Table 4, showing ions with m/z values of 143, 170, 186, and 254. These ions are indicative of small nitrogen-containing metabolites, such as diketopiperazine derivatives. Additionally, peaks detected between 26 and 31 min displayed high m/z values (> 700), suggesting the presence of lipopeptide and/or macrolide-like compounds. Notably, these signals were absent in the solvent blank, confirming their biological origin and highlighting their potential biological significance.

**Table 3:** Selected GC–MS peaks of bacterial extract.

Peak no.	RT (min)	Compound	Area %
1	33.67	Pentadecanoic acid, methyl ester	7.18
2	34.46	Pentadecanoic acid	13.35
3	35.07	Pentadecanoic acid, ethyl ester	4.65
9	44.52	Bis(2-ethylhexyl) phthalate	4.85
13	49.13	Squalene	9.69
15	52.85	Card-20(22)-enolide derivative	10.02

**Table 4:** Selected LC–MS peaks of bacterial extract.

Peak no.	RT (min)	m/z (major)	Area %
1	0.64	143.99, 186.03	5.77
2	0.73	143.97, 254.12	6.71
3	0.76	143.96, 254.13	8.86
4	0.82	143.98, 186.06	4.27
5	0.85	143.98, 186.07	3.86
6	0.89	143.98, 186.06	5.54

### In silico analysis

#### Protein model validation

The quality of the target protein models was evaluated using Ramachandran plot and Verify3D analyses. The Verify3D assessment confirmed the reliability of the predicted structures, with an average 3D–1D score ≥ 0.1, indicating high overall structural accuracy. The Ramachandran plot results (Table 5) show that most residues were located within the favoured and allowed regions, confirming the stereochemical quality and stability of the model. These results validate the structural integrity of the predicted protein and its suitability for subsequent molecular docking analyses.

**Table 5:**
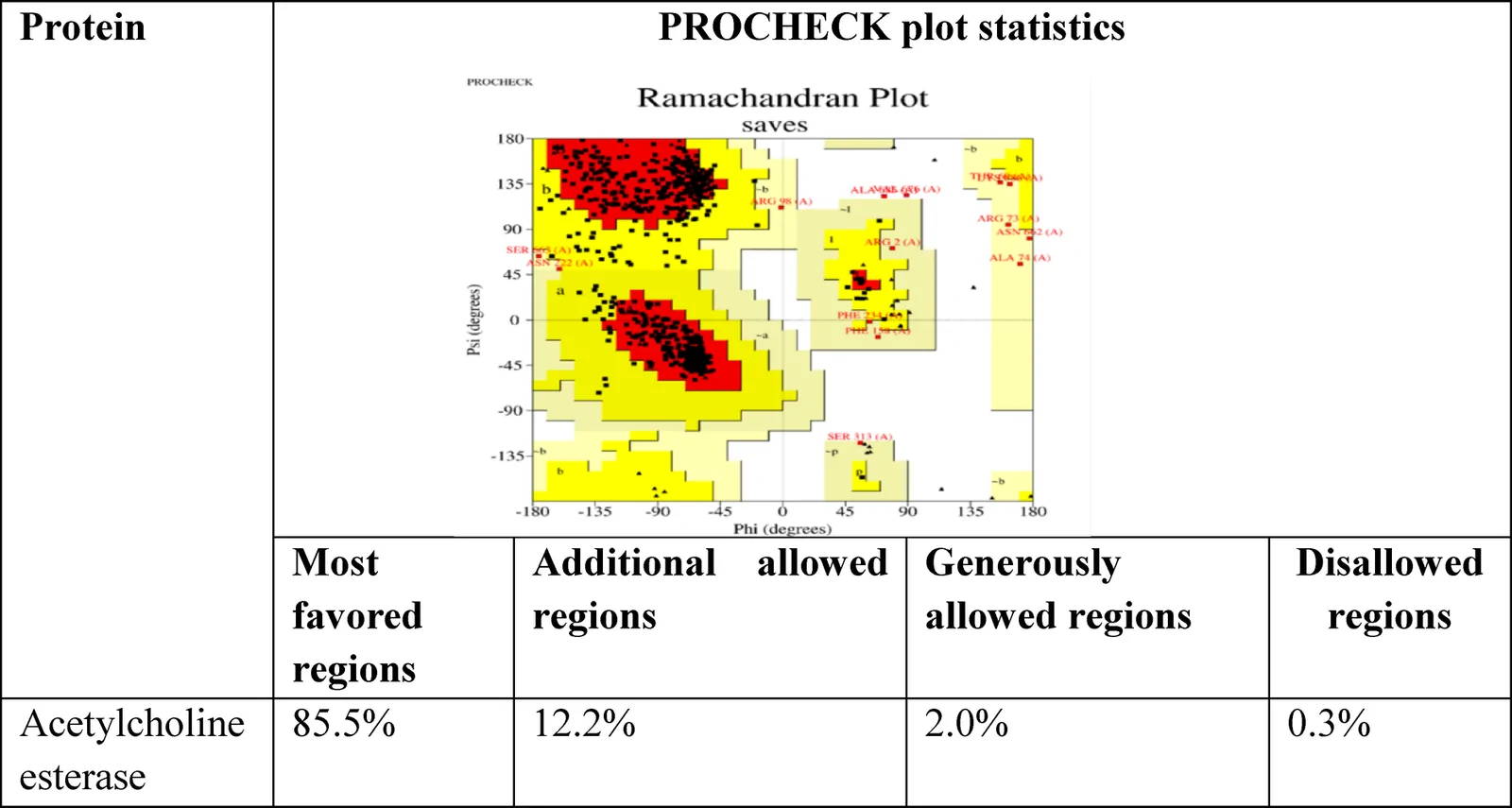
PROCHECK plot statistical analysis of protein target models.

### Molecular docking simulation of bioactive metabolites from *S. marcescens* against *S. frugiperda* acetylcholinesterase

The seven identified metabolites exhibited moderate to strong binding affinities toward acetylcholinesterase (AChE), suggesting their potential inhibitory activity against this key enzyme. Among them, squalene demonstrated the strongest interaction, with a binding energy of − 8.6 kcal/mol and an RMSD value of 1.4 Å (Table 6). Molecular docking simulations were conducted to investigate the potential interaction of secondary metabolites from *S. marcescens* with *S. frugiperda* acetylcholinesterase (AChE). The results revealed distinct variations in binding affinities and interaction patterns among the tested compounds (Table 6).

**Fig. 5:**
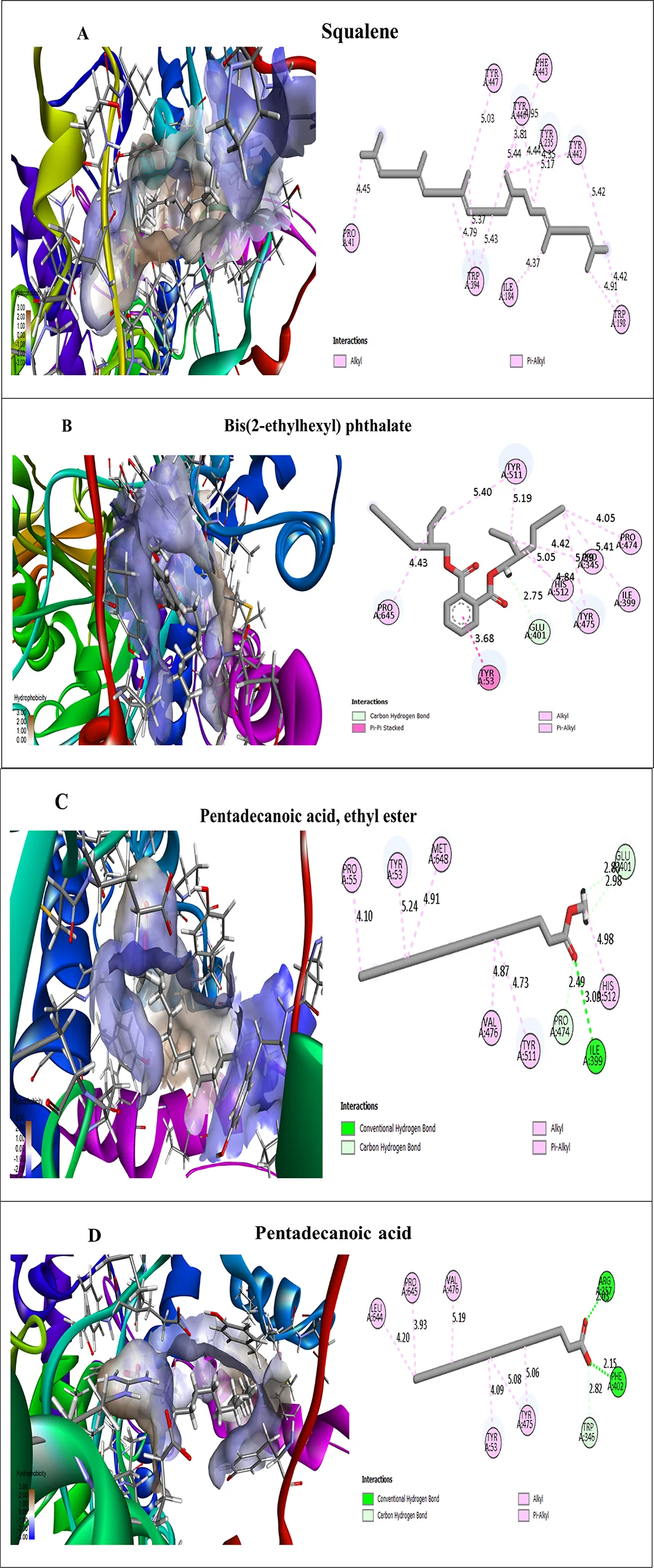

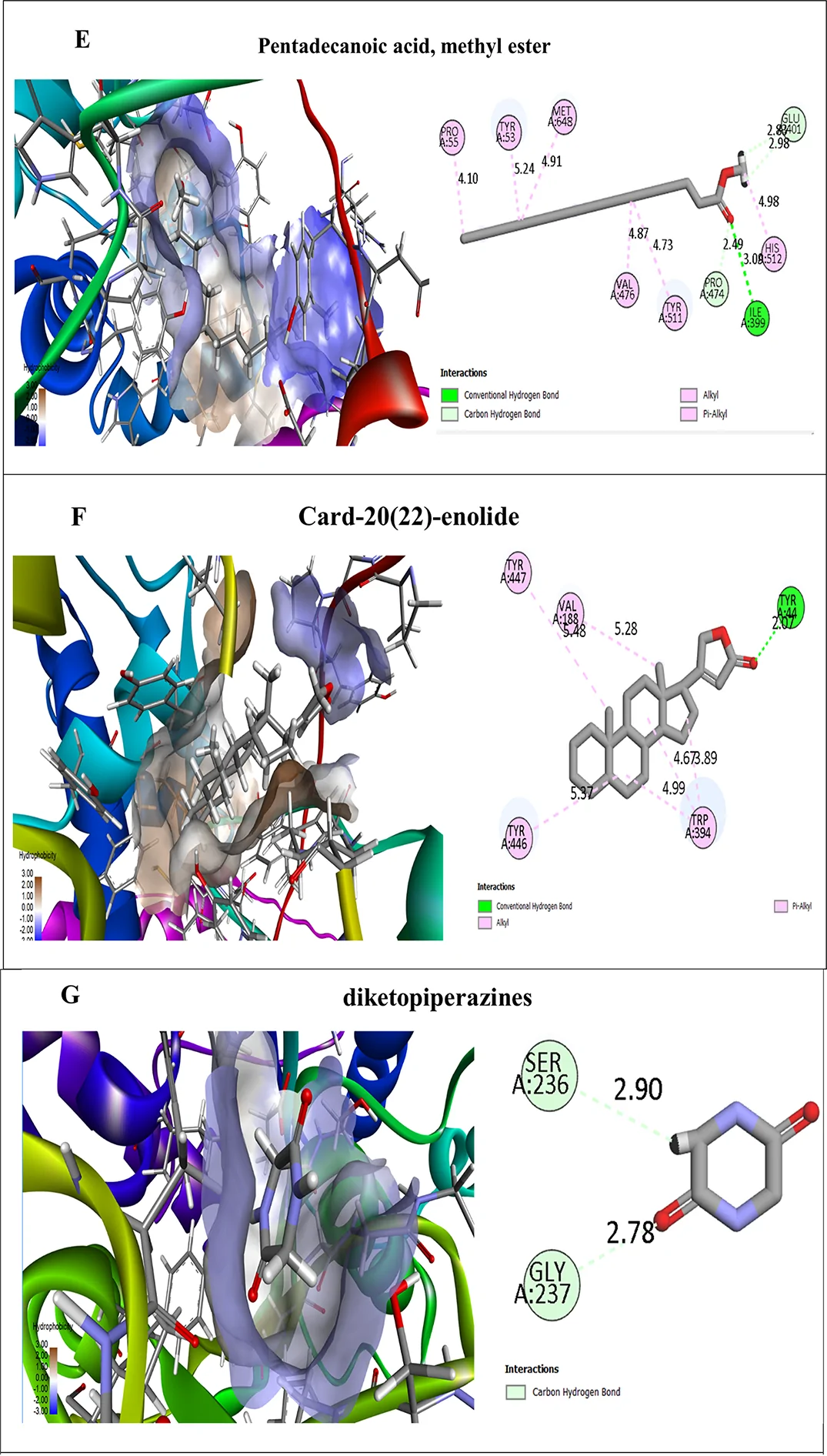
Docking interactions of identified metabolites with acetylcholinesterase active sites. (**A**) docking pose of Squalene; (**B**) docking pose of Bis(2-ethylhexyl) phthalate; (**C**) docking pose of Pentadecanoic acid ethyl ester; (**D**) docking pose of Pentadecanoic acid; (**E**) docking pose of Pentadecanoic acid, methyl ester; (**F**) docking pose of Card-20(22)-enolide; (**G**) docking pose of diketopiperazines.

Squalene exhibited the strongest binding affinity toward acetylcholinesterase (AChE), with its interaction primarily stabilized through extensive hydrophobic contacts within the active site (Fig. 5A). Among the fatty acid derivatives, bis(2-ethylhexyl) phthalate (Fig. 5B) and pentadecanoic acid, ethyl ester (Fig. 5C) showed high binding affinities of − 7.45 and − 7.3 kcal/mol, with RMSD values of 1.7 Å and 1.3 Å, respectively, indicating stable accommodation within the enzyme binding pocket. Pentadecanoic acid (Fig. 5D) and pentadecanoic acid, methyl ester (Fig. 5E) exhibited slightly lower but comparable binding energies of − 6.7 and − 6.4 kcal/mol, respectively, suggesting that methyl esterification had only a marginal effect on their interaction with AChE. Card-20(22)-enolide displayed a similar binding affinity (–6.6 kcal/mol), reflecting a moderate interaction with the catalytic pocket (Fig. 5F). In contrast, diketopiperazines demonstrated the weakest interaction, with a binding energy of − 4.4 kcal/mol and an RMSD of 0.95 Å (Fig. 5G), suggesting limited inhibitory potential.

Overall, the docking analysis indicates that the secondary metabolites identified from *S. marcescens* possess varying degrees of affinity toward AChE in *S. frugiperda*. The comparatively stronger interactions of squalene, bis(2-ethylhexyl) phthalate, and fatty acid derivatives suggest that these metabolites are the most promising candidates for further investigation as natural insecticidal agents and provide a plausible mechanistic explanation for the observed larvicidal activity.

**Table 6:** Molecular docking interactions of identified metabolites with the active-site residues of the acetylcholinesterase protein model.

Ligands	3D structure	Interaction affinity score (kcal/mol), RMSDValue (Å)	(Interactions other than hydrogen bond), (Hydrogen bonds, (amino acids involved in Hydrogen bonds, distance of hydrogen bond Å)
Diketopiperazines		− 4.40, 0.95	(Carbon hydrogen bond), No
Pentadecanoic acid, methyl ester		− 6.44, 1.6	Carbon hydrogen bond, Alkyl, and Pi alkyl, (yes, ILE399, 3)
Pentadecanoic acid, ethyl ester		− 7.30, 1.3	Carbon hydrogen bond, Alkyl, and Pi alkyl, (No)
Pentadecanoic acid		− 6.7, 1.5	Carbon hydrogen bond, Alkyl, and Pi alkyl, (yes, PHE402, 2.15)
Bis(2-ethylhexyl) phthalate		− 7.45, 1.7	Carbon hydrogen bond, Alkyl, Pi alkyl, and Pi-Pi stacked, No
Squalene		− 8.6, 1.4	Alkyl, and Pi alkyl
Card-20(22)-enolide		− 6.6, 1.2	Alkyl and Pi alkyl, (yes, TYR44, 2.07)

## Discussion

Importantly, this study provides a comprehensive characterization of metabolites produced by *S. marcescens* associated with *S. frugiperda* through the integration of metabolomic profiling, biological assays, and computational modeling. While previous studies have primarily focused on the pathogenicity of *S. marcescens* or on individual enzymatic components such as chitinases, the present work expands this perspective by linking the bacterial metabolome to its insecticidal activity. The combined use of GC–MS and LC–MS enabled the detection of a broader spectrum of metabolites than previously reported, capturing both volatile and non-volatile compounds. Furthermore, coupling metabolite identification with molecular docking analysis provided mechanistic insight into the potential inhibition of acetylcholinesterase, thereby bridging chemical characterization with functional biological interpretation. This integrative framework represents an important step toward understanding the chemical ecology of *S. marcescens*–FAW interactions and identifying candidate bioactive molecules for sustainable pest management.

Within this broader framework, it is essential to consider how these findings align with previous research on the entomopathogenic potential of *S. marcescens*. Previous studies have consistently demonstrated the insecticidal activity of *S. marcescens* or its derived products against the fall armyworm (FAW). For instance, Danai-Tambhale et al.^37^ reported that chitinolytic bacterial isolates obtained from soil, including *S. marcescens*, caused rapid larval mortality, with some strains inducing lethal effects within only a few hours of exposure. Similarly, El-Sayed et al.^10^, demonstrated that recombinant *S. marcescens*chitinase B exhibited strong insecticidal activity against FAW larvae, causing high mortality rates and significant developmental delays comparable to those produced by conventional chemical insecticides. Histopathological investigations by Guo et al.^2^, further showed that infection with live *S. marcescens* disrupts midgut tissues, leading to gut rupture, systemic infection, and eventual larval death. In addition to these direct pathogenic effects, enzyme-rich extracts of *S. marcescens* have also been reported to reduce FAW damage on maize plants under greenhouse conditions, resulting in substantial reductions in foliar injury^38^. Notably, the combination of Serratia-derived enzymatic extracts with botanical insecticides produced enhanced protective effects, suggesting potential synergistic interactions. Collectively, these studies support the growing consensus that *S. marcescens* represents a promising biological control agent for FAW management due to its ability to produce bioactive compounds and enzymes that disrupt larval development and survival across diverse experimental systems.

Consistent with these earlier reports, the present findings suggest that *S. marcescens* exerts a progressive and time-dependent insecticidal effect on *S. frugiperda*. Such delayed activity is characteristic of microbial and enzyme-based bioinsecticides. Mechanistic studies indicate that oral infection by *S. marcescens* disrupts midgut integrity and microbiota homeostasis, eventually leading to systemic infection and larval mortality^2^. In particular, bacterial chitinases have been proposed as a primary mode of action because these enzymes degrade chitin-containing structures such as the peritrophic membrane, thereby compromising the gut barrier and facilitating pathogen invasion^10^. These processes explain the gradual and cumulative mortality patterns typically observed following exposure to microbial bioinsecticides compared with the rapid knockdown associated with chemical pesticides.

While laboratory studies provide valuable mechanistic insights, the performance of microbial agents under field conditions is often influenced by environmental and ecological variables. Field evaluations therefore provide an essential perspective on the practical applicability of these biological agents. In the present study, the effectiveness of *S. marcescens* extracts appeared to be influenced by environmental conditions, host plant characteristics, and initial pest pressure. Variations in plant age, microclimatic conditions, and infestation intensity may affect the extent and consistency of larval suppression, reflecting the complex ecological interactions between insect hosts, microbial pathogens, and host plants^39^. These observations are consistent with greenhouse studies indicating that enzymatic or microbial extracts tend to provide greater protection when applied repeatedly or at higher concentrations, particularly when host plants are under stress or exhibit enhanced defensive responses^38^.

Furthermore, the variability observed in field performance indicates that the crude extract produced a rapid insecticidal effect within 48 h that was sustained throughout the evaluation period. This temporal stability suggests that the bioactive metabolites remained effective under field conditions, providing prolonged larval suppression. Such rapid and persistent activity is a desirable attribute of microbial biopesticides, although their residual efficacy may ultimately be influenced by environmental factors, including ultraviolet radiation, temperature, rainfall, and microbial degradation^40^. The stable efficacy observed for the LC treatment also suggests that the active compounds produced by strain INS420 were sufficiently persistent to withstand the environmental conditions encountered during the experimental period. In contrast to many microbial biocontrol agents whose activity declines rapidly after field application because of photodegradation and environmental stress, the present crude extract maintained comparable efficacy throughout the assessment period. Comparable findings have been reported for microbial formulations in which appropriate formulations or stable bioactive metabolites prolonged insecticidal activity under field conditions^41^. The significant increase in efficacy of the IP treatment at 5 days after application at Kafr El Akram, compared with day 2, indicates that this formulation required a longer period to reach maximum biological activity. Such delayed responses have been described for several microbial insecticides, in which the infection process, toxin accumulation, or gradual exposure of target insects results in increasing mortality several days after application. The lack of similar temporal variation at Ezbet Abu Garaf further suggests that local environmental conditions may influence the rate of treatment activity while having little effect on its overall efficacy^42^.

Several studies have suggested that the efficacy of *S. marcescens* can be influenced by additional factors such as larval feeding behaviour, environmental stressors, or co-application with other control agents. Consequently, microbial bioinsecticides are unlikely to function as standalone solutions. Instead, they are most effective when incorporated into integrated pest management (IPM) programs, where their activity complements chemical, botanical, or other biological control methods^43^. Such integrated strategies may enhance overall pest suppression while reducing reliance on synthetic pesticides.

To further elucidate the biochemical basis underlying the observed insecticidal activity, comprehensive metabolite profiling was conducted. Previous investigations of Serratia metabolites have largely focused on individual compounds or relied on a single analytical platform, most commonly GC–MS or LC–MS. In contrast, the present study employed a complementary metabolomic strategy integrating both techniques, thereby enabling the detection of a broader spectrum of metabolites, including volatile and lipophilic compounds as well as non-volatile and more polar metabolites. This combined analytical approach provided a more comprehensive overview of the secondary metabolome of the bacterium associated with *S. frugiperda*. To our knowledge, this work represents one of the first detailed chemical characterizations of metabolites produced by a bacterium associated with the fall armyworm. The analysis confirmed the presence of several classes of known bioactive metabolites, including fatty acid derivatives, lipopeptides, and diketopiperazines, which have previously been reported to exhibit insecticidal, antimicrobial, or signaling activities in microbial–insect interactions. In addition, the detection of less commonly reported metabolites, such as a cardenolide-like compound, suggests the presence of previously unexplored chemical diversity that may contribute to the observed biological activity. Collectively, these findings highlight the value of integrating multiple advanced analytical platforms for comprehensive metabolite profiling and provide new insights into the chemical ecology and insecticidal potential of *S. marcescens* associated with FAW.

To explore the possible molecular mechanisms underlying these biological effects, molecular docking analysis was performed targeting acetylcholinesterase (AChE), a key enzyme in the insect nervous system. Acetylcholinesterase is responsible for hydrolyzing the neurotransmitter acetylcholine, thereby terminating nerve impulses. Inhibition of this enzyme disrupts neural transmission, leading to overstimulation, paralysis, and ultimately death. Several metabolites identified in this study, including pentadecanoic acid and its derivatives (pentadecanoic acid ethyl ester and pentadecanoic acid methyl ester), as well as card-20(22)-enolide, were predicted to form hydrogen bonds with key residues within the AChE active site, highlighting their potential as candidate compounds for insecticidal development targeting *S. frugiperda*. Similar findings have been reported in studies investigating plant-derived insecticidal compounds. For example, Kobenan et al.^44^, demonstrated the larvicidal efficacy of aromatic plant extracts through both experimental bioassays and molecular docking analyses, showing that compounds such as p-cymene and thymol exhibit strong hydrophobic interactions with acetylcholinesterase. Likewise, Zheng et al.^45^, employed molecular docking to investigate the interactions between chemical insecticides and both wild-type and mutant forms of AChE in FAW, providing insights into the molecular basis of emerging insecticide resistance. These studies collectively support the relevance of AChE as a key molecular target in the development of novel bio-insecticidal compounds.

## Conclusion

This study provides a comprehensive characterization of metabolites produced by *S. marcescens* associated with *S. frugiperda*, integrating metabolomic profiling, biological assays, and molecular docking analysis. Unlike previous research focused primarily on pathogenicity or individual enzymes, this work links the bacterial metabolome to its insecticidal activity, revealing both volatile and non-volatile compounds with potential bioactivity. Mechanistic insights from docking studies suggest that several metabolites, including fatty acid derivatives and a cardenolide-like compound, may inhibit acetylcholinesterase, offering a plausible molecular basis for observed larval mortality. These findings are consistent with prior reports on the entomopathogenic potential of *S. marcescens*, confirming its progressive, time-dependent effects on FAW larvae through gut disruption and enzymatic activity. While laboratory assays highlight clear bio-insecticidal potential, field performance is influenced by environmental conditions and host-plant interactions, emphasizing the need to integrate microbial agents within broader pest management strategies. Overall, this integrative approach not only expands the chemical and functional understanding of *S. marcescens*–FAW interactions but also identifies candidate metabolites for the development of sustainable, biologically based pest control solutions.

## Supplementary Information

Below is the link to the electronic supplementary material.

Supplementary Material 1

Supplementary Material 2

Supplementary Material 3

## Data Availability

The data supporting the findings of this study are available within the article and its Supplementary Materials. Raw data files have been uploaded as supplementary files and are available from the corresponding author upon reasonable request.
