# Supplementary material for: Insecticidal metabolites from Serratia marcescens associated with the fall armyworm (Spodoptera frugiperda): molecular identification, metabolomic profiling and molecular docking insights
Source: Sci Rep. 2026 Aug 19;16:26057. doi: 10.1038/s41598-026-66375-7 (PMC13490546; doi:10.1038/s41598-026-66375-7)
Supplement: Supplementary file 1 [file 41598_2026_66375_MOESM1_ESM.pdf]

# Ain Shams University-Faculty of Pharmacy
# Center for Drug Discovery Research and Development
# HS-GC-MS

Sample Information

| | |
|---|---|
| Analyzed by | : Admin |
| Analyzed | : 11/18/2024 9:55:09 AM |
| Sample Type | : Unknown |
| Level # | : 1 |
| Sample Name | : 1 Dr.Ghada |
| Sample ID | : 010 |
| IS Amount | : [1]=1 |
| Sample Amount | : 1 |
| Dilution Factor | : 1 |
| Vial # | : 1 |
| Injection Volume | : 1.00 |
| Data File | : C:\GCMSsolution\Data\Project1\1 Dr.Ghada _010_11182024_10.qgd |
| Org Data File | : C:\GCMSsolution\Data\Project1\1 Dr.Ghada _010_11182024_10.qgd |
| Method File | : C:\GCMSsolution\Method file\Hexane -DCM split 5  IA.qgm |
| Org Method File | : C:\GCMSsolution\Method file\Hexane -DCM split 5  IA.qgm |
| Report File | : |
| Tuning File | : C:\GCMSsolution\System\Tune1\DFTPP_QP2020.qgt |
| Modified by | : Admin |
| Modified | : 11/18/2024 10:59:34 AM |

Chromatogram 1 Dr.Ghada

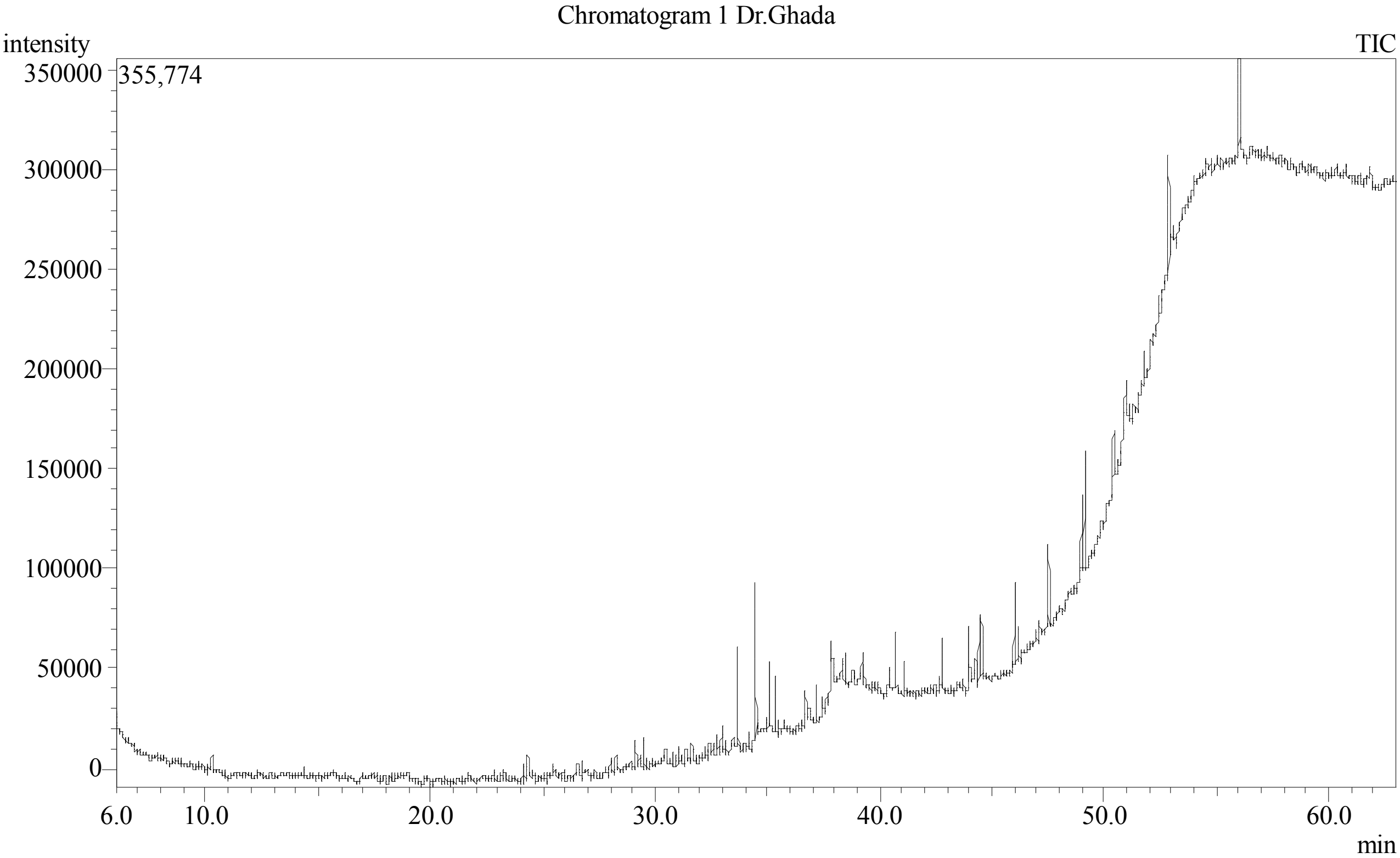

Chromatogram 1 Dr.Ghada

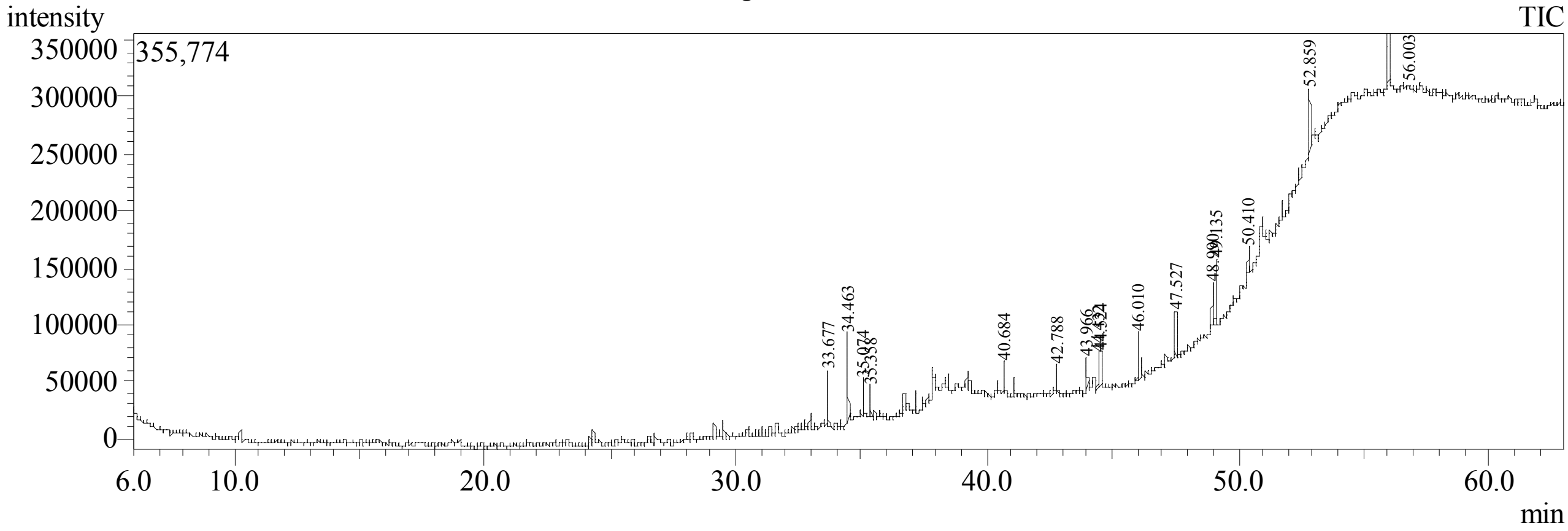


Peak Report TIC

| Peak# | R.Time | Area | Area% | Name | Base m/z |
|---|---|---|---|---|---|
| 1 | 33.677 | 129135 | 7.18 | Pentadecanoic acid, methyl ester | 87.10 |
| 2 | 34.463 | 239965 | 13.35 | Pentadecanoic acid | 73.10 |
| 3 | 35.074 | 83623 | 4.65 | Pentadecanoic acid, ethyl ester | 88.15 |
| 4 | 35.358 | 66033 | 3.67 | 6-Octadecenoic acid, methyl ester, (Z)- | 55.10 |
| 5 | 40.684 | 74502 | 4.15 | 2-Propenoic acid, 3-(4-methoxyphenyl)-, 2-ethylhexyl ester | 178.15 |
| 6 | 42.788 | 59999 | 3.34 | Heneicosane | 85.20 |
| 7 | 43.966 | 64906 | 3.61 | | 117.15 |
| 8 | 44.432 | 81803 | 4.55 | Tetratetracontane | 85.20 |
| 9 | 44.524 | 87160 | 4.85 | Bis(2-ethylhexyl) phthalate | 149.10 |
| 10 | 46.010 | 131870 | 7.34 | Tetratetracontane | 85.20 |
| 11 | 47.527 | 102862 | 5.72 | Tetracontane | 85.20 |
| 12 | 48.990 | 94507 | 5.26 | Pentatriacontane | 85.20 |
| 13 | 49.135 | 174145 | 9.69 | Squalene | 81.15 |
| 14 | 50.410 | 57076 | 3.18 | Pentatriacontane | 85.20 |
| 15 | 52.859 | 180110 | 10.02 | Card-20(22)-enolide, 3,5,14,19-tetrahydroxy-, (3.beta.,5.beta.)- | 81.15 |
| 16 | 56.003 | 169677 | 9.44 | Card-20(22)-enolide, 3,5,14,19-tetrahydroxy-, (3.beta.,5.beta.)- | 107.20 |
| | | 1797373 | 100.00 | | |

Library

<< Target >>
Line#:1 R.Time:33.675(Scan#:5536) Retention Index:1904 MassPeaks:117
RawMode:Averaged 33.670-33.680(5535-5537) BasePeak:87.10(10961)
BG Mode:Calc. from Peak Group 1 - Event 1 Scan

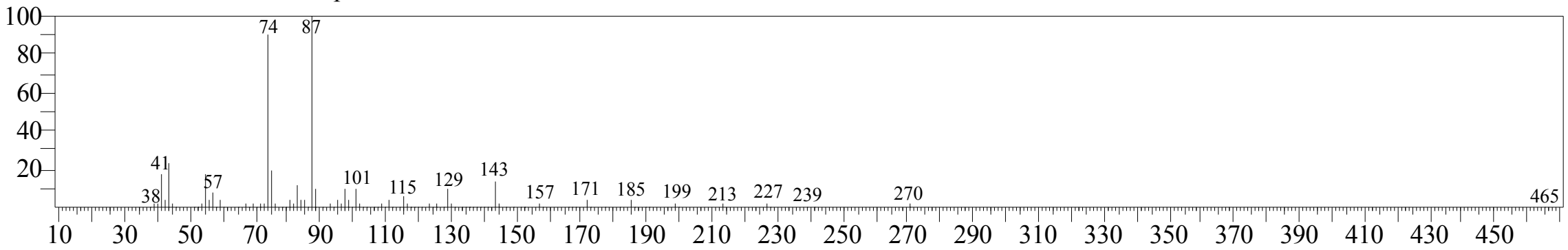


Hit#:1 Entry:99980 Library:NIST17-1.lib
SI:89 Formula:C16H32O2 CAS:7132-64-1 MolWeight:256 RetIndex:1779
CompName:Pentadecanoic acid, methyl ester $$ Methyl n-pentadecanoate $$ Methyl pentadecanoate $$ n-Pentadecanoic acid methyl ester $$

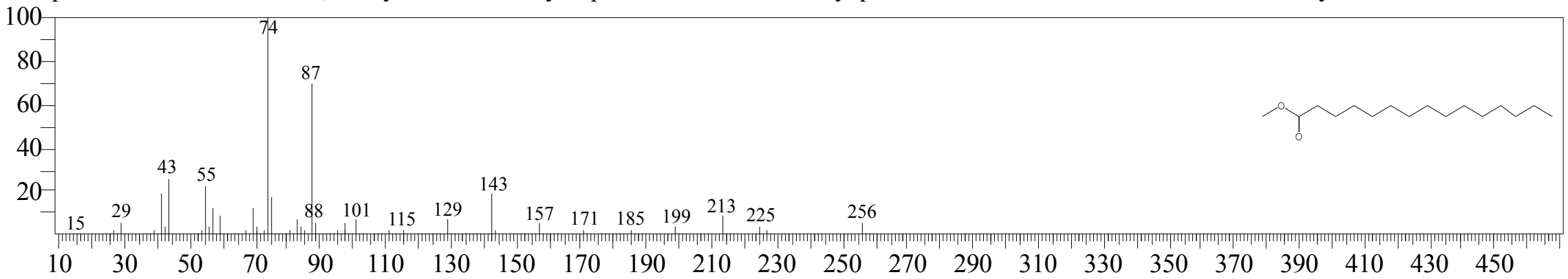


Hit#:2 Entry:30647 Library:NIST17s.lib
SI:89 Formula:C17H34O2 CAS:112-39-0 MolWeight:270 RetIndex:1878
CompName:Hexadecanoic acid, methyl ester $$ Palmitic acid, methyl ester $$ n-Hexadecanoic acid methyl ester $$ Metholene 2216 $$ Methyl hexadecano

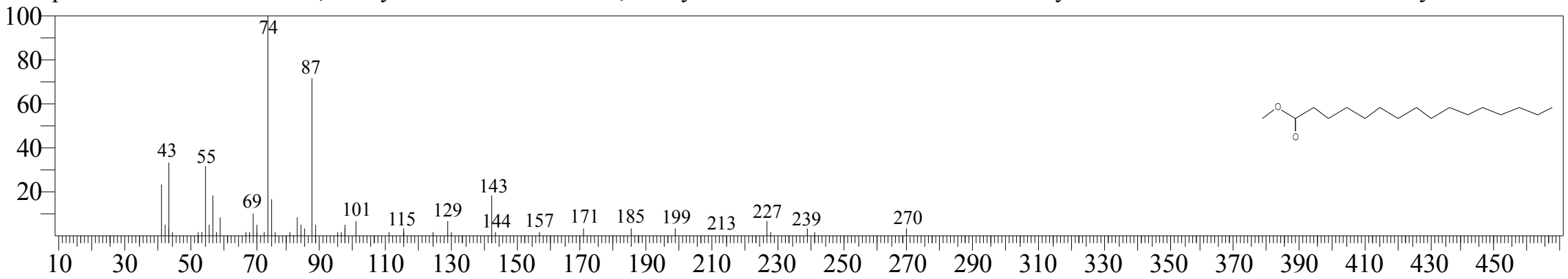


Hit#:3 Entry:74180 Library:NIST17-1.lib
SI:89 Formula:C14H28O2 CAS:1731-88-0 MolWeight:228 RetIndex:1580
CompName:Tridecanoic acid, methyl ester $$ Methyl tridecanoate $$ n-Tridecanoic acid methyl ester $$ Methyl ester of tridecanoic acid $$ Methyl n-tridec

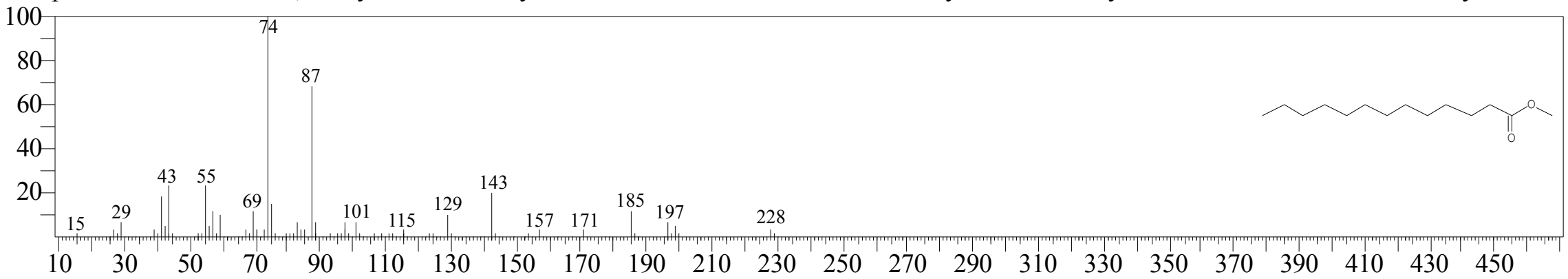


Hit#:4 Entry:24159 Library:NIST17s.lib
SI:88 Formula:C13H26O2 CAS:111-82-0 MolWeight:214 RetIndex:1481
CompName:Dodecanoic acid, methyl ester $$ Lauric acid, methyl ester $$ Metholene 2296 $$ Methyl dodecanoate $$ Methyl dodecylate $$ Methyl laurate

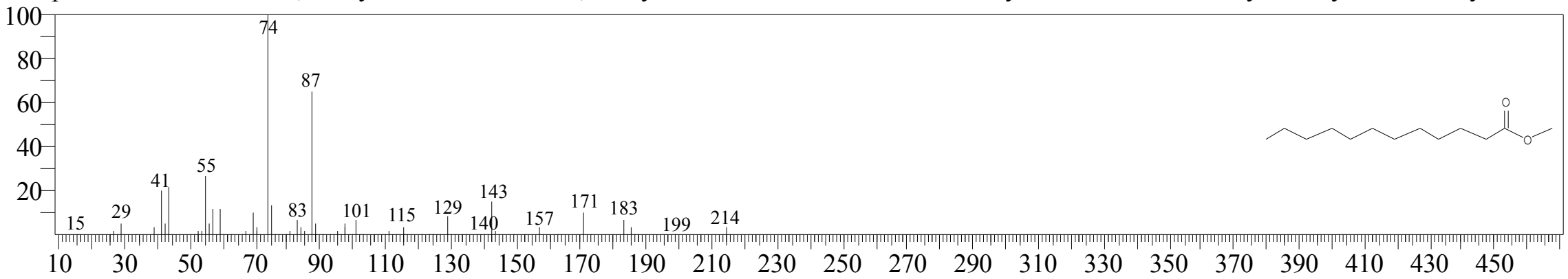


Hit#:5 Entry:127712 Library:NIST17-1.lib
SI:87 Formula:C18H36O2 CAS:1731-92-6 MolWeight:284 RetIndex:1978
CompName:Heptadecanoic acid, methyl ester $$ Margaric acid methyl ester $$ Methyl heptadecanoate $$ Methyl margarate $$ n-Heptadecanoic acid methy

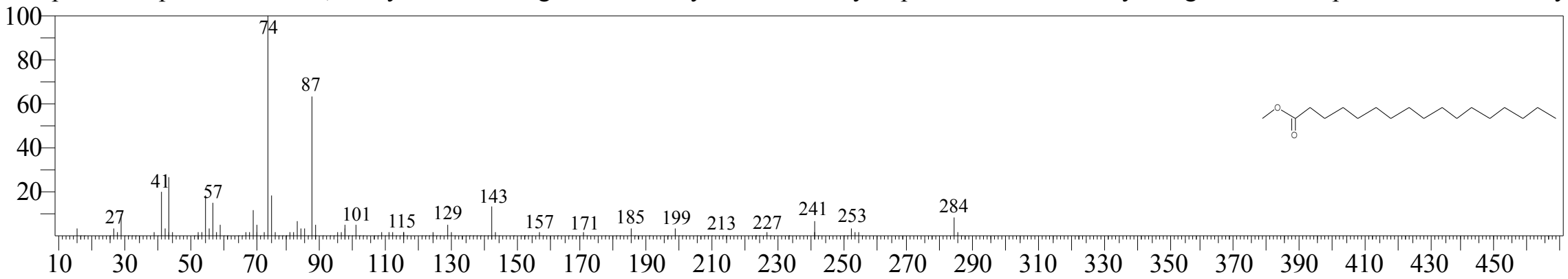

<< Target >>
Line#:2   R.Time:34.465(Scan#:5694) Retention Index:1943 MassPeaks:123
RawMode:Averaged 34.460-34.470(5693-5695) BasePeak:73.10(5918)
BG Mode:Calc. from Peak  Group 1 - Event 1  Scan

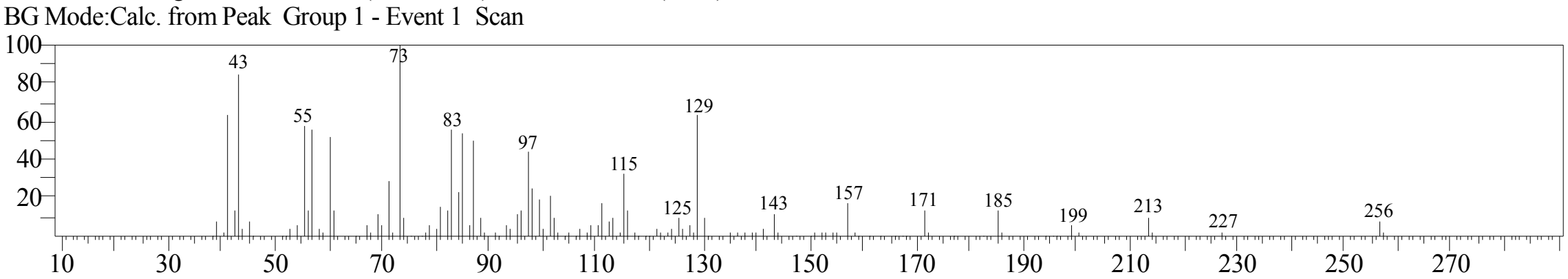


Hit#:1  Entry:27872  Library:NIST17s.lib
SI:87  Formula:C15H30O2  CAS:1002-84-2  MolWeight:242  RetIndex:1869
CompName:Pentadecanoic acid $$ Pentadecylic acid $$ n-Pentadecanoic acid $$ n-Pentadecylic acid $$

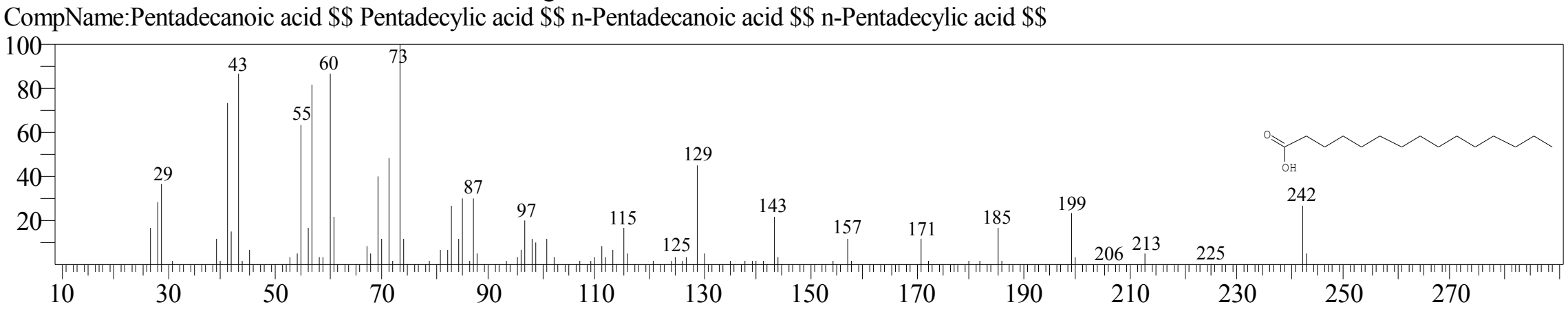


Hit#:2  Entry:29349  Library:NIST17s.lib
SI:87  Formula:C16H32O2  CAS:57-10-3  MolWeight:256  RetIndex:1968
CompName:n-Hexadecanoic acid $$ Hexadecanoic acid $$ n-Hexadecoic acid $$ Palmitic acid $$ Pentadecanecarboxylic acid $$ 1-Pentadecanecarboxylic

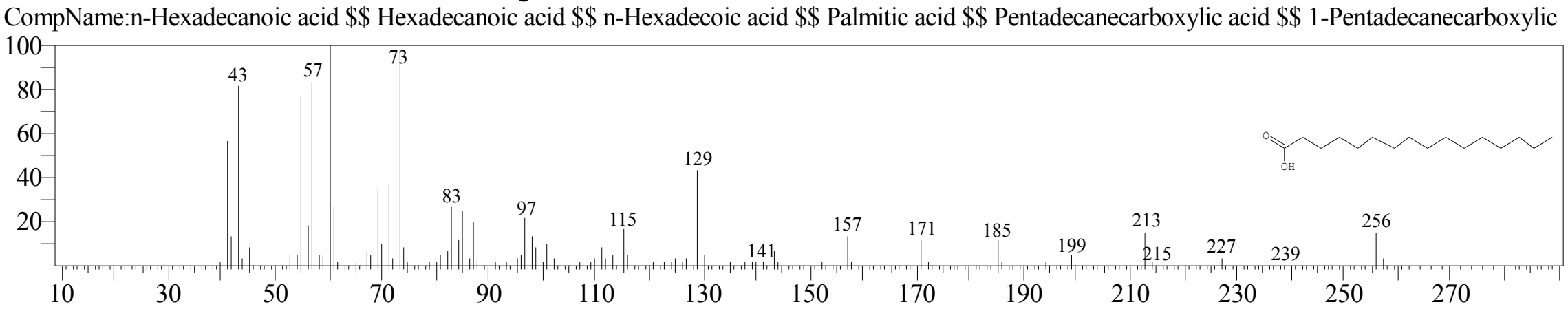


Hit#:3  Entry:24153  Library:NIST17s.lib
SI:86  Formula:C13H26O2  CAS:638-53-9  MolWeight:214  RetIndex:1670
CompName:Tridecanoic acid $$ n-Tridecanoic acid $$ n-Tridecoic acid $$ Tridecylic acid $$

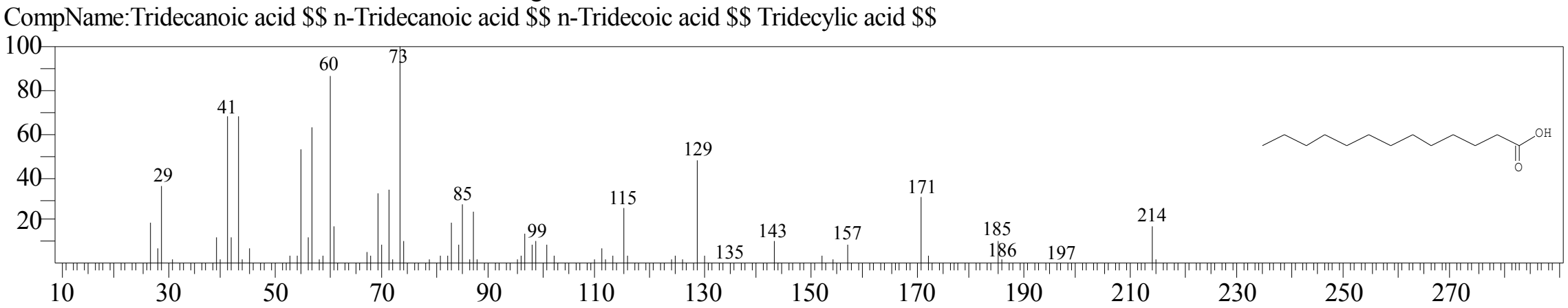


Hit#:4  Entry:74178  Library:NIST17-1.lib
SI:85  Formula:C14H28O2  CAS:544-63-8  MolWeight:228  RetIndex:1769
CompName:Tetradecanoic acid $$ Myristic acid $$ n-Tetradecanoic acid $$ n-Tetradecoic acid $$ Neo-Fat 14 $$ Univol U 316S $$ 1-Tridecanecarboxylic a

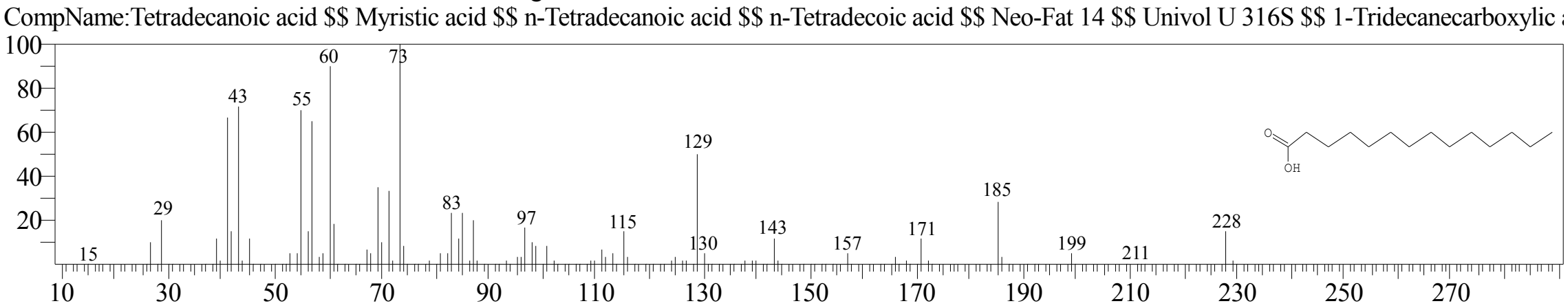


Hit#:5  Entry:127688  Library:NIST17-1.lib
SI:84  Formula:C18H36O2  CAS:57-11-4  MolWeight:284  RetIndex:2167
CompName:Octadecanoic acid $$ Stearic acid $$ n-Octadecanoic acid $$ Humko Industrene R $$ Hydrofol Acid 150 $$ Hystrene S-97 $$ Hystrene T-70 $

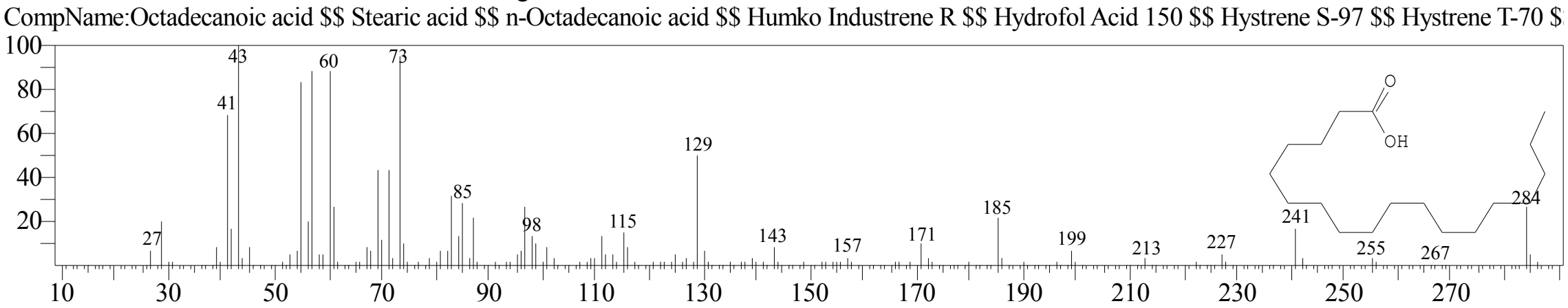

<< Target >>
Line#:3   R.Time:35.075(Scan#:5816) Retention Index:1973 MassPeaks:108
RawMode:Averaged 35.070-35.080(5815-5817) BasePeak:88.15(9124)
BG Mode:Calc. from Peak  Group 1 - Event 1  Scan

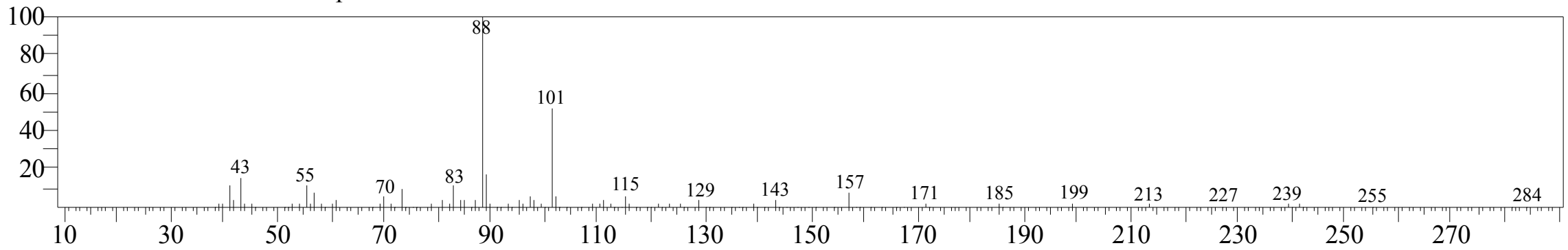


Hit#:1  Entry:30652  Library:NIST17s.lib
SI:87  Formula:C17H34O2  CAS:41114-00-5  MolWeight:270  RetIndex:1878
CompName:Pentadecanoic acid, ethyl ester $$ Ethyl pentadecanoate $$ n-Pentadecanoic acid ethyl ester $$

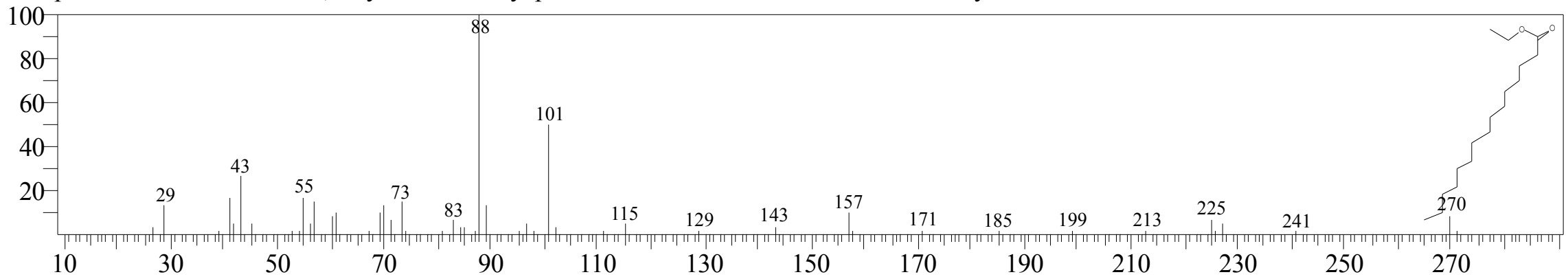


Hit#:2  Entry:29360  Library:NIST17s.lib
SI:87  Formula:C16H32O2  CAS:124-06-1  MolWeight:256  RetIndex:1779
CompName:Tetradecanoic acid, ethyl ester $$ Myristic acid, ethyl ester $$ Ethyl myristate $$ Ethyl tetradecanoate $$ Ethyl ester of tetradecanoic acid $$ Et

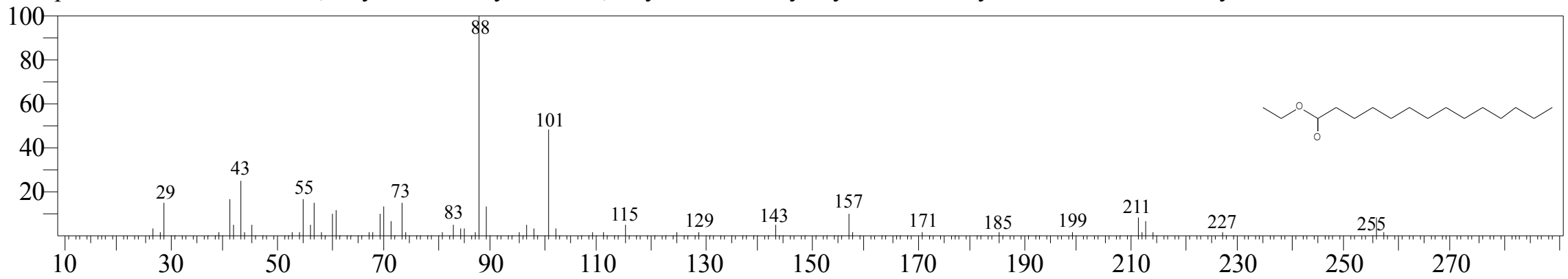


Hit#:3  Entry:27879  Library:NIST17s.lib
SI:86  Formula:C15H30O2  CAS:28267-29-0  MolWeight:242  RetIndex:1680
CompName:Ethyl tridecanoate $$ Tridecanoic acid ethyl ester $$ n-Tridecanoic acid ethyl ester $$

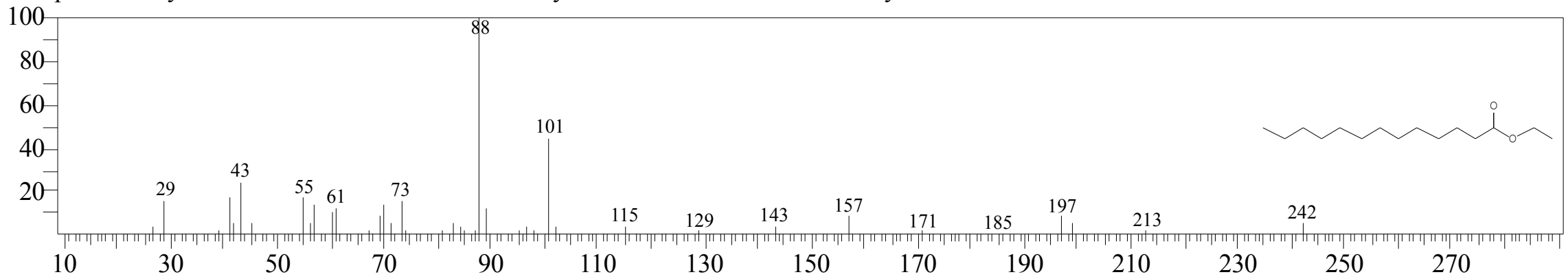


Hit#:4  Entry:26240  Library:NIST17s.lib
SI:86  Formula:C14H28O2  CAS:106-33-2  MolWeight:228  RetIndex:1580
CompName:Dodecanoic acid, ethyl ester $$ Lauric acid, ethyl ester $$ Ethyl dodecanoate $$ Ethyl dodecylate $$ Ethyl laurate $$ Ethyl laurinate $$ Ethyl n-

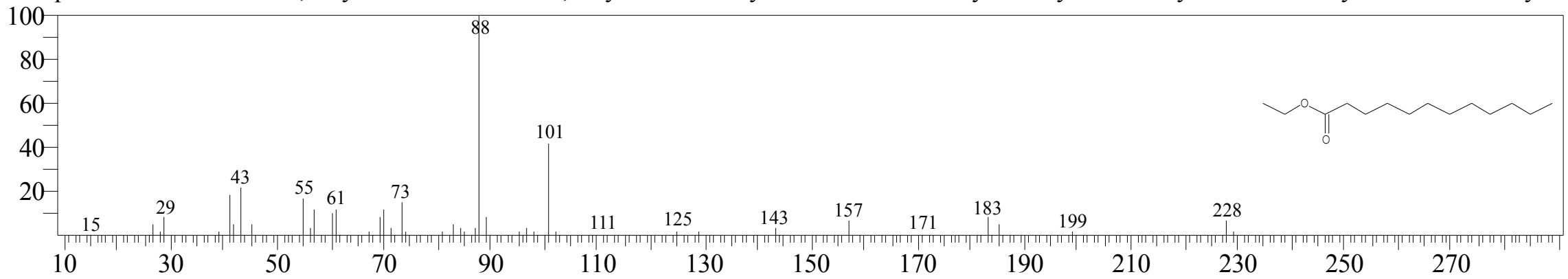


Hit#:5  Entry:31791  Library:NIST17s.lib
SI:86  Formula:C18H36O2  CAS:628-97-7  MolWeight:284  RetIndex:1978
CompName:Hexadecanoic acid, ethyl ester $$ Palmitic acid, ethyl ester $$ Ethyl hexadecanoate $$ Ethyl palmitate $$ Ethyl n-hexadecanoate $$

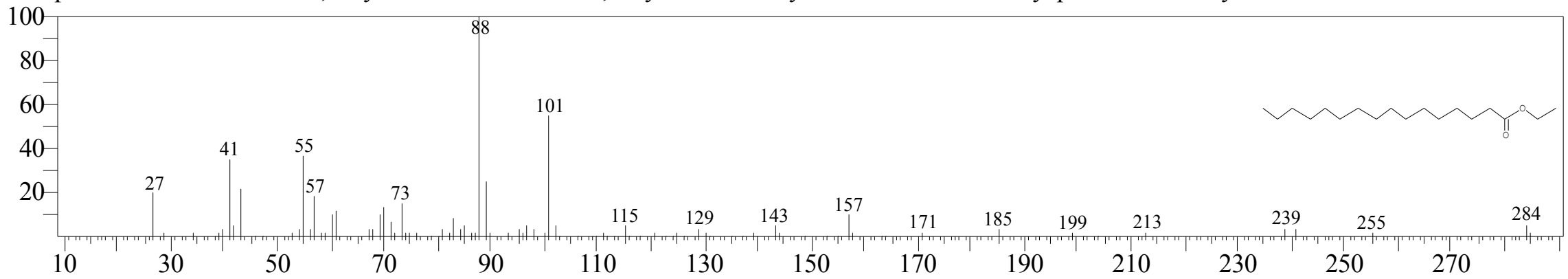

<< Target >>
Line#:4   R.Time:35.360(Scan#:5873) Retention Index:1987 MassPeaks:114
RawMode:Averaged 35.355-35.365(5872-5874) BasePeak:55.10(1546)
BG Mode:Calc. from Peak  Group 1 - Event 1  Scan

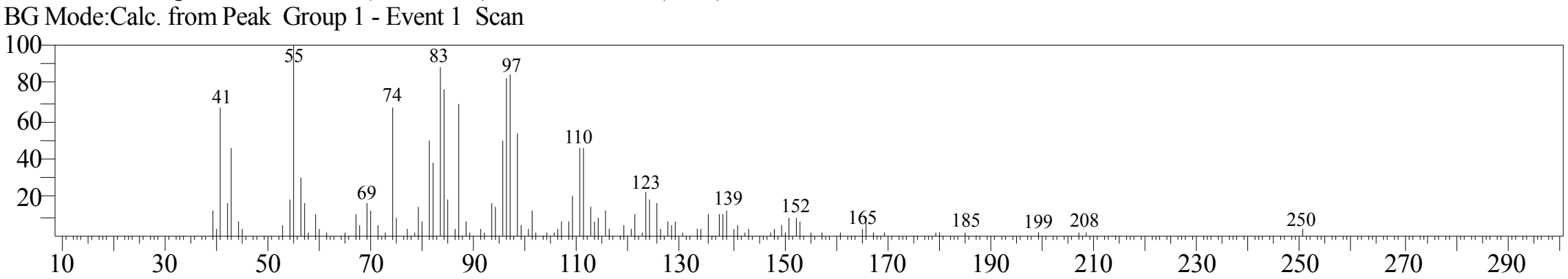


Hit#:1  Entry:139641  Library:NIST17-1.lib
SI:85  Formula:C19H36O2  CAS:2777-58-4  MolWeight:296  RetIndex:2085
CompName:6-Octadecenoic acid, methyl ester, (Z)- $$ Methyl cis-6-octadecenoate $$ Methyl petroselinate $$ Methyl (6Z)-6-octadecenoate # $$ cis-6-Octa

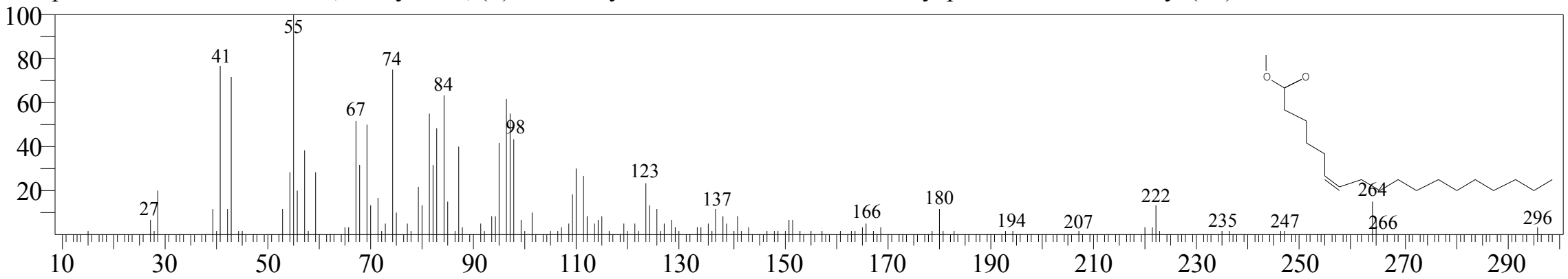


Hit#:2  Entry:30412  Library:NIST17s.lib
SI:81  Formula:C17H32O2  CAS:56875-67-3  MolWeight:268  RetIndex:1886
CompName:7-Hexadecenoic acid, methyl ester, (Z)- $$ Methyl (7E)-7-hexadecenoate # $$

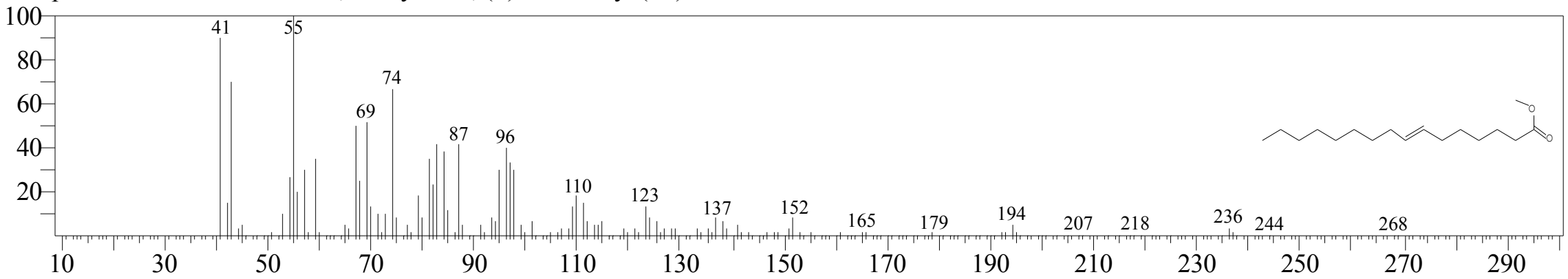


Hit#:3  Entry:137670  Library:NIST17-1.lib
SI:80  Formula:C19H34O2  CAS:0-00-0  MolWeight:294  RetIndex:2075
CompName:17-Octadecynoic acid, methyl ester

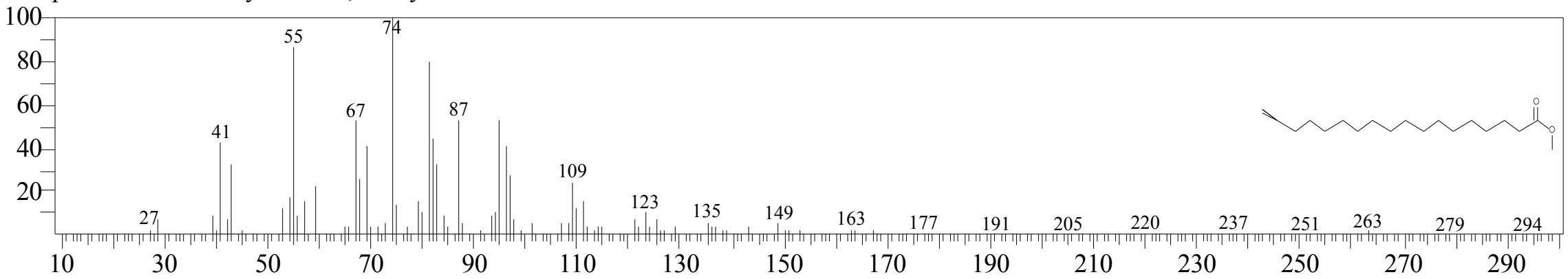


Hit#:4  Entry:72106  Library:NIST17-1.lib
SI:79  Formula:C13H22O3  CAS:22418-58-2  MolWeight:226  RetIndex:1677
CompName:Methyl 12-oxo-9-dodecenoate $$ Methyl (9E)-12-oxo-9-dodecenoate # $$

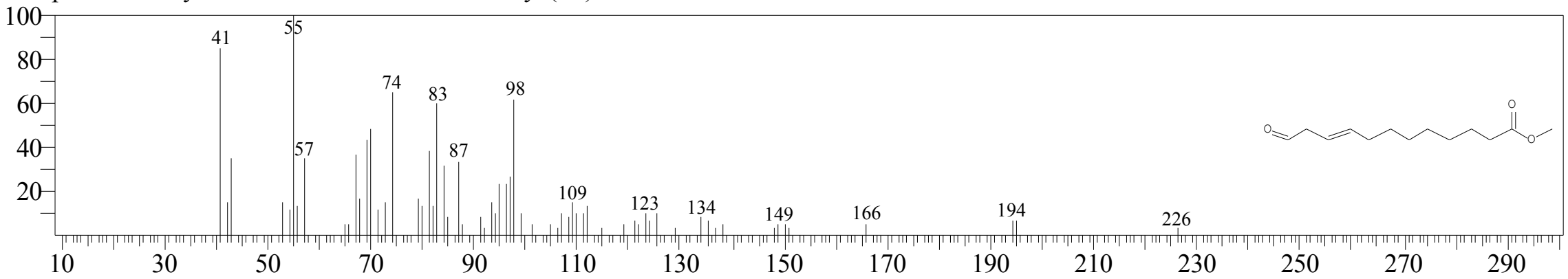


Hit#:5  Entry:21222  Library:NIST17s.lib
SI:78  Formula:C12H22O2  CAS:111-81-9  MolWeight:198  RetIndex:1371
CompName:10-Undecenoic acid, methyl ester $$ Methyl undecenate $$ Methyl undecenoate $$ Methyl 10-undecenate $$ Methyl 10-undecenoate $$ Undec

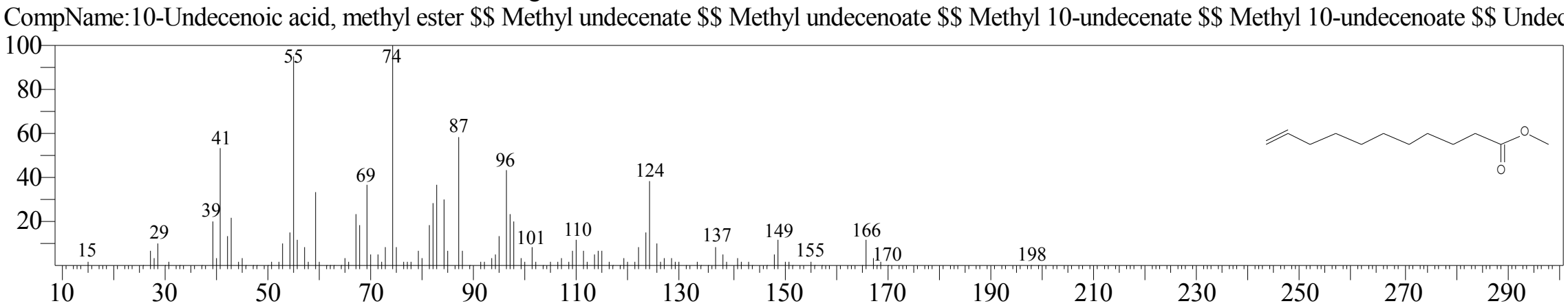

<< Target >>
Line#:5 R.Time:40.685(Scan#:6938) Retention Index:2271 MassPeaks:127
RawMode:Averaged 40.680-40.690(6937-6939) BasePeak:178.15(5668)
BG Mode:Calc. from Peak Group 1 - Event 1 Scan

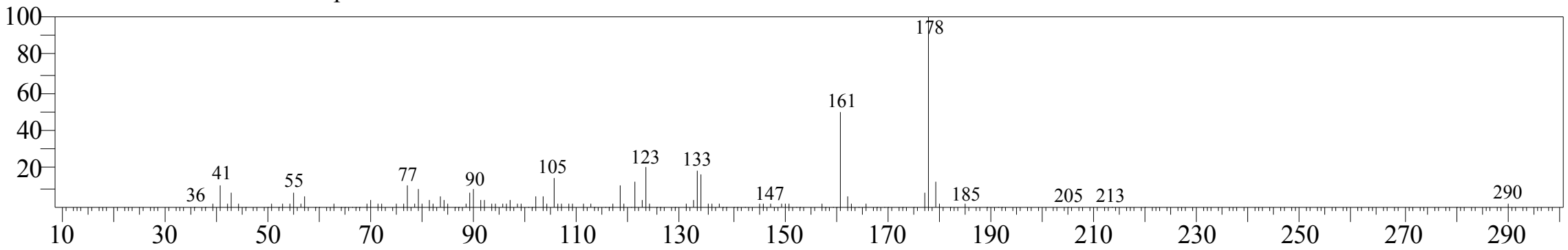


Hit#:1 Entry:32289 Library:NIST17s.lib
SI:86 Formula:C18H26O3 CAS:5466-77-3 MolWeight:290 RetIndex:2088
CompName:2-Propenoic acid, 3-(4-methoxyphenyl)-, 2-ethylhexyl ester $$ Octinoxate $$ Parsol MCX $$ Parsol MOX $$ 2-Ethylhexyl p-methoxycinnamat

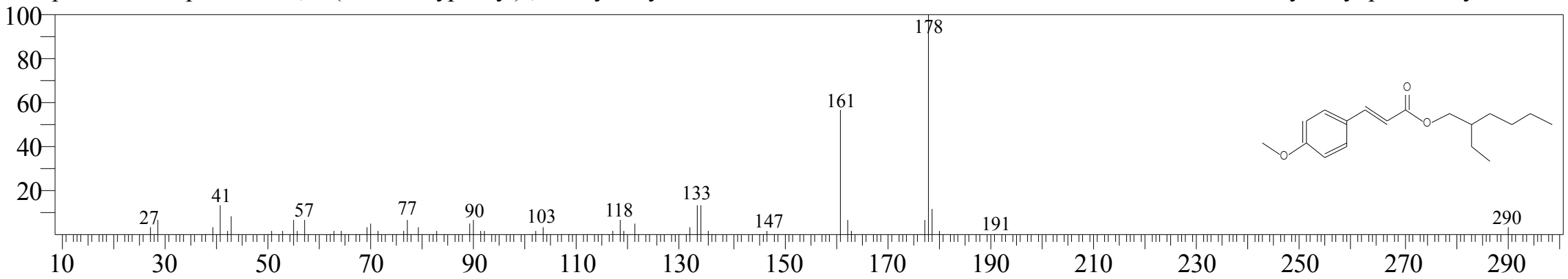


Hit#:2 Entry:133588 Library:NIST17-1.lib
SI:79 Formula:C18H26O3 CAS:83834-59-7 MolWeight:290 RetIndex:2088
CompName:2-Ethylhexyl trans-4-methoxycinnamate $$ 2-Ethylhexyl (2E)-3-(4-methoxyphenyl)-2-propenoate # $$

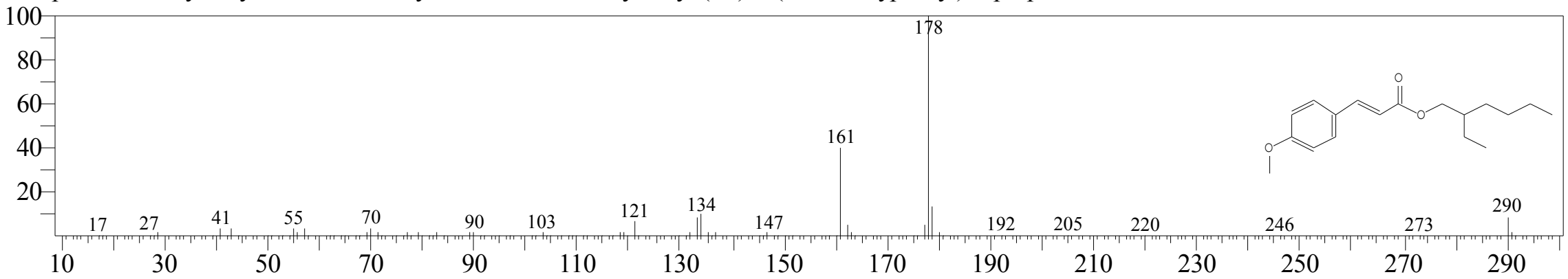


Hit#:3 Entry:28691 Library:NIST17s.lib
SI:74 Formula:C14H18O4 CAS:104-28-9 MolWeight:250 RetIndex:1830
CompName:Cinoxate $$ 2-Propenoic acid, 3-(4-methoxyphenyl)-, 2-ethoxyethyl ester $$ Cinnamic acid, p-methoxy-, 2-ethoxyethyl ester $$ p-Methoxycinna

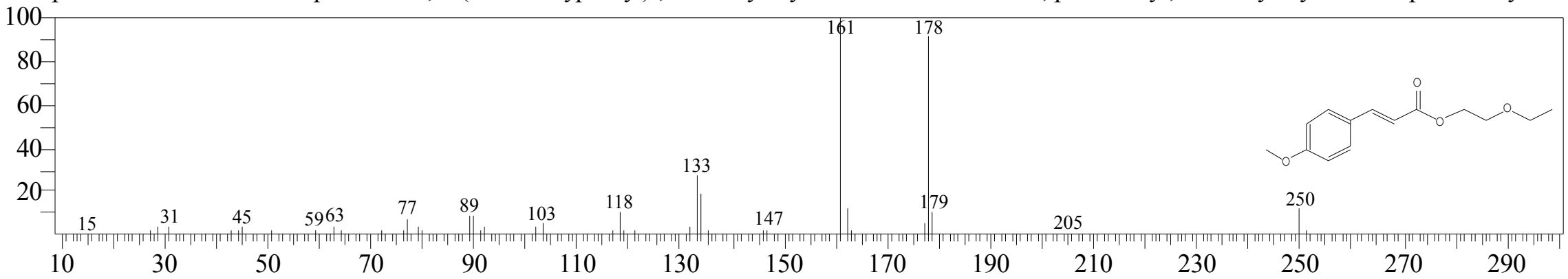


Hit#:4 Entry:17022 Library:NIST17s.lib
SI:73 Formula:C10H10O3 CAS:830-09-1 MolWeight:178 RetIndex:1546
CompName:2-Propenoic acid, 3-(4-methoxyphenyl)- $$ 4-Methoxycinnamic acid $$ p-Methoxycinnamic acid $$ Cinnamic acid, p-methoxy- $$ Cinnamic ac

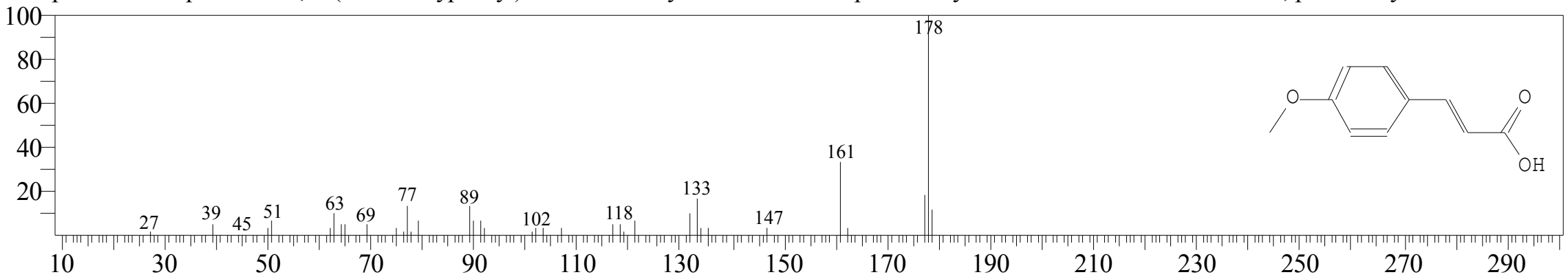


Hit#:5 Entry:33176 Library:NIST17-1.lib
SI:73 Formula:C10H10O3 CAS:943-89-5 MolWeight:178 RetIndex:1546
CompName:2-Propenoic acid, 3-(4-methoxyphenyl)-, (E)- $$ trans-2-Propenoic acid, 3-(4-methoxyphenyl)- $$ (2E)-3-(4-Methoxyphenyl)-2-propenoic acid

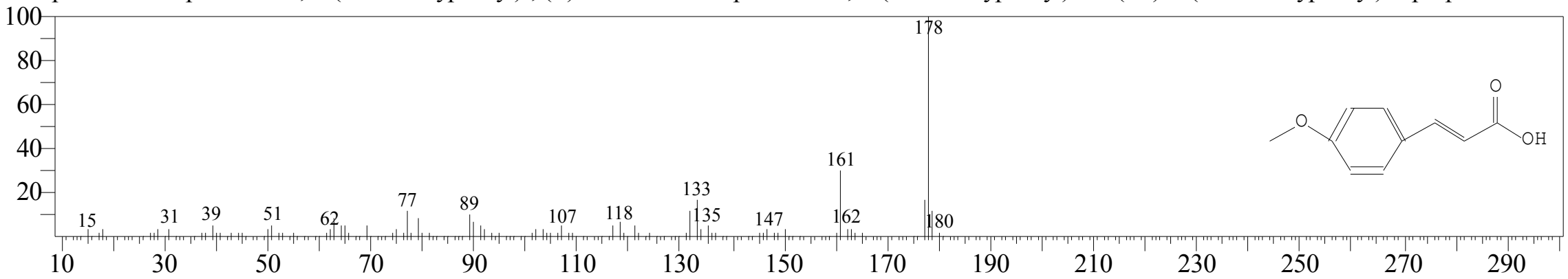

<< Target >>
Line#:6 R.Time:42.790(Scan#:7359) Retention Index:2393 MassPeaks:108
RawMode:Averaged 42.785-42.795(7358-7360) BasePeak:85.20(3519)
BG Mode:Calc. from Peak Group 1 - Event 1 Scan

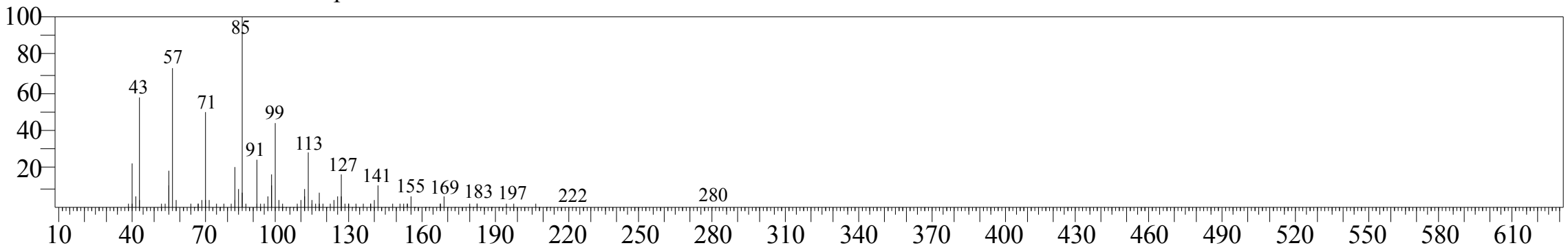


Hit#:1 Entry:32774 Library:NIST17s.lib
SI:84 Formula:C21H44 CAS:629-94-7 MolWeight:296 RetIndex:2109
CompName:Heneicosane $$ n-Heneicosane $$ Henicosane # $$

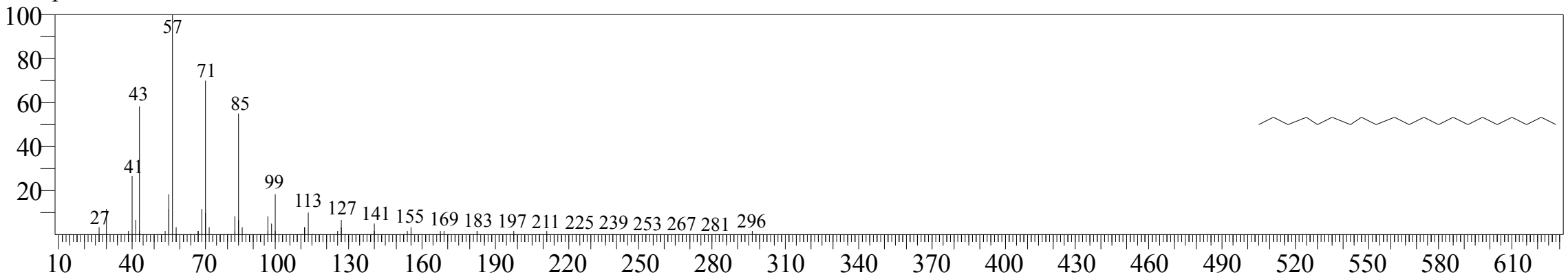


Hit#:2 Entry:125569 Library:NIST17-1.lib
SI:84 Formula:C20H42 CAS:112-95-8 MolWeight:282 RetIndex:2009
CompName:Eicosane $$ n-Eicosane $$ Icosane # $$ n-Icosane $$

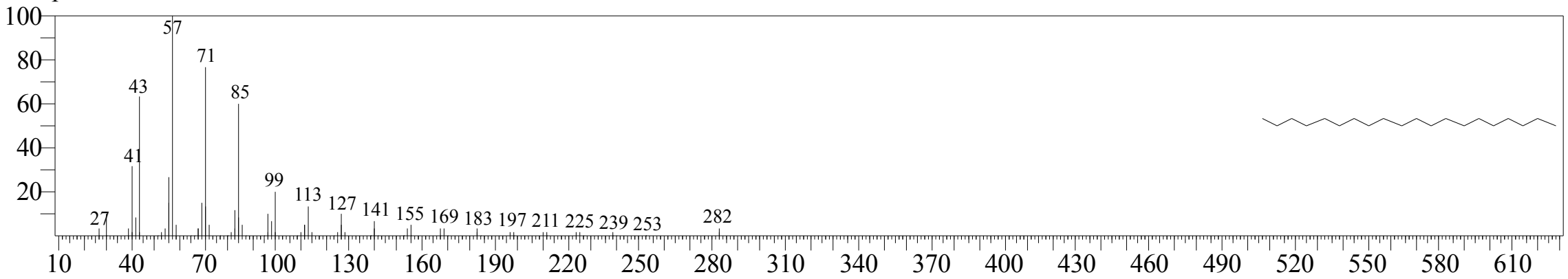


Hit#:3 Entry:7330 Library:NIST17-2.lib
SI:84 Formula:C44H90 CAS:7098-22-8 MolWeight:618 RetIndex:4395
CompName:Tetratetracontane $$ n-Tetratetracontane $$

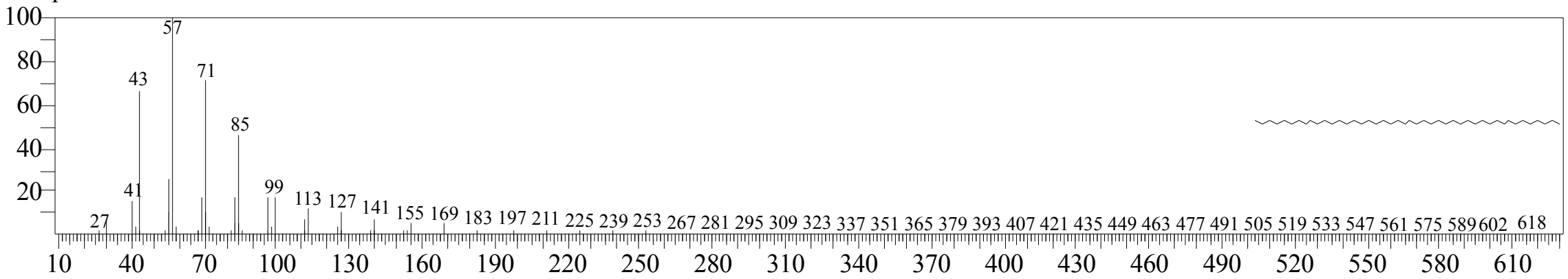


Hit#:4 Entry:38741 Library:NIST17s.lib
SI:83 Formula:C35H72 CAS:630-07-9 MolWeight:492 RetIndex:3500
CompName:Pentatriacontane $$ n-Pentatriacontane $$

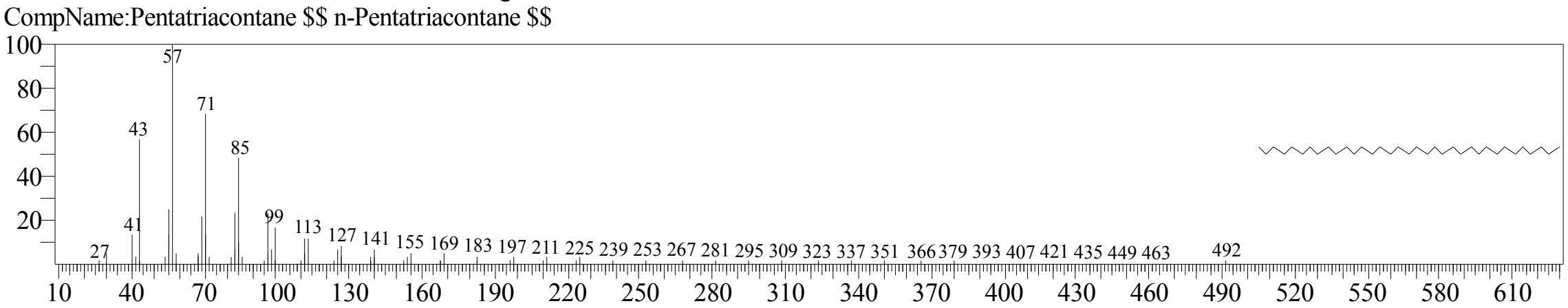


Hit#:5 Entry:27663 Library:NIST17s.lib
SI:83 Formula:C17H36 CAS:629-78-7 MolWeight:240 RetIndex:1711
CompName:Heptadecane $$ n-Heptadecane $$ Normal-heptadecane $$

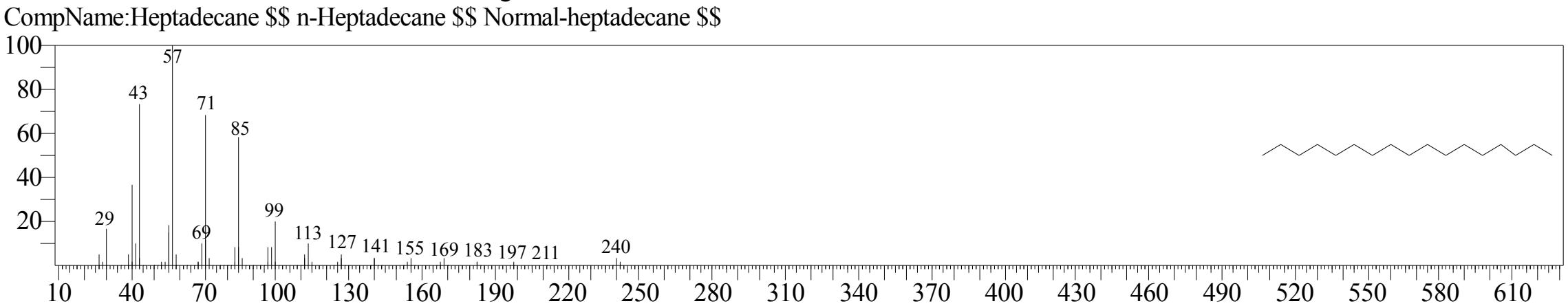

<< Target >>
Line#:7   R.Time:43.965(Scan#:7594) Retention Index:2465 MassPeaks:127
RawMode:Averaged 43.960-43.970(7593-7595) BasePeak:117.15(5582)
BG Mode:Calc. from Peak  Group 1 - Event 1  Scan

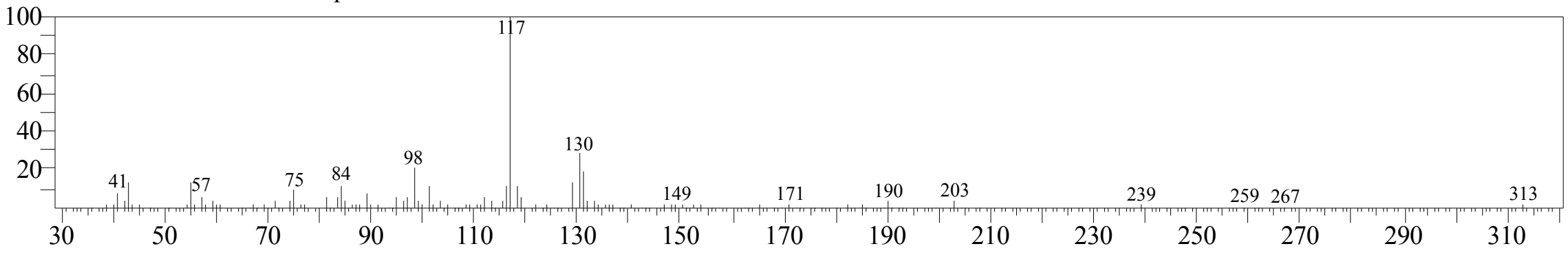


No hit compound

<< Target >>
Line#:8   R.Time:44.430(Scan#:7687) Retention Index:2493 MassPeaks:111
RawMode:Averaged 44.425-44.435(7686-7688) BasePeak:85.20(5400)
BG Mode:Calc. from Peak  Group 1 - Event 1  Scan

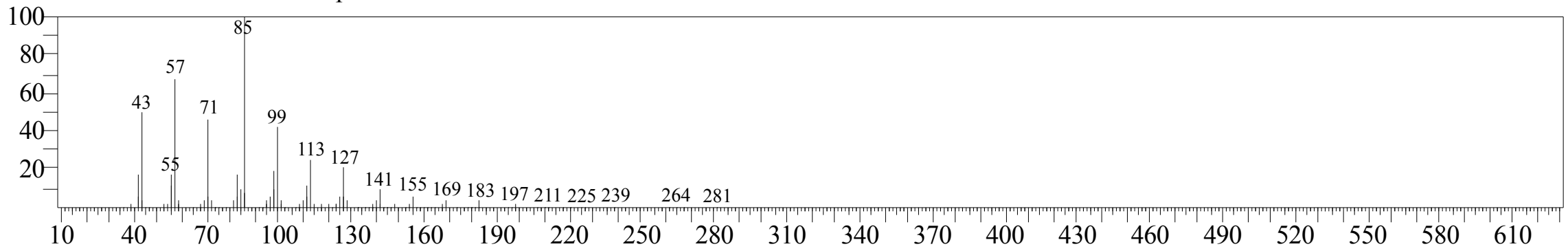


Hit#:1  Entry:7330  Library:NIST17-2.lib
SI:86  Formula:C44H90  CAS:7098-22-8  MolWeight:618  RetIndex:4395
CompName:Tetratetracontane $$ n-Tetratetracontane $$

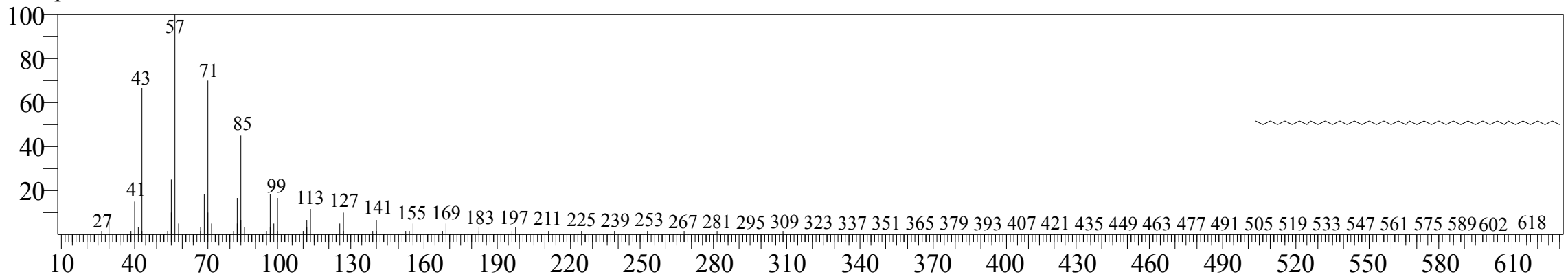


Hit#:2  Entry:38741  Library:NIST17s.lib
SI:86  Formula:C35H72  CAS:630-07-9  MolWeight:492  RetIndex:3500
CompName:Pentatriacontane $$ n-Pentatriacontane $$

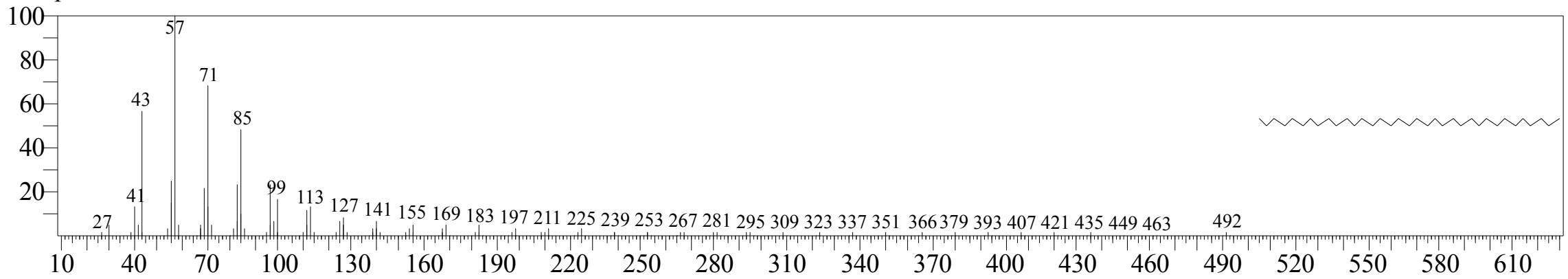


Hit#:3  Entry:38991  Library:NIST17s.lib
SI:85  Formula:C40H82  CAS:4181-95-7  MolWeight:562  RetIndex:3997
CompName:Tetracontane $$ n-Tetracontane $$

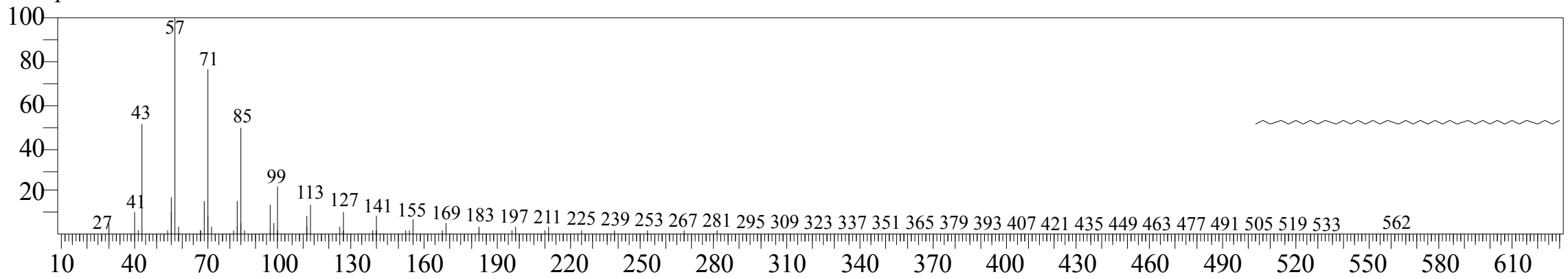


Hit#:4  Entry:125569  Library:NIST17-1.lib
SI:85  Formula:C20H42  CAS:112-95-8  MolWeight:282  RetIndex:2009
CompName:Eicosane $$ n-Eicosane $$ Icosane # $$ n-Icosane $$

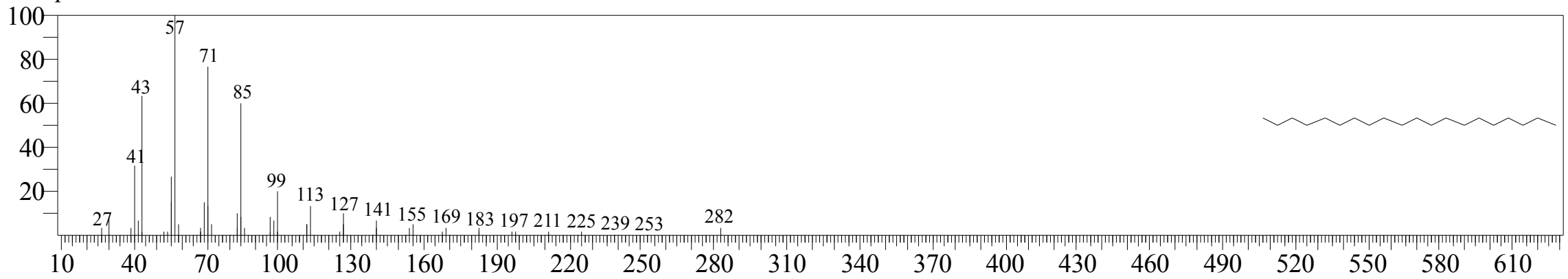


Hit#:5  Entry:32774  Library:NIST17s.lib
SI:85  Formula:C21H44  CAS:629-94-7  MolWeight:296  RetIndex:2109
CompName:Heneicosane $$ n-Heneicosane $$ Henicosane # $$

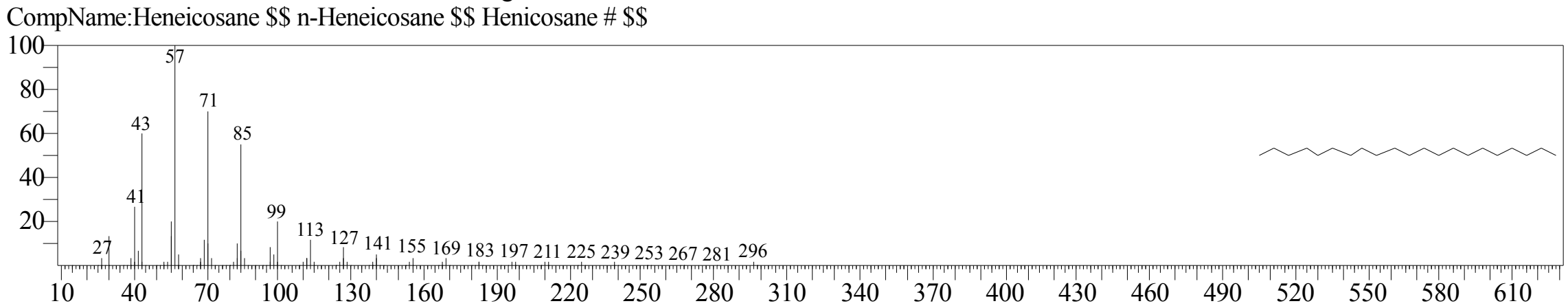

<< Target >>
Line#:9   R.Time:44.525(Scan#:7706) Retention Index:2499 MassPeaks:106
RawMode:Averaged 44.520-44.530(7705-7707) BasePeak:149.10(8288)
BG Mode:Calc. from Peak  Group 1 - Event 1  Scan

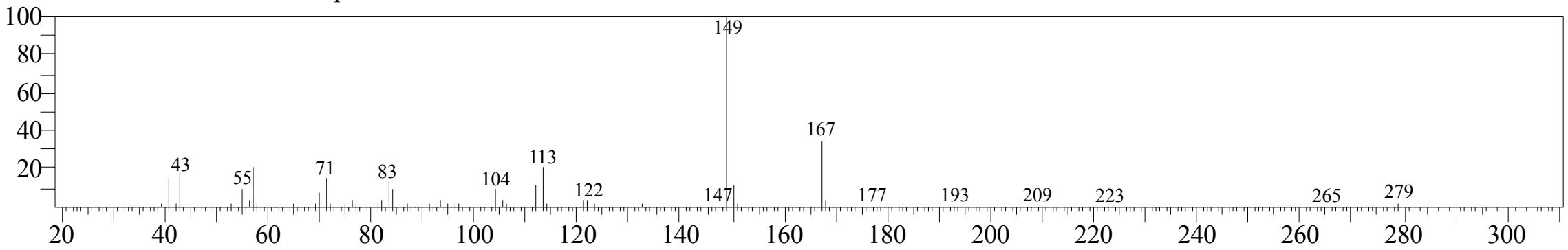


Hit#:1  Entry:37380  Library:NIST17s.lib
SI:92  Formula:C24H38O4  CAS:117-81-7  MolWeight:390  RetIndex:2704
CompName:Bis(2-ethylhexyl) phthalate $$ Phthalic acid, bis(2-ethylhexyl) ester $$ Bis(2-ethylhexyl) 1,2-benzenedicarboxylate $$ Bisoflex 81 $$ Compoun

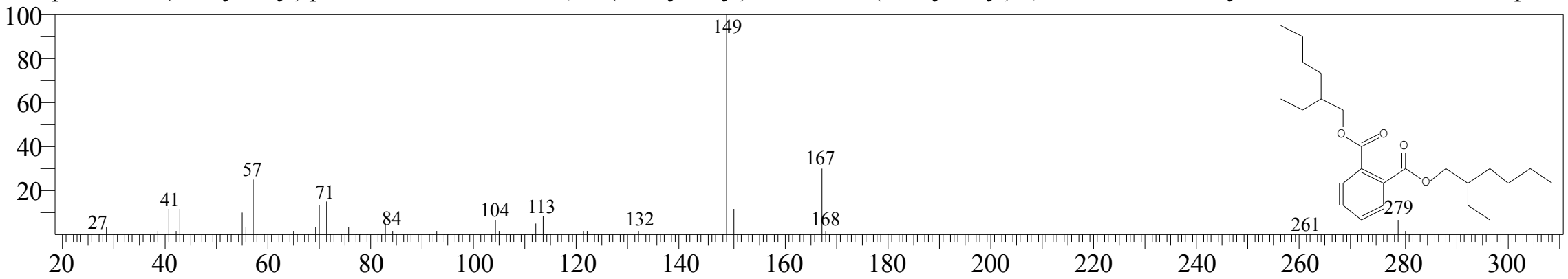


Hit#:2  Entry:222221  Library:NIST17-1.lib
SI:89  Formula:C24H38O4  CAS:0-00-0  MolWeight:390  RetIndex:2704
CompName:Phthalic acid, di(2-propylpentyl) ester

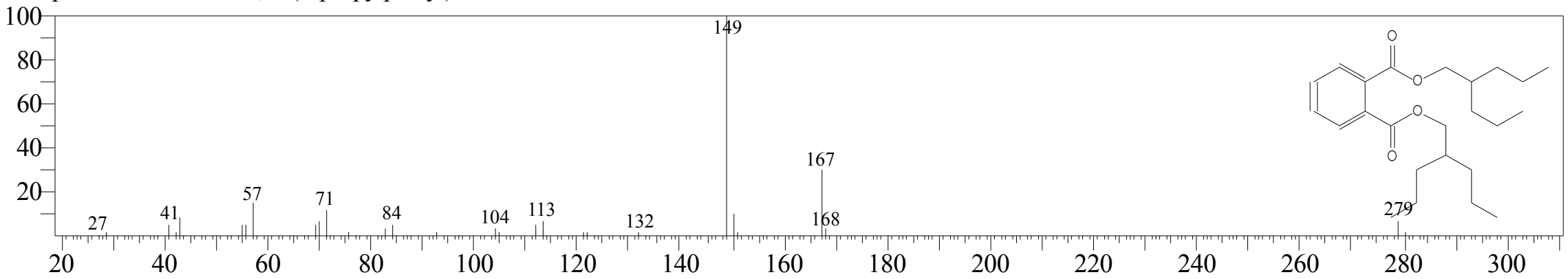


Hit#:3  Entry:222222  Library:NIST17-1.lib
SI:88  Formula:C24H38O4  CAS:0-00-0  MolWeight:390  RetIndex:2575
CompName:Phthalic acid, di(6-methylhept-2-yl) ester

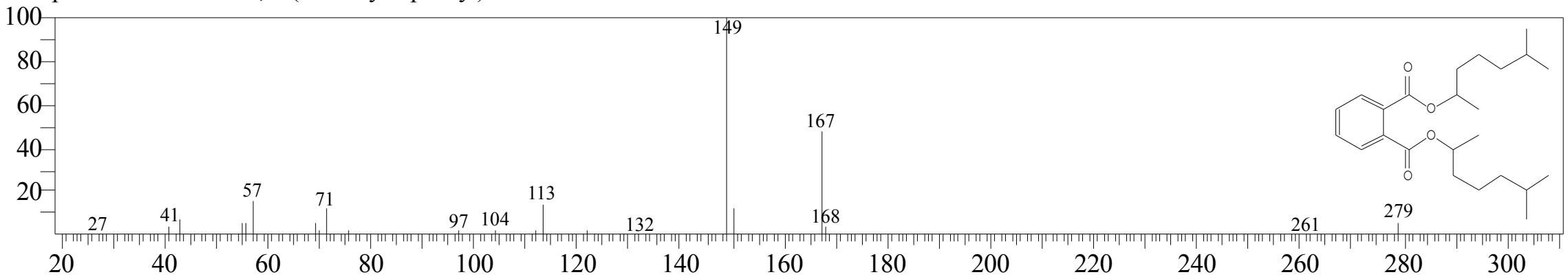


Hit#:4  Entry:31276  Library:NIST17s.lib
SI:87  Formula:C16H22O4  CAS:4376-20-9  MolWeight:278  RetIndex:2162
CompName:Mono(2-ethylhexyl) phthalate $$ 1,2-Benzenedicarboxylic acid, mono(2-ethylhexyl) ester $$ Phthalic acid, mono-(2-ethylhexyl) ester $$ Mehp

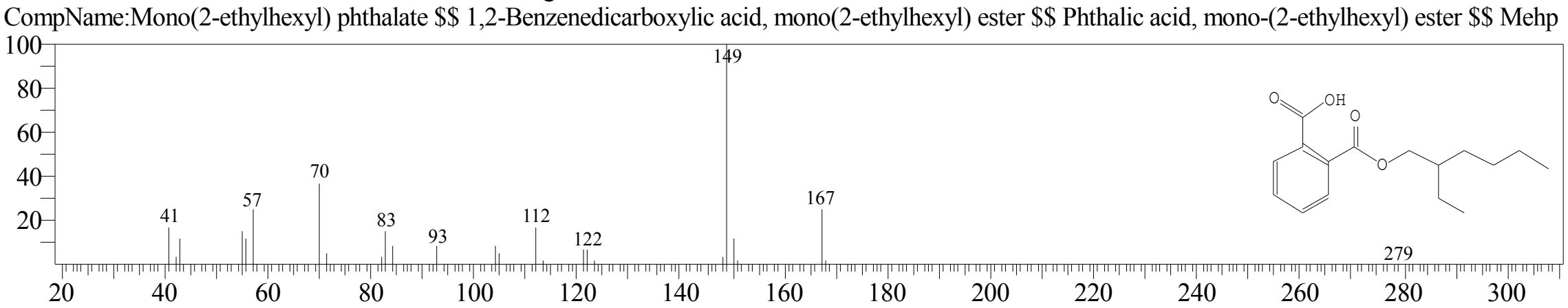


Hit#:5  Entry:236172  Library:NIST17-1.lib
SI:85  Formula:C26H42O4  CAS:0-00-0  MolWeight:418  RetIndex:2838
CompName:Phthalic acid, 6-ethyloct-3-yl 2-ethylhexyl ester

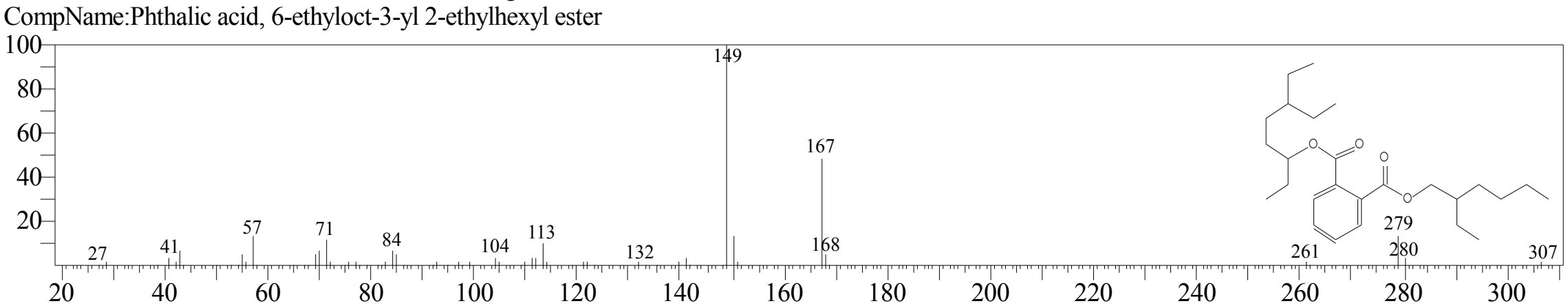

<< Target >>
Line#:10 R.Time:46.010(Scan#:8003) Retention Index:2593 MassPeaks:123
RawMode:Averaged 46.005-46.015(8002-8004) BasePeak:85.20(6697)
BG Mode:Calc. from Peak Group 1 - Event 1 Scan

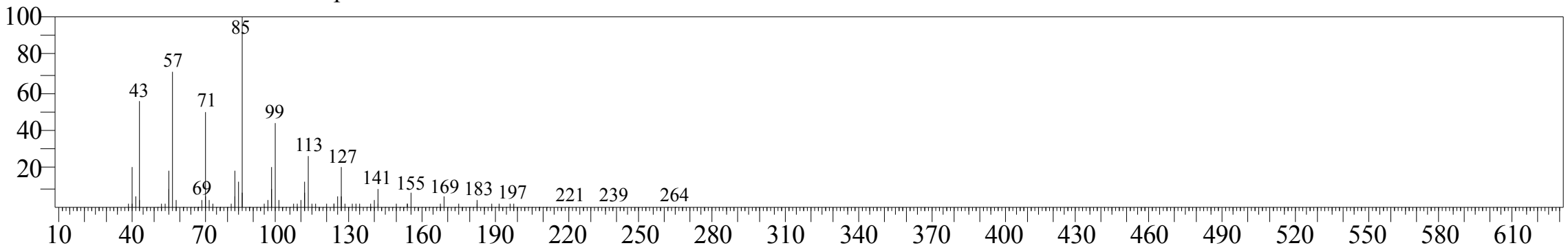


Hit#:1 Entry:7330 Library:NIST17-2.lib
SI:87 Formula:C44H90 CAS:7098-22-8 MolWeight:618 RetIndex:4395
CompName:Tetratetracontane $$ n-Tetratetracontane $$

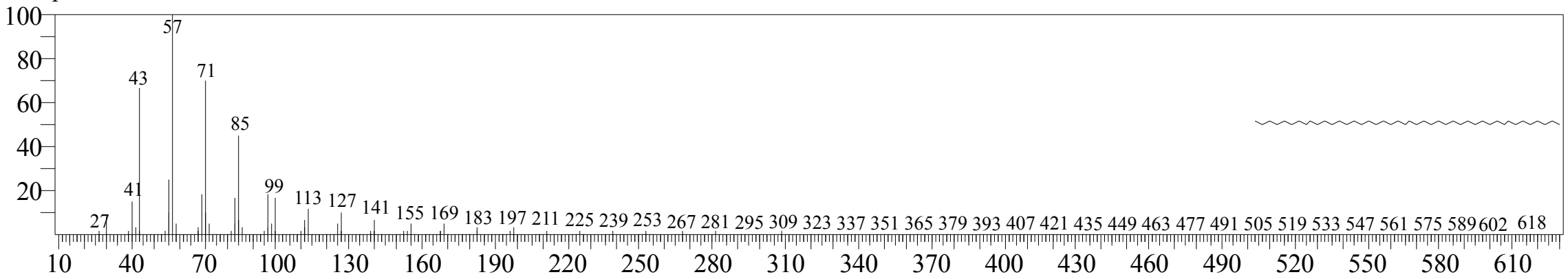


Hit#:2 Entry:38741 Library:NIST17s.lib
SI:87 Formula:C35H72 CAS:630-07-9 MolWeight:492 RetIndex:3500
CompName:Pentatriacontane $$ n-Pentatriacontane $$

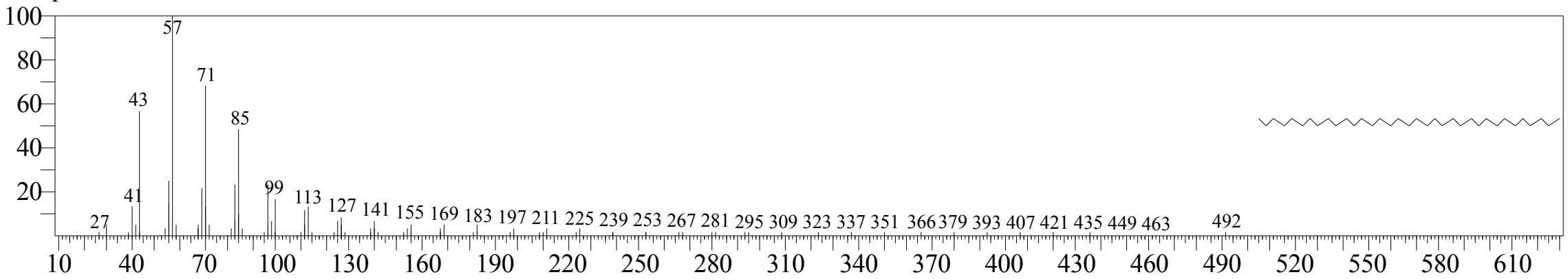


Hit#:3 Entry:760 Library:NIST17-2.lib
SI:86 Formula:C36H74 CAS:630-06-8 MolWeight:506 RetIndex:3600
CompName:Hexatriacontane $$ n-Hexatriacontane $$

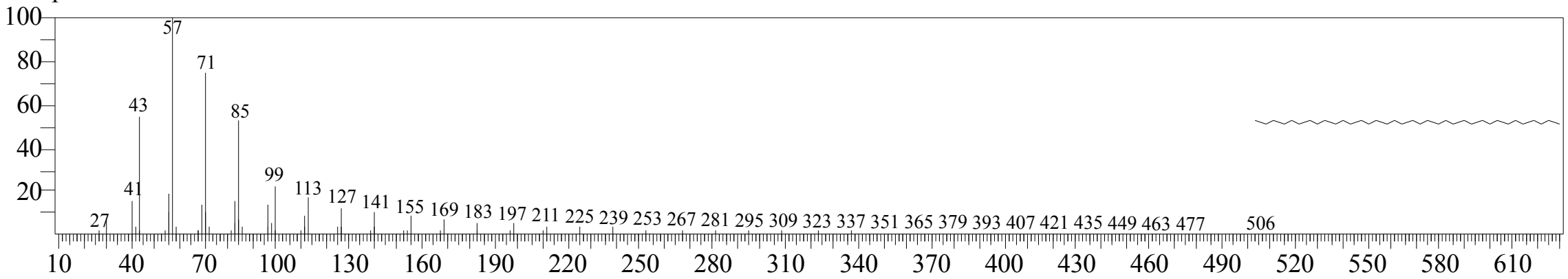


Hit#:4 Entry:125569 Library:NIST17-1.lib
SI:86 Formula:C20H42 CAS:112-95-8 MolWeight:282 RetIndex:2009
CompName:Eicosane $$ n-Eicosane $$ Icosane # $$ n-Icosane $$

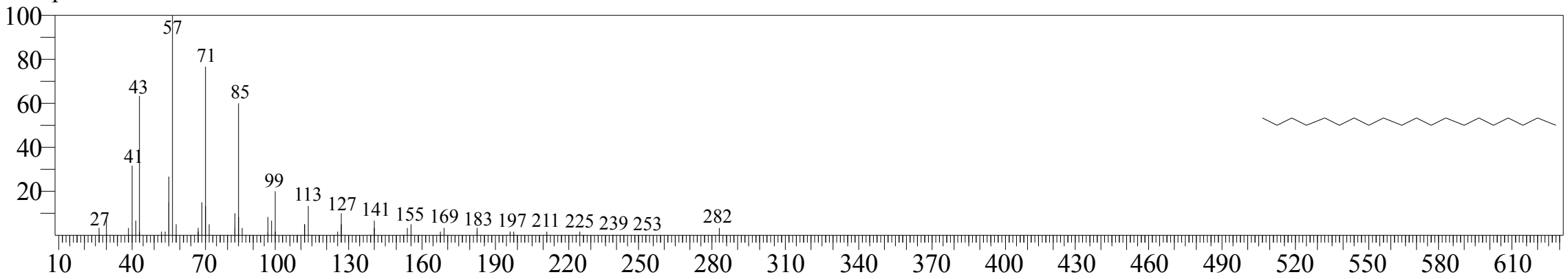


Hit#:5 Entry:32774 Library:NIST17s.lib
SI:86 Formula:C21H44 CAS:629-94-7 MolWeight:296 RetIndex:2109
CompName:Heneicosane $$ n-Heneicosane $$ Henicosane # $$

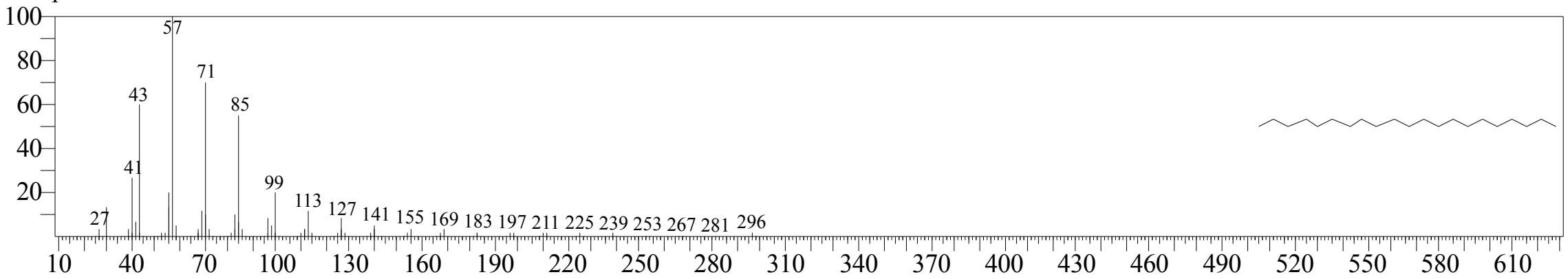

<< Target >>
Line#:11 R.Time:47.525(Scan#:8306) Retention Index:2693 MassPeaks:129
RawMode:Averaged 47.520-47.530(8305-8307) BasePeak:85.20(6475)
BG Mode:Calc. from Peak Group 1 - Event 1 Scan

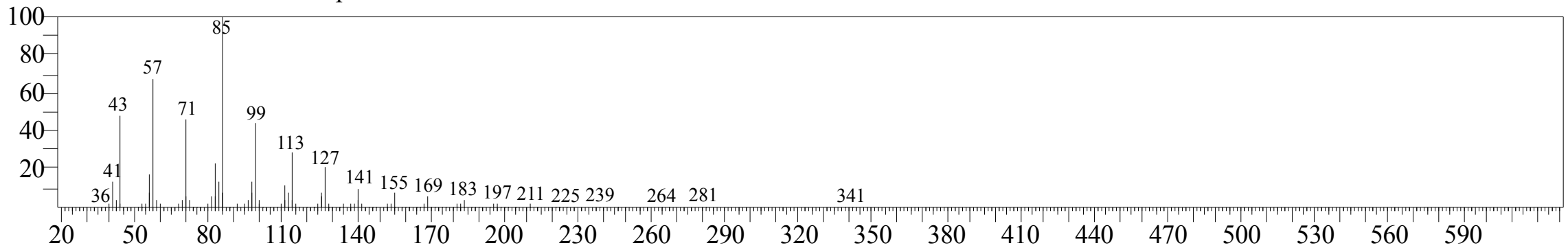


Hit#:1 Entry:38991 Library:NIST17s.lib
SI:86 Formula:C40H82 CAS:4181-95-7 MolWeight:562 RetIndex:3997
CompName:Tetracontane $$ n-Tetracontane $$

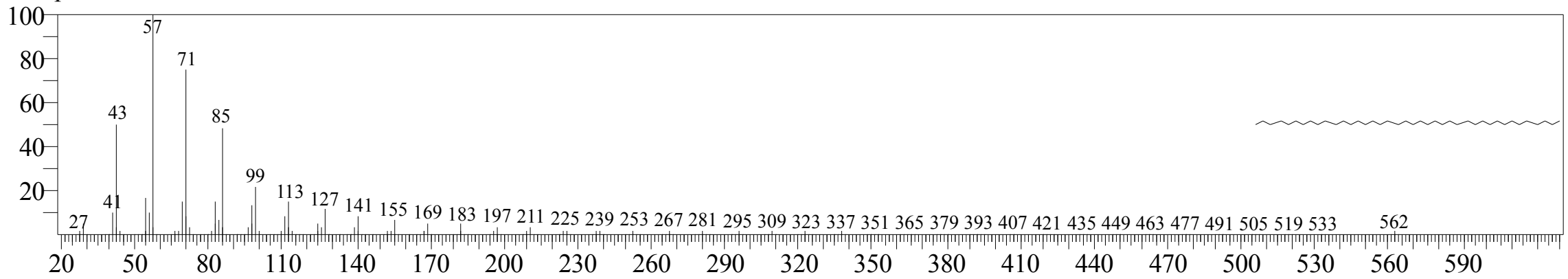


Hit#:2 Entry:38741 Library:NIST17s.lib
SI:86 Formula:C35H72 CAS:630-07-9 MolWeight:492 RetIndex:3500
CompName:Pentatriacontane $$ n-Pentatriacontane $$

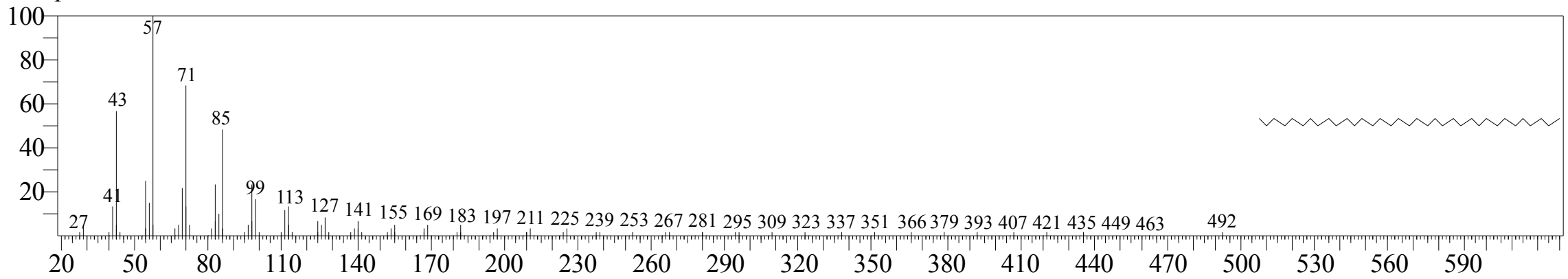


Hit#:3 Entry:7330 Library:NIST17-2.lib
SI:86 Formula:C44H90 CAS:7098-22-8 MolWeight:618 RetIndex:4395
CompName:Tetratetracontane $$ n-Tetratetracontane $$

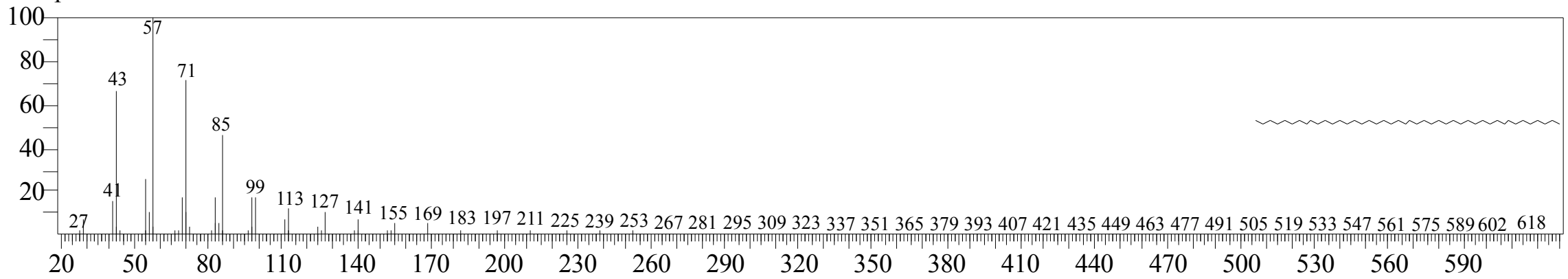


Hit#:4 Entry:760 Library:NIST17-2.lib
SI:86 Formula:C36H74 CAS:630-06-8 MolWeight:506 RetIndex:3600
CompName:Hexatriacontane $$ n-Hexatriacontane $$

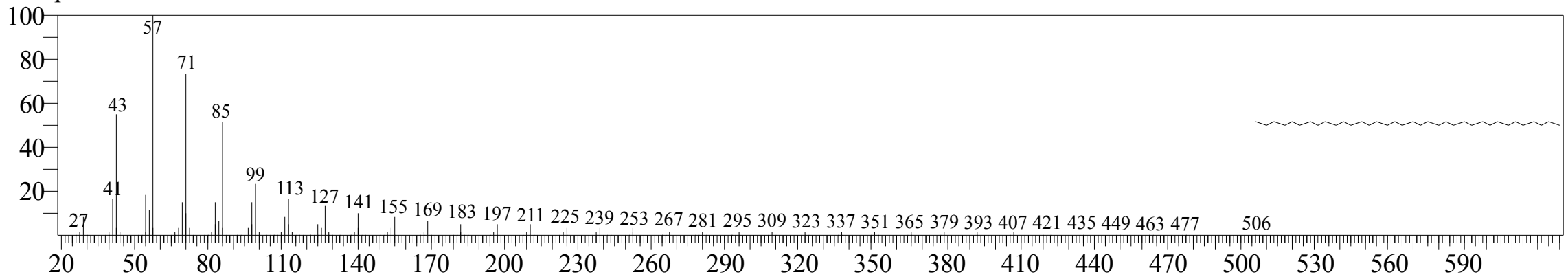


Hit#:5 Entry:253745 Library:NIST17-1.lib
SI:85 Formula:C34H70 CAS:14167-59-0 MolWeight:478 RetIndex:3401
CompName:Tetratriacontane $$ n-Tetratriacontane $$

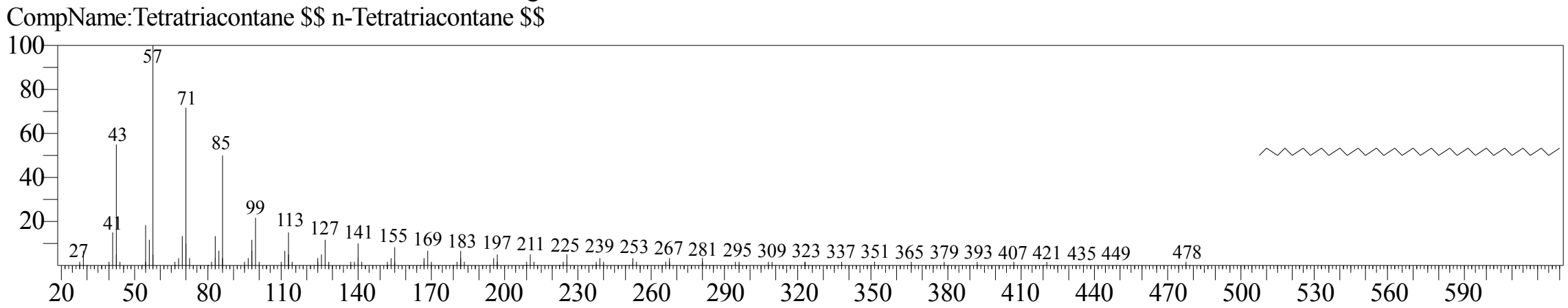

<< Target >>
Line#:12   R.Time:48.990(Scan#:8599) Retention Index:2793 MassPeaks:134
RawMode:Averaged 48.985-48.995(8598-8600) BasePeak:85.20(6069)
BG Mode:Calc. from Peak  Group 1 - Event 1  Scan

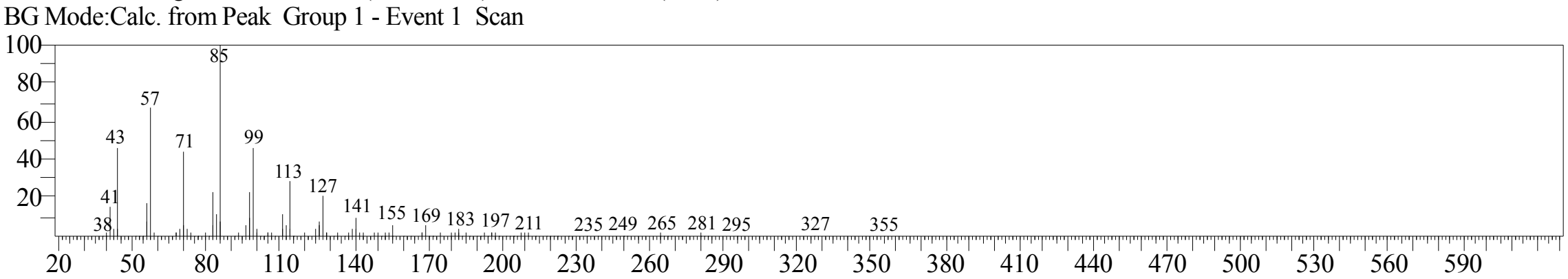


Hit#:1  Entry:38741  Library:NIST17s.lib
SI:85  Formula:C35H72  CAS:630-07-9  MolWeight:492  RetIndex:3500
CompName:Pentatriacontane $$ n-Pentatriacontane $$

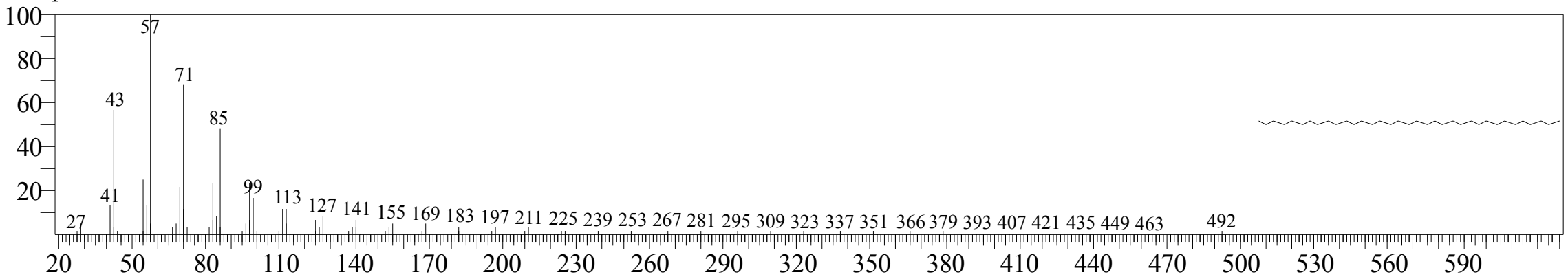


Hit#:2  Entry:38991  Library:NIST17s.lib
SI:85  Formula:C40H82  CAS:4181-95-7  MolWeight:562  RetIndex:3997
CompName:Tetracontane $$ n-Tetracontane $$

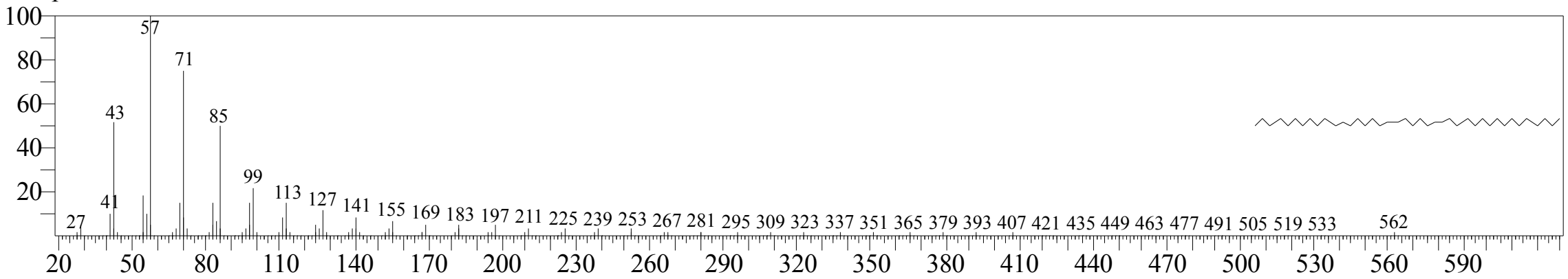


Hit#:3  Entry:7330  Library:NIST17-2.lib
SI:84  Formula:C44H90  CAS:7098-22-8  MolWeight:618  RetIndex:4395
CompName:Tetratetracontane $$ n-Tetratetracontane $$

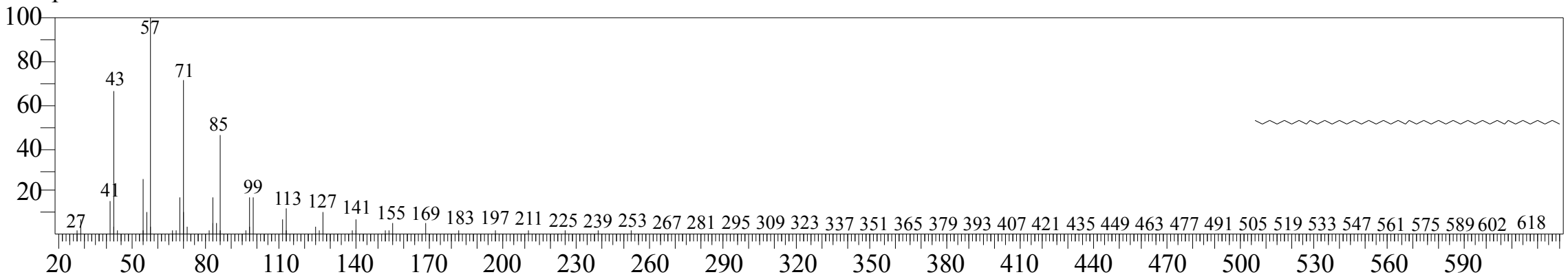


Hit#:4  Entry:760  Library:NIST17-2.lib
SI:84  Formula:C36H74  CAS:630-06-8  MolWeight:506  RetIndex:3600
CompName:Hexatriacontane $$ n-Hexatriacontane $$

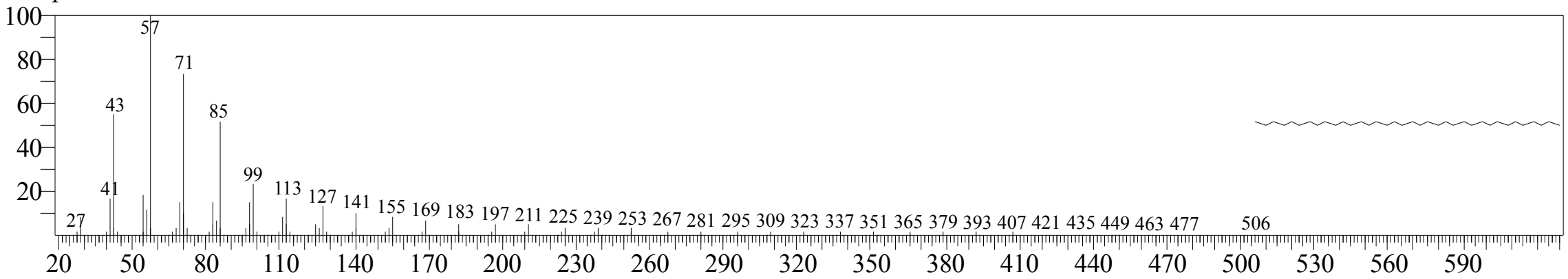


Hit#:5  Entry:125569  Library:NIST17-1.lib
SI:84  Formula:C20H42  CAS:112-95-8  MolWeight:282  RetIndex:2009
CompName:Eicosane $$ n-Eicosane $$ Icosane # $$ n-Icosane $$

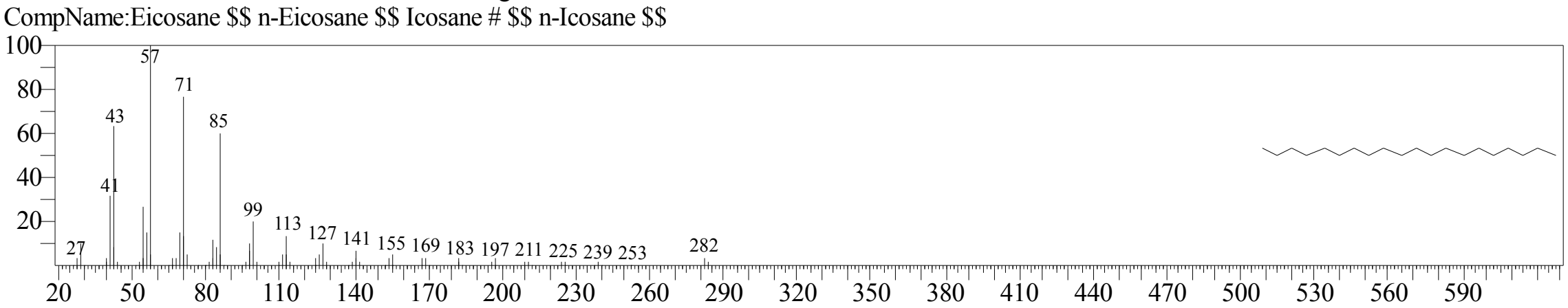

<< Target >>
Line#:13 R.Time:49.135(Scan#:8628) Retention Index:2803 MassPeaks:157
RawMode:Averaged 49.130-49.140(8627-8629) BasePeak:81.15(13014)
BG Mode:Calc. from Peak Group 1 - Event 1 Scan

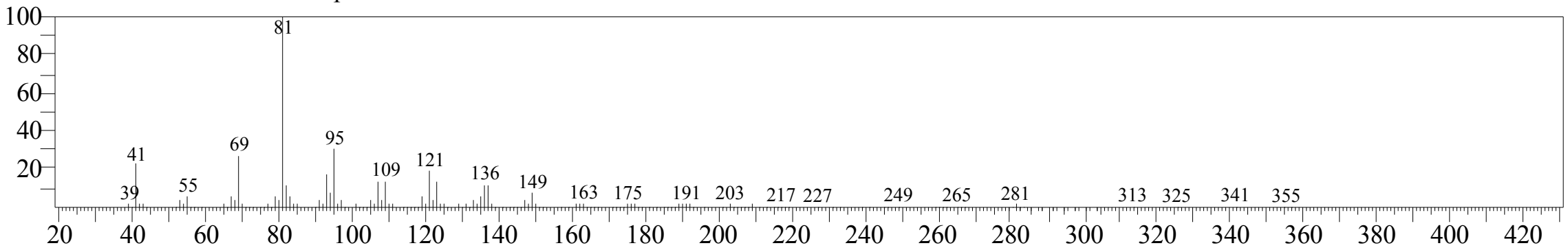


Hit#:1 Entry:232590 Library:NIST17-1.lib
SI:84 Formula:C30H50 CAS:111-02-4 MolWeight:410 RetIndex:2914
CompName:Squalene $$ 2,6,10,14,18,22-Tetracosahexaene, 2,6,10,15,19,23-hexamethyl-, (all-E)- $$ all-trans-Squalene $$ trans-Squalene $$ Spinacen $$ S

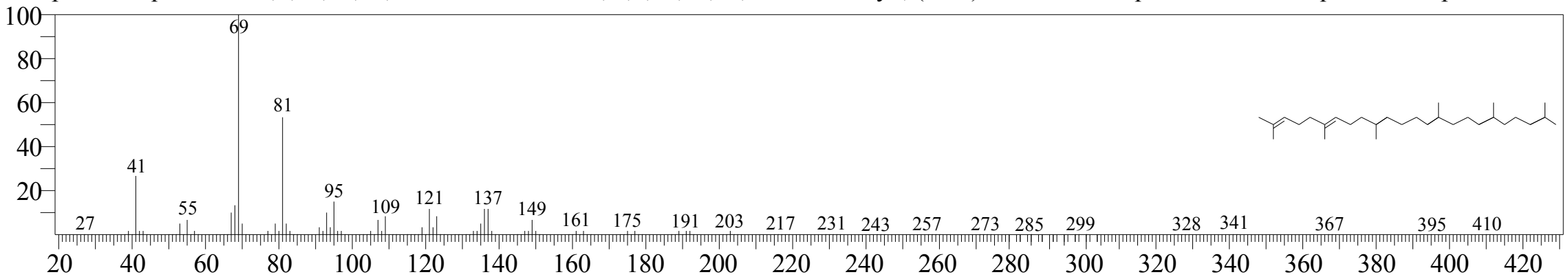


Hit#:2 Entry:90471 Library:NIST17-1.lib
SI:80 Formula:C17H26O CAS:24708-33-6 MolWeight:246 RetIndex:1833
CompName:Avocadynofuran $$ Furan, 2-(12-tridecyn-1-yl)- $$ Furan, 2-(12-tridecynyl)- $$ 2-(Tridec-12-yn-1-yl)furan $$

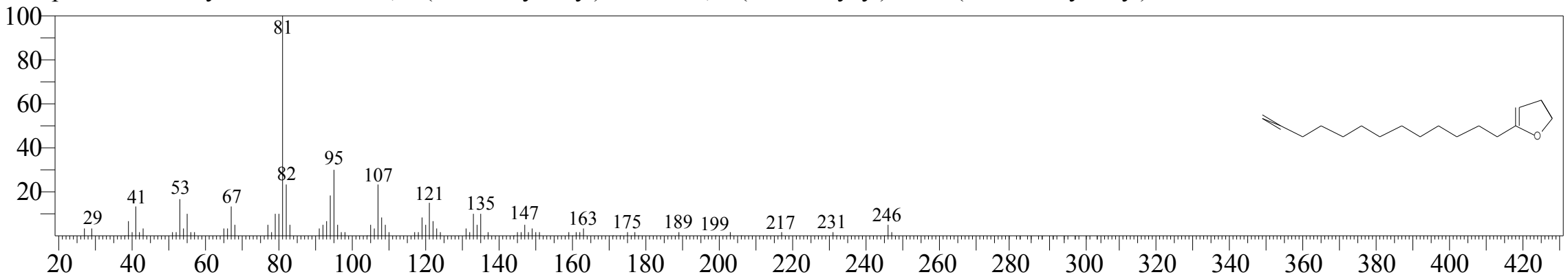


Hit#:3 Entry:68703 Library:NIST17-1.lib
SI:80 Formula:C15H26O CAS:198991-79-6 MolWeight:222 RetIndex:1660
CompName:(2E,4S,7E)-4-Isopropyl-1,7-dimethylcyclodeca-2,7-dienol $$ Germacrene D-4-ol $$ 2,7-Cyclodecadien-1-ol, 1,7-dimethyl-4-(1-methylethyl)-, (

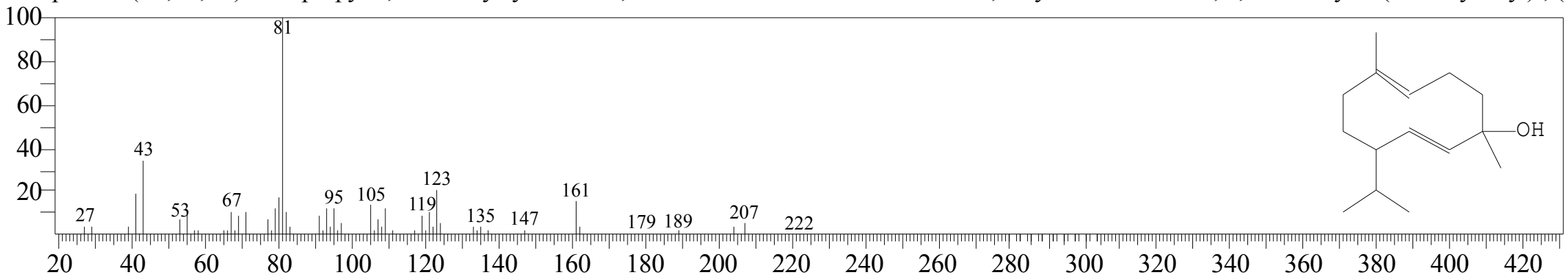


Hit#:4 Entry:38107 Library:NIST17s.lib
SI:80 Formula:C30H50O CAS:7200-26-2 MolWeight:426 RetIndex:2955
CompName:Oxirane, 2,2-dimethyl-3-(3,7,12,16,20-pentamethyl-3,7,11,15,19-heneicosapentaenyl)-, (all-E)- $$ 2,2-Dimethyl-3-[(3E,7E,11E,15E)-3,7,12,16,

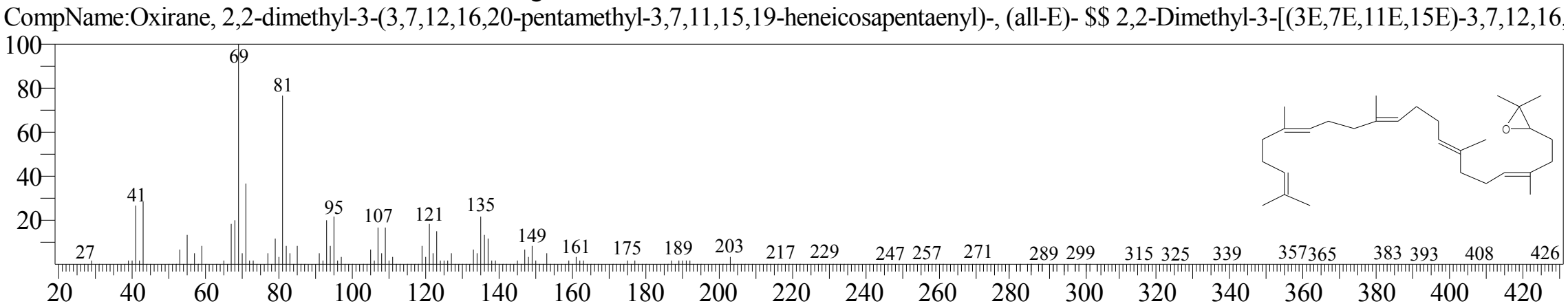


Hit#:5 Entry:233601 Library:NIST17-1.lib
SI:80 Formula:C29H48O CAS:0-00-0 MolWeight:412 RetIndex:2878
CompName:2,2-Dimethyl-3-(3,7,16,20-tetramethyl-heneicosa-3,7,11,15,19-pentaenyl)-oxirane $$ 2,2-Dimethyl-3-[(3E,7E,11E,15E)-3,7,16,20-tetramethyl-

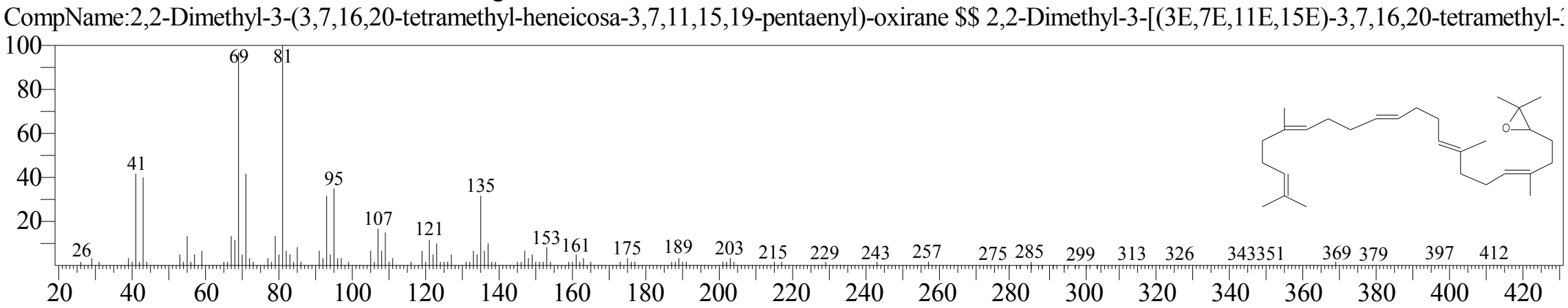

<< Target >>
Line#:14   R.Time:50.410(Scan#:8883) Retention Index:2893 MassPeaks:140
RawMode:Averaged 50.405-50.415(8882-8884) BasePeak:85.20(3734)
BG Mode:Calc. from Peak  Group 1 - Event 1  Scan

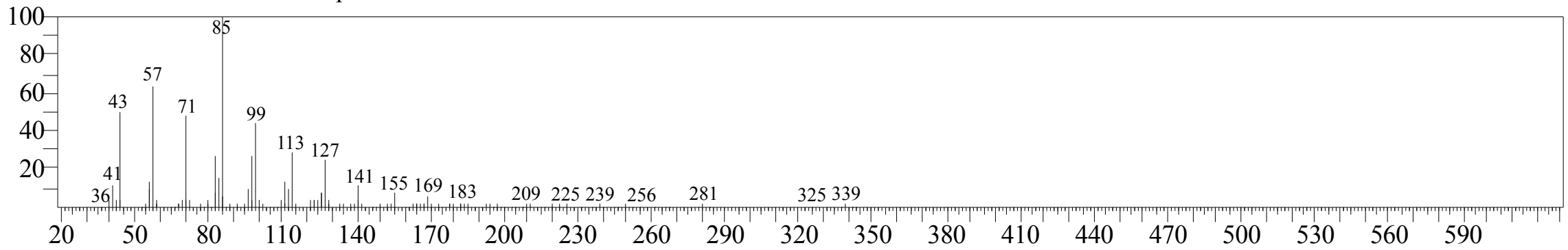


Hit#:1  Entry:38741  Library:NIST17s.lib
SI:84  Formula:C35H72  CAS:630-07-9  MolWeight:492  RetIndex:3500
CompName:Pentatriacontane $$ n-Pentatriacontane $$

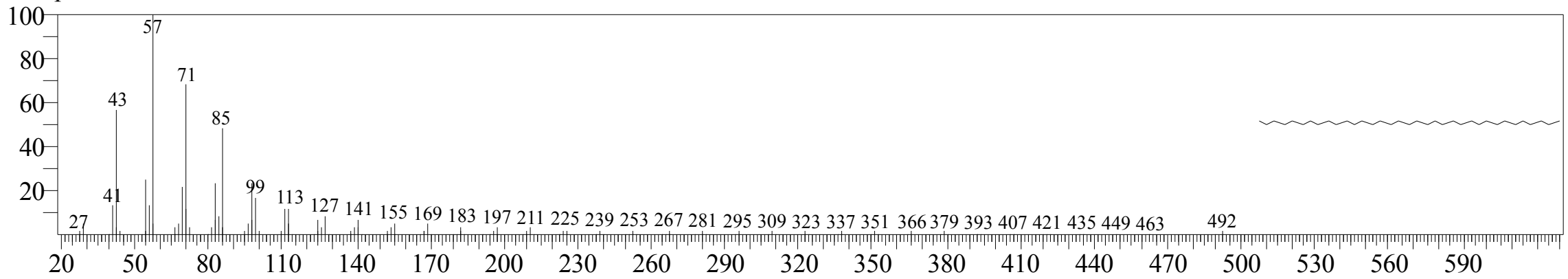


Hit#:2  Entry:7330  Library:NIST17-2.lib
SI:83  Formula:C44H90  CAS:7098-22-8  MolWeight:618  RetIndex:4395
CompName:Tetratetracontane $$ n-Tetratetracontane $$

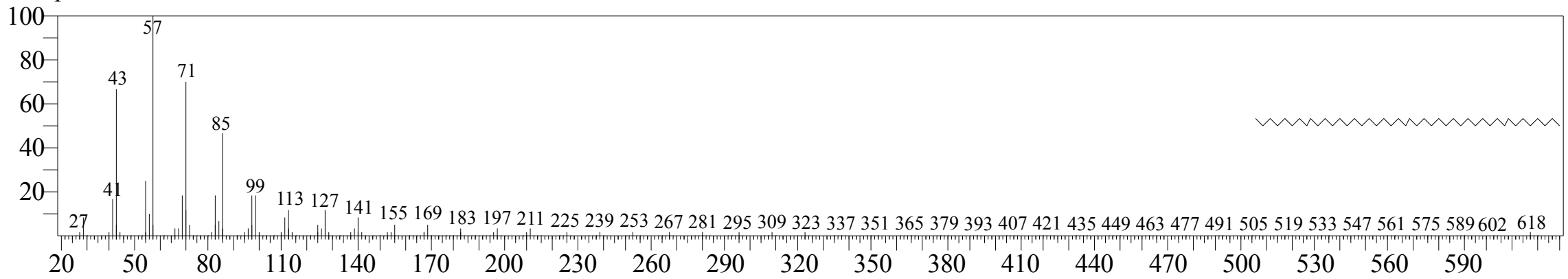


Hit#:3  Entry:38991  Library:NIST17s.lib
SI:83  Formula:C40H82  CAS:4181-95-7  MolWeight:562  RetIndex:3997
CompName:Tetracontane $$ n-Tetracontane $$

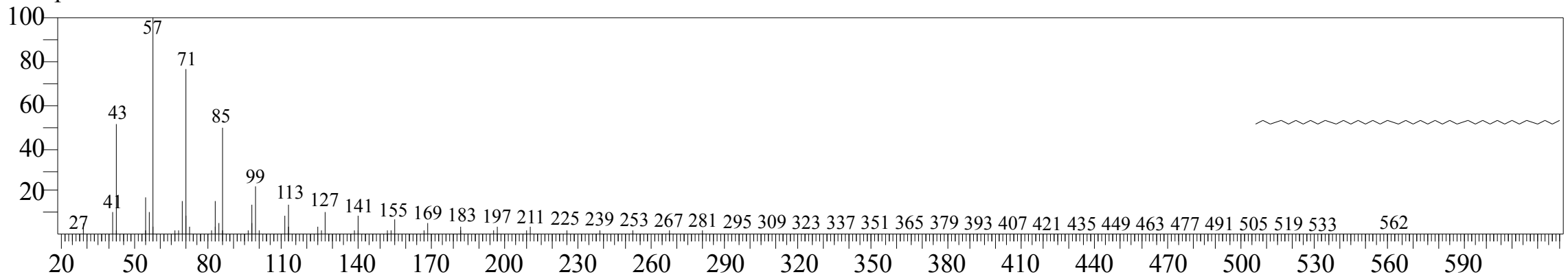


Hit#:4  Entry:760  Library:NIST17-2.lib
SI:83  Formula:C36H74  CAS:630-06-8  MolWeight:506  RetIndex:3600
CompName:Hexatriacontane $$ n-Hexatriacontane $$

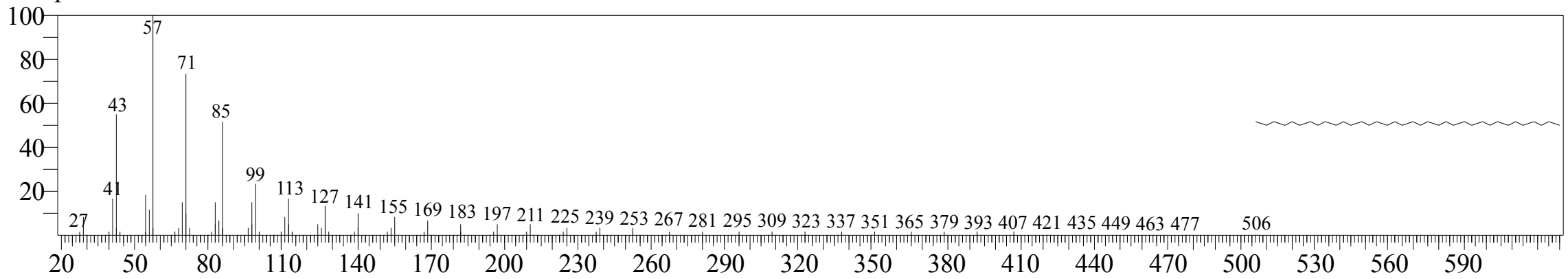


Hit#:5  Entry:183880  Library:NIST17-1.lib
SI:83  Formula:C21H40O3  CAS:0-00-0  MolWeight:340  RetIndex:2342
CompName:Carbonic acid, octadecyl vinyl ester

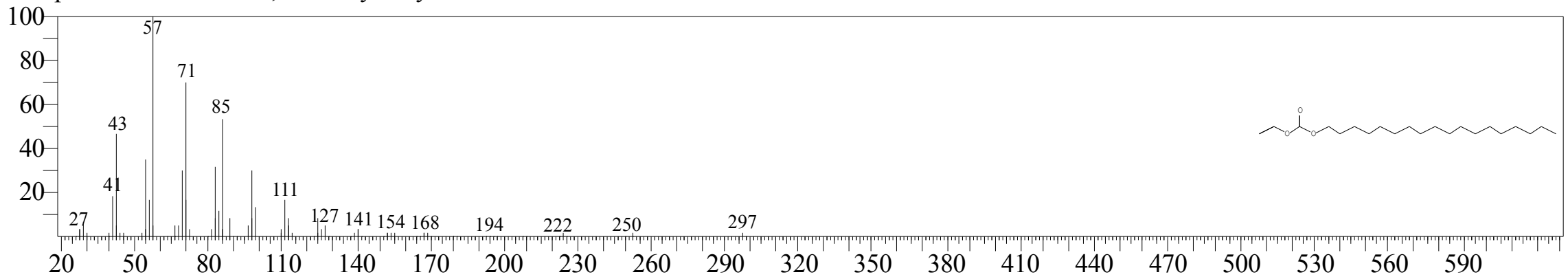

<< Target >>
Line#:15 R.Time:52.860(Scan#:9373) Retention Index:3072! MassPeaks:219
RawMode:Averaged 52.855-52.865(9372-9374) BasePeak:81.15(3225)
BG Mode:Calc. from Peak Group 1 - Event 1 Scan

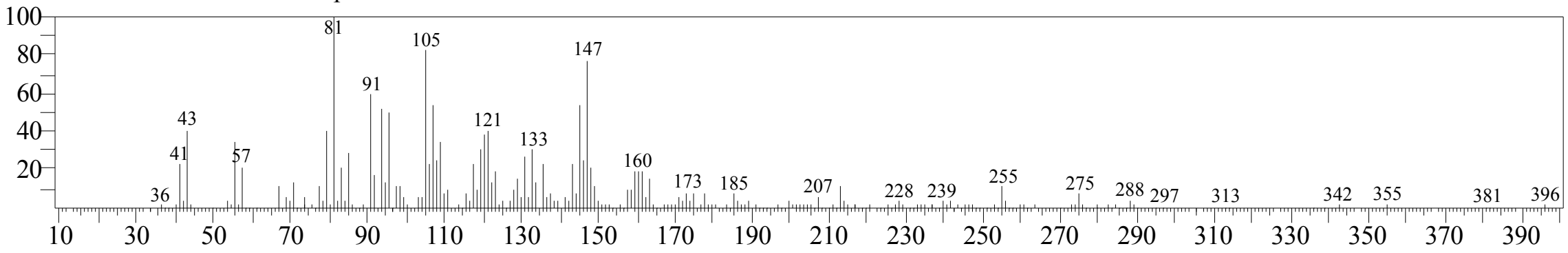


Hit#:1 Entry:230552 Library:NIST17-1.lib
SI:79 Formula:C23H34O6 CAS:560-54-3 MolWeight:406 RetIndex:3238
CompName:Card-20(22)-enolide, 3,5,14,19-tetrahydroxy-, (3.beta.,5.beta.)- $$ Strophanthidol $$ 5.beta.-Card-20(22)-enolide, 3.beta.,5,14,19-tetrahydroxy

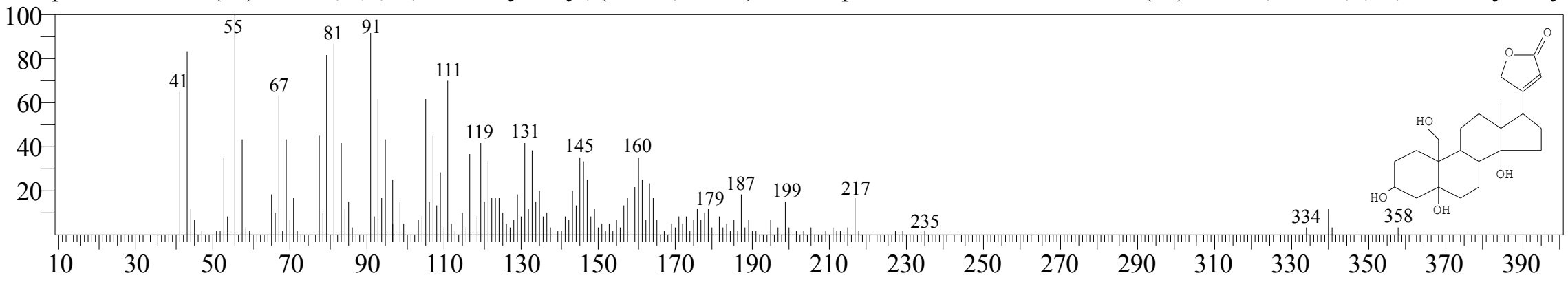


Hit#:2 Entry:131749 Library:NIST17-1.lib
SI:76 Formula:C20H32O CAS:3275-64-7 MolWeight:288 RetIndex:1963
CompName:5-.alpha.-Androst-2-en-17-.beta.-ol, 17-methyl- $$ Androst-2-en-17-ol, 17-methyl-, (5-.alpha.,17-.beta.)- $$ 17-.alpha.-Methyl-(5-.alpha.)-.delta.

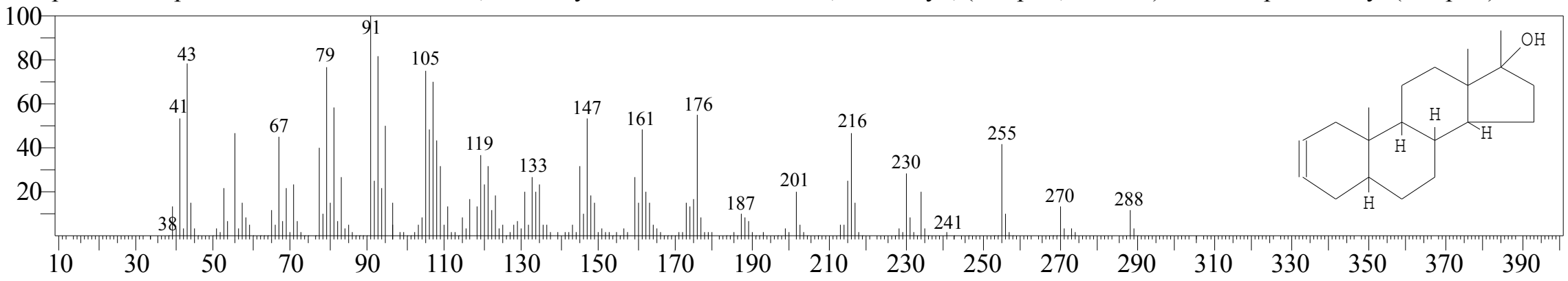


Hit#:3 Entry:192706 Library:NIST17-1.lib
SI:75 Formula:C20H30O5 CAS:5508-58-7 MolWeight:350 RetIndex:2944
CompName:Andrographolide $$ 2(3H)-Furanone, 3-[2-[decahydro-6-hydroxy-5-(hydroxymethyl)-5,8a-dimethyl-2-methylene-1-naphthalenyl]ethylidene]dih

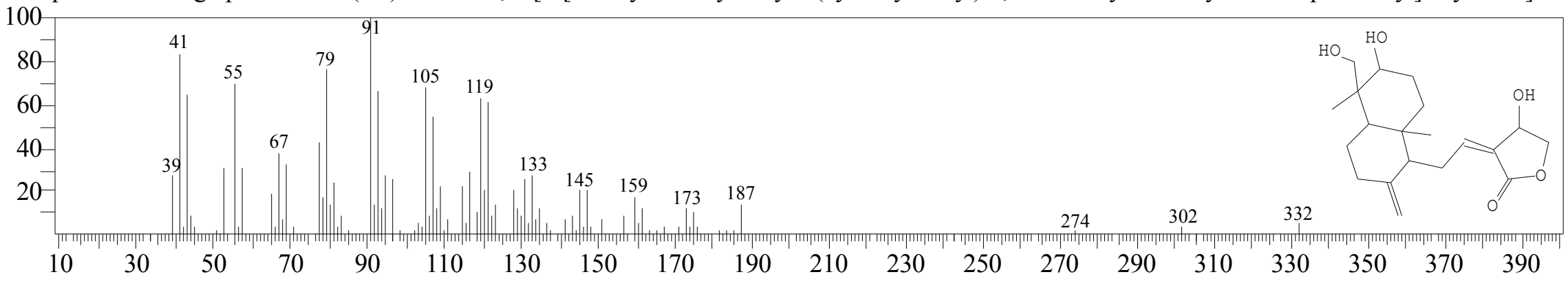


Hit#:4 Entry:162389 Library:NIST17-1.lib
SI:74 Formula:C21H34O2 CAS:57344-99-7 MolWeight:318 RetIndex:2251
CompName:Androstan-17-one, 3-ethyl-3-hydroxy-, (5.alpha.)- $$ 3-Ethyl-3-hydroxyandrostan-17-one # $$

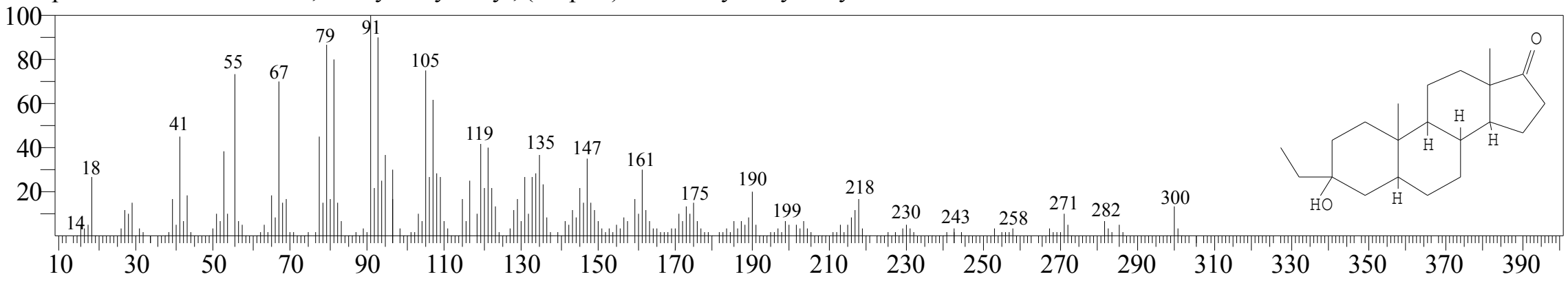


Hit#:5 Entry:52805 Library:NIST17-1.lib
SI:73 Formula:C15H24 CAS:3691-12-1 MolWeight:204 RetIndex:1490
CompName:.alpha.-Guaiene $$ Azulene, 1,2,3,4,5,6,7,8-octahydro-1,4-dimethyl-7-(1-methylethenyl)-, [1S-(1.alpha.,4.alpha.,7.alpha.)]- $$ (1S,4S,7R)-1,4-I

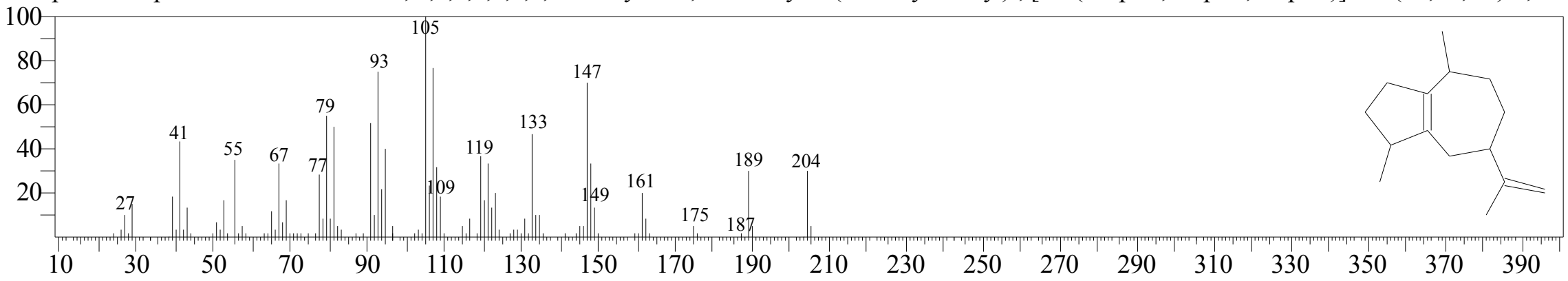

<< Target >>
Line#:16 R.Time:56.000(Scan#:10001) Retention Index:3302! MassPeaks:211
RawMode:Averaged 55.995-56.005(10000-10002) BasePeak:107.20(1738)
BG Mode:Calc. from Peak Group 1 - Event 1 Scan

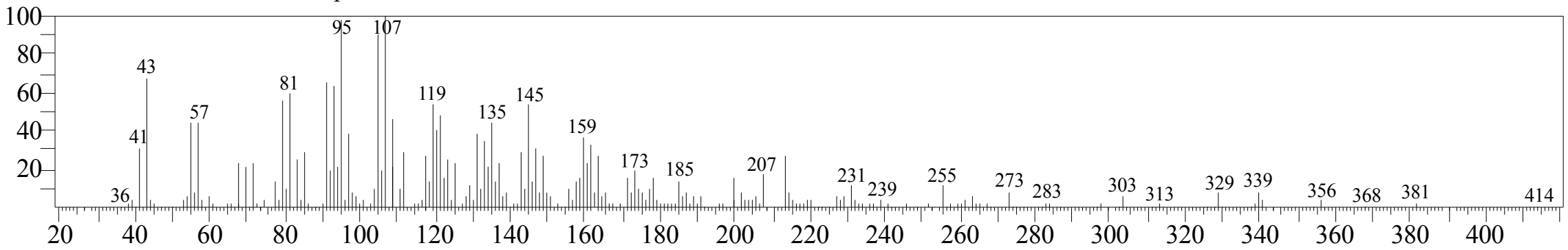


Hit#:1 Entry:230552 Library:NIST17-1.lib
SI:79 Formula:C23H34O6 CAS:560-54-3 MolWeight:406 RetIndex:3238
CompName:Card-20(22)-enolide, 3,5,14,19-tetrahydroxy-, (3.beta.,5.beta.)- $$ Strophanthidol $$ 5.beta.-Card-20(22)-enolide, 3.beta.,5,14,19-tetrahydroxy-

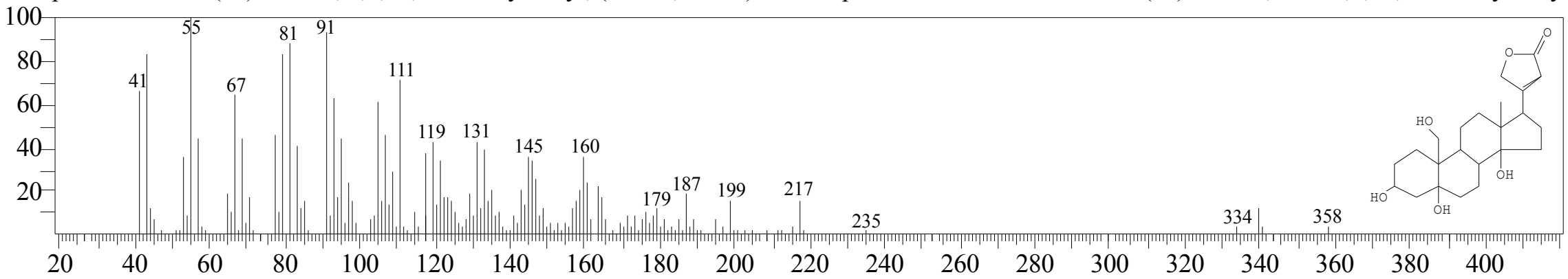


Hit#:2 Entry:131755 Library:NIST17-1.lib
SI:75 Formula:C20H32O CAS:0-00-0 MolWeight:288 RetIndex:2149
CompName:5-(7a-Isopropenyl-4,5-dimethyl-octahydroinden-4-yl)-3-methyl-penta-2,4-dien-1-ol $$ (2E,4Z)-5-(7a-Isopropenyl-4,5-dimethyloctahydro-1H-in

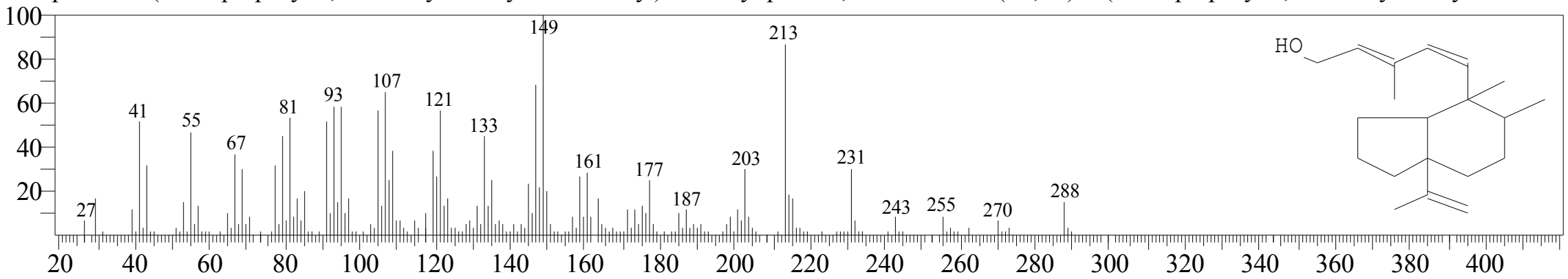


Hit#:3 Entry:192706 Library:NIST17-1.lib
SI:73 Formula:C20H30O5 CAS:5508-58-7 MolWeight:350 RetIndex:2944
CompName:Andrographolide $$ 2(3H)-Furanone, 3-[2-[decahydro-6-hydroxy-5-(hydroxymethyl)-5,8a-dimethyl-2-methylene-1-naphthalenyl]ethylidene]dih

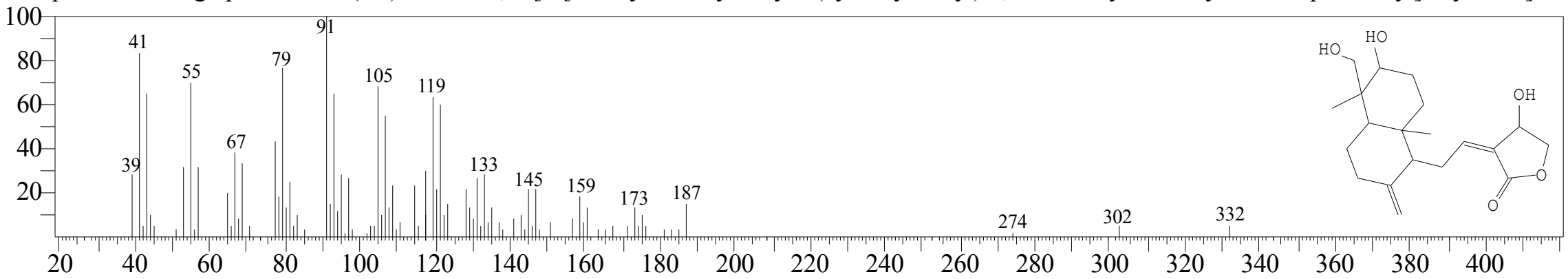


Hit#:4 Entry:68664 Library:NIST17-1.lib
SI:72 Formula:C15H26O CAS:489-41-8 MolWeight:222 RetIndex:1530
CompName:(-)-Globulol $$ (1aR,4R,4aR,7R,7aS,7bS)-1,1,4,7-Tetramethyldecahydro-1H-cyclopropa[e]azulen-4-ol $$ Globulol $$ 1H-Cycloprop[e]azulen-

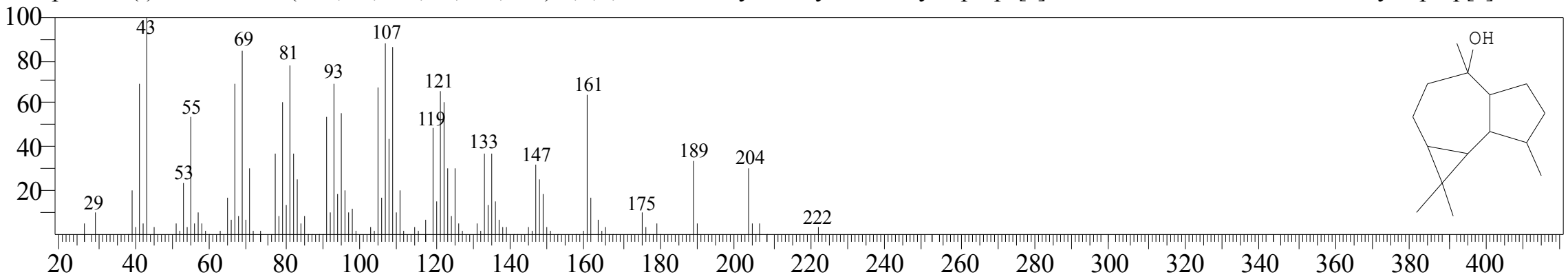


Hit#:5 Entry:37732 Library:NIST17s.lib
SI:72 Formula:C23H32O6 CAS:66-28-4 MolWeight:404 RetIndex:3184
CompName:Strophanthidin $$ Card-20(22)-enolide, 3,5,14-trihydroxy-19-oxo-, (3.beta.,5.beta.)- $$ 5.beta.-Card-20(22)-enolide, 3.beta.,5,14-trihydroxy-19

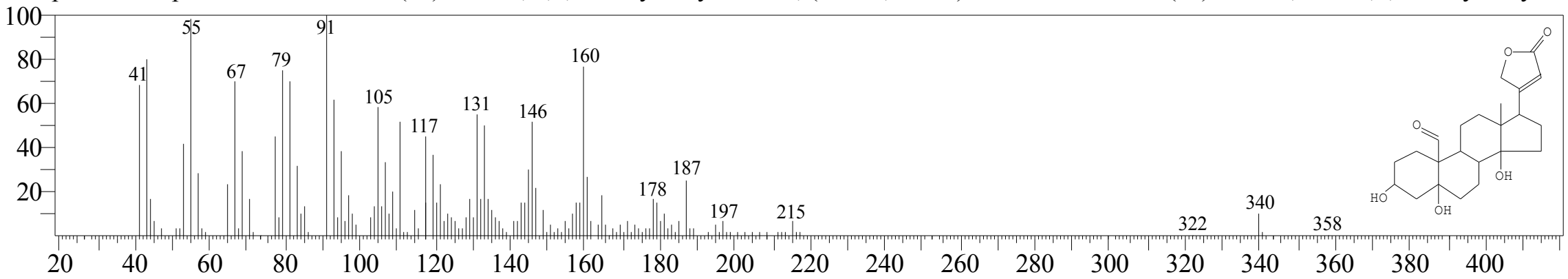


Method

[Comment]

═══ Analytical Line 1 ═══

[AOC-20i]
# of Rinses with Presolvent :3
# of Rinses with Solvent(post) :3
# of Rinses with Sample :2
Plunger Speed(Suction) :High

Viscosity Comp. Time :0.2 sec
Plunger Speed(Injection) :High
Syringe Insertion Speed :High
Injection Mode :Normal
Pumping Times :5
Inj. Port Dwell Time :0.3 sec
Terminal Air Gap :No
Plunger Washing Speed :High
Washing Volume :8uL
Syringe Suction Position :0.0 mm
Syringe Injection Position :0.0 mm
Use 3 Solvent Vial :1 vial

[GC-2010]
Column Oven Temp. :50.0 °C
Injection Temp. :280.00 °C
Injection Mode :Split
Flow Control Mode :Pressure
Pressure :80.0 kPa
Total Flow :11.2 mL/min
Column Flow :1.37 mL/min
Linear Velocity :42.5 cm/sec
Purge Flow :3.0 mL/min
Split Ratio :5.0
High Pressure Injection :OFF
Carrier Gas Saver :OFF
Splitter Hold :OFF
Oven Temp. Program

| Rate | Temperature(°C) | Hold Time(min) |
|---|---|---|
| - | 50.0 | 3.00 |
| 5.00 | 300.0 | 10.00 |

< Ready Check Heat Unit >
Column Oven : Yes
SPL1 : Yes
MS : Yes
< Ready Check Detector(FTD/BID) >
< Ready Check Baseline Drift >
< Ready Check Injection Flow >
SPL1 Carrier : Yes
SPL1 Purge : Yes
< Ready Check APC Flow >
< Ready Check Detector APC Flow >
External Wait :No
Equilibrium Time :3.0 min

[GC Program]

[GCMS-QP2020]
IonSourceTemp :220.00 °C
Interface Temp. :280.00 °C
Solvent Cut Time :5.50 min
Detector Gain Mode :Relative to the Tuning Result
Detector Gain :0.84 kV +0.00 kV
Threshold :0

[MS Table]
--Group 1 - Event 1--
Start Time :6.00min
End Time :63.00min
ACQ Mode :Scan
Event Time :0.30sec
Scan Speed :1666
Start m/z :35.00
End m/z :500.00

Sample Inlet Unit :GC

[MS Program]
Use MS Program :OFF
