# Supplementary material for: Insecticidal metabolites from Serratia marcescens associated with the fall armyworm (Spodoptera frugiperda): molecular identification, metabolomic profiling and molecular docking insights
Source: Sci Rep. 2026 Aug 19;16:26057. doi: 10.1038/s41598-026-66375-7 (PMC13490546; doi:10.1038/s41598-026-66375-7)
Supplement: Supplementary file 2 [file 41598_2026_66375_MOESM2_ESM.pdf]

Center for DRUG DISCOVERY RESEARCH and DEVELOPMENT

Openlynx Report -

Sample: 122 Vial:1:A,2 ID:
File:E2479R Date:06-Nov-2024 Time:23:37:05
Description:Eth.AC (2)

Printed: Sun Nov 24 12:56:41 2024

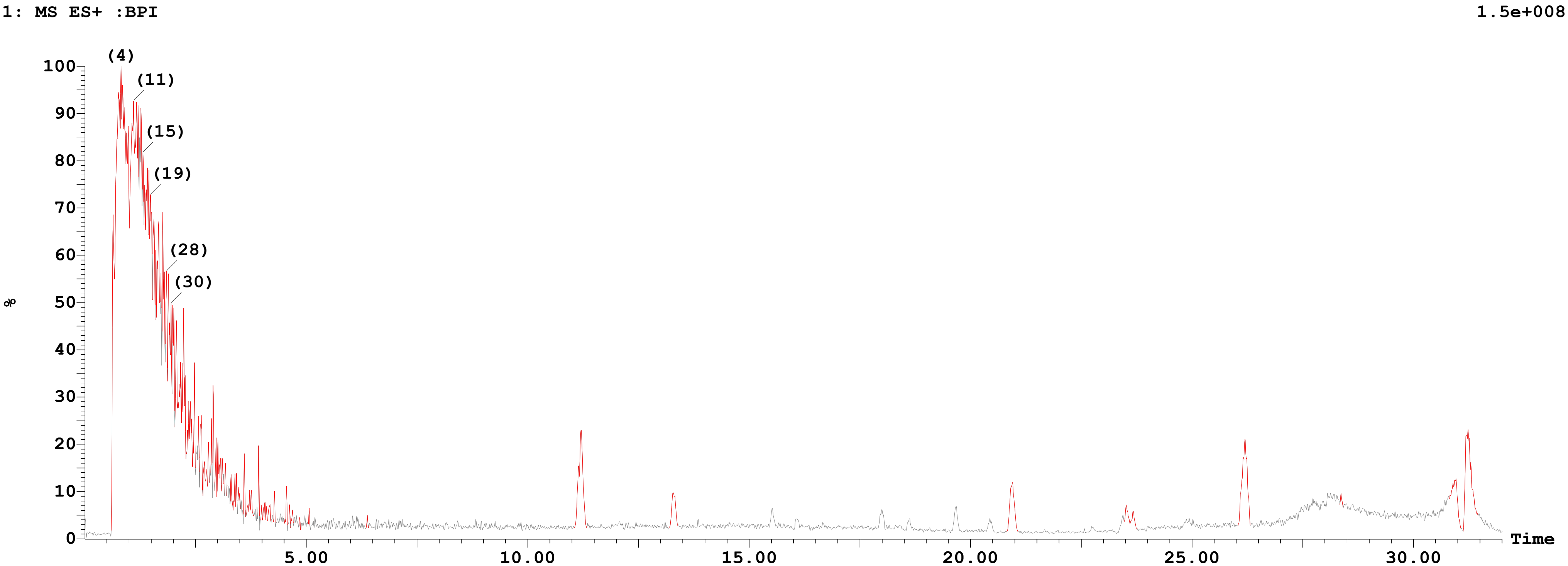

Peak Number	Time	AreaAbs	Area %Total
1	0.64	4507483.5000	5.77
2	0.73	5241811.0000	6.71
3	0.76	6921047.5000	8.86
4	0.82	3334375.7500	4.27
5	0.85	3014336.2500	3.86
6	0.89	4326839.0000	5.54
7	0.94	2407751.2500	3.08
8	0.98	1560228.8750	2.00
9	1.06	1727088.3750	2.21
10	1.06	1067151.6250	1.37
11	1.10	2181437.7500	2.79
12	1.17	844899.5625	1.08
13	1.20	527751.3125	0.68
14	1.27	266192.5313	0.34

Sample: 122 Vial:1:A,2 ID:
File:E2479R Date:06-Nov-2024 Time:23:37:05
Description:Eth.AC (2)

Printed: Sun Nov 24 12:56:41 2024

15	1.31	199572.9219	0.26
16	1.35	288092.3750	0.37
17	1.41	1013361.5625	1.30
18	1.45	716895.9375	0.92
19	1.48	574281.4375	0.74
20	1.50	610308.6250	0.78
21	1.54	188019.8281	0.24
22	1.57	554343.2500	0.71
23	1.60	293887.8438	0.38
24	1.67	855887.8125	1.10
25	1.72	84883.4297	0.11
26	1.76	855901.4375	1.10
27	1.80	539758.0625	0.69
28	1.85	685767.1250	0.88
29	1.89	1035362.6875	1.33
30	1.95	496560.4063	0.64
31	1.98	268724.0625	0.34
32	2.01	389070.0000	0.50
33	2.07	1048756.3750	1.34
34	2.13	507318.4375	0.65
35	2.16	702368.8125	0.90
36	2.20	536381.8125	0.69
37	2.23	972729.7500	1.25
38	2.26	561167.3750	0.72
39	2.32	166726.8125	0.21
40	2.35	390382.0000	0.50
41	2.38	462972.0313	0.59
42	2.41	237241.8125	0.30
43	2.48	763224.0625	0.98
44	2.57	179386.5938	0.23
45	2.60	343218.4375	0.44
46	2.64	480136.6250	0.61
47	2.70	373907.2188	0.48
48	2.76	170317.1406	0.22
49	2.79	239038.1406	0.31
50	2.86	190389.0781	0.24
51	2.89	530927.8125	0.68
52	2.96	341101.6875	0.44
53	3.01	516821.7500	0.66
54	3.07	261465.7031	0.33
55	3.11	55048.5313	0.07
56	3.18	184397.0000	0.24
57	3.30	172472.1563	0.22

Openlynx Report -
Sample: 122 Vial:1:A,2 ID:
File:E2479R Date:06-Nov-2024 Time:23:37:05
Description:Eth.AC (2)

Printed: Sun Nov 24 12:56:41 2024

58	3.39	250149.3438	0.32
59	3.43	144136.2656	0.18
60	3.46	76210.9609	0.10
61	3.49	62144.9375	0.08
62	3.60	370292.4688	0.47
63	3.69	108390.2656	0.14
64	3.72	184628.7656	0.24
65	3.76	187816.6250	0.24
66	3.92	382066.5000	0.49
67	3.99	86739.4219	0.11
68	4.03	76450.6953	0.10
69	4.07	137512.9219	0.18
70	4.10	81765.3281	0.10
71	4.18	163551.1563	0.21
72	4.28	191980.9531	0.25
73	4.55	252273.7656	0.32
74	4.62	100595.8203	0.13
75	4.69	134477.5156	0.17
76	4.85	64497.6406	0.08
77	5.07	91766.0391	0.12
78	6.38	86563.8750	0.11
79	11.15	900238.6875	1.15
80	11.20	2591268.0000	3.32
81	13.28	728134.0000	0.93
82	13.32	336580.1875	0.43
83	20.94	1804611.0000	2.31
84	23.51	355366.1250	0.46
85	23.67	335871.7500	0.43
86	26.16	1383154.3750	1.77
87	26.19	1426162.2500	1.83
88	26.24	864775.0625	1.11
89	28.37	128701.9688	0.16
90	30.89	368651.2500	0.47
91	30.96	1023587.1875	1.31
92	31.19	1700386.1250	2.18
93	31.23	982117.8750	1.26
94	31.26	771065.0000	0.99
95	31.29	1179696.6250	1.51

Center for DRUG DISCOVERY RESEARCH and DEVELOPMENT
Openlynx Report -

Sample: 122
File:E2479R
Description:Eth.AC (2)

Vial:1:A,2
Date:06-Nov-2024

ID:
Time:23:37:05

Printed: Sun Nov 24 12:56:41 2024

Peak ID	Time	Error PPM
1	0.64	

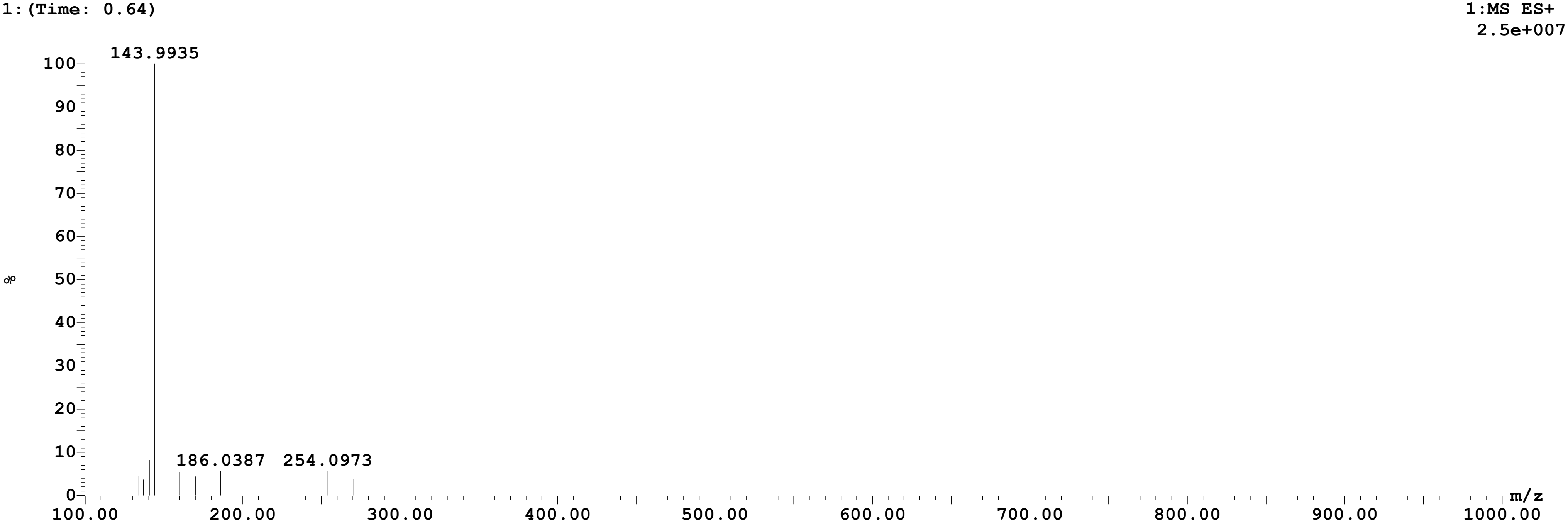

Center for DRUG DISCOVERY RESEARCH and DEVELOPMENT
Openlynx Report -

Sample: 122 Vial:1:A,2 ID:
File:E2479R Date:06-Nov-2024 Time:23:37:05
Description:Eth.AC (2)

Printed: Sun Nov 24 12:56:41 2024

Peak ID	Time	Error PPM
2	0.73	

2:(Time: 0.73)

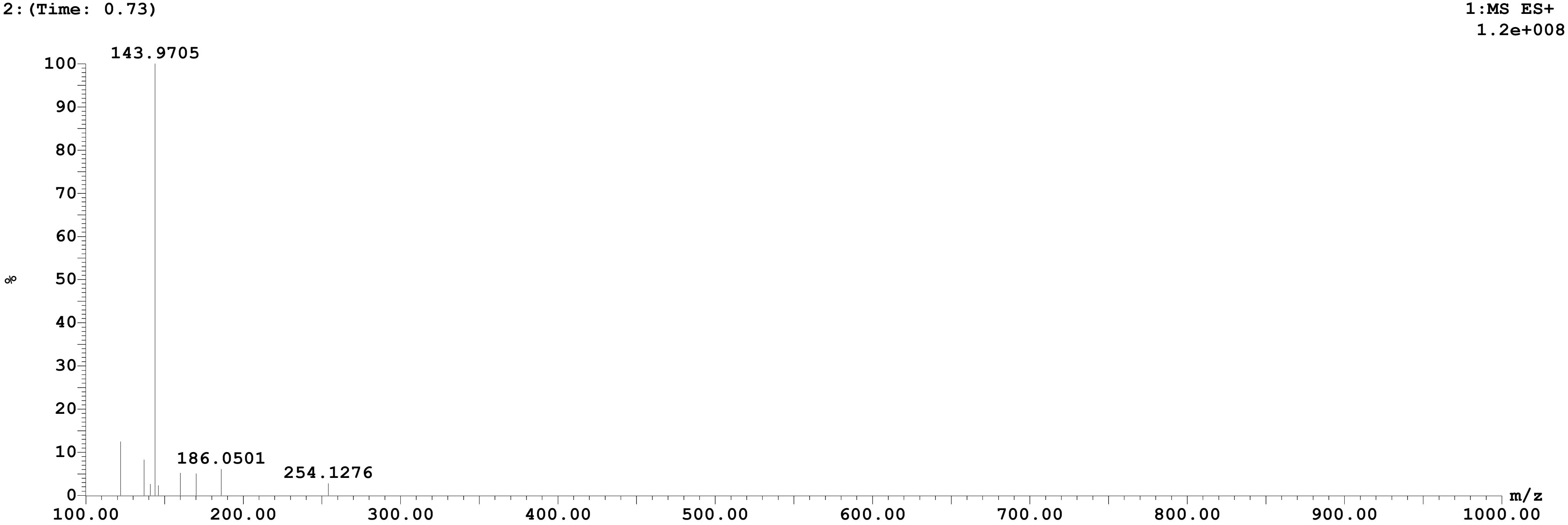

Sample: 122 Vial:1:A,2 ID:
File:E2479R Date:06-Nov-2024 Time:23:37:05
Description:Eth.AC (2)

Printed: Sun Nov 24 12:56:41 2024

Peak ID	Time	Error PPM
3	0.76	

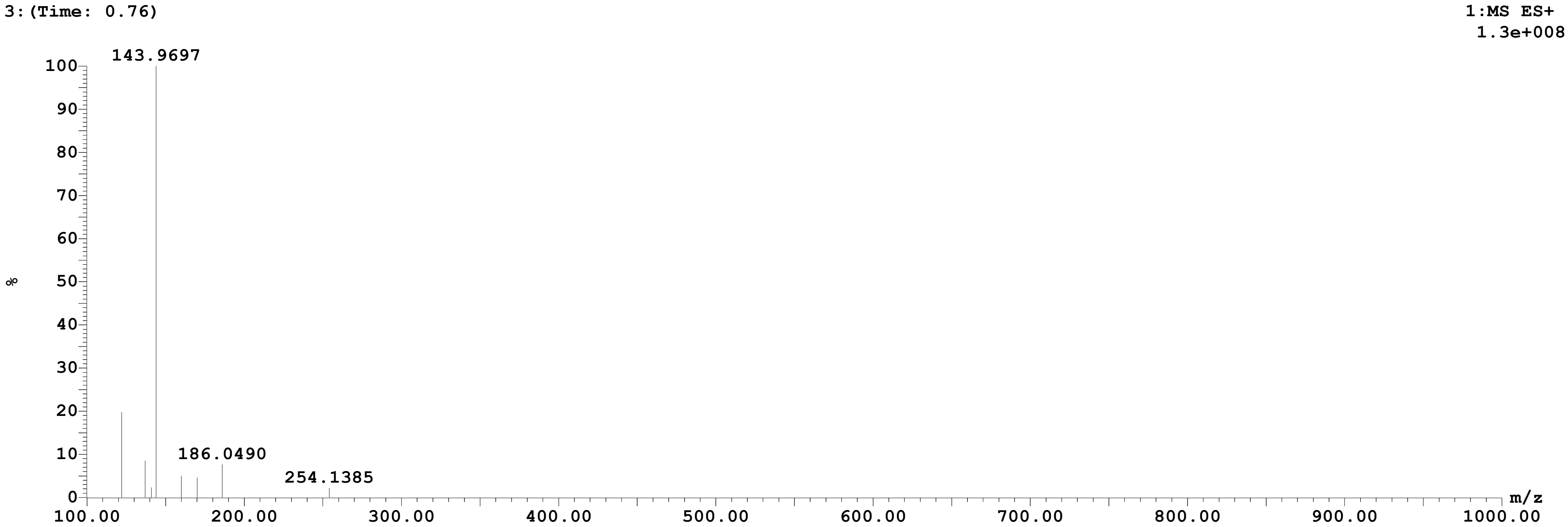

Center for DRUG DISCOVERY RESEARCH and DEVELOPMENT
Openlynx Report -

Sample: 122 Vial:1:A,2 ID:
File:E2479R Date:06-Nov-2024 Time:23:37:05
Description:Eth.AC (2)

Printed: Sun Nov 24 12:56:41 2024

Peak ID	Time	Error PPM
4	0.82	

4:(Time: 0.82)

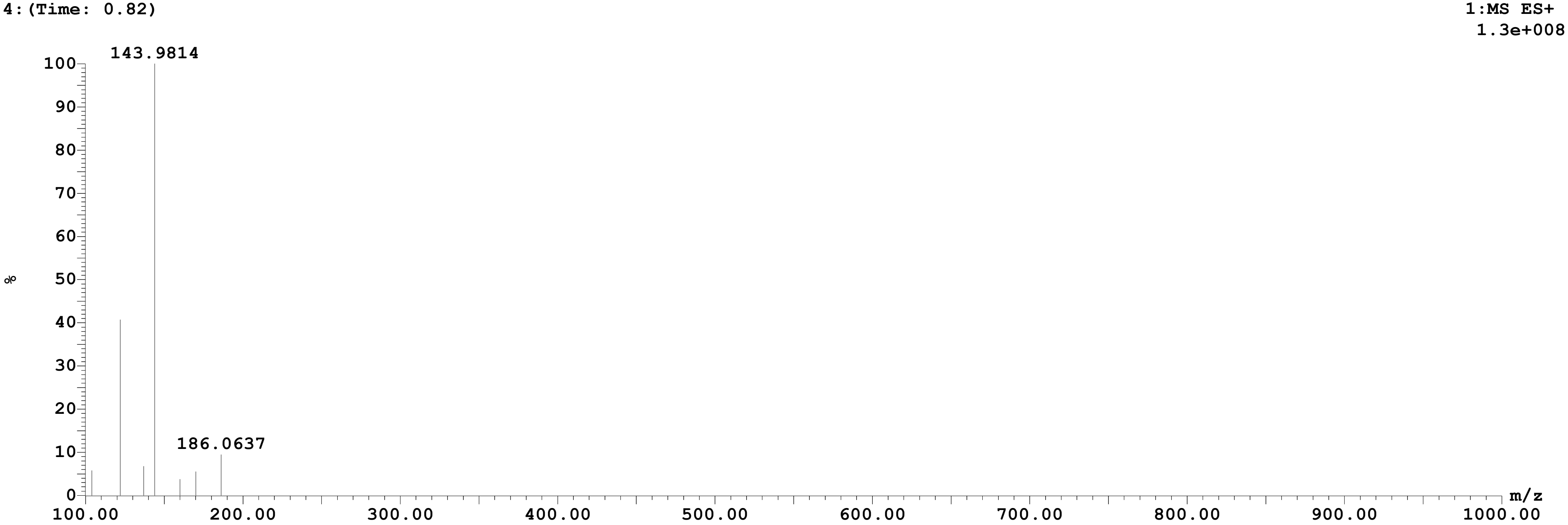

Center for DRUG DISCOVERY RESEARCH and DEVELOPMENT
Openlynx Report -

Sample: 122	Vial:1:A,2	ID:
File:E2479R	Date:06-Nov-2024	Time:23:37:05
Description:Eth.AC (2)		

Printed: Sun Nov 24 12:56:41 2024

Peak ID	Time	Error PPM
5	0.85	

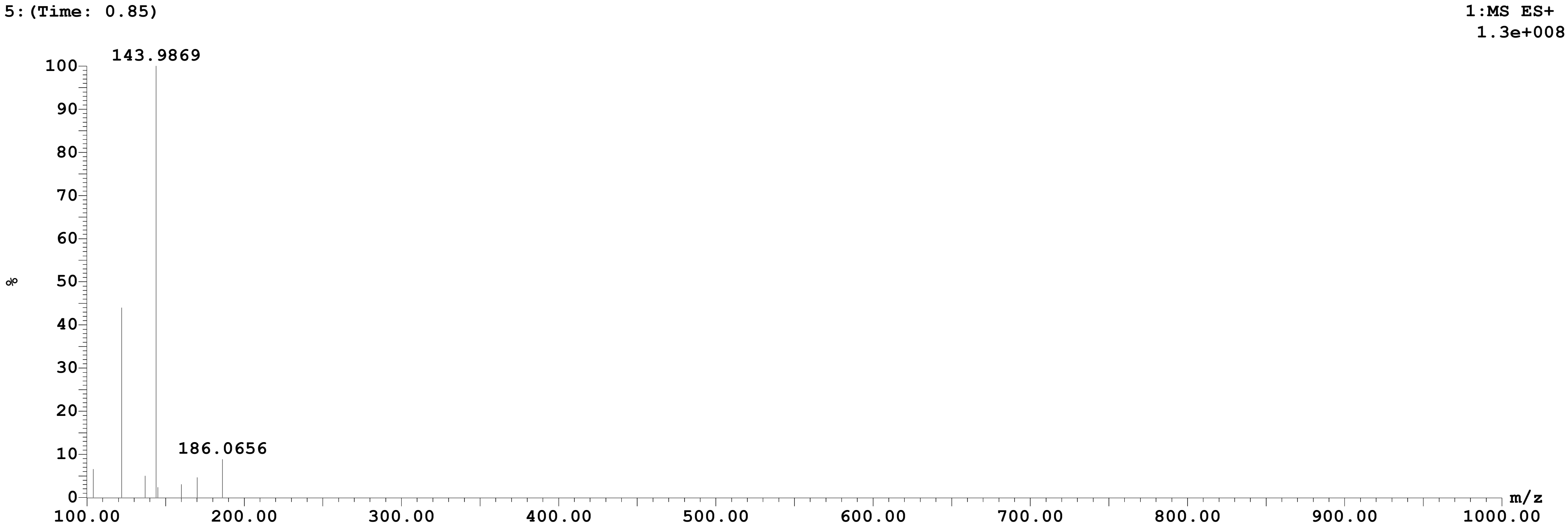

Openlynx Report -

Sample: 122
Vial:1:A,2
ID:
File:E2479R
Date:06-Nov-2024
Time:23:37:05
Description:Eth.AC (2)

Printed: Sun Nov 24 12:56:41 2024

Peak ID	Time	Error PPM
6	0.89	

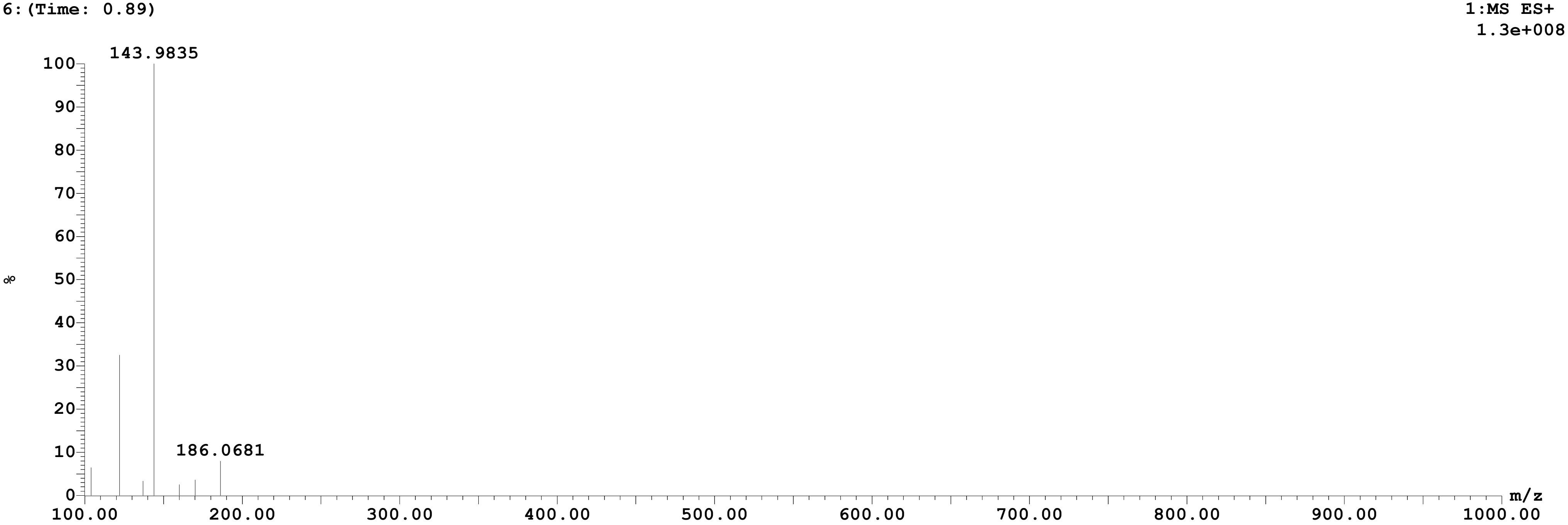

Center for DRUG DISCOVERY RESEARCH and DEVELOPMENT
Openlynx Report -

Sample: 122 Vial:1:A,2 ID:
File:E2479R Date:06-Nov-2024 Time:23:37:05
Description:Eth.AC (2)

Printed: Sun Nov 24 12:56:41 2024

Peak ID	Time	Error PPM
7	0.94	

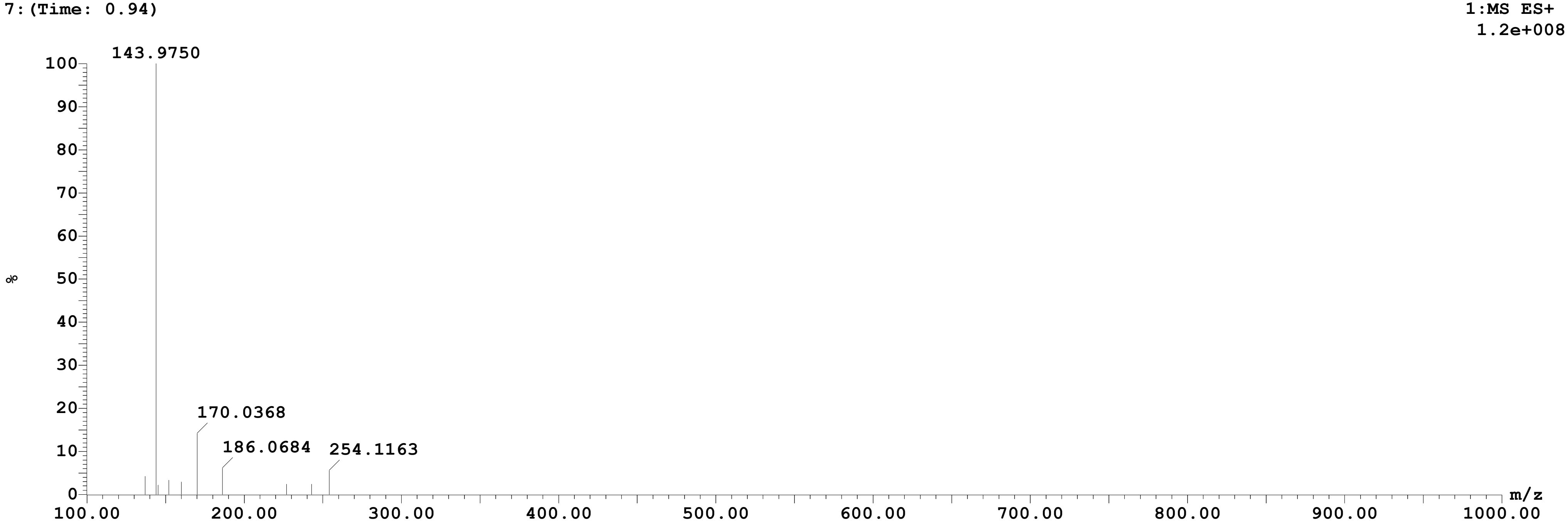

Center for DRUG DISCOVERY RESEARCH and DEVELOPMENT
Openlynx Report -

Sample: 122 | Vial:1:A,2 | ID:
File:E2479R | Date:06-Nov-2024 | Time:23:37:05
Description:Eth.AC (2)

Printed: Sun Nov 24 12:56:41 2024

Peak ID	Time	Error PPM
8	0.98	

8: (Time: 0.98)

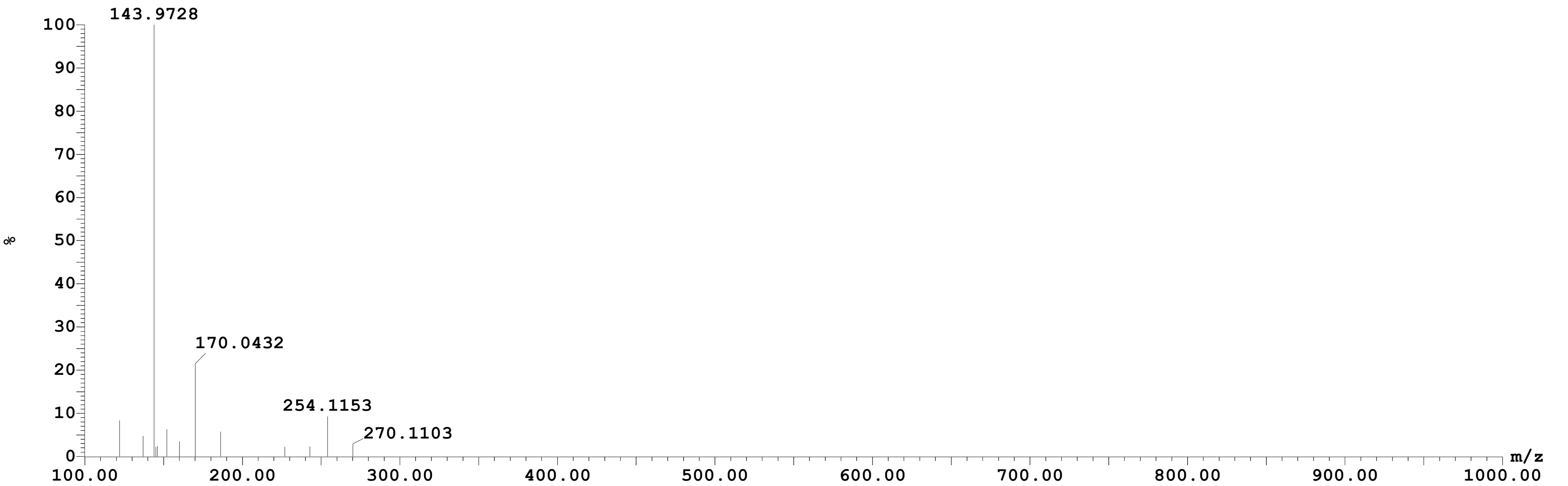

Center for DRUG DISCOVERY RESEARCH and DEVELOPMENT
Openlynx Report -

Sample: 122
File:E2479R
Description:Eth.AC (2)

Vial:1:A,2
Date:06-Nov-2024

ID:
Time:23:37:05

Printed: Sun Nov 24 12:56:41 2024

Peak ID	Time	Error PPM
9	1.06	

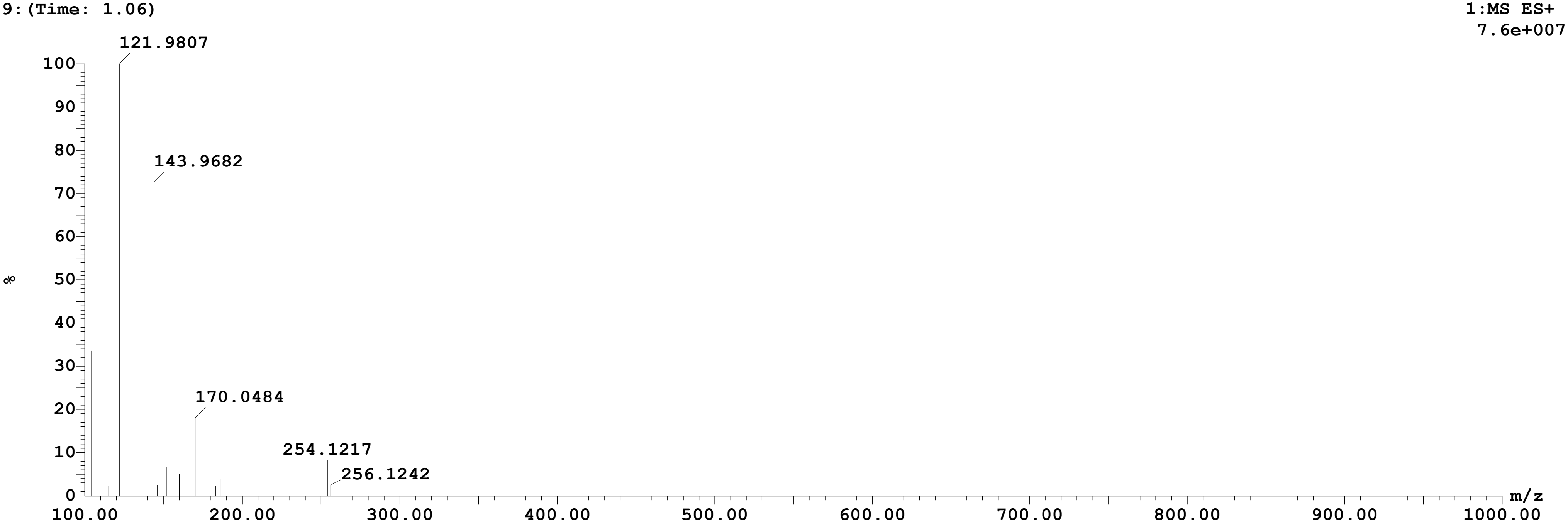

Center for DRUG DISCOVERY RESEARCH and DEVELOPMENT
Openlynx Report -

Sample: 122	Vial:1:A,2	ID:
File:E2479R	Date:06-Nov-2024	Time:23:37:05
Description:Eth.AC (2)		

Printed: Sun Nov 24 12:56:41 2024

Peak ID	Time	Error PPM
10	1.06	

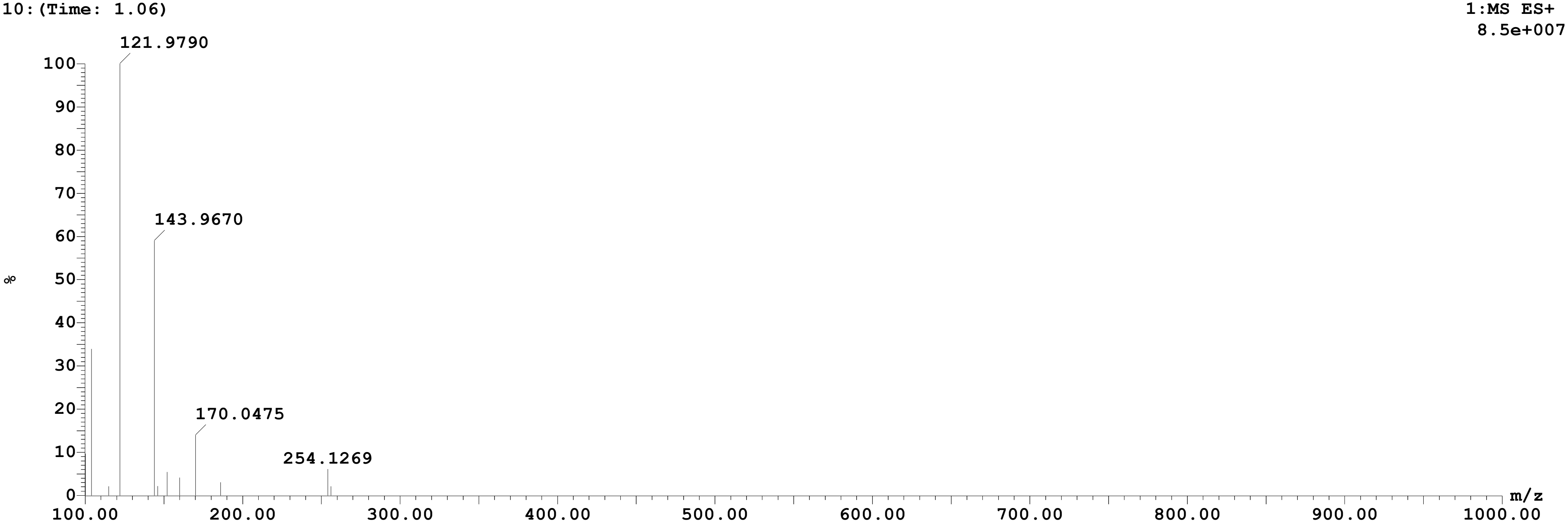

Center for DRUG DISCOVERY RESEARCH and DEVELOPMENT
Openlynx Report -

Sample: 122 | Vial:1:A,2 | ID:
File:E2479R | Date:06-Nov-2024 | Time:23:37:05
Description:Eth.AC (2)

Printed: Sun Nov 24 12:56:41 2024

Peak ID	Time	Error PPM
11	1.10	

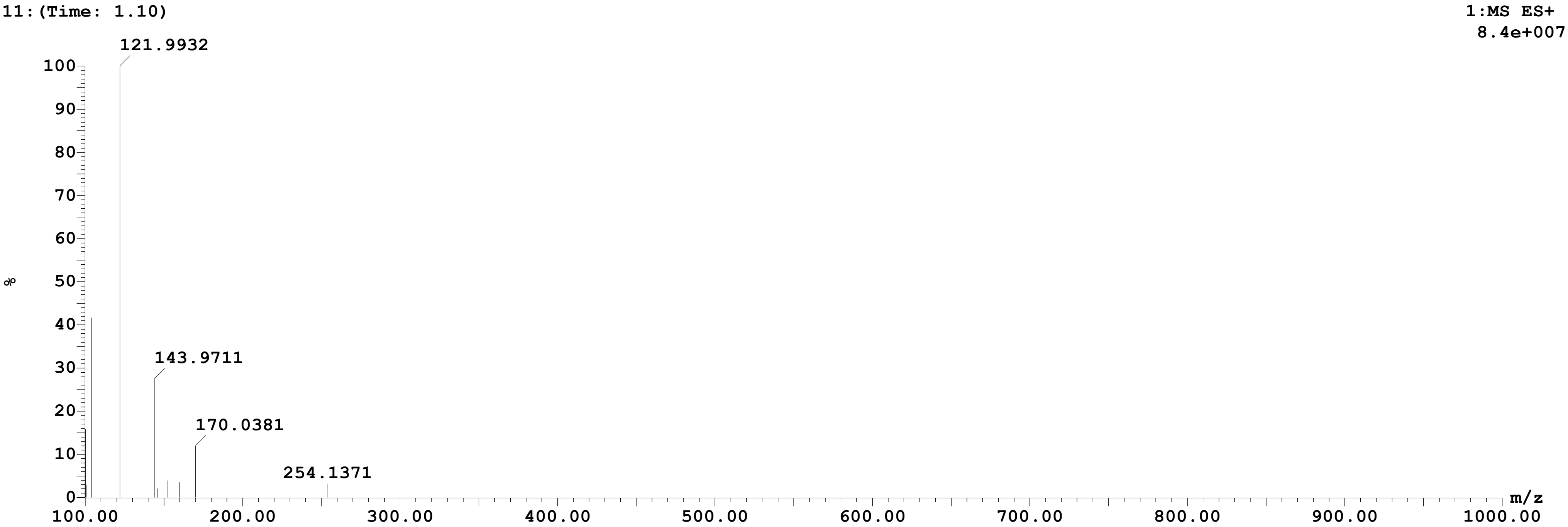

Openlynx Report -

Sample: 122 Vial:1:A,2 ID:
File:E2479R Date:06-Nov-2024 Time:23:37:05
Description:Eth.AC (2)

Printed: Sun Nov 24 12:56:41 2024

**Peak ID**	**Time**	**Error PPM**
12	1.17	

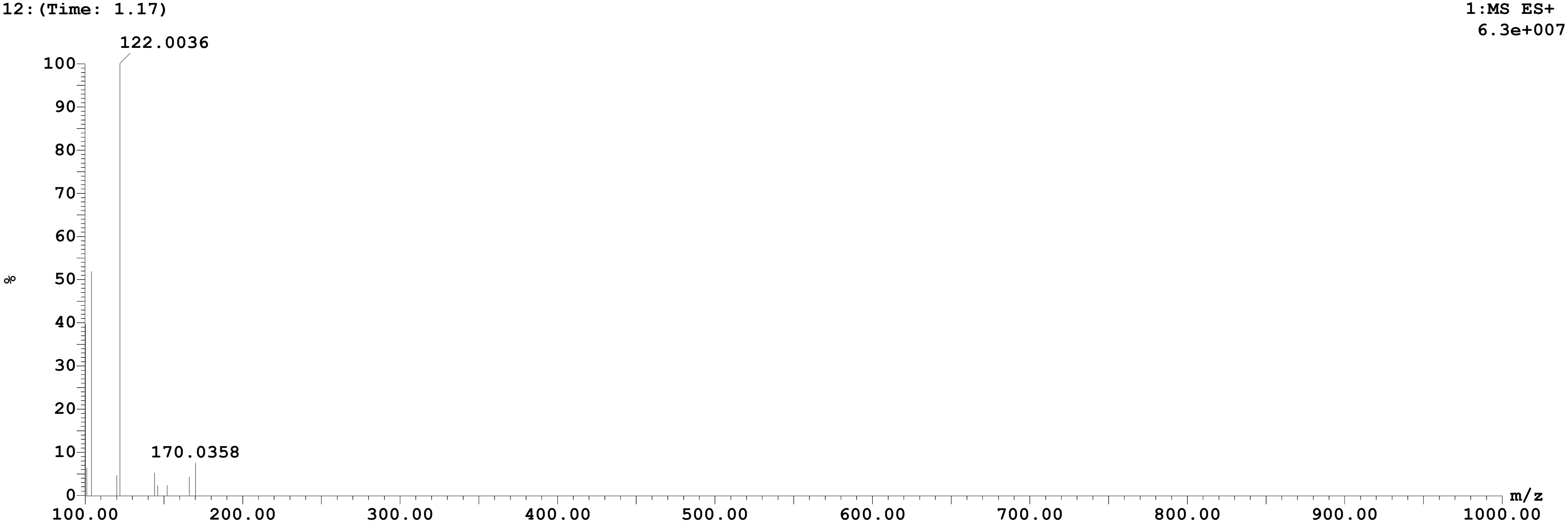

Center for DRUG DISCOVERY RESEARCH and DEVELOPMENT
Openlynx Report -

Sample: 122
Vial:1:A,2
ID:
File:E2479R
Date:06-Nov-2024
Time:23:37:05
Description:Eth.AC (2)

Printed: Sun Nov 24 12:56:41 2024

Peak ID	Time	Error PPM
13	1.20	

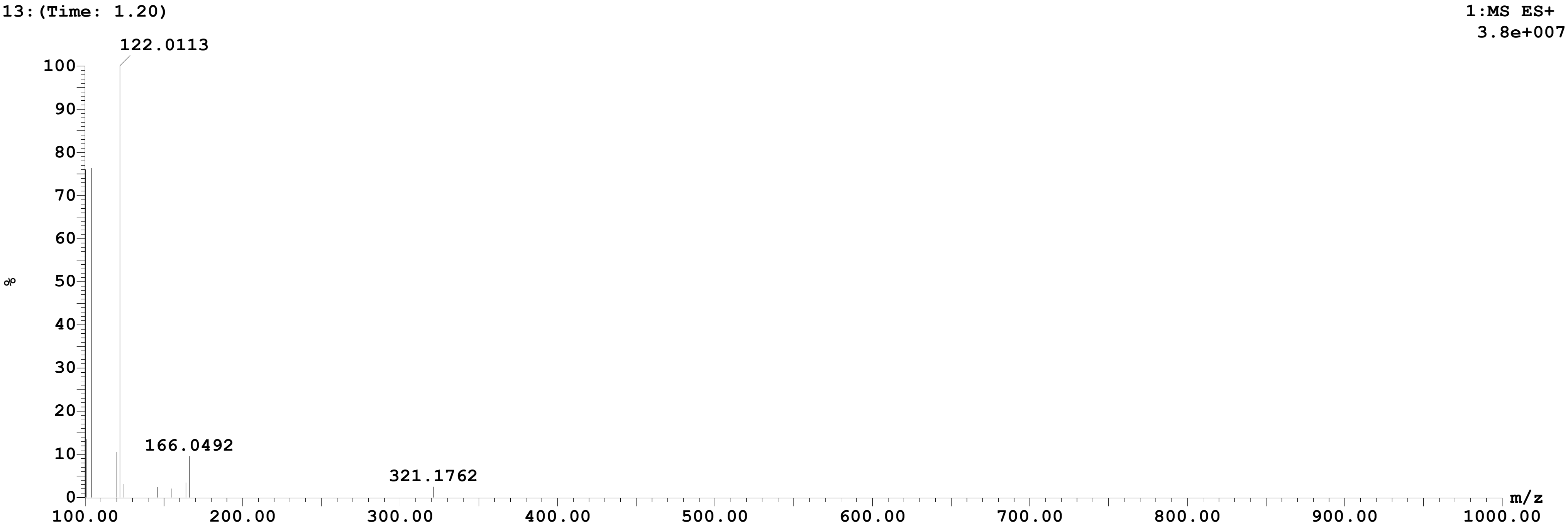

Center for DRUG DISCOVERY RESEARCH and DEVELOPMENT
Openlynx Report -

Sample: 122 Vial:1:A,2 ID:
File:E2479R Date:06-Nov-2024 Time:23:37:05
Description:Eth.AC (2)

Printed: Sun Nov 24 12:56:41 2024

Peak ID	Time	Error PPM
14	1.27	

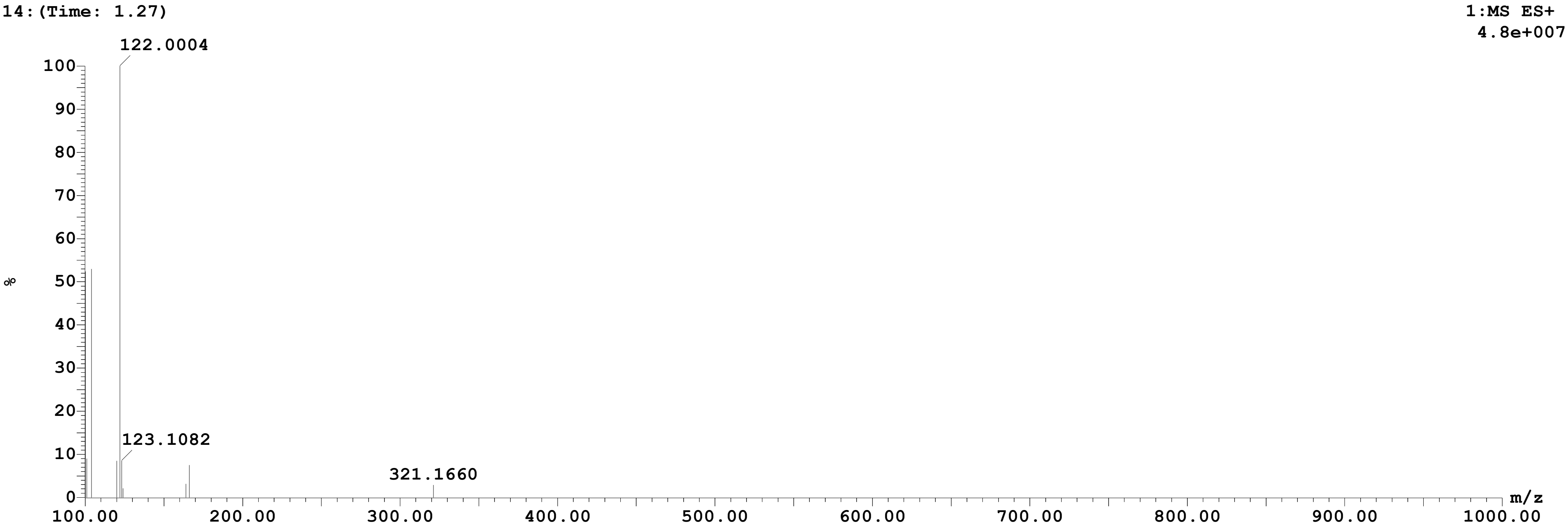

Center for DRUG DISCOVERY RESEARCH and DEVELOPMENT
Openlynx Report -

Sample: 122
File:E2479R
Description:Eth.AC (2)

Vial:1:A,2
Date:06-Nov-2024

ID:
Time:23:37:05

Printed: Sun Nov 24 12:56:41 2024

Peak ID	Time	Error PPM
15	1.31	

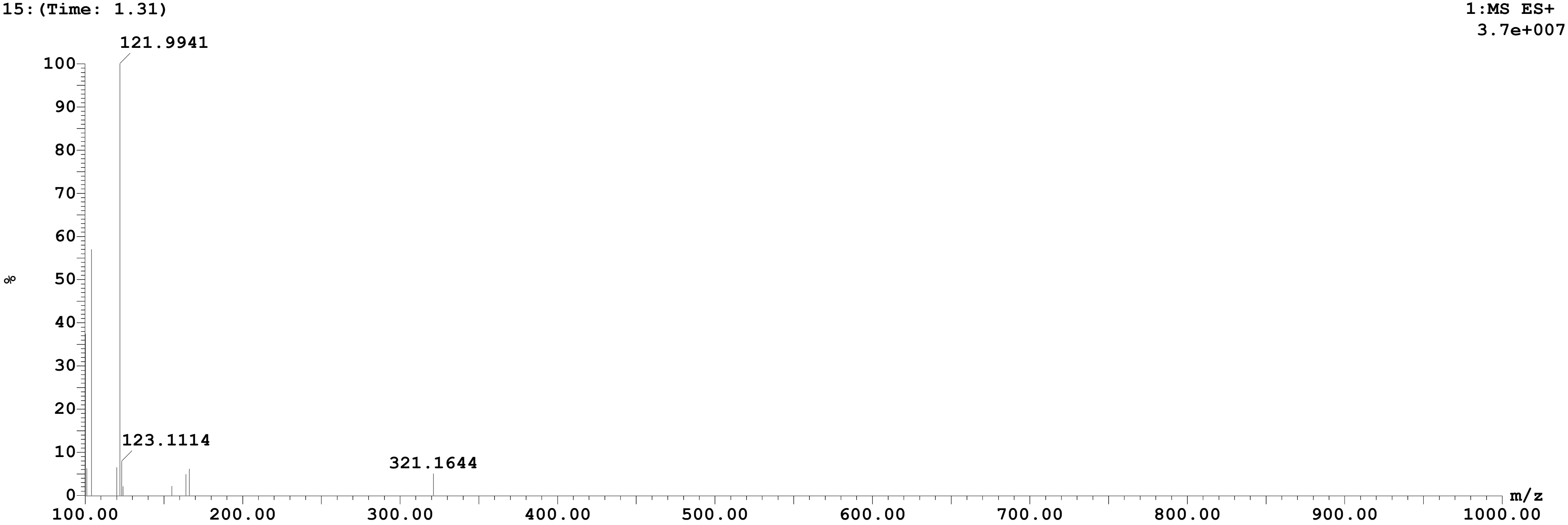

Center for DRUG DISCOVERY RESEARCH and DEVELOPMENT
Openlynx Report -

Sample: 122 | Vial:1:A,2 | ID:
File:E2479R | Date:06-Nov-2024 | Time:23:37:05
Description:Eth.AC (2)

Printed: Sun Nov 24 12:56:41 2024

Peak ID	Time	Error PPM
16	1.35	

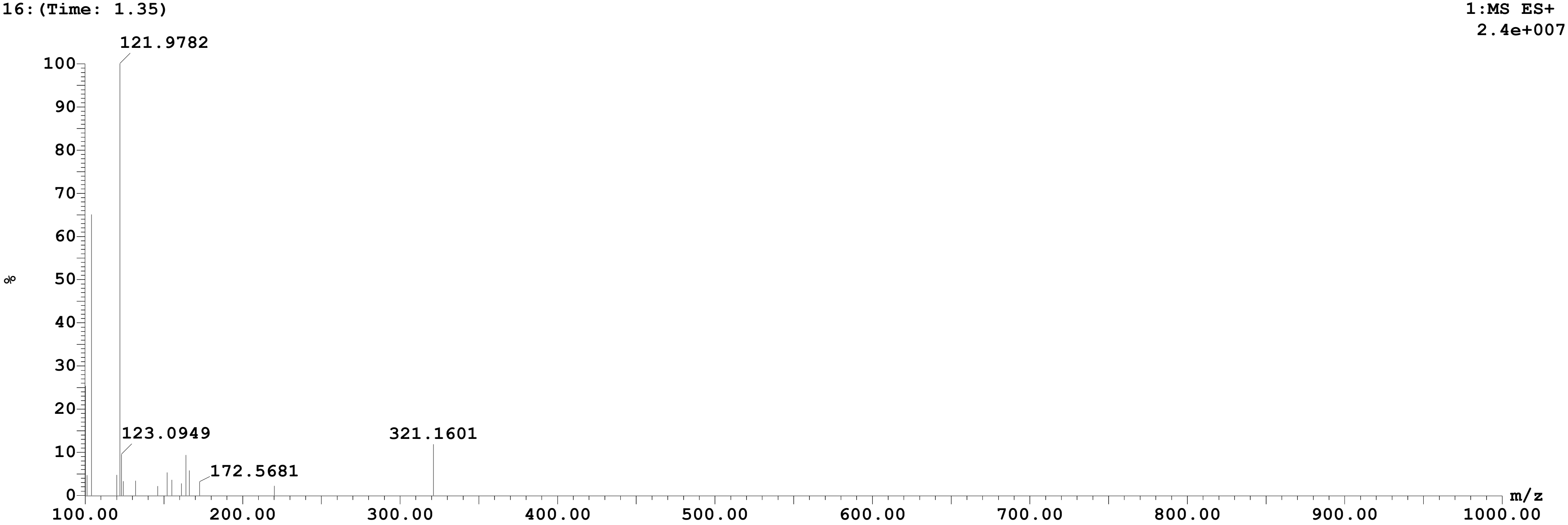

Center for DRUG DISCOVERY RESEARCH and DEVELOPMENT
Openlynx Report -

Sample: 122
File:E2479R
Description:Eth.AC (2)

Vial:1:A,2
Date:06-Nov-2024

ID:
Time:23:37:05

Printed: Sun Nov 24 12:56:41 2024

Peak ID	Time	Error PPM
17	1.41	

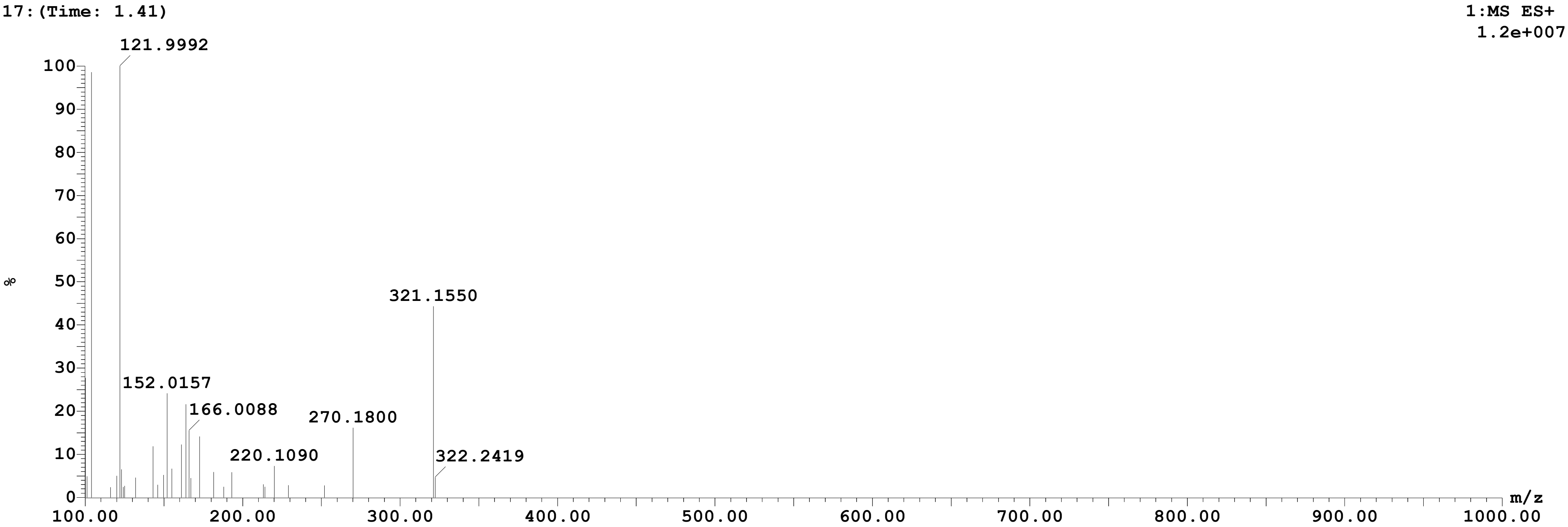

Center for DRUG DISCOVERY RESEARCH and DEVELOPMENT
Openlynx Report -

Sample: 122
File:E2479R
Description:Eth.AC (2)
Vial:1:A,2
Date:06-Nov-2024
ID:
Time:23:37:05

Printed: Sun Nov 24 12:56:41 2024

Peak ID	Time	Error PPM
18	1.45	

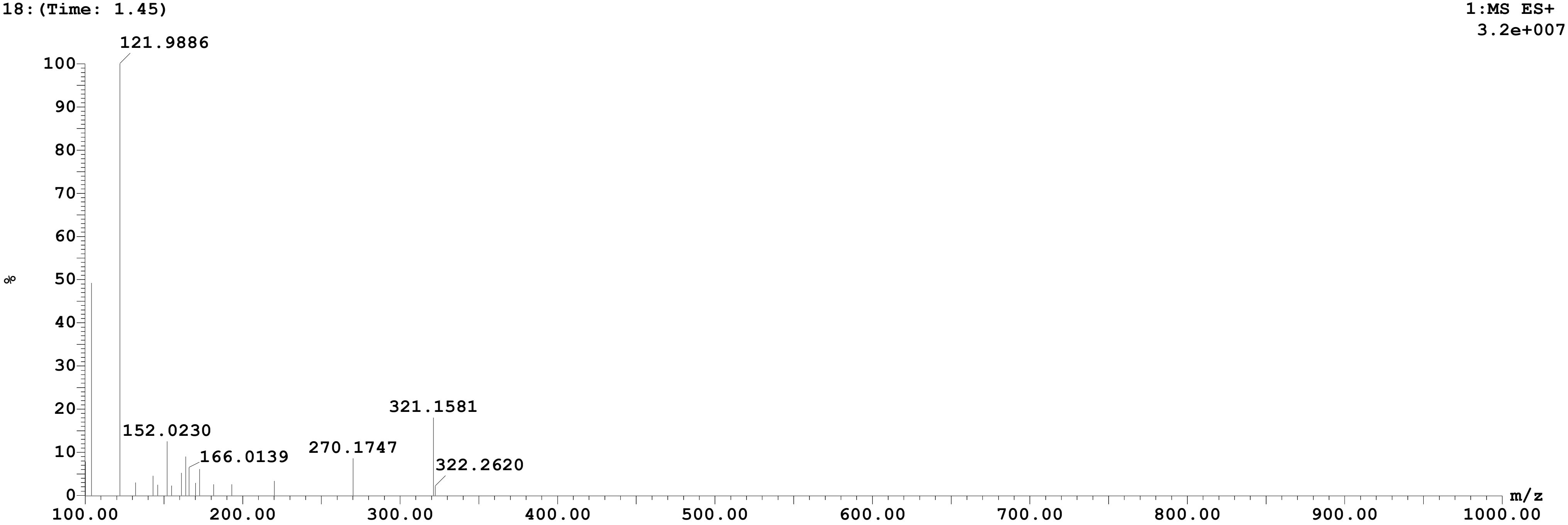

Center for DRUG DISCOVERY RESEARCH and DEVELOPMENT
Openlynx Report -

Sample: 122 | Vial:1:A,2 | ID:
File:E2479R | Date:06-Nov-2024 | Time:23:37:05
Description:Eth.AC (2)

Printed: Sun Nov 24 12:56:41 2024

Peak ID	Time	Error PPM
19	1.48	

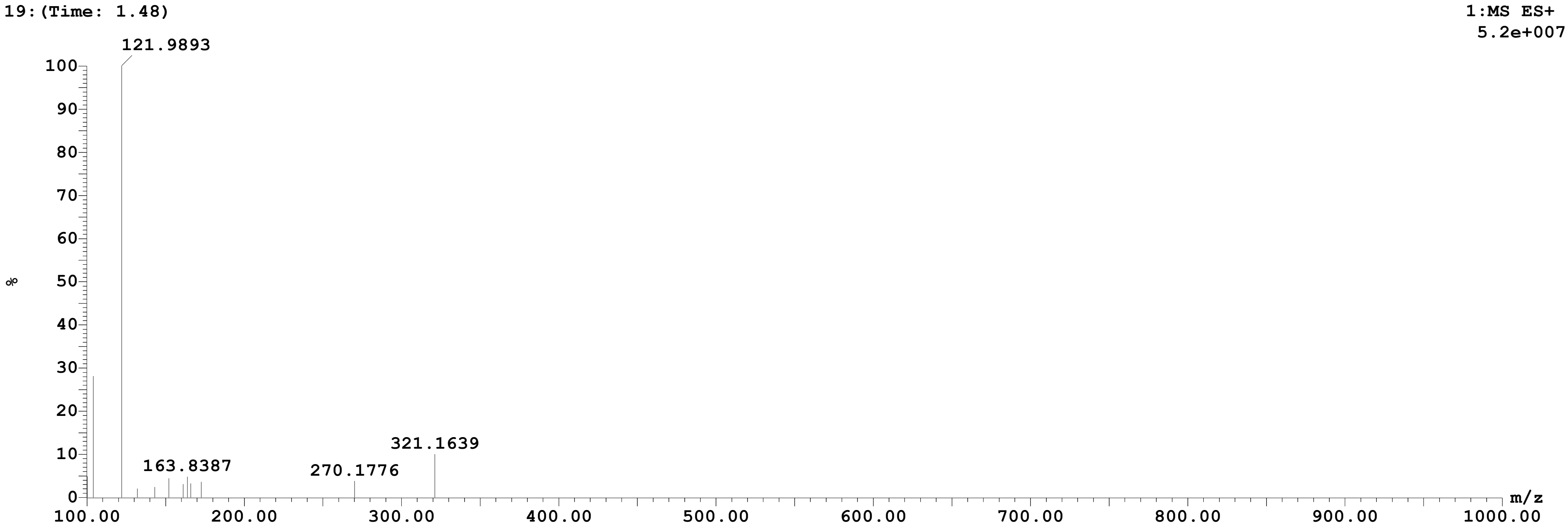

Openlynx Report -

Sample: 122 Vial:1:A,2 ID:
File:E2479R Date:06-Nov-2024 Time:23:37:05
Description:Eth.AC (2)

Printed: Sun Nov 24 12:56:41 2024

Peak ID	Time	Error PPM
20	1.50	

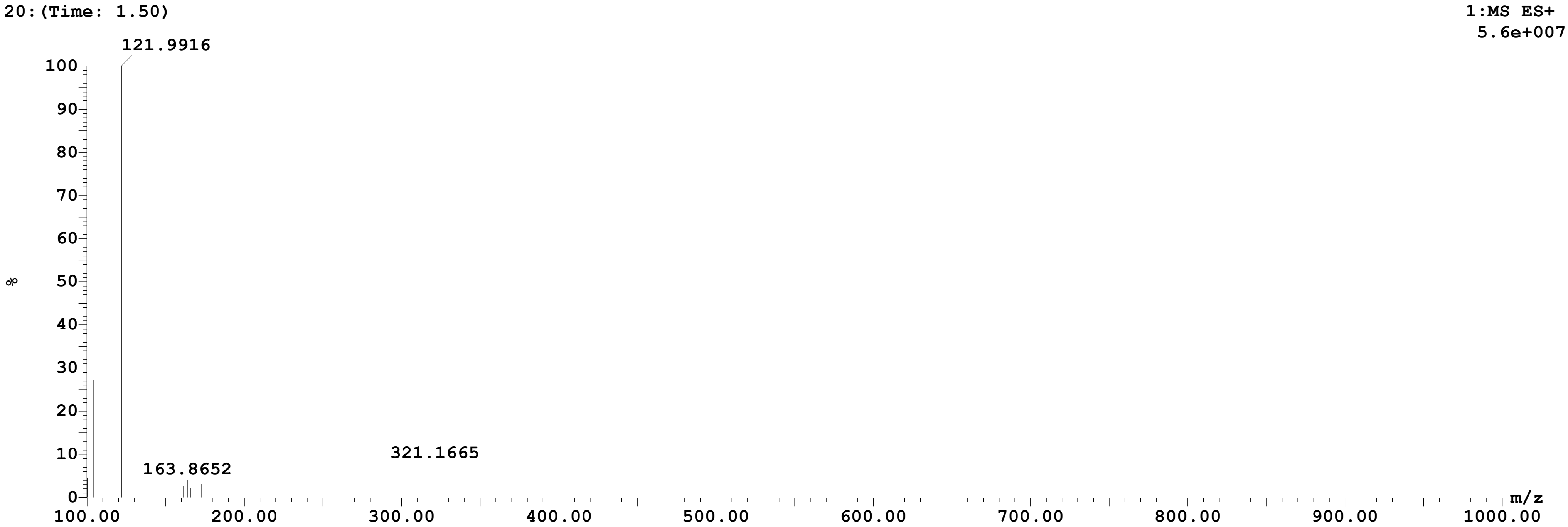

Openlynx Report -
Sample: 122 Vial:1:A,2 ID:
File:E2479R Date:06-Nov-2024 Time:23:37:05
Description:Eth.AC (2)

Printed: Sun Nov 24 12:56:41 2024

Peak ID	Time	Error PPM
21	1.54	

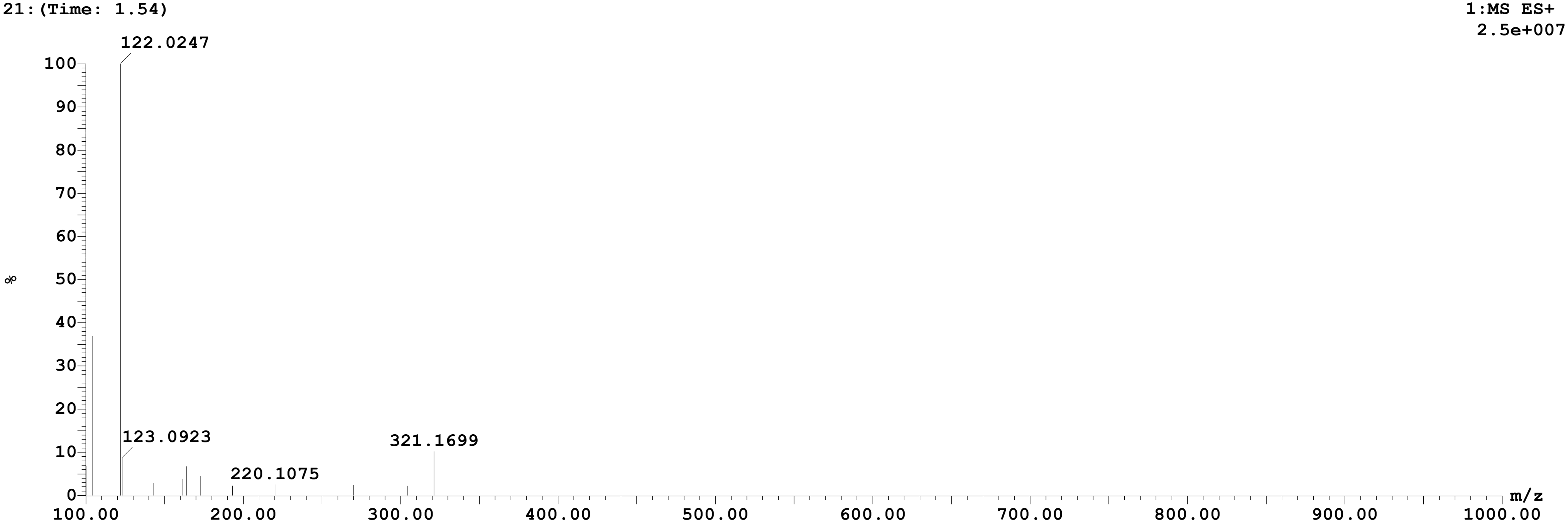

Openlynx Report -

Sample: 122 Vial:1:A,2 ID:
File:E2479R Date:06-Nov-2024 Time:23:37:05
Description:Eth.AC (2)

Printed: Sun Nov 24 12:56:41 2024

Peak ID	Time	Error PPM
22	1.57	

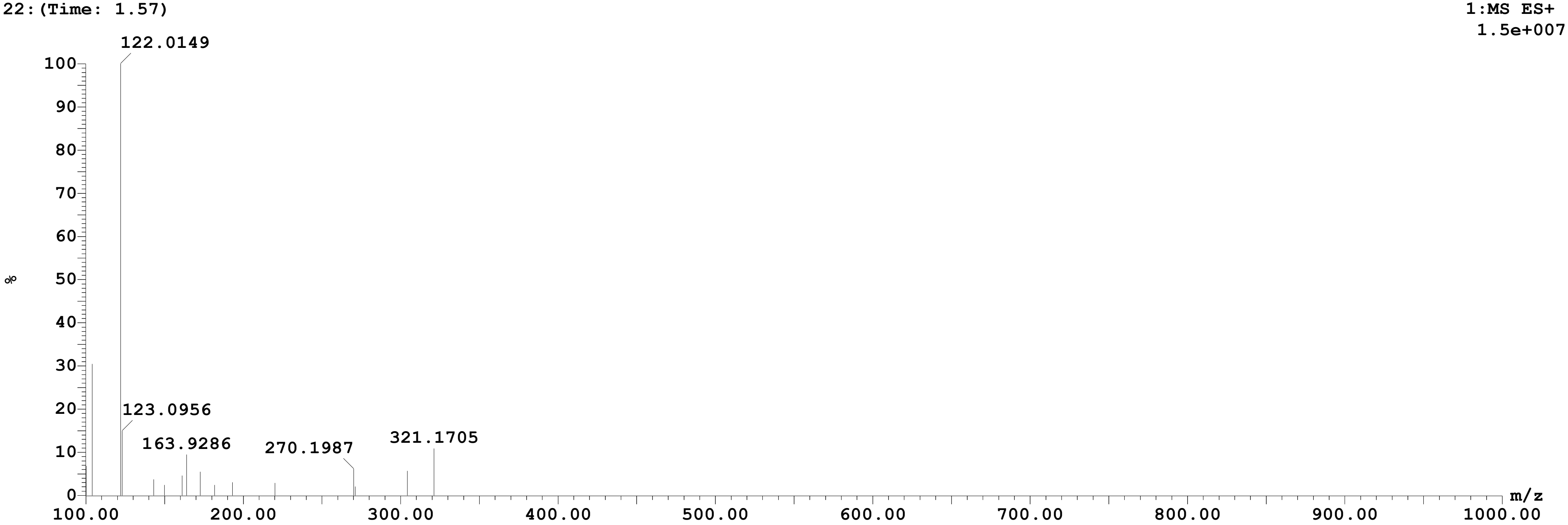

Openlynx Report -
Sample: 122 Vial:1:A,2 ID:
File:E2479R Date:06-Nov-2024 Time:23:37:05
Description:Eth.AC (2)

Printed: Sun Nov 24 12:56:41 2024

Peak ID	Time	Error PPM
23	1.60	

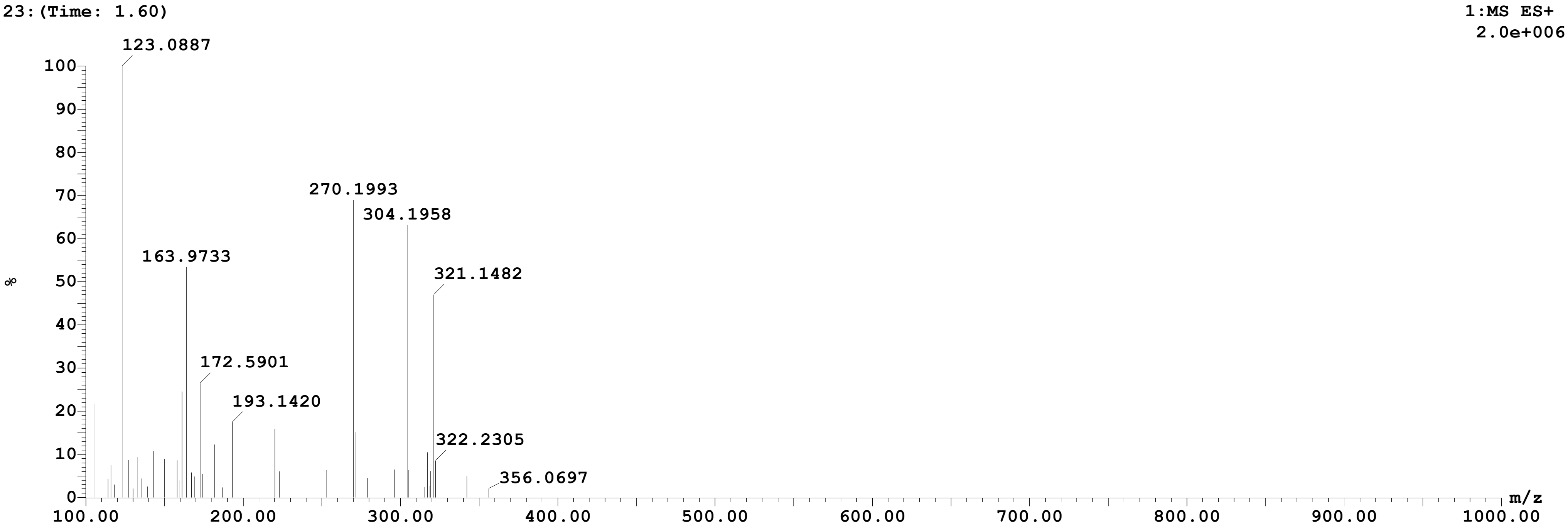

Center for DRUG DISCOVERY RESEARCH and DEVELOPMENT
Openlynx Report -

Sample: 122
File:E2479R
Description:Eth.AC (2)

Vial:1:A,2
Date:06-Nov-2024

ID:
Time:23:37:05

Printed: Sun Nov 24 12:56:41 2024

Peak ID	Time	Error PPM
24	1.67	

24:(Time: 1.67)

1:MS ES+
1.0e+006

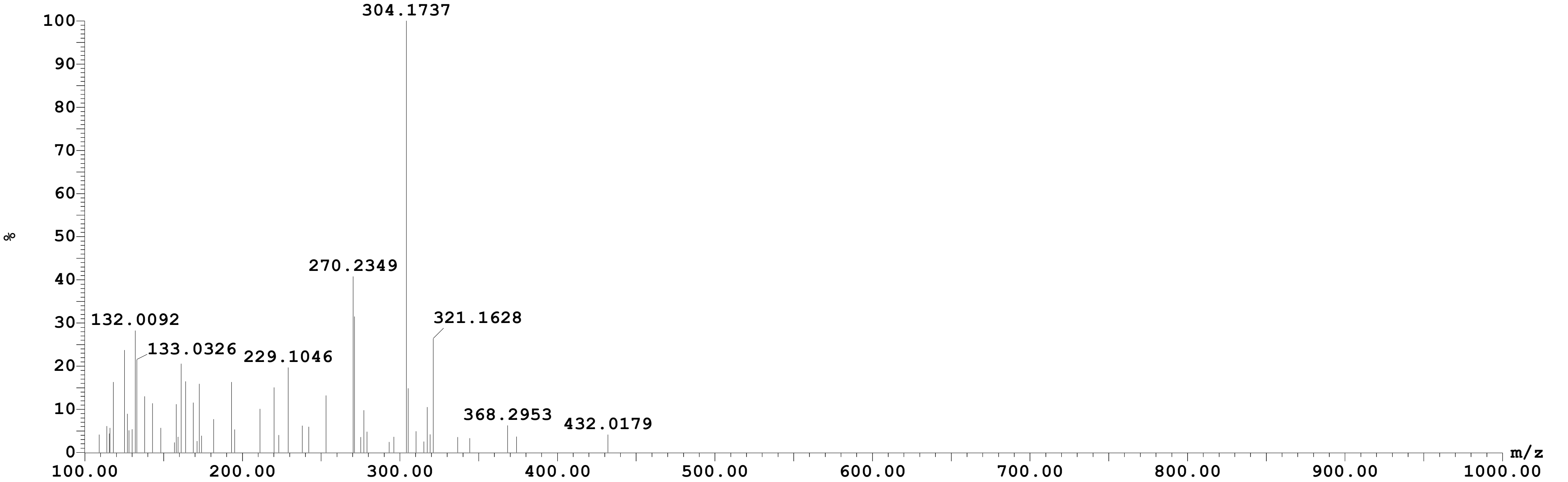

Openlynx Report -

Sample: 122
File:E2479R
Description:Eth.AC (2)

Vial:1:A,2
Date:06-Nov-2024

ID:
Time:23:37:05

Printed: Sun Nov 24 12:56:41 2024

Peak ID	Time	Error PPM
25	1.72	

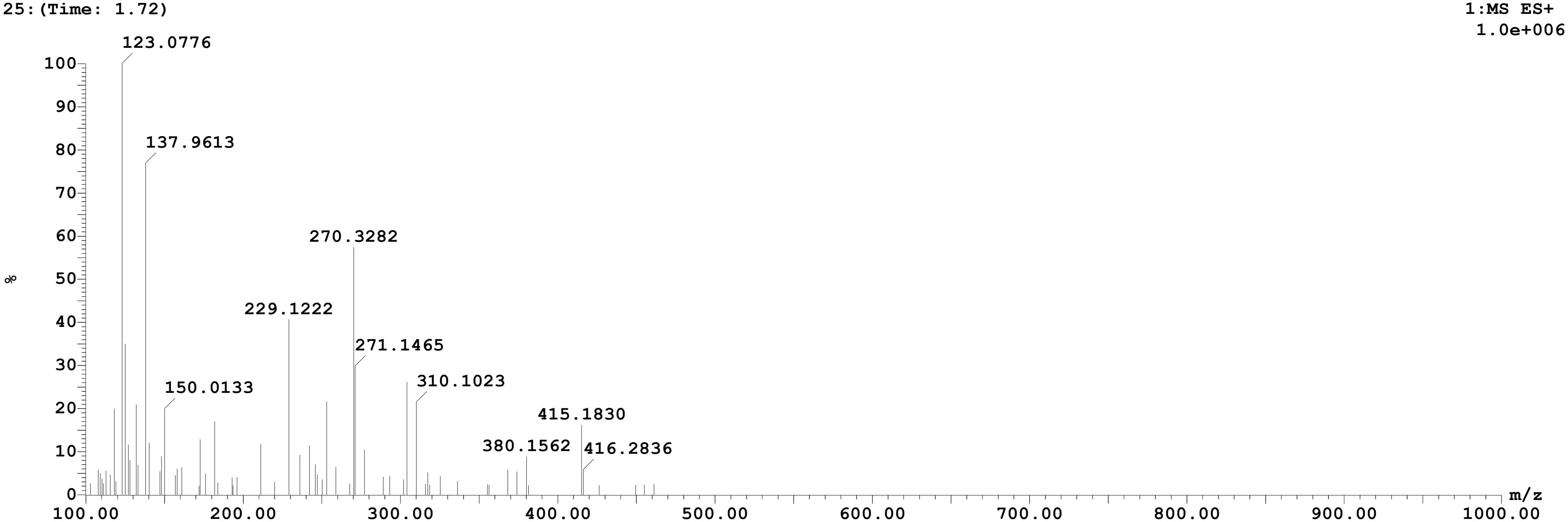

Openlynx Report -

Sample: 122
Vial:1:A,2
ID:
File:E2479R
Date:06-Nov-2024
Time:23:37:05
Description:Eth.AC (2)

Printed: Sun Nov 24 12:56:41 2024

Peak ID	Time	Error PPM
26	1.76	

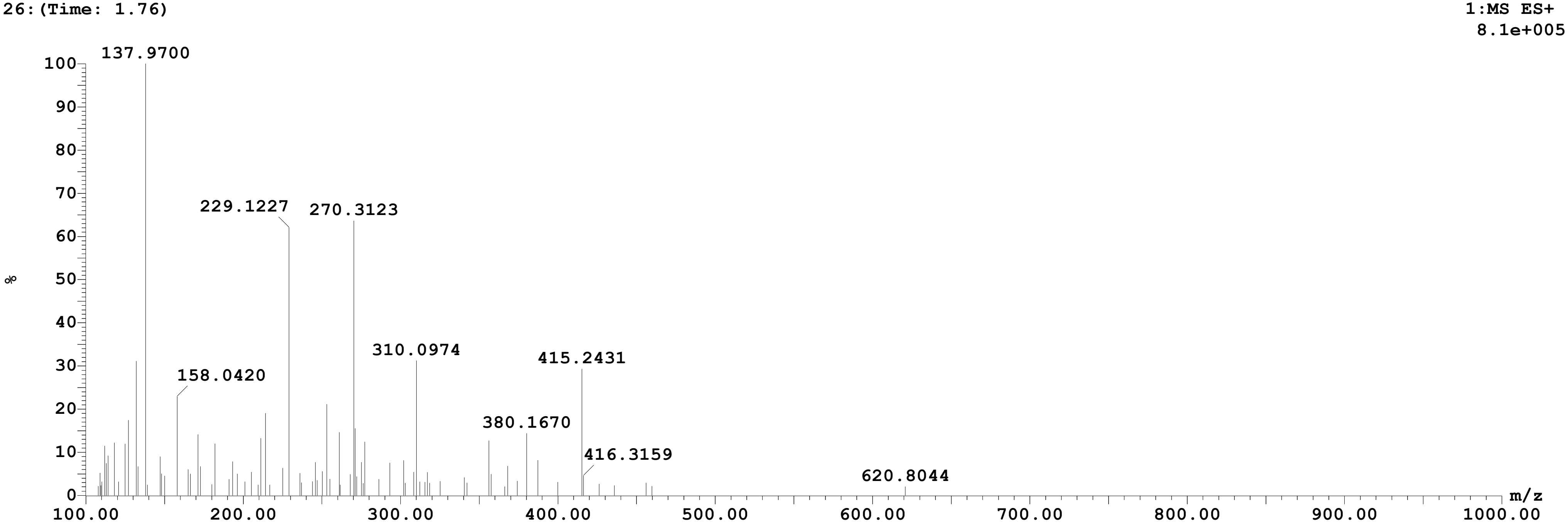

Center for DRUG DISCOVERY RESEARCH and DEVELOPMENT
Openlynx Report -

Sample: 122 Vial:1:A,2 ID:
File:E2479R Date:06-Nov-2024 Time:23:37:05
Description:Eth.AC (2)

Printed: Sun Nov 24 12:56:41 2024

Peak ID	Time	Error PPM
27	1.80	

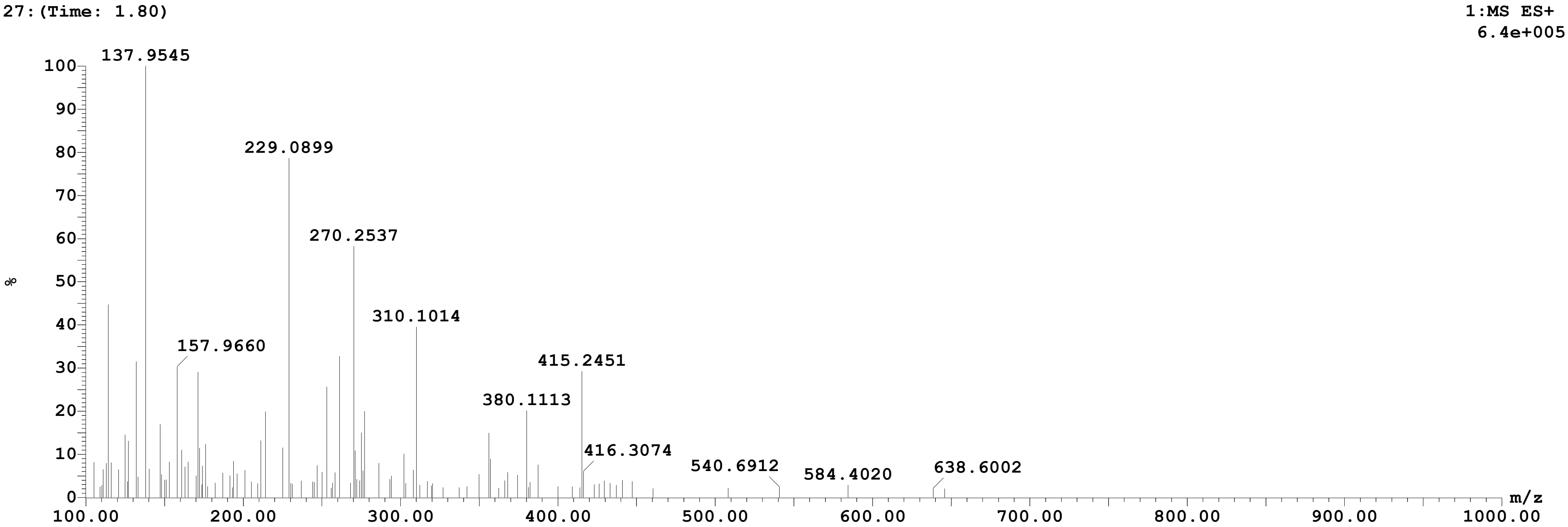

Center for DRUG DISCOVERY RESEARCH and DEVELOPMENT
Openlynx Report -

Sample: 122 | Vial:1:A,2 | ID:
File:E2479R | Date:06-Nov-2024 | Time:23:37:05
Description:Eth.AC (2)

Printed: Sun Nov 24 12:56:41 2024

Peak ID	Time	Error PPM
28	1.85	

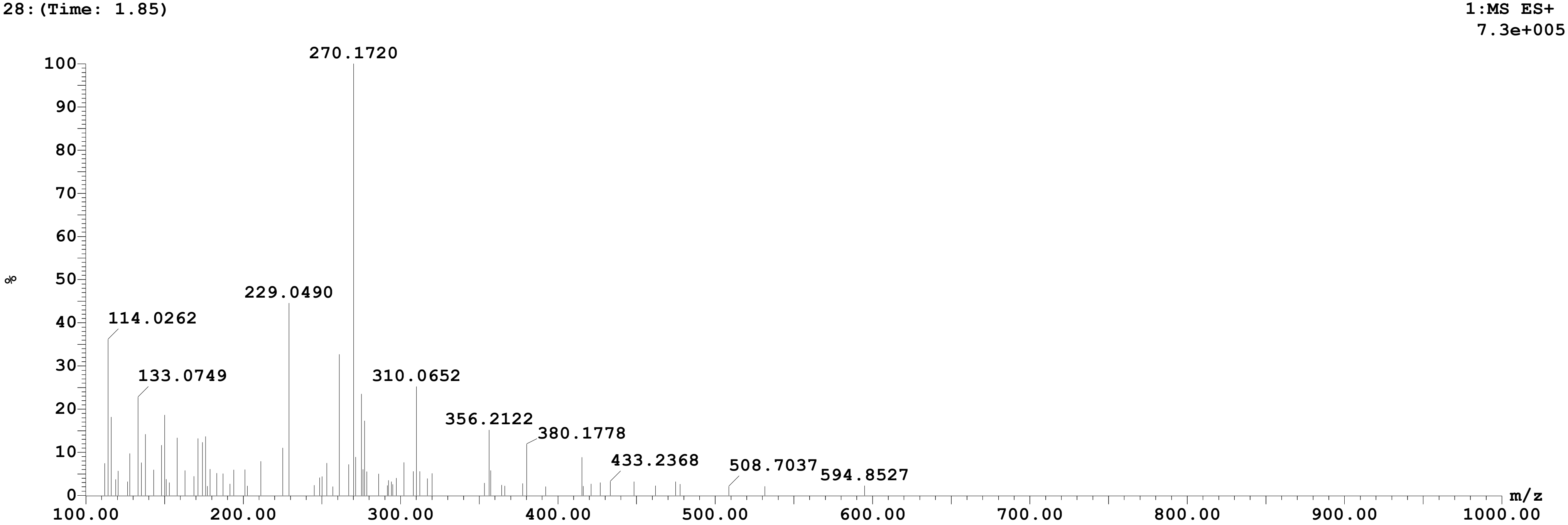

Center for DRUG DISCOVERY RESEARCH and DEVELOPMENT
Openlynx Report -

Sample: 122 Vial:1:A,2 ID:
File:E2479R Date:06-Nov-2024 Time:23:37:05
Description:Eth.AC (2)

Printed: Sun Nov 24 12:56:41 2024

Peak ID	Time	Error PPM
29	1.89	

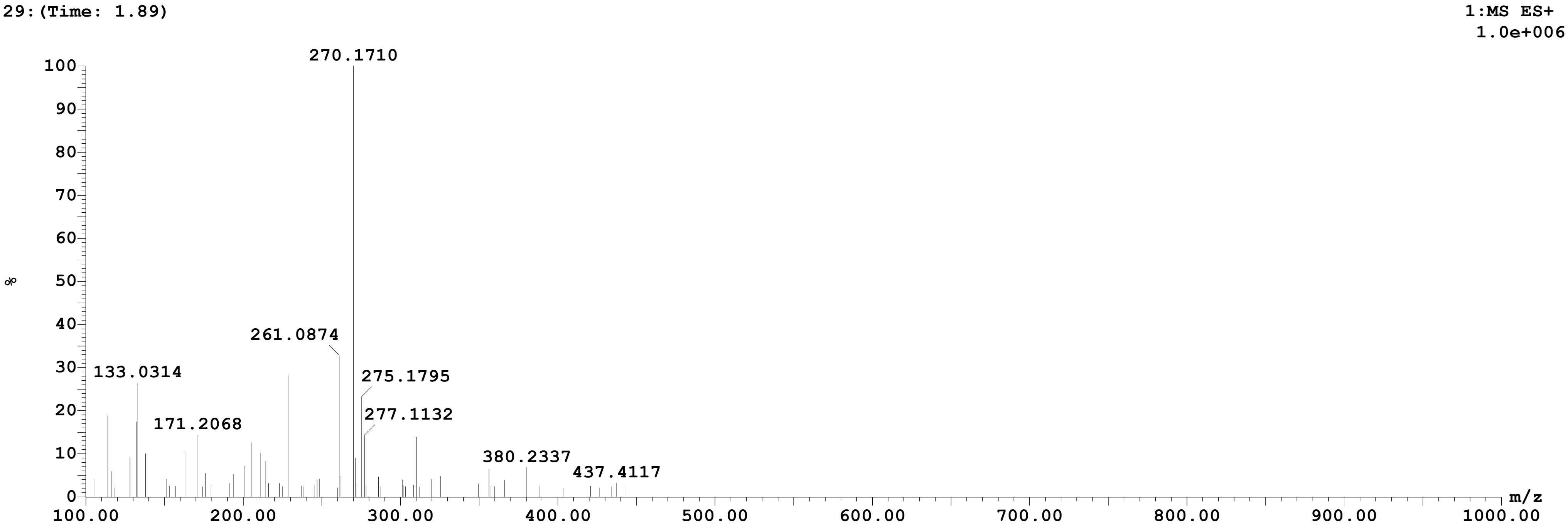

Sample: 122 Vial:1:A,2 ID:
File:E2479R Date:06-Nov-2024 Time:23:37:05
Description:Eth.AC (2)

Printed: Sun Nov 24 12:56:41 2024

Peak ID	Time	Error PPM
30	1.95	

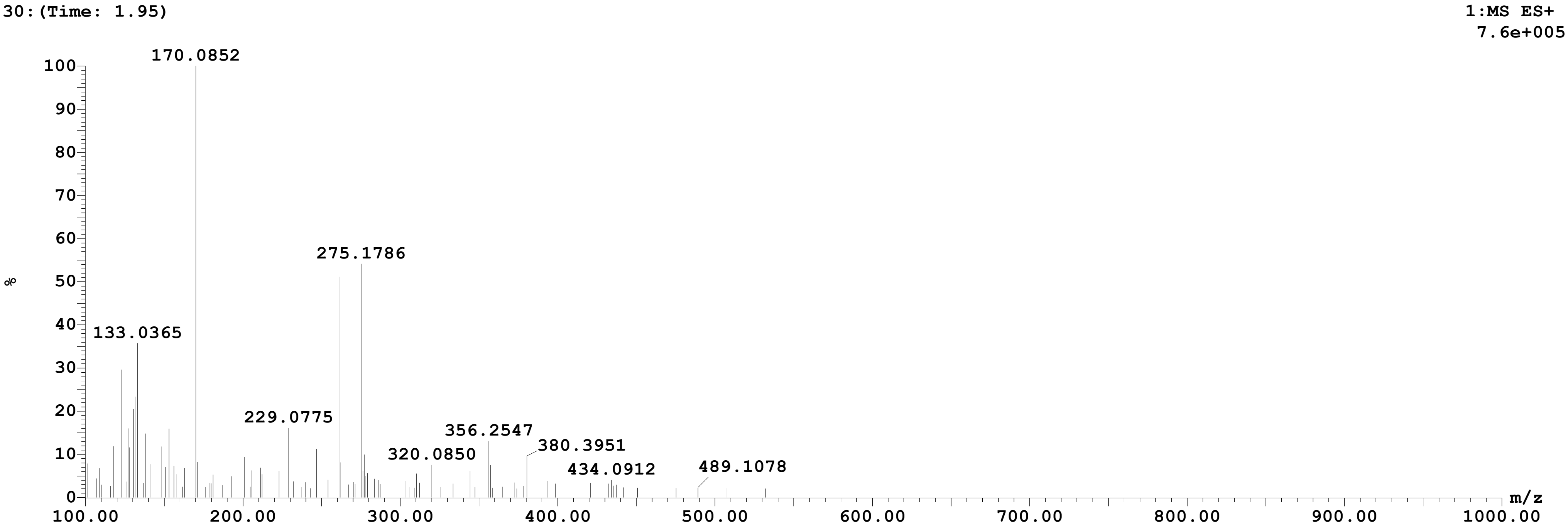

Openlynx Report -

Sample: 122 Vial:1:A,2 ID:
File:E2479R Date:06-Nov-2024 Time:23:37:05
Description:Eth.AC (2)

Printed: Sun Nov 24 12:56:41 2024

Peak ID	Time	Error PPM
31	1.98	

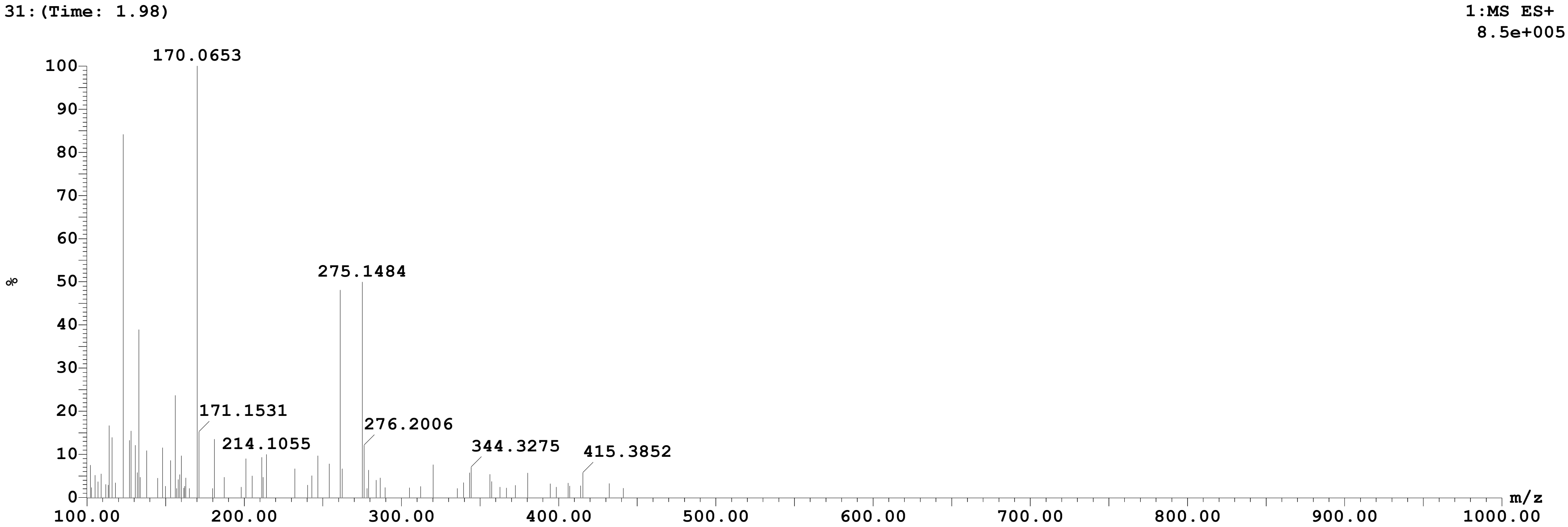

Openlynx Report -

Sample: 122
File:E2479R
Description:Eth.AC (2)

Vial:1:A,2
Date:06-Nov-2024

ID:
Time:23:37:05

Printed: Sun Nov 24 12:56:41 2024

Peak ID	Time	Error PPM
32	2.01	

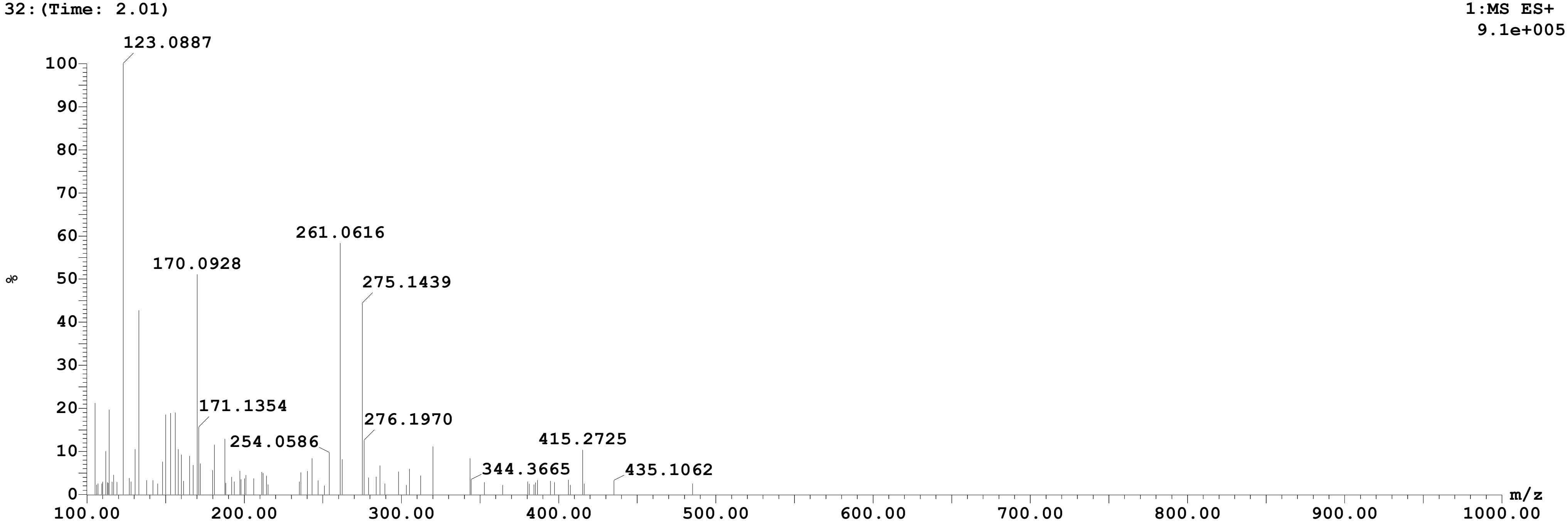

Center for DRUG DISCOVERY RESEARCH and DEVELOPMENT
Openlynx Report -

Sample: 122 | Vial:1:A,2 | ID:
File:E2479R | Date:06-Nov-2024 | Time:23:37:05
Description:Eth.AC (2)

Printed: Sun Nov 24 12:56:41 2024

Peak ID	Time	Error PPM
33	2.07	

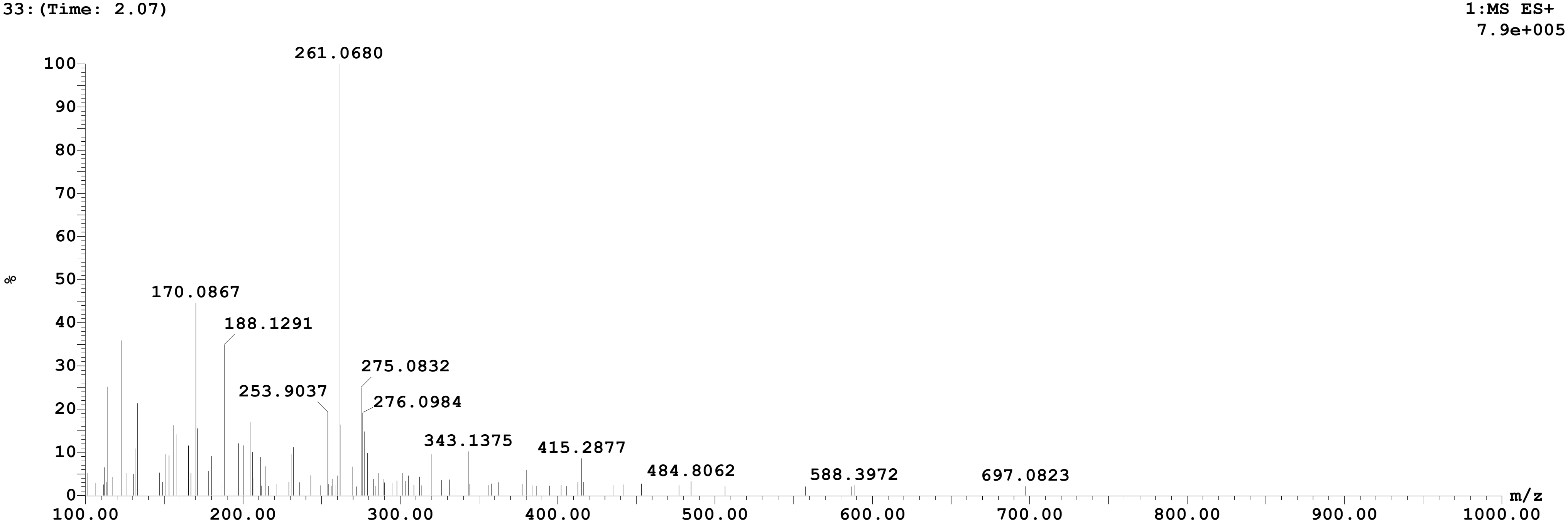

Center for DRUG DISCOVERY RESEARCH and DEVELOPMENT
Openlynx Report -

Sample: 122 Vial:1:A,2 ID:
File:E2479R Date:06-Nov-2024 Time:23:37:05
Description:Eth.AC (2)

Printed: Sun Nov 24 12:56:41 2024

Peak ID	Time	Error PPM
34	2.13	

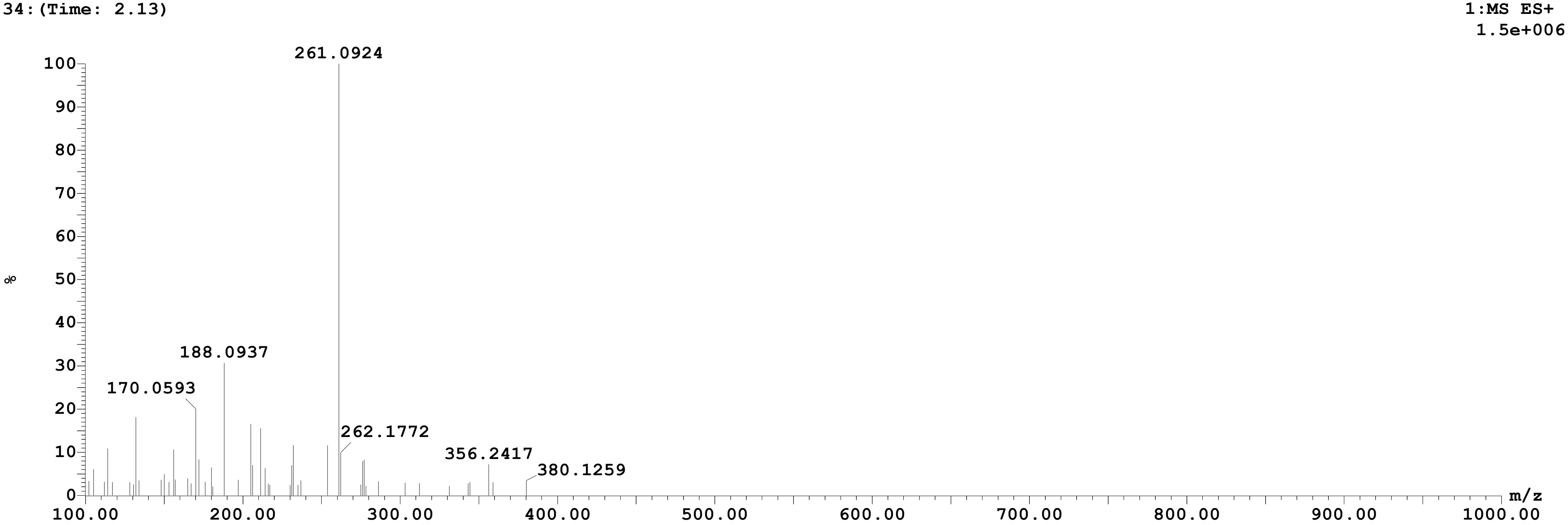

Openlynx Report -

Sample: 122 Vial:1:A,2 ID:
File:E2479R Date:06-Nov-2024 Time:23:37:05
Description:Eth.AC (2)

Printed: Sun Nov 24 12:56:41 2024

Peak ID	Time	Error PPM
35	2.16	

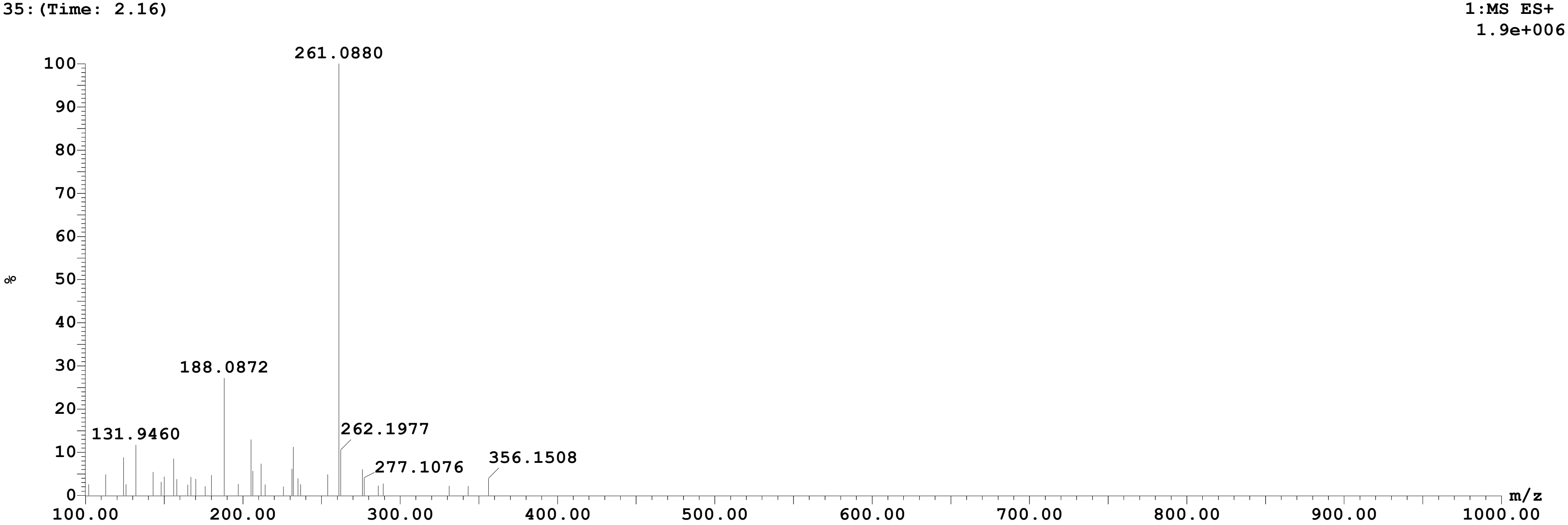

Center for DRUG DISCOVERY RESEARCH and DEVELOPMENT
Openlynx Report -

Sample: 122 | Vial:1:A,2 | ID:
File:E2479R | Date:06-Nov-2024 | Time:23:37:05
Description:Eth.AC (2)

Printed: Sun Nov 24 12:56:41 2024

Peak ID	Time	Error PPM
36	2.20	

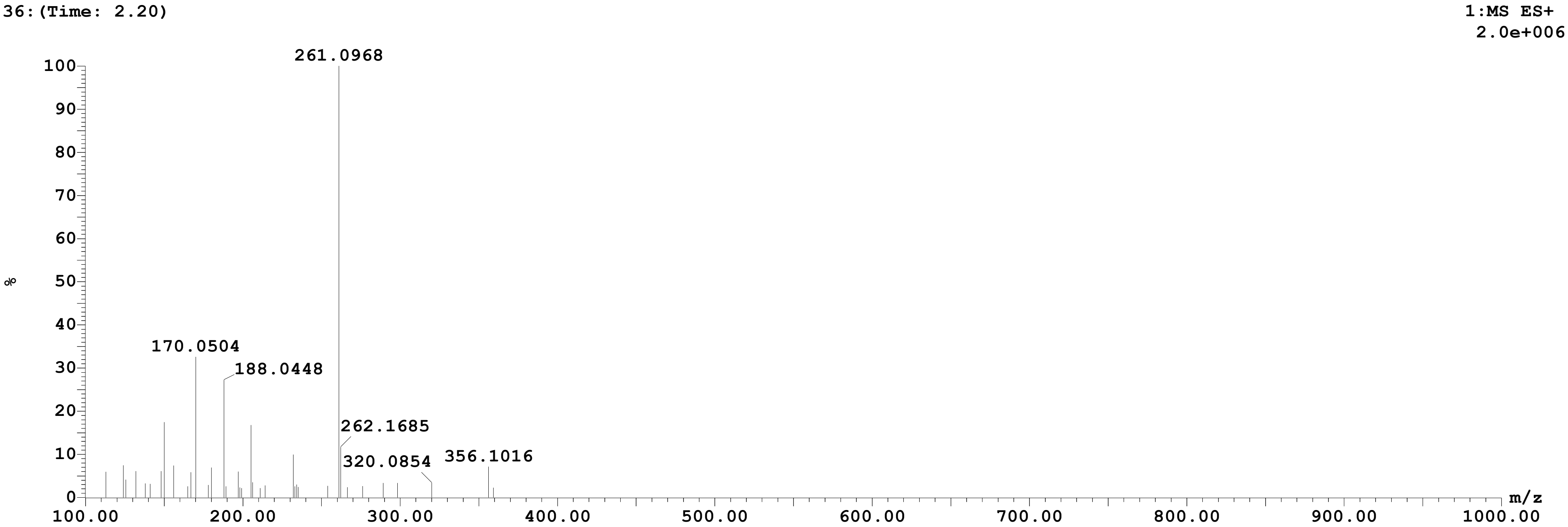

Center for DRUG DISCOVERY RESEARCH and DEVELOPMENT
Openlynx Report -

Sample: 122 Vial:1:A,2 ID:
File:E2479R Date:06-Nov-2024 Time:23:37:05
Description:Eth.AC (2)

Printed: Sun Nov 24 12:56:41 2024

Peak ID	Time	Error PPM
37	2.23	

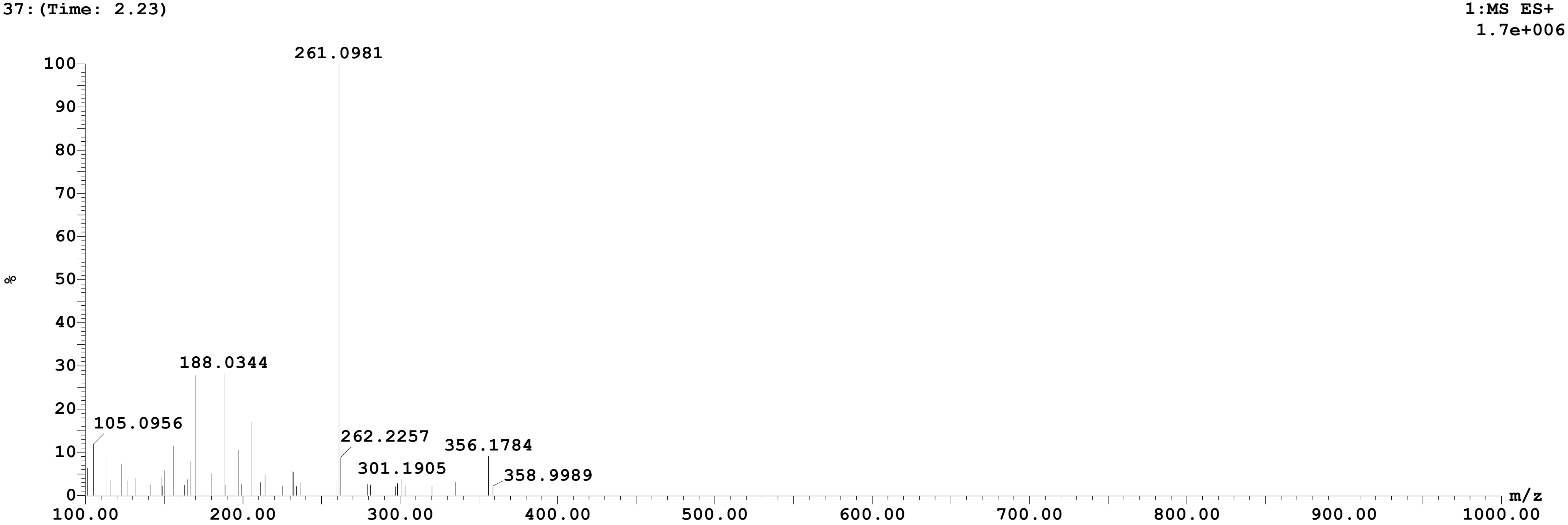

Center for DRUG DISCOVERY RESEARCH and DEVELOPMENT
Openlynx Report -

Sample: 122 | Vial:1:A,2 | ID:
File:E2479R | Date:06-Nov-2024 | Time:23:37:05
Description:Eth.AC (2)

Printed: Sun Nov 24 12:56:41 2024

Peak ID	Time	Error PPM
38	2.26	

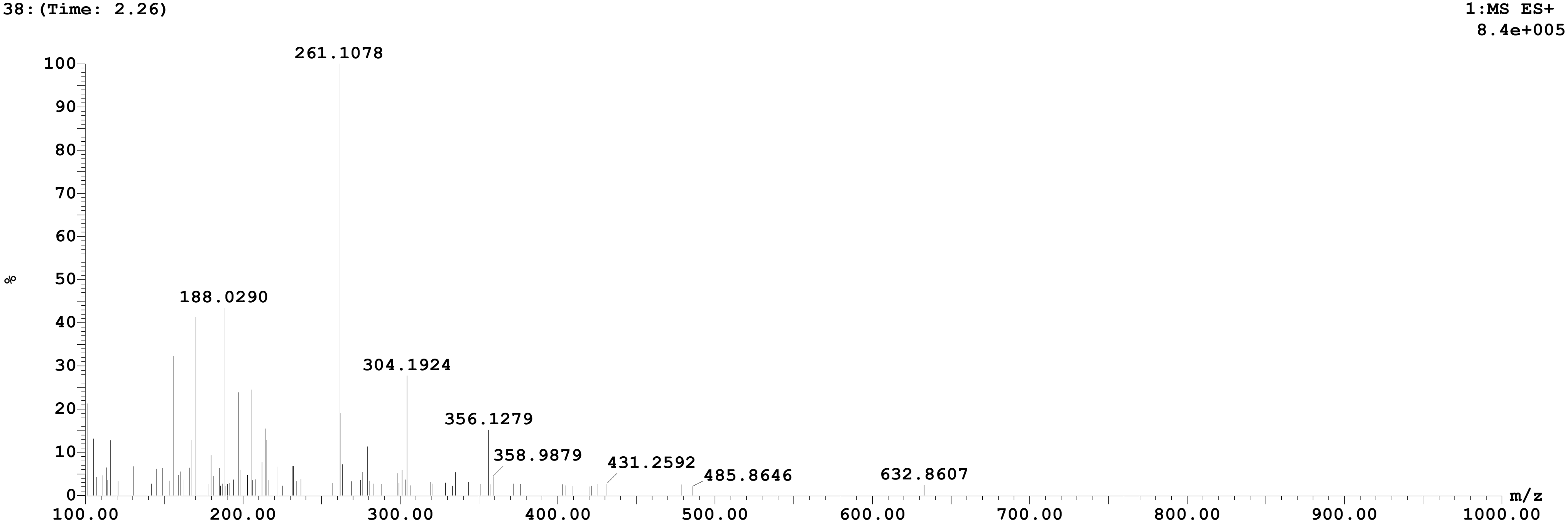

Center for DRUG DISCOVERY RESEARCH and DEVELOPMENT

Openlynx Report -

Sample: 122 Vial:1:A,2 ID:
File:E2479R Date:06-Nov-2024 Time:23:37:05
Description:Eth.AC (2)

Printed: Sun Nov 24 12:56:41 2024

Peak ID	Time	Error PPM
39	2.32	

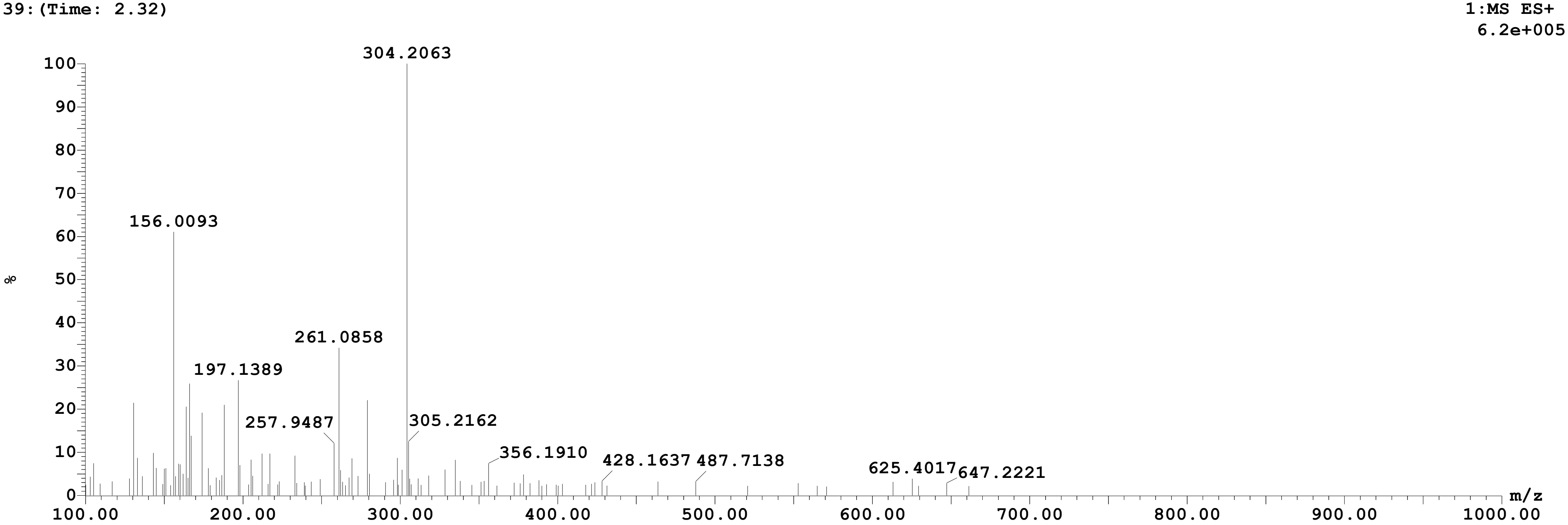

Center for DRUG DISCOVERY RESEARCH and DEVELOPMENT
Openlynx Report -

Sample: 122	Vial:1:A,2	ID:
File:E2479R	Date:06-Nov-2024	Time:23:37:05
Description:Eth.AC (2)		

Printed: Sun Nov 24 12:56:41 2024

Peak ID	Time	Error PPM
40	2.35	

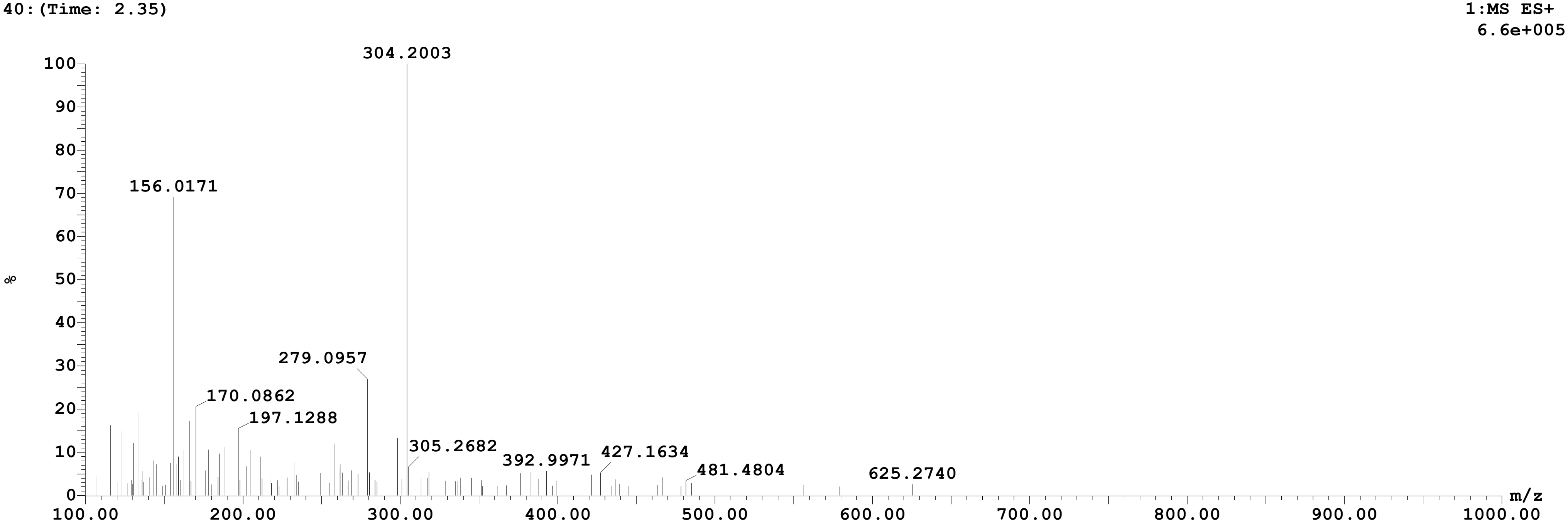

Openlynx Report -

Sample: 122 Vial:1:A,2 ID:
File:E2479R Date:06-Nov-2024 Time:23:37:05
Description:Eth.AC (2)

Printed: Sun Nov 24 12:56:41 2024

Peak ID	Time	Error PPM
41	2.38	

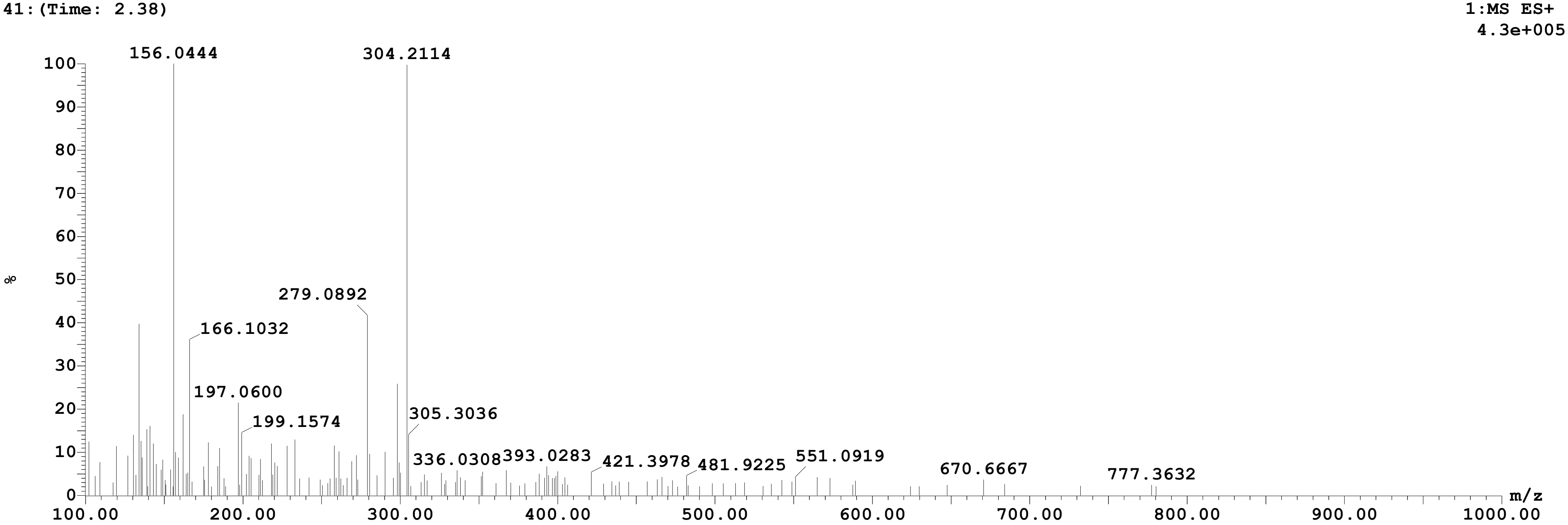

Center for DRUG DISCOVERY RESEARCH and DEVELOPMENT
Openlynx Report -

Sample: 122 Vial:1:A,2 ID:
File:E2479R Date:06-Nov-2024 Time:23:37:05
Description:Eth.AC (2)

Printed: Sun Nov 24 12:56:41 2024

Peak ID	Time	Error PPM
42	2.41	

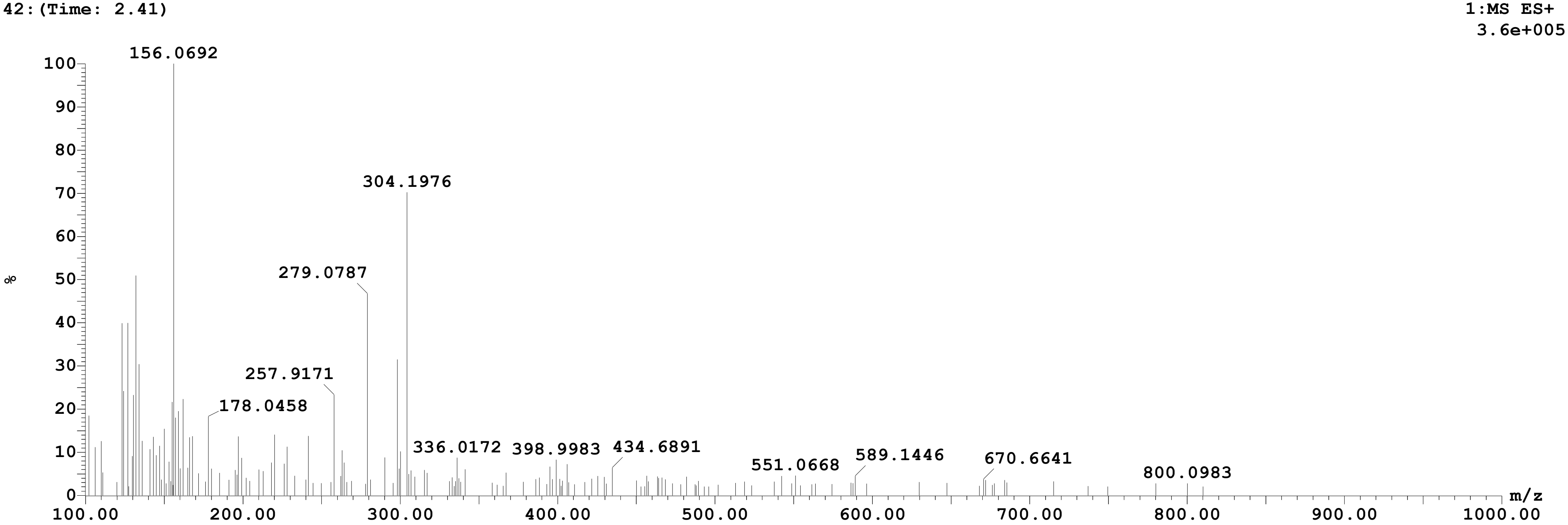

Openlynx Report -

Sample: 122 | Vial:1:A,2 | ID:
File:E2479R | Date:06-Nov-2024 | Time:23:37:05
Description:Eth.AC (2)

Printed: Sun Nov 24 12:56:41 2024

Peak ID	Time	Error PPM
43	2.48	

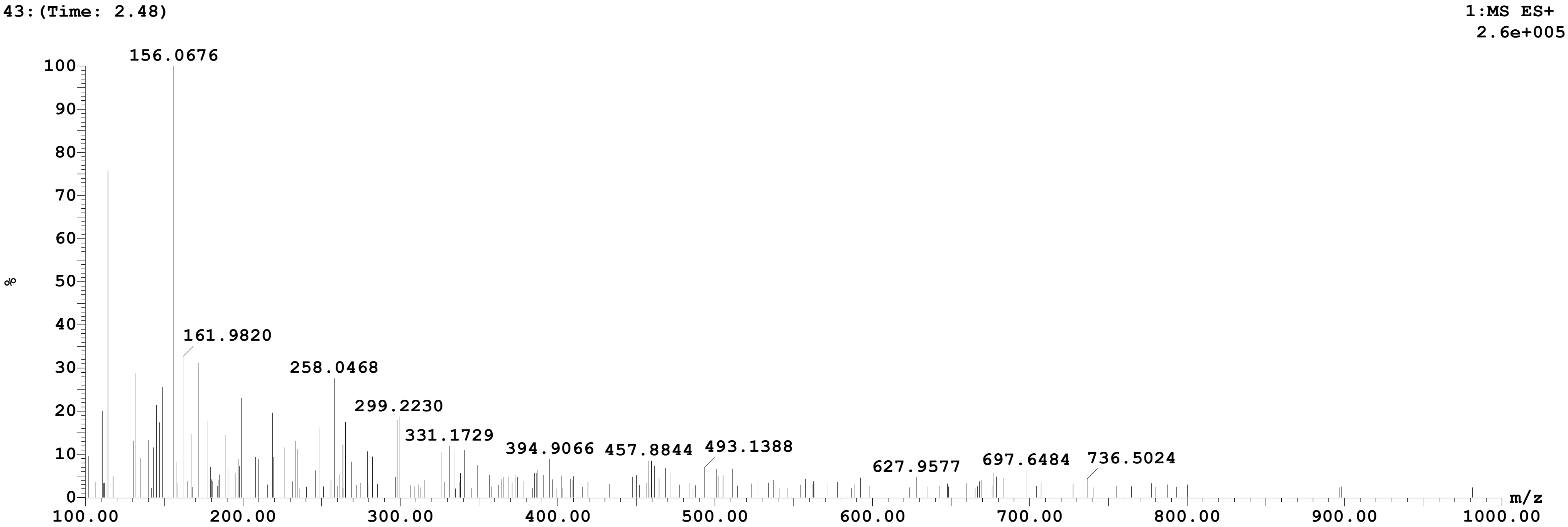

Openlynx Report -

Sample: 122 Vial:1:A,2 ID:
File:E2479R Date:06-Nov-2024 Time:23:37:05
Description:Eth.AC (2)

Printed: Sun Nov 24 12:56:41 2024

Peak ID	Time	Error PPM
44	2.57	

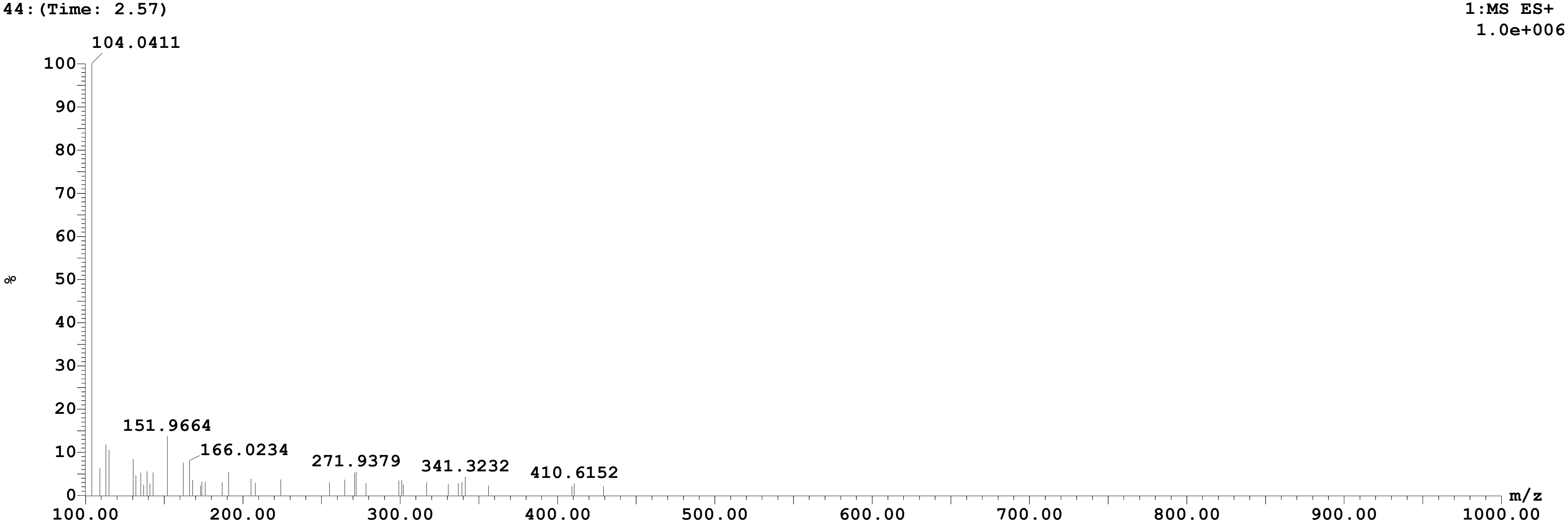

Center for DRUG DISCOVERY RESEARCH and DEVELOPMENT
Openlynx Report -

Sample: 122 | Vial:1:A,2 | ID:
File:E2479R | Date:06-Nov-2024 | Time:23:37:05
Description:Eth.AC (2)

Printed: Sun Nov 24 12:56:41 2024

Peak ID	Time	Error PPM
45	2.60	

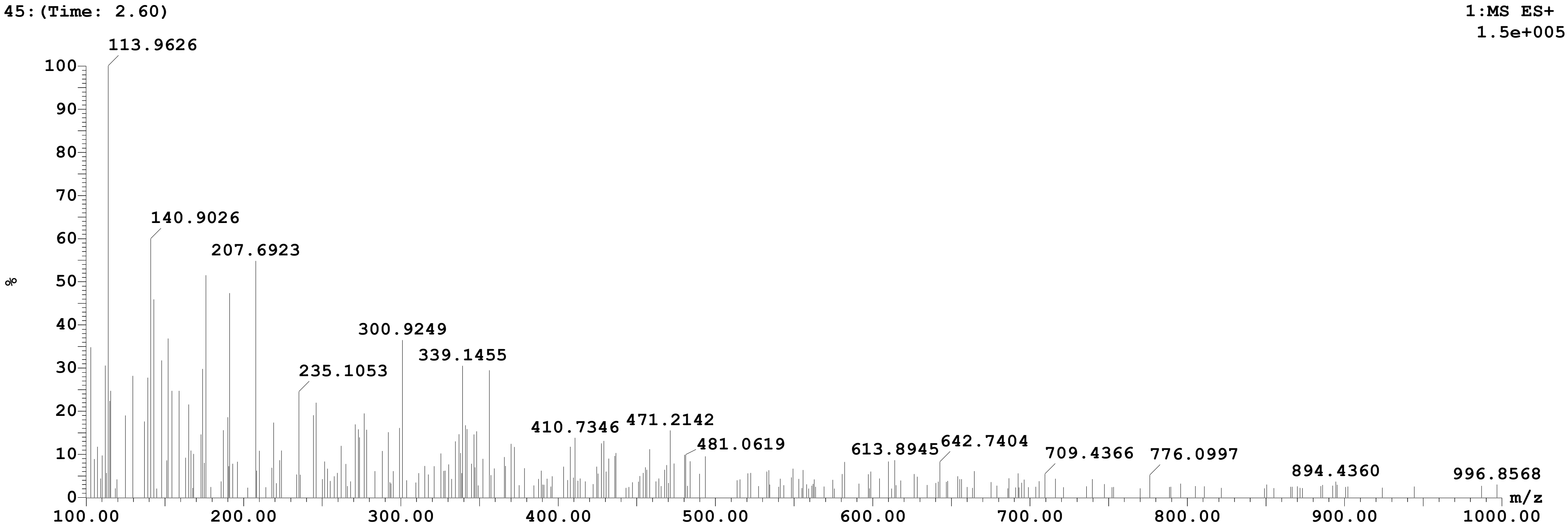

Center for DRUG DISCOVERY RESEARCH and DEVELOPMENT
Openlynx Report -

Sample: 122 | Vial:1:A,2 | ID:
File:E2479R | Date:06-Nov-2024 | Time:23:37:05
Description:Eth.AC (2)

Printed: Sun Nov 24 12:56:41 2024

Peak ID	Time	Error PPM
46	2.64	

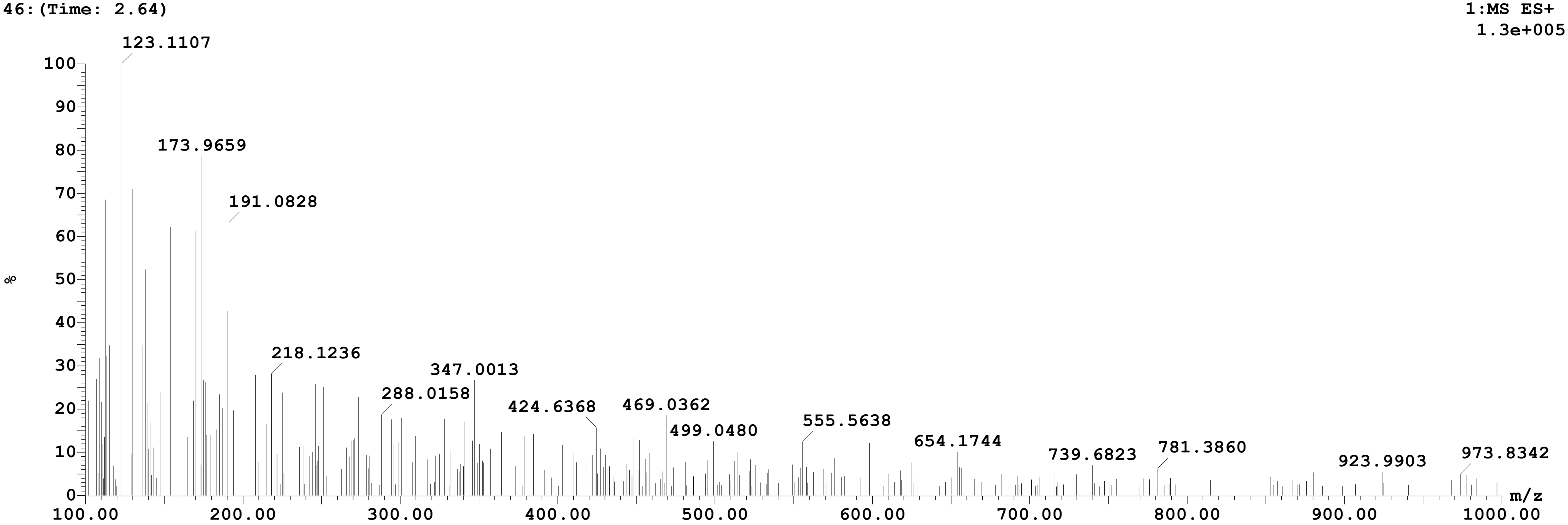

Center for DRUG DISCOVERY RESEARCH and DEVELOPMENT
Openlynx Report -

Sample: 122 | Vial:1:A,2 | ID:
File:E2479R | Date:06-Nov-2024 | Time:23:37:05
Description:Eth.AC (2)

Printed: Sun Nov 24 12:56:41 2024

Peak ID	Time	Error PPM
47	2.70	

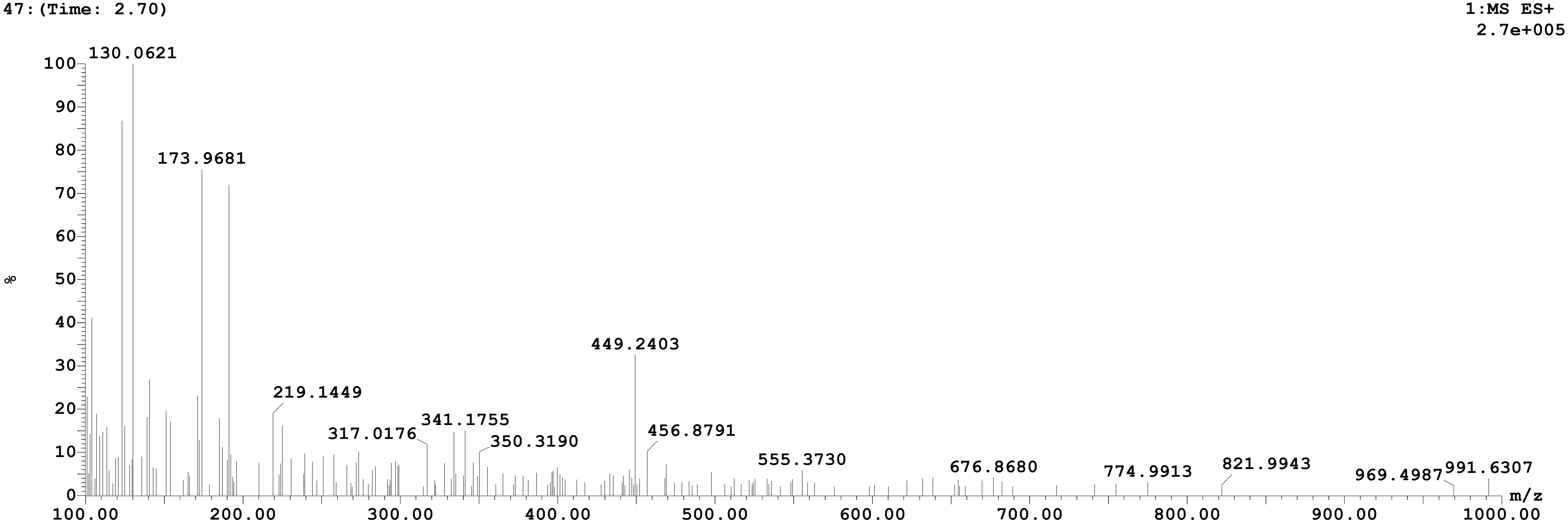

Center for DRUG DISCOVERY RESEARCH and DEVELOPMENT
Openlynx Report -

Sample: 122 | Vial:1:A,2 | ID:
File:E2479R | Date:06-Nov-2024 | Time:23:37:05
Description:Eth.AC (2)

Printed: Sun Nov 24 12:56:41 2024

Peak ID	Time	Error PPM
48	2.76	

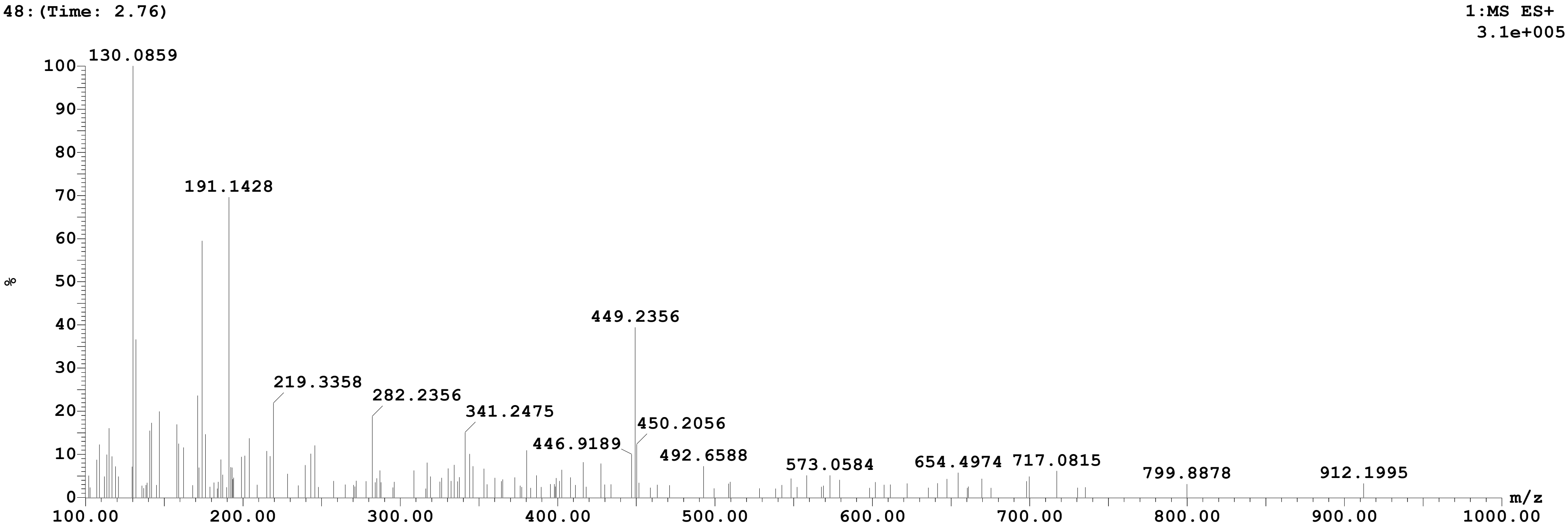

Center for DRUG DISCOVERY RESEARCH and DEVELOPMENT
Openlynx Report -

Sample: 122 | Vial:1:A,2 | ID:
File:E2479R | Date:06-Nov-2024 | Time:23:37:05
Description:Eth.AC (2)

Printed: Sun Nov 24 12:56:41 2024

Peak ID	Time	Error PPM
49	2.79	

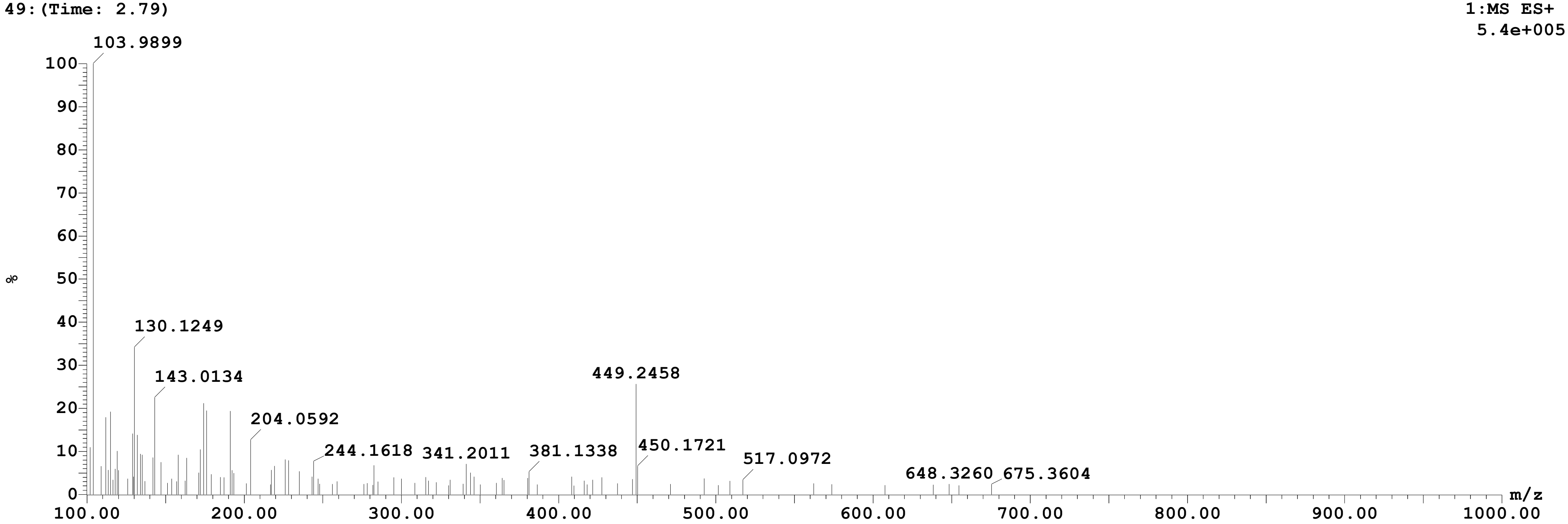

Center for DRUG DISCOVERY RESEARCH and DEVELOPMENT
Openlynx Report -

Sample: 122
File:E2479R
Description:Eth.AC (2)

Vial:1:A,2
Date:06-Nov-2024

ID:
Time:23:37:05

Printed: Sun Nov 24 12:56:41 2024

Peak ID	Time	Error PPM
50	2.86	

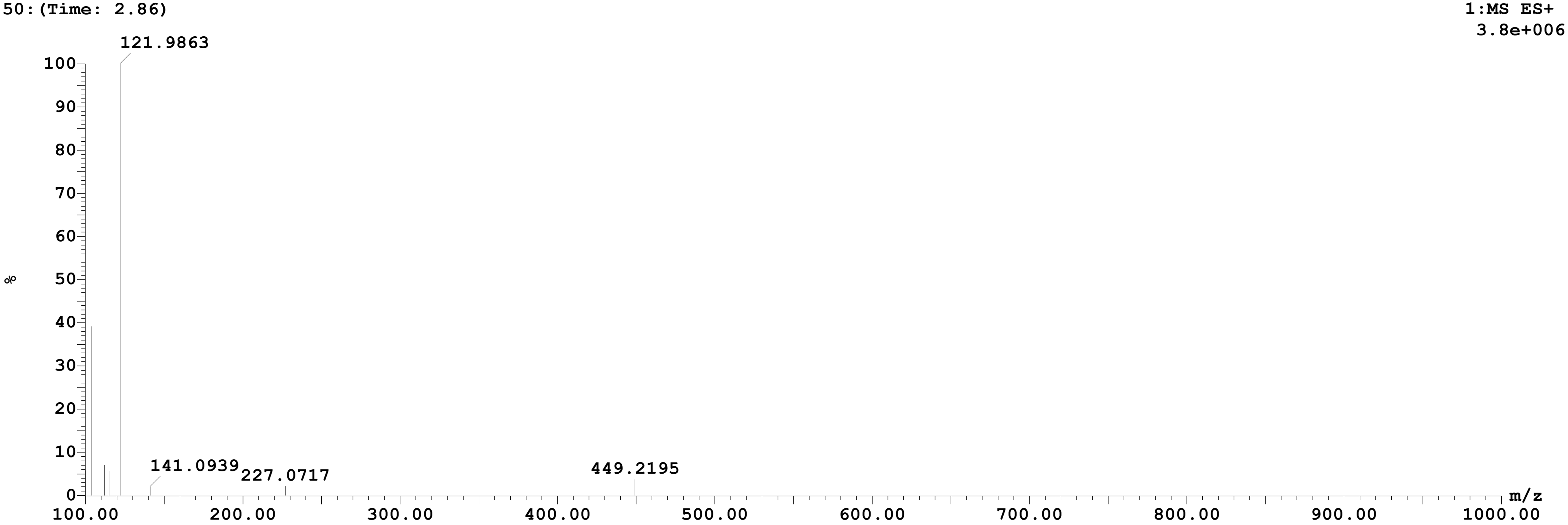

Center for DRUG DISCOVERY RESEARCH and DEVELOPMENT
Openlynx Report -

Sample: 122
File:E2479R
Description:Eth.AC (2)

Vial:1:A,2
Date:06-Nov-2024

ID:
Time:23:37:05

Printed: Sun Nov 24 12:56:41 2024

Peak ID	Time	Error PPM
51	2.89	

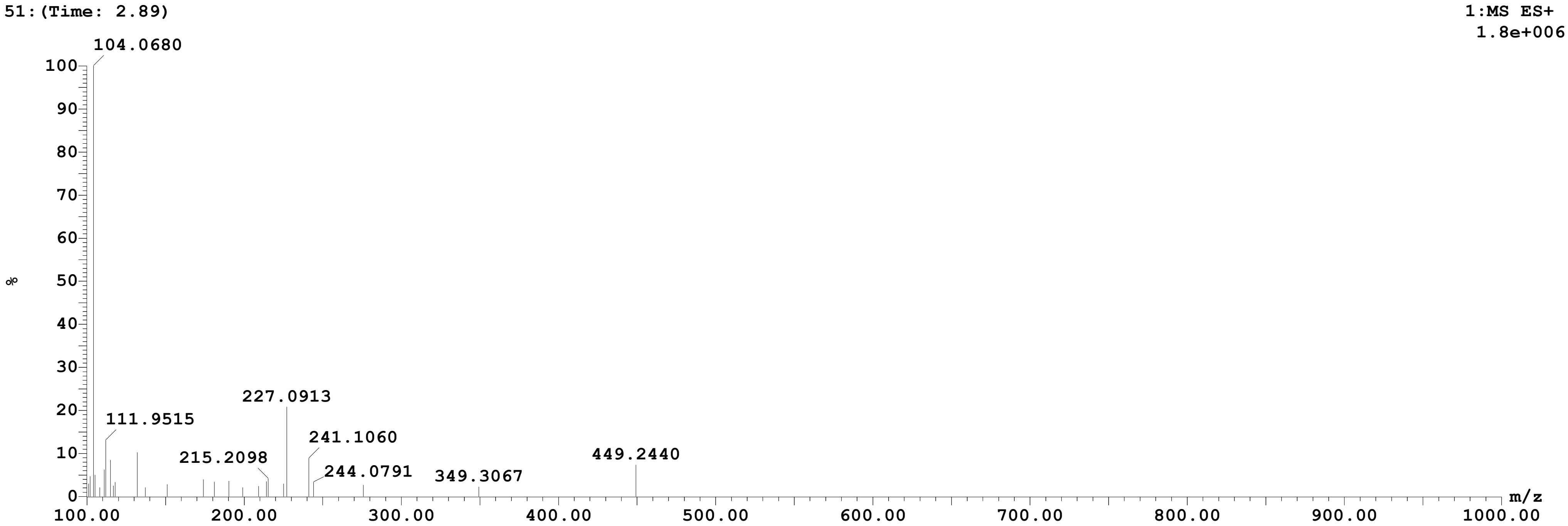

Openlynx Report -

Sample: 122 Vial:1:A,2 ID:
File:E2479R Date:06-Nov-2024 Time:23:37:05
Description:Eth.AC (2)

Printed: Sun Nov 24 12:56:41 2024

Peak ID	Time	Error PPM
52	2.96	

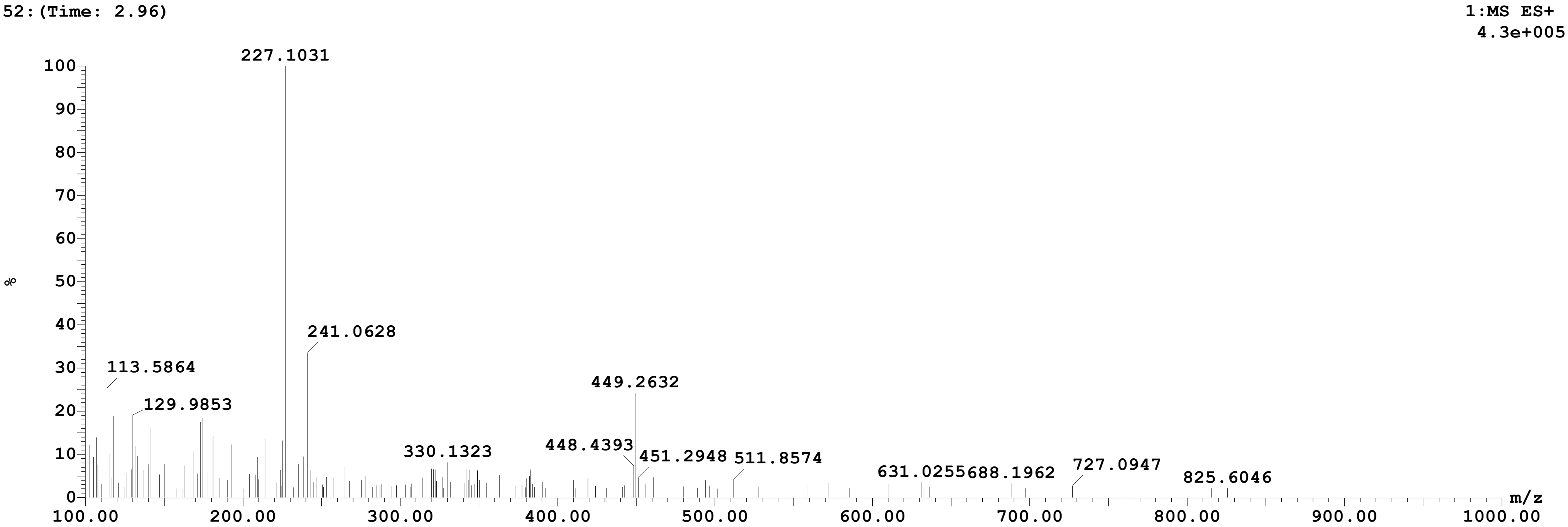

Center for DRUG DISCOVERY RESEARCH and DEVELOPMENT
Openlynx Report -

Sample: 122 | Vial:1:A,2 | ID:
File:E2479R | Date:06-Nov-2024 | Time:23:37:05
Description:Eth.AC (2)

Printed: Sun Nov 24 12:56:41 2024

Peak ID	Time	Error PPM
53	3.01	

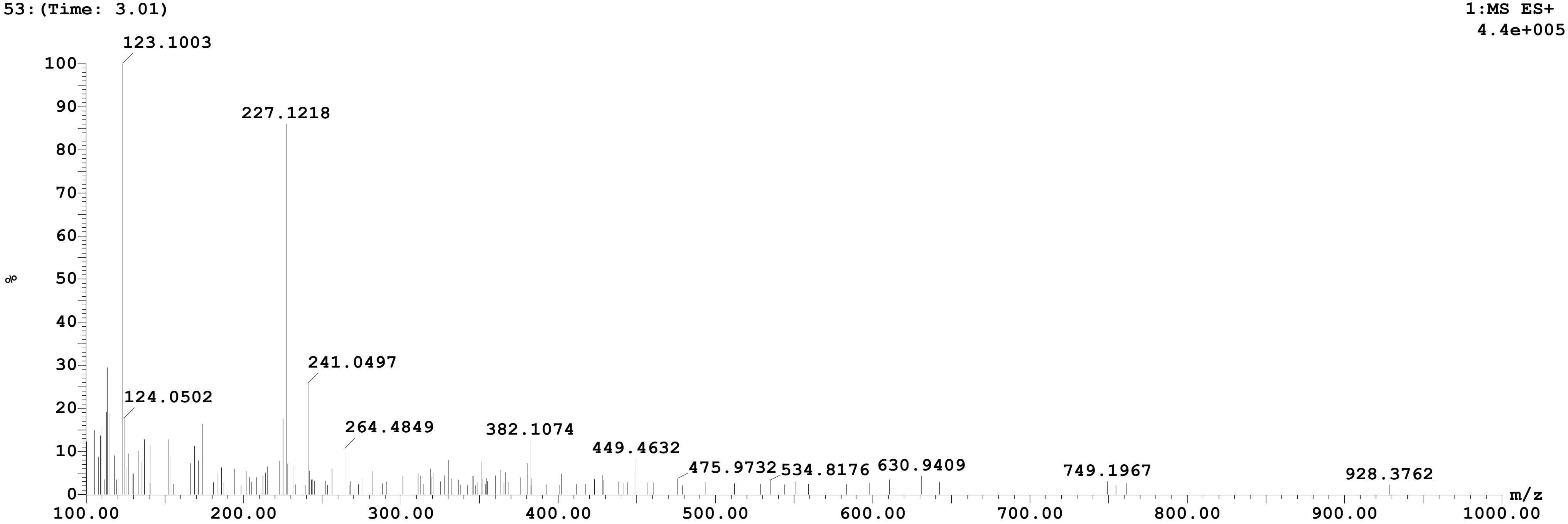

Center for DRUG DISCOVERY RESEARCH and DEVELOPMENT
Openlynx Report -

Sample: 122
File:E2479R
Description:Eth.AC (2)

Vial:1:A,2
Date:06-Nov-2024

ID:
Time:23:37:05

Printed: Sun Nov 24 12:56:41 2024

Peak ID	Time	Error PPM
54	3.07	

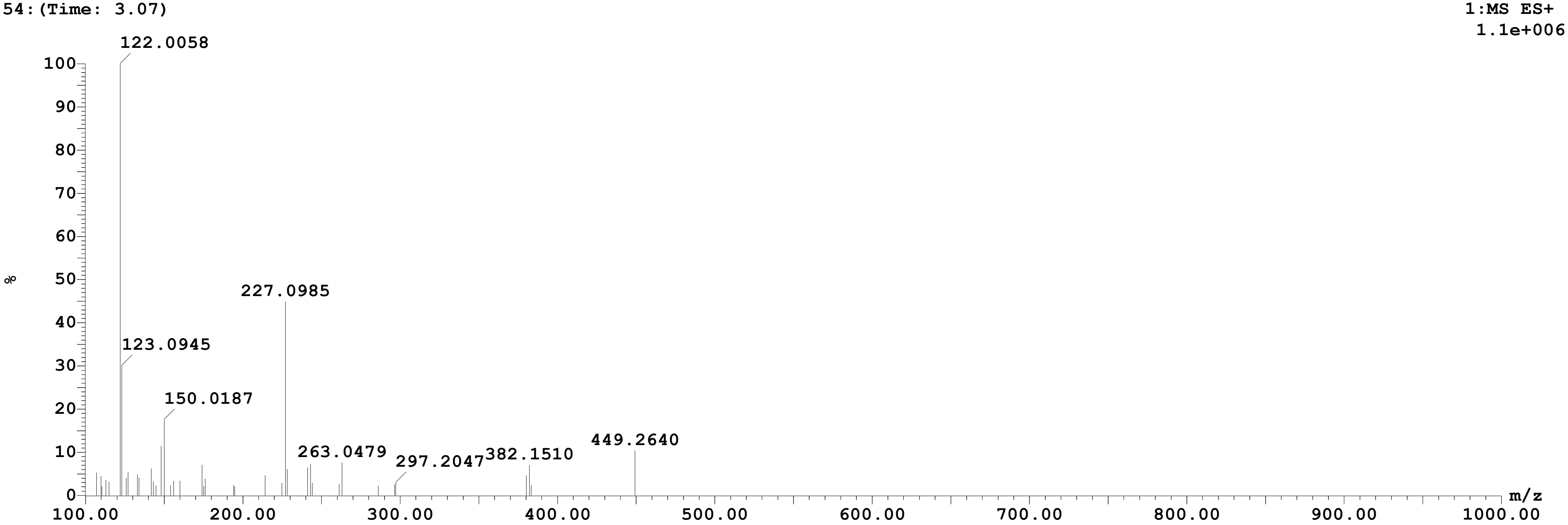

Center for DRUG DISCOVERY RESEARCH and DEVELOPMENT
Openlynx Report -

Sample: 122
File:E2479R
Description:Eth.AC (2)

Vial:1:A,2
Date:06-Nov-2024

ID:
Time:23:37:05

Printed: Sun Nov 24 12:56:41 2024

Peak ID	Time	Error PPM
55	3.11	

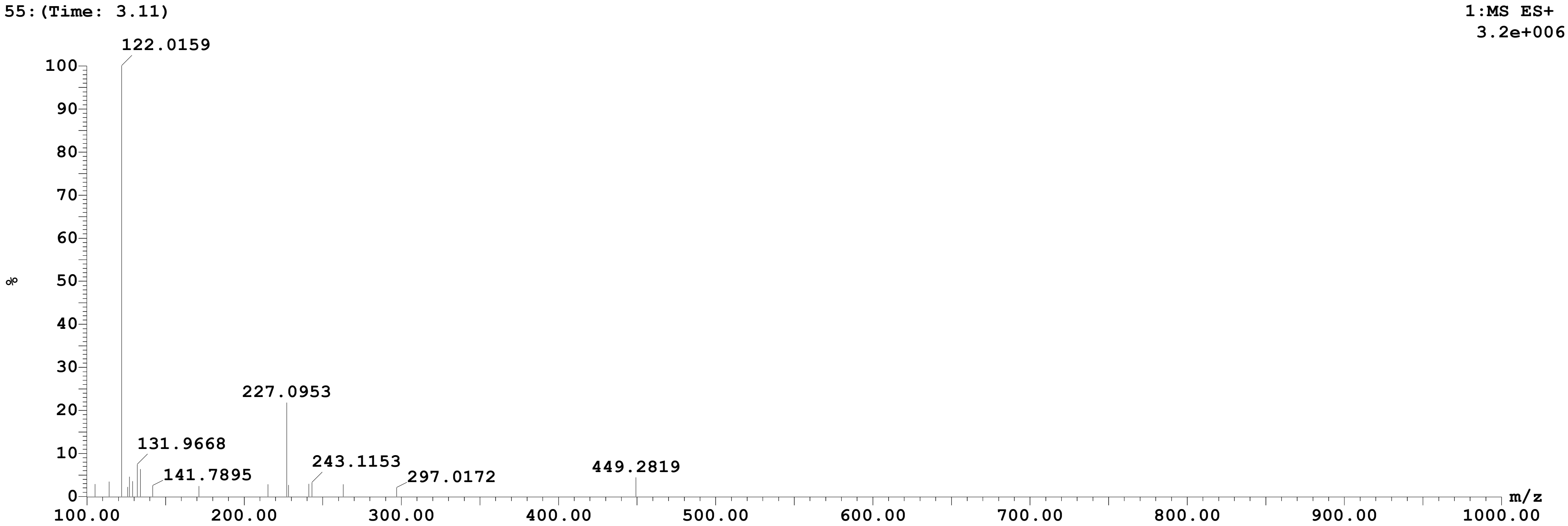

Center for DRUG DISCOVERY RESEARCH and DEVELOPMENT
Openlynx Report -

Sample: 122 | Vial:1:A,2 | ID:
File:E2479R | Date:06-Nov-2024 | Time:23:37:05
Description:Eth.AC (2)

Printed: Sun Nov 24 12:56:41 2024

Peak ID	Time	Error PPM
56	3.18	

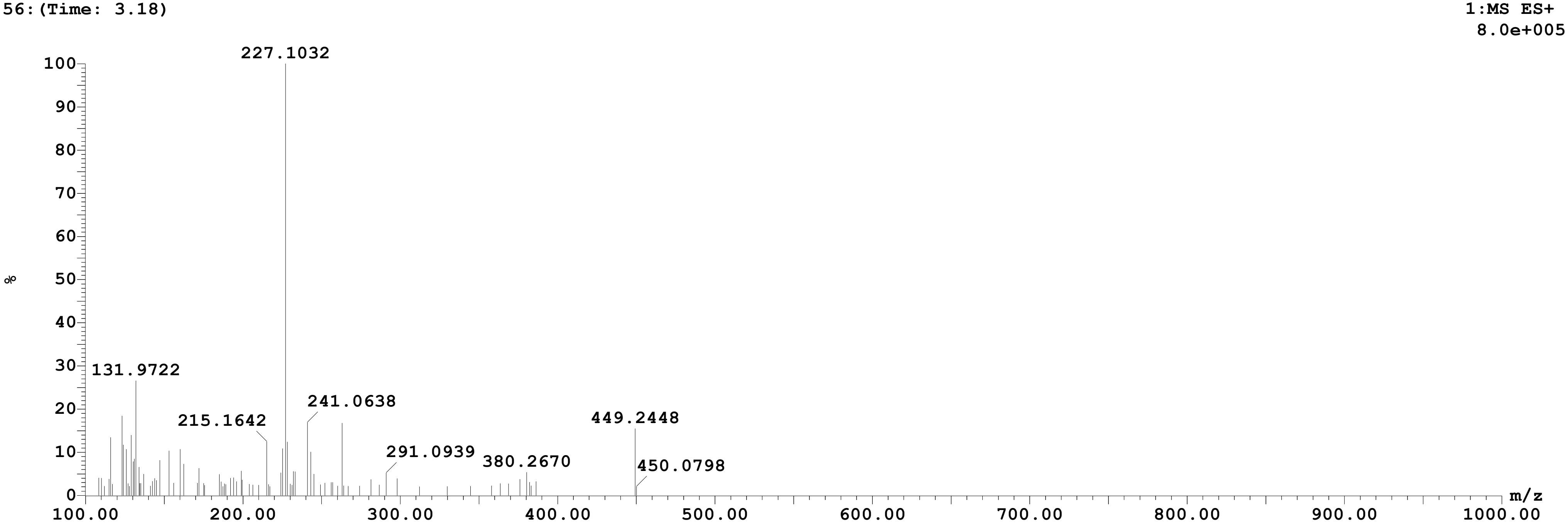

Openlynx Report -

Sample: 122 Vial:1:A,2 ID:
File:E2479R Date:06-Nov-2024 Time:23:37:05
Description:Eth.AC (2)

Printed: Sun Nov 24 12:56:41 2024

Peak ID	Time	Error PPM
57	3.30	

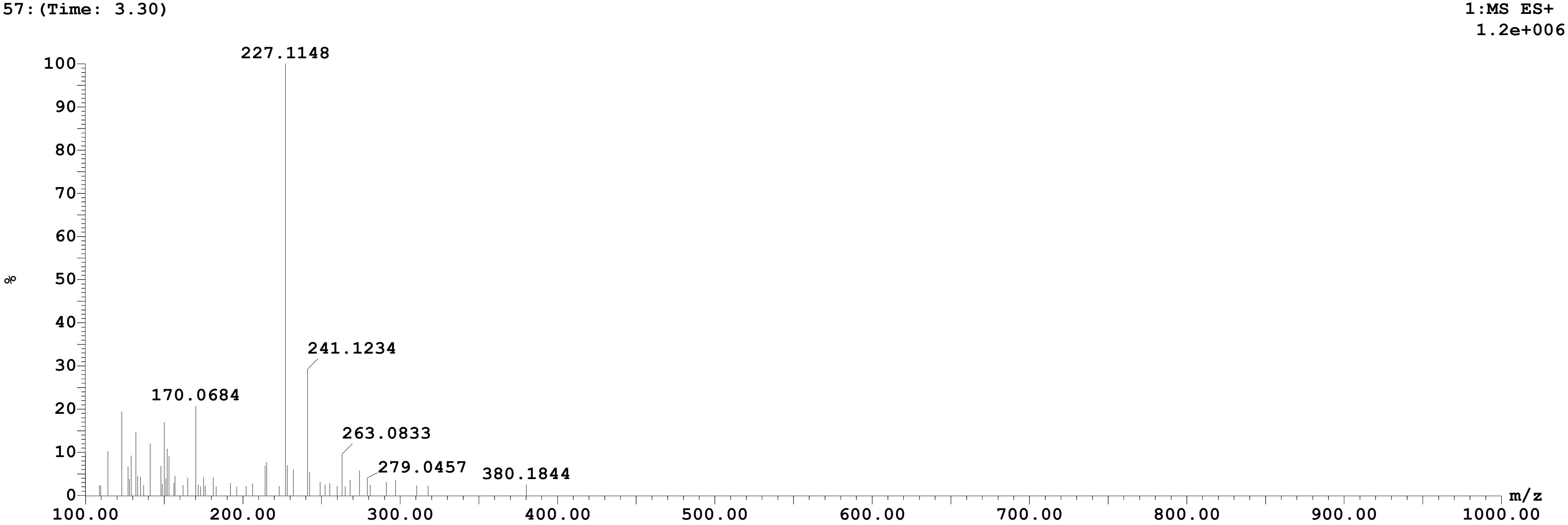

Center for DRUG DISCOVERY RESEARCH and DEVELOPMENT
Openlynx Report -

Sample: 122	Vial:1:A,2	ID:
File:E2479R	Date:06-Nov-2024	Time:23:37:05
Description:Eth.AC (2)		

Printed: Sun Nov 24 12:56:41 2024

Peak ID	Time	Error PPM
58	3.39	

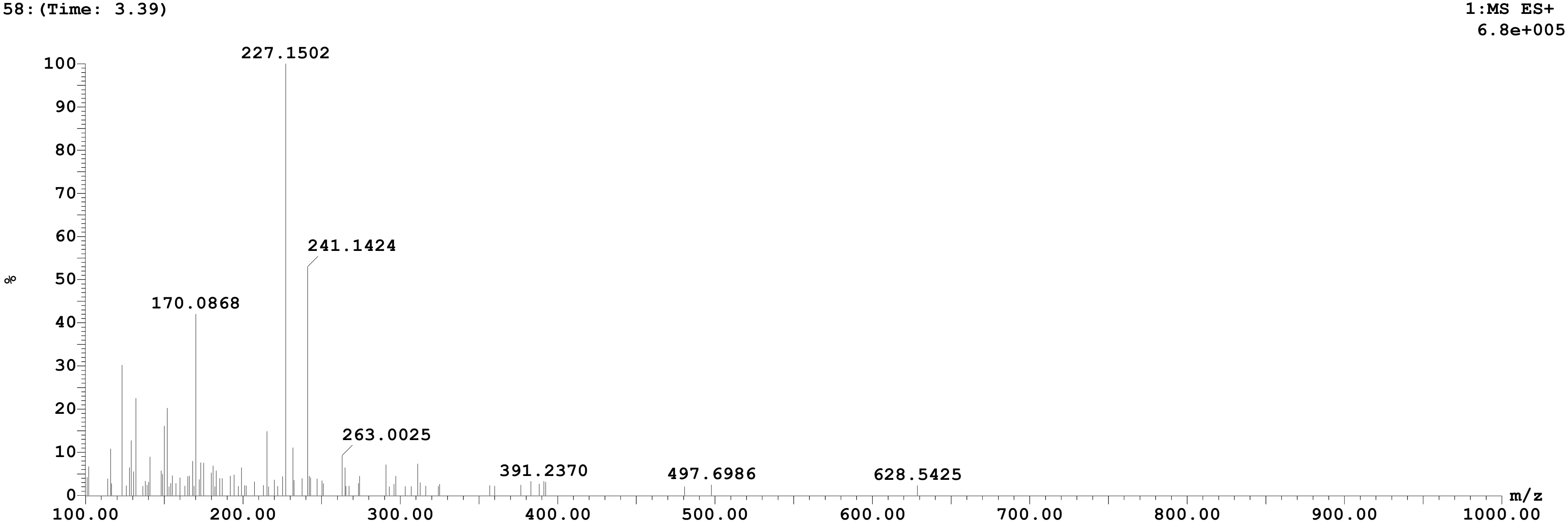

Center for DRUG DISCOVERY RESEARCH and DEVELOPMENT
Openlynx Report -

Sample: 122
File:E2479R
Description:Eth.AC (2)

Vial:1:A,2
Date:06-Nov-2024

ID:
Time:23:37:05

Printed: Sun Nov 24 12:56:41 2024

Peak ID	Time	Error PPM
59	3.43	

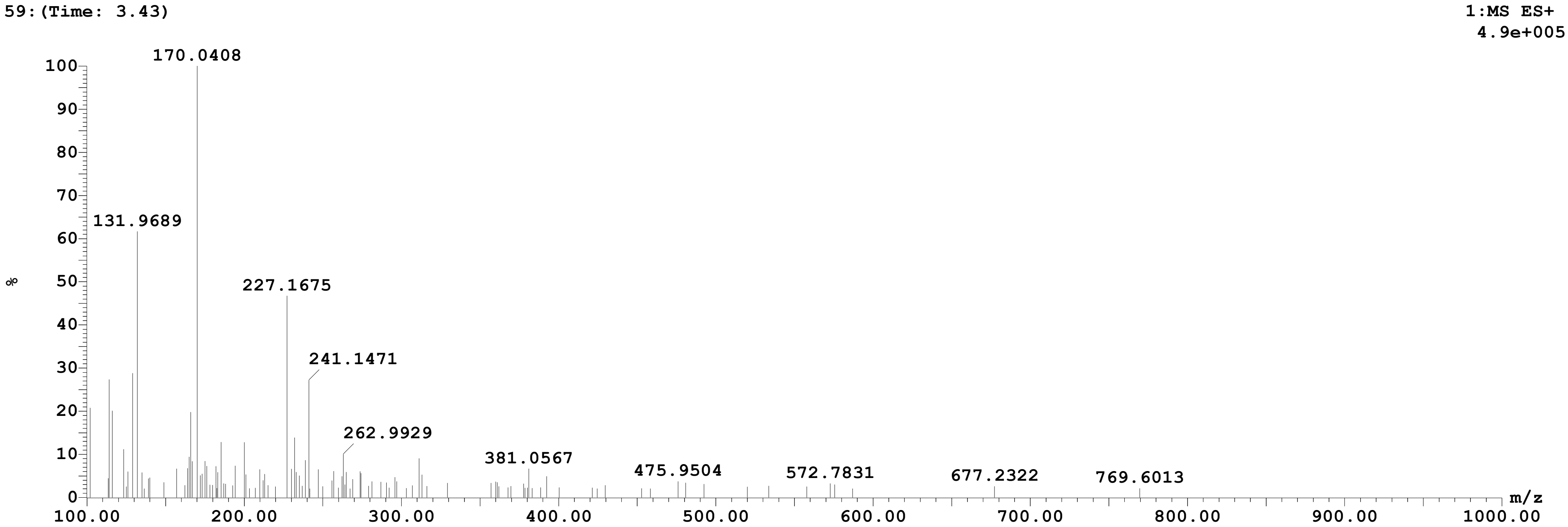

Center for DRUG DISCOVERY RESEARCH and DEVELOPMENT

Openlynx Report -

Sample: 122 Vial:1:A,2 ID:
File:E2479R Date:06-Nov-2024 Time:23:37:05
Description:Eth.AC (2)

Printed: Sun Nov 24 12:56:41 2024

Peak ID	Time	Error PPM
60	3.46	

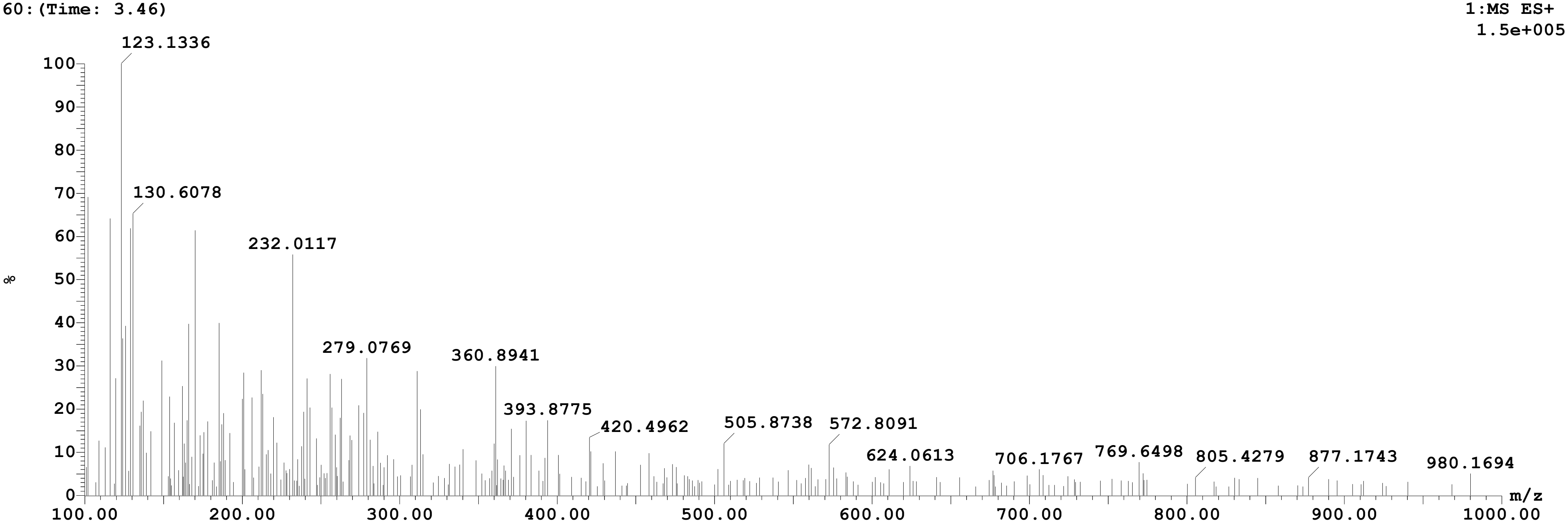

Openlynx Report -

Sample: 122 Vial:1:A,2 ID:
File:E2479R Date:06-Nov-2024 Time:23:37:05
Description:Eth.AC (2)

Printed: Sun Nov 24 12:56:41 2024

Peak ID	Time	Error PPM
61	3.49	

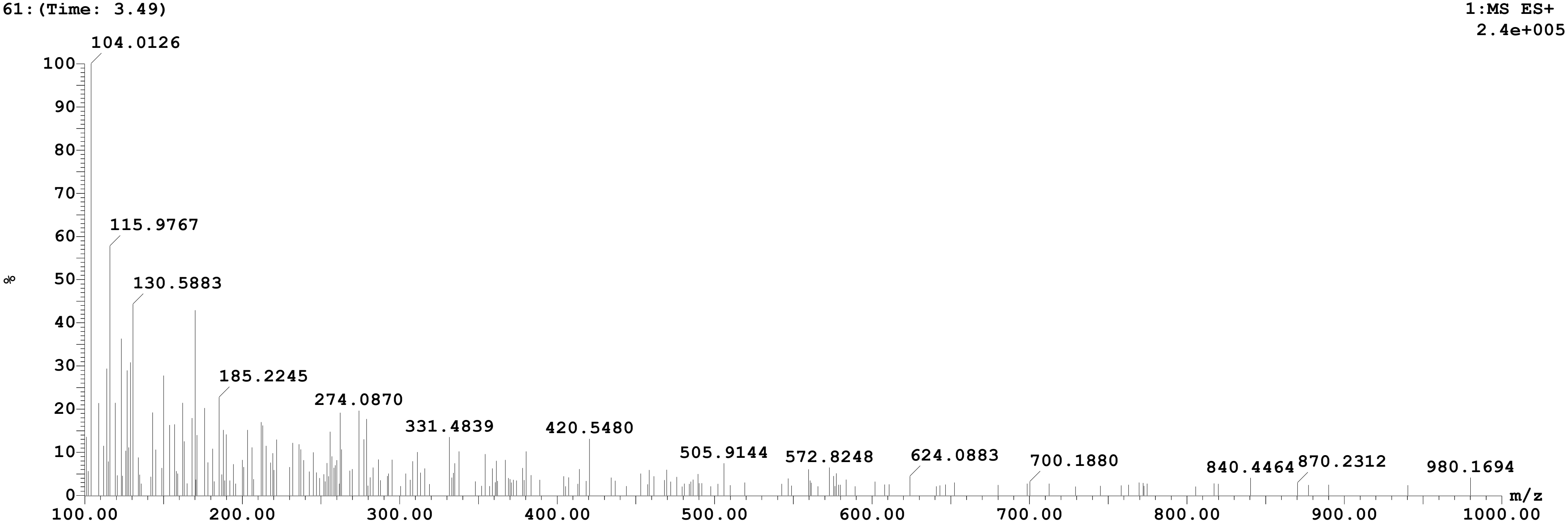

Openlynx Report -

Sample: 122
File:E2479R
Description:Eth.AC (2)

Vial:1:A,2
Date:06-Nov-2024

ID:
Time:23:37:05

Printed: Sun Nov 24 12:56:41 2024

Peak ID	Time	Error PPM
62	3.60	

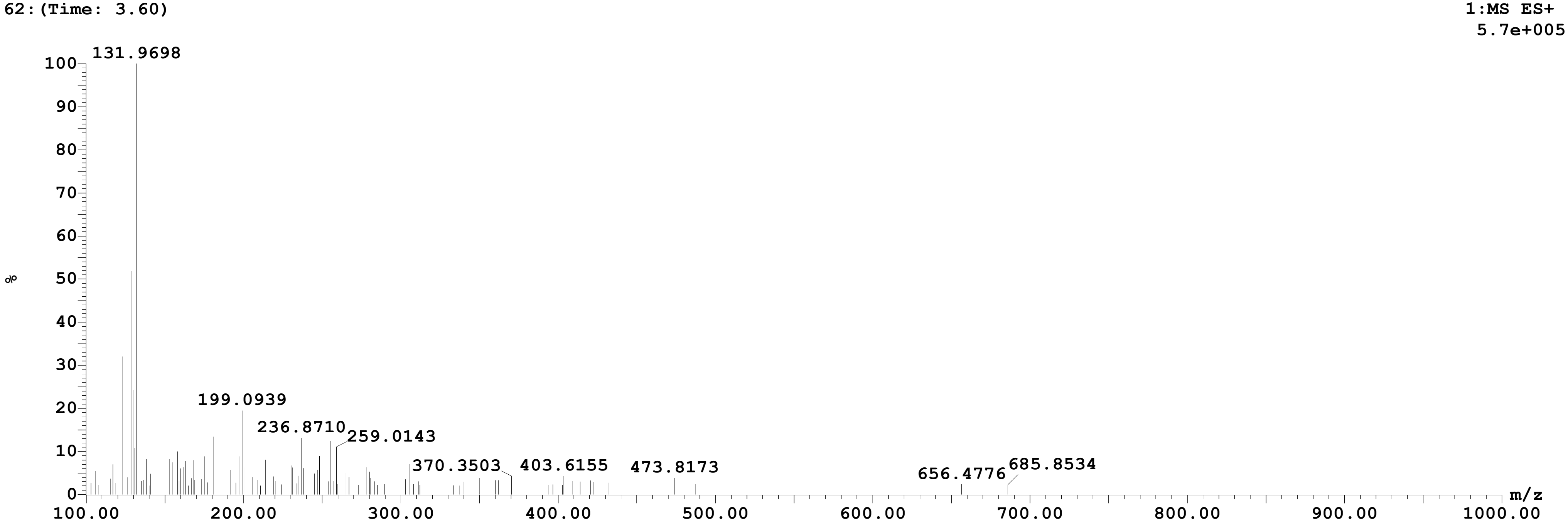

Center for DRUG DISCOVERY RESEARCH and DEVELOPMENT
Openlynx Report -

Sample: 122 | Vial:1:A,2 | ID:
File:E2479R | Date:06-Nov-2024 | Time:23:37:05
Description:Eth.AC (2)

Printed: Sun Nov 24 12:56:41 2024

Peak ID	Time	Error PPM
63	3.69	

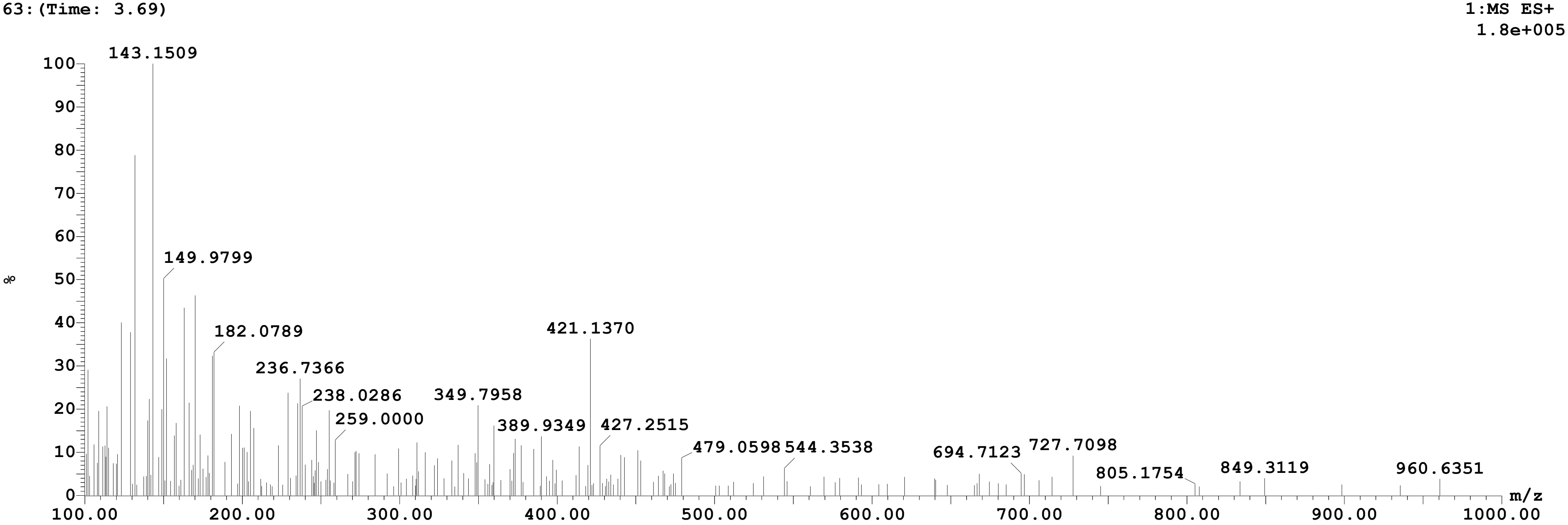

Center for DRUG DISCOVERY RESEARCH and DEVELOPMENT
Openlynx Report -

Sample: 122 Vial:1:A,2 ID:
File:E2479R Date:06-Nov-2024 Time:23:37:05
Description:Eth.AC (2)

Printed: Sun Nov 24 12:56:41 2024

Peak ID	Time	Error PPM
64	3.72	

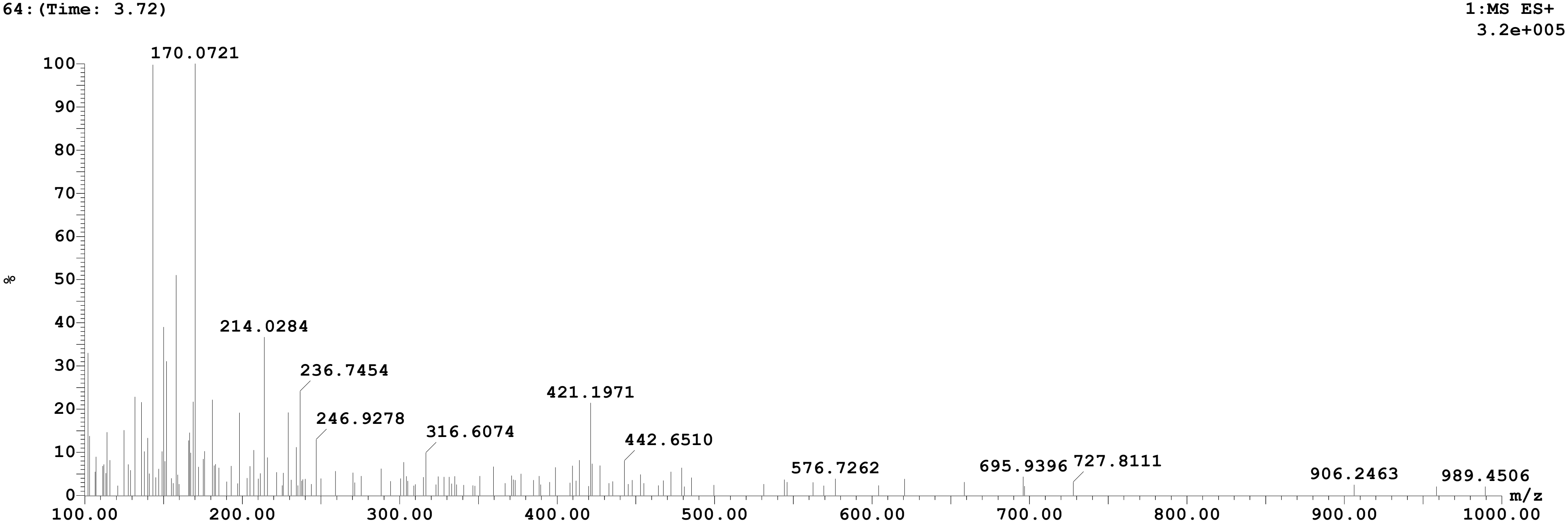

Center for DRUG DISCOVERY RESEARCH and DEVELOPMENT
Openlynx Report -

Sample: 122 Vial:1:A,2 ID:
File:E2479R Date:06-Nov-2024 Time:23:37:05
Description:Eth.AC (2)

Printed: Sun Nov 24 12:56:41 2024

Peak ID	Time	Error PPM
65	3.76	

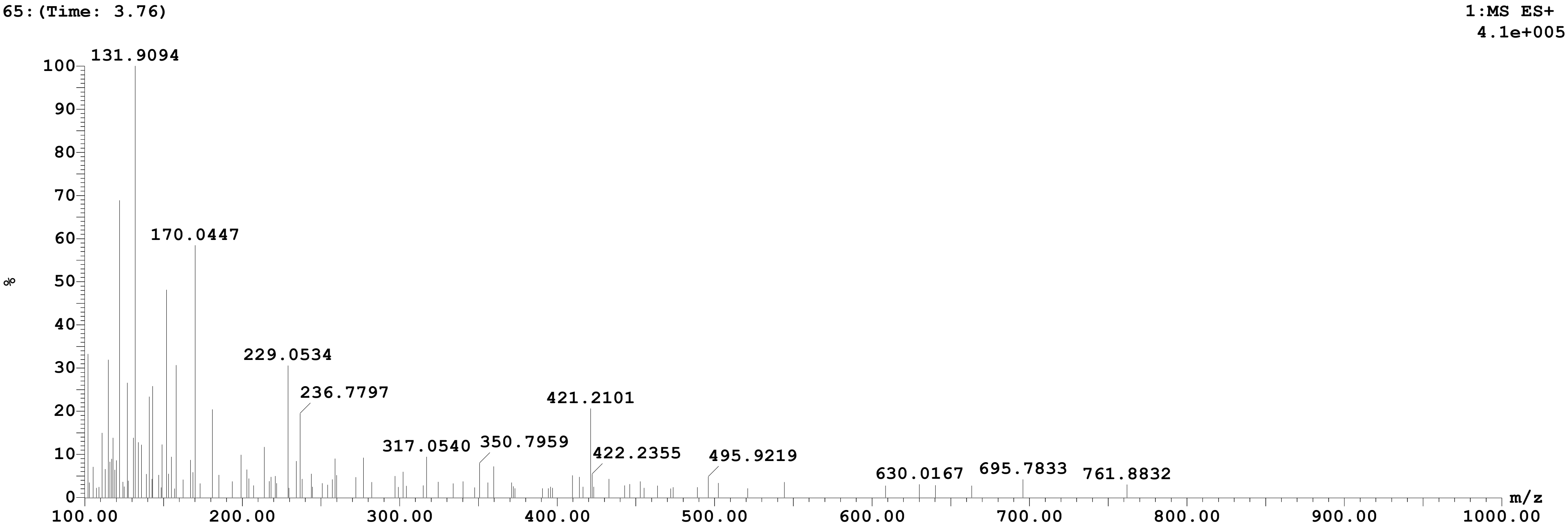

Center for DRUG DISCOVERY RESEARCH and DEVELOPMENT

Openlynx Report -

Sample: 122 Vial:1:A,2 ID:
File:E2479R Date:06-Nov-2024 Time:23:37:05
Description:Eth.AC (2)

Printed: Sun Nov 24 12:56:41 2024

Peak ID	Time	Error PPM
66	3.92	

66:(Time: 3.92)

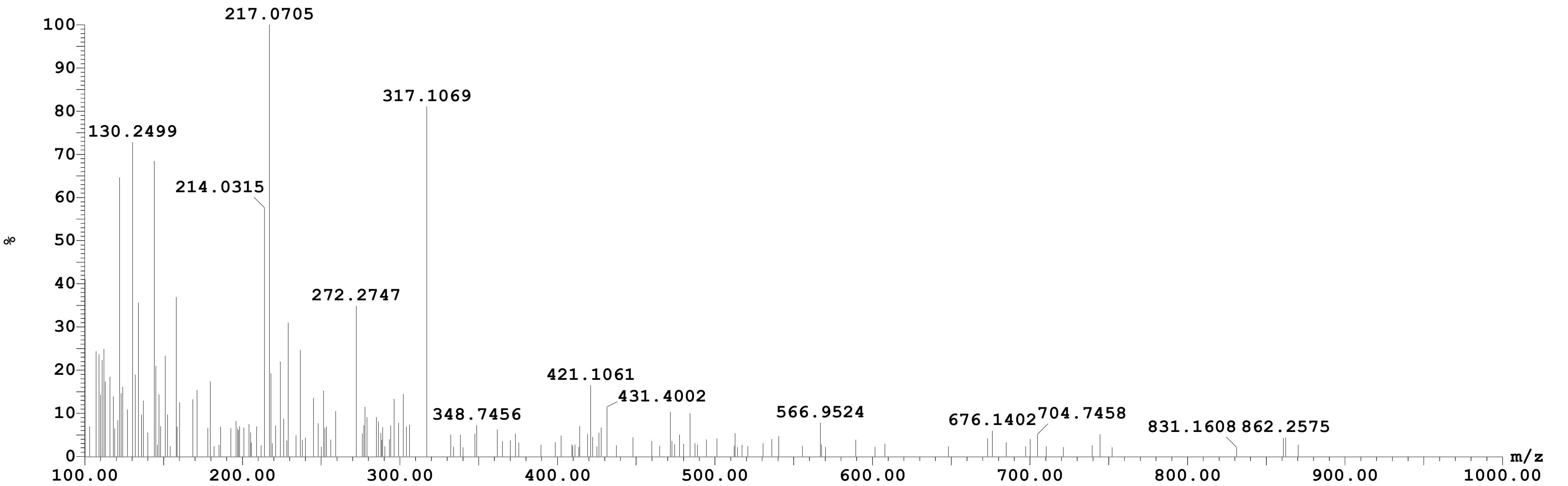

Center for DRUG DISCOVERY RESEARCH and DEVELOPMENT
Openlynx Report -

Sample: 122
File:E2479R
Description:Eth.AC (2)

Vial:1:A,2
Date:06-Nov-2024

ID:
Time:23:37:05

Printed: Sun Nov 24 12:56:41 2024

Peak ID	Time	Error PPM
67	3.99	

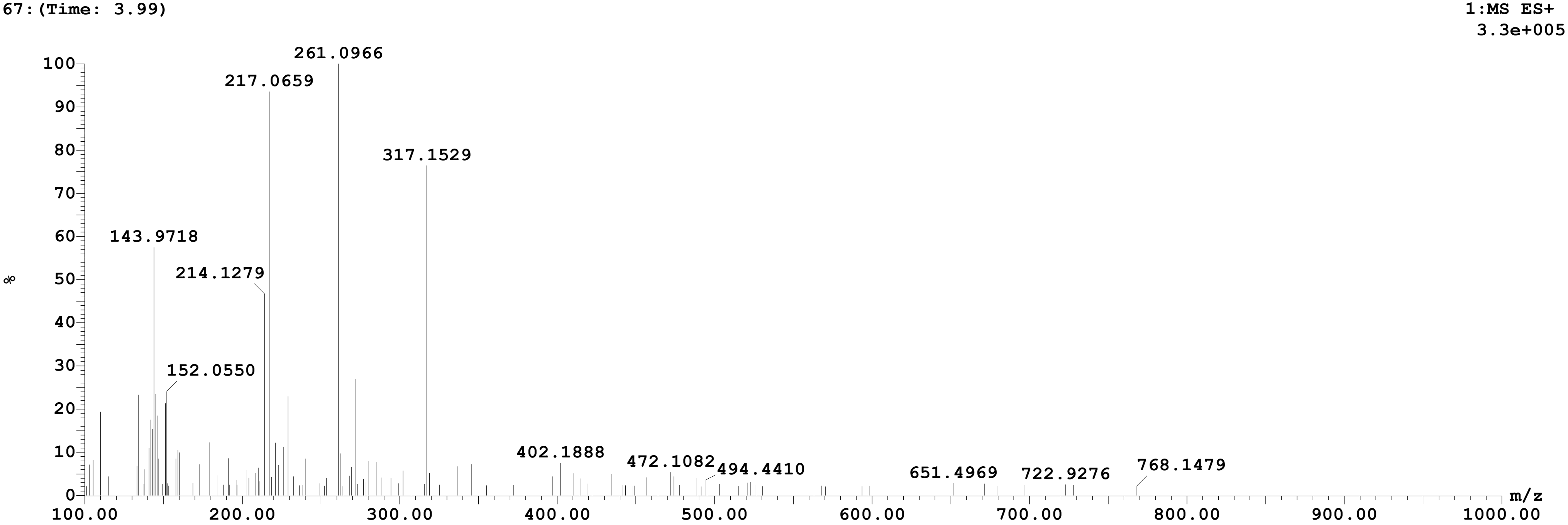

Center for DRUG DISCOVERY RESEARCH and DEVELOPMENT
Openlynx Report -

Sample: 122 | Vial:1:A,2 | ID:
File:E2479R | Date:06-Nov-2024 | Time:23:37:05
Description:Eth.AC (2)

Printed: Sun Nov 24 12:56:41 2024

Peak ID	Time	Error PPM
68	4.03	

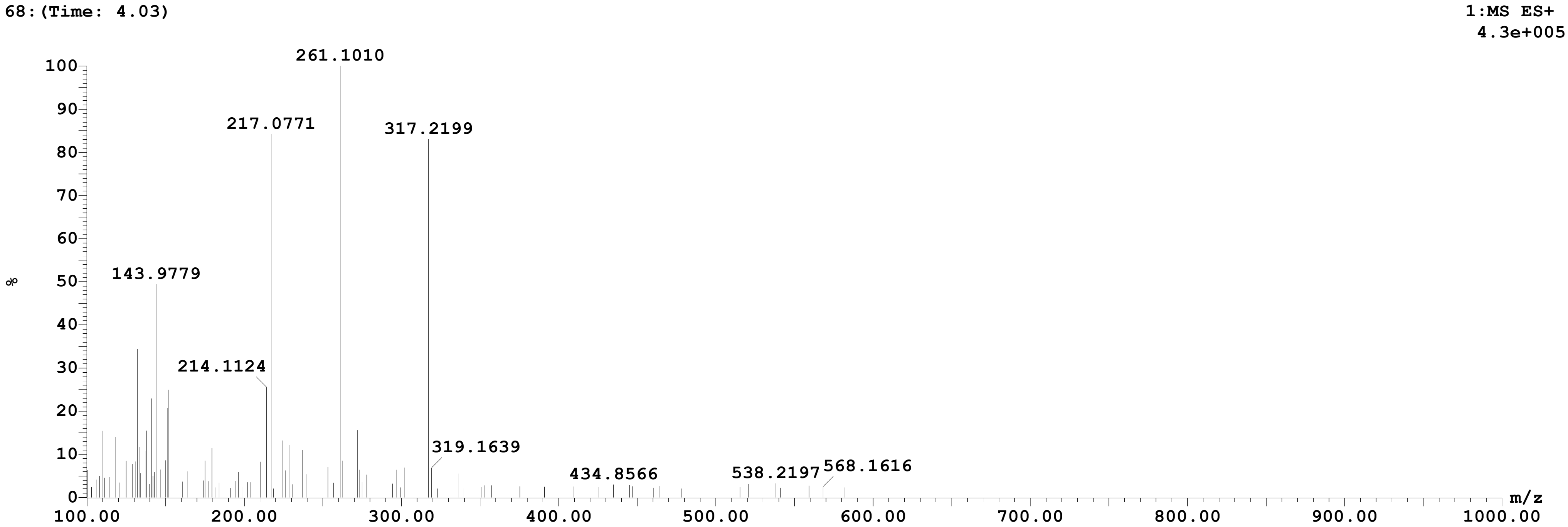

Openlynx Report -

Sample: 122 Vial:1:A,2 ID:
File:E2479R Date:06-Nov-2024 Time:23:37:05
Description:Eth.AC (2)

Printed: Sun Nov 24 12:56:41 2024

Peak ID	Time	Error PPM
69	4.07	

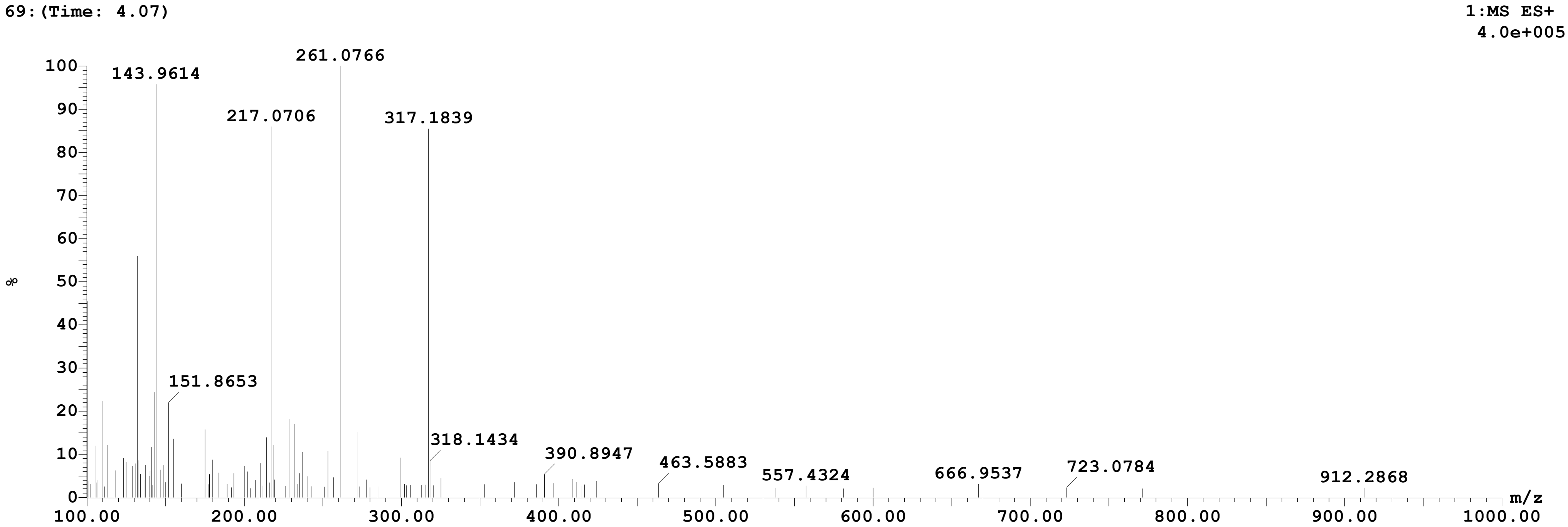

Center for DRUG DISCOVERY RESEARCH and DEVELOPMENT
Openlynx Report -

Sample: 122　　Vial:1:A,2　　ID:
File:E2479R　　Date:06-Nov-2024　　Time:23:37:05
Description:Eth.AC (2)

Printed: Sun Nov 24 12:56:41 2024

Peak ID	Time	Error PPM
70	4.10	

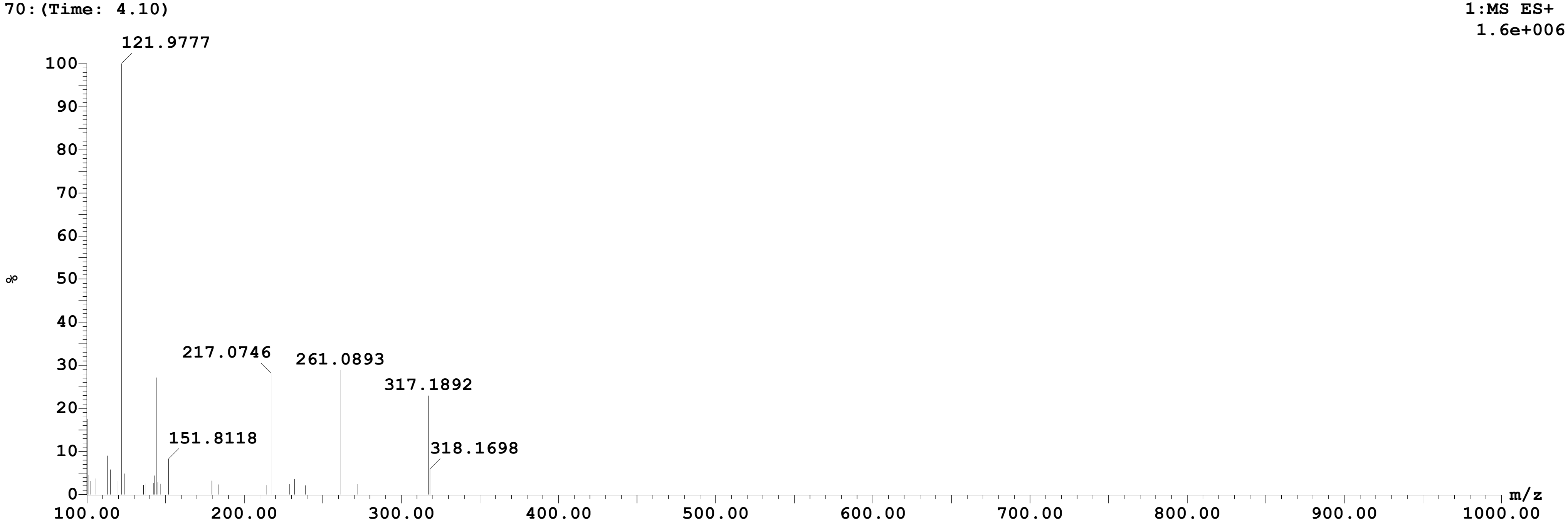

Center for DRUG DISCOVERY RESEARCH and DEVELOPMENT
Openlynx Report -

Sample: 122 | Vial:1:A,2 | ID:
File:E2479R | Date:06-Nov-2024 | Time:23:37:05
Description:Eth.AC (2)

Printed: Sun Nov 24 12:56:41 2024

Peak ID	Time	Error PPM
71	4.18	

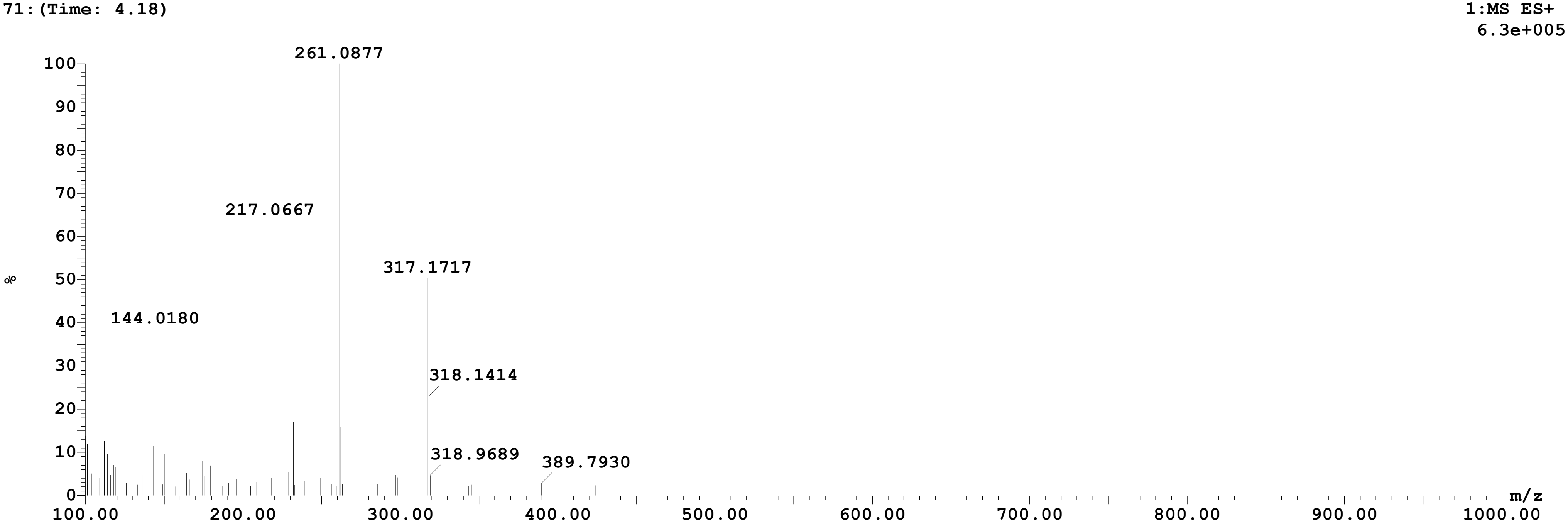

Center for DRUG DISCOVERY RESEARCH and DEVELOPMENT
Openlynx Report -

Sample: 122 Vial:1:A,2 ID:
File:E2479R Date:06-Nov-2024 Time:23:37:05
Description:Eth.AC (2)

Printed: Sun Nov 24 12:56:41 2024

Peak ID	Time	Error PPM
72	4.28	

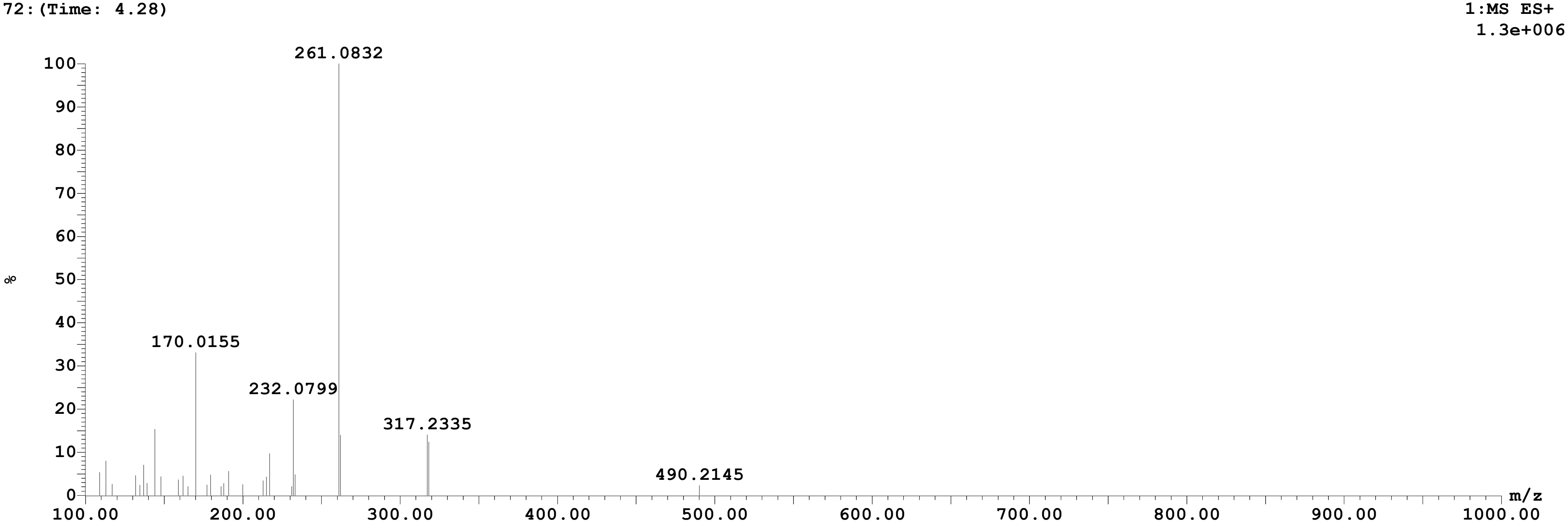

Center for DRUG DISCOVERY RESEARCH and DEVELOPMENT
Openlynx Report -

Sample: 122 | Vial:1:A,2 | ID:
File:E2479R | Date:06-Nov-2024 | Time:23:37:05
Description:Eth.AC (2)

Printed: Sun Nov 24 12:56:41 2024

Peak ID	Time	Error PPM
73	4.55	

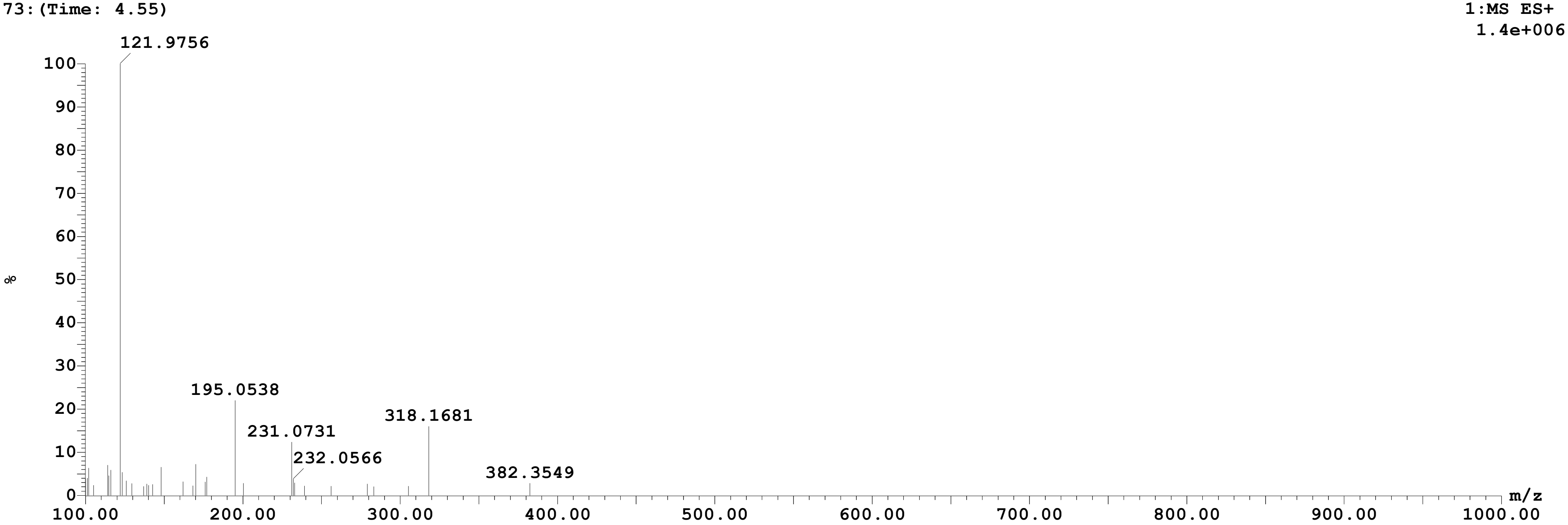

Center for DRUG DISCOVERY RESEARCH and DEVELOPMENT
Openlynx Report -

Sample: 122 | Vial:1:A,2 | ID:
File:E2479R | Date:06-Nov-2024 | Time:23:37:05
Description:Eth.AC (2)

Printed: Sun Nov 24 12:56:41 2024

Peak ID	Time	Error PPM
74	4.62	

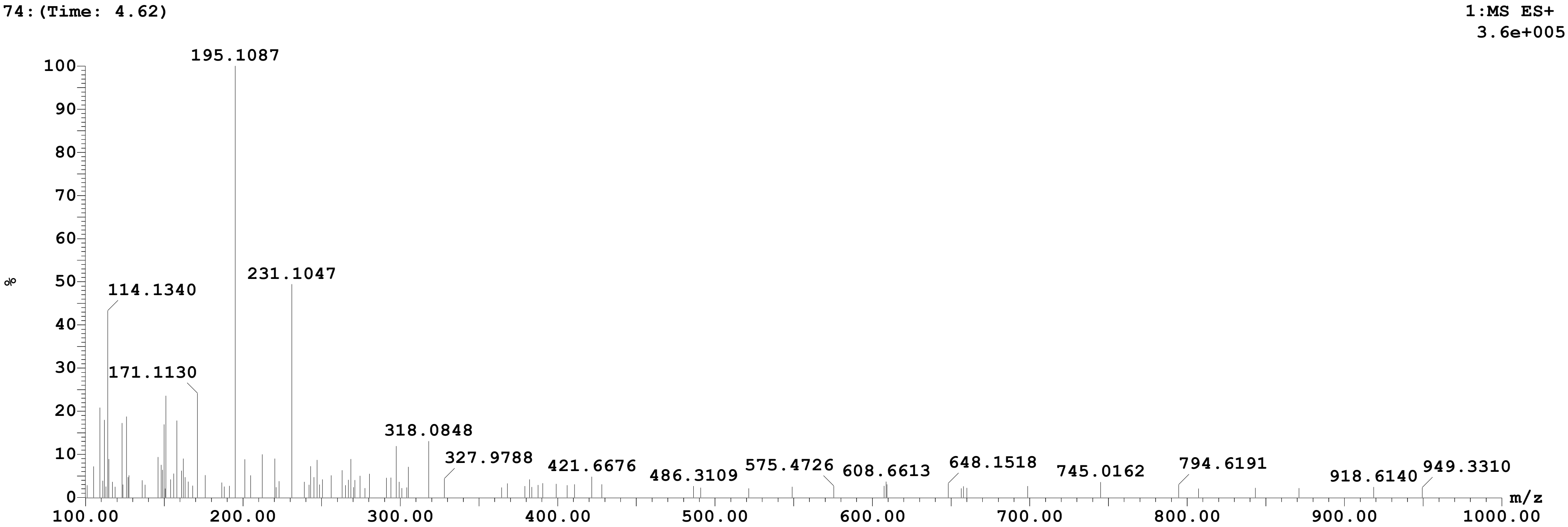

Center for DRUG DISCOVERY RESEARCH and DEVELOPMENT
Openlynx Report -

Sample: 122 Vial:1:A,2 ID:
File:E2479R Date:06-Nov-2024 Time:23:37:05
Description:Eth.AC (2)

Printed: Sun Nov 24 12:56:41 2024

Peak ID	Time	Error PPM
75	4.69	

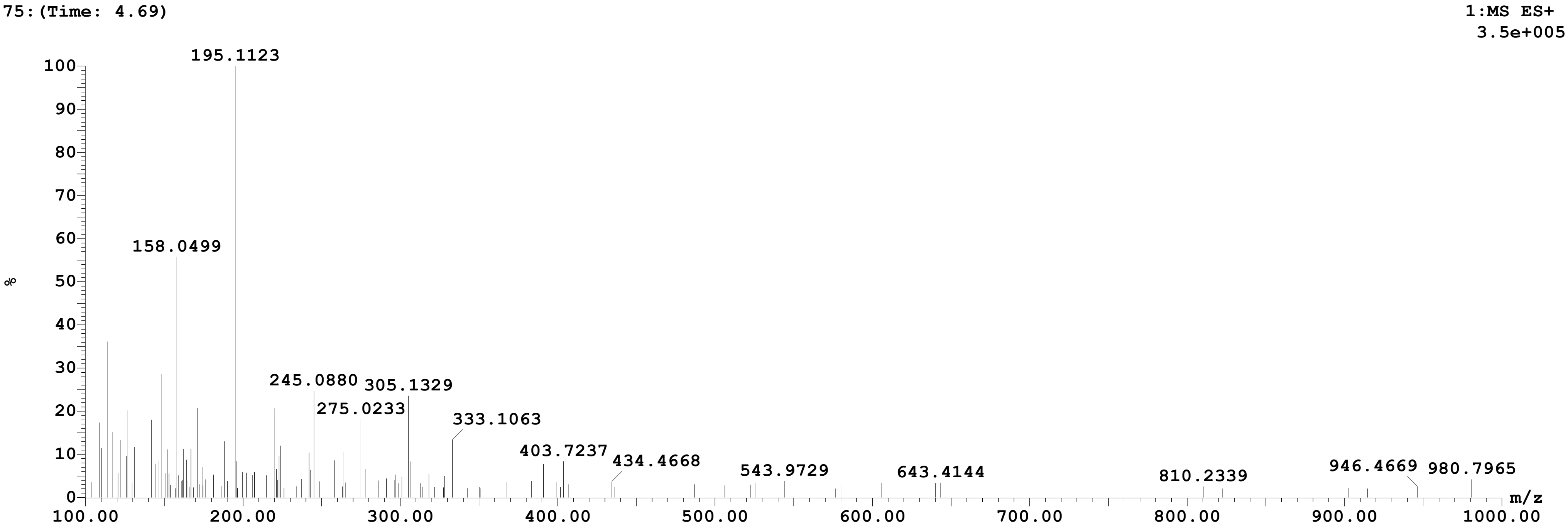

Center for DRUG DISCOVERY RESEARCH and DEVELOPMENT

Openlynx Report -

Sample: 122	Vial:1:A,2	ID:
File:E2479R	Date:06-Nov-2024	Time:23:37:05
Description:Eth.AC (2)		

Printed: Sun Nov 24 12:56:41 2024

Peak ID	Time	Error PPM
76	4.85	

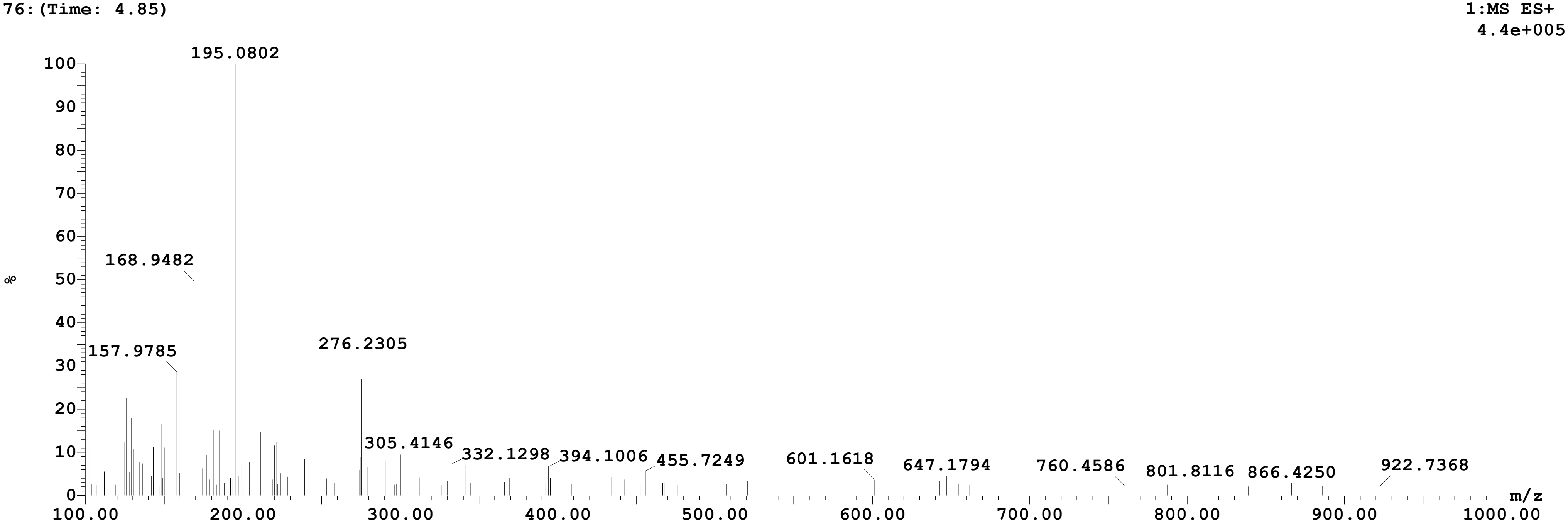

Center for DRUG DISCOVERY RESEARCH and DEVELOPMENT
Openlynx Report -

Sample: 122 | Vial:1:A,2 | ID:
File:E2479R | Date:06-Nov-2024 | Time:23:37:05
Description:Eth.AC (2)

Printed: Sun Nov 24 12:56:41 2024

Peak ID	Time	Error PPM
77	5.07	

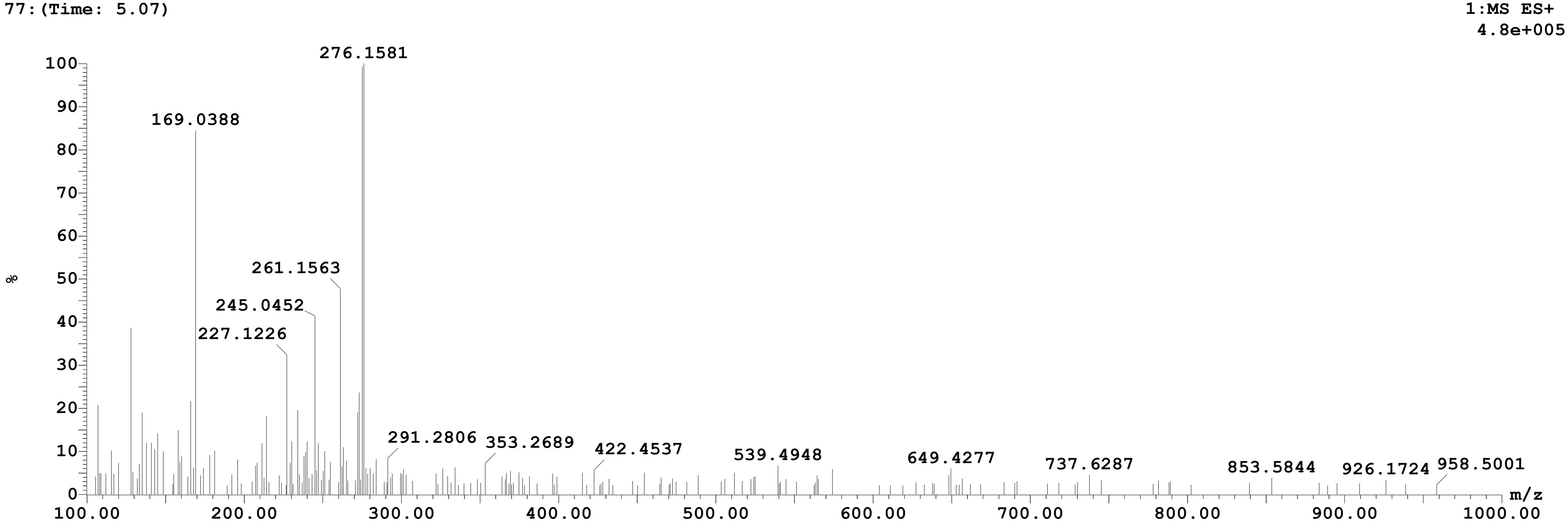

Center for DRUG DISCOVERY RESEARCH and DEVELOPMENT
Openlynx Report -

Sample: 122 | Vial:1:A,2 | ID:
File:E2479R | Date:06-Nov-2024 | Time:23:37:05
Description:Eth.AC (2)

Printed: Sun Nov 24 12:56:41 2024

Peak ID	Time	Error PPM
78	6.38	

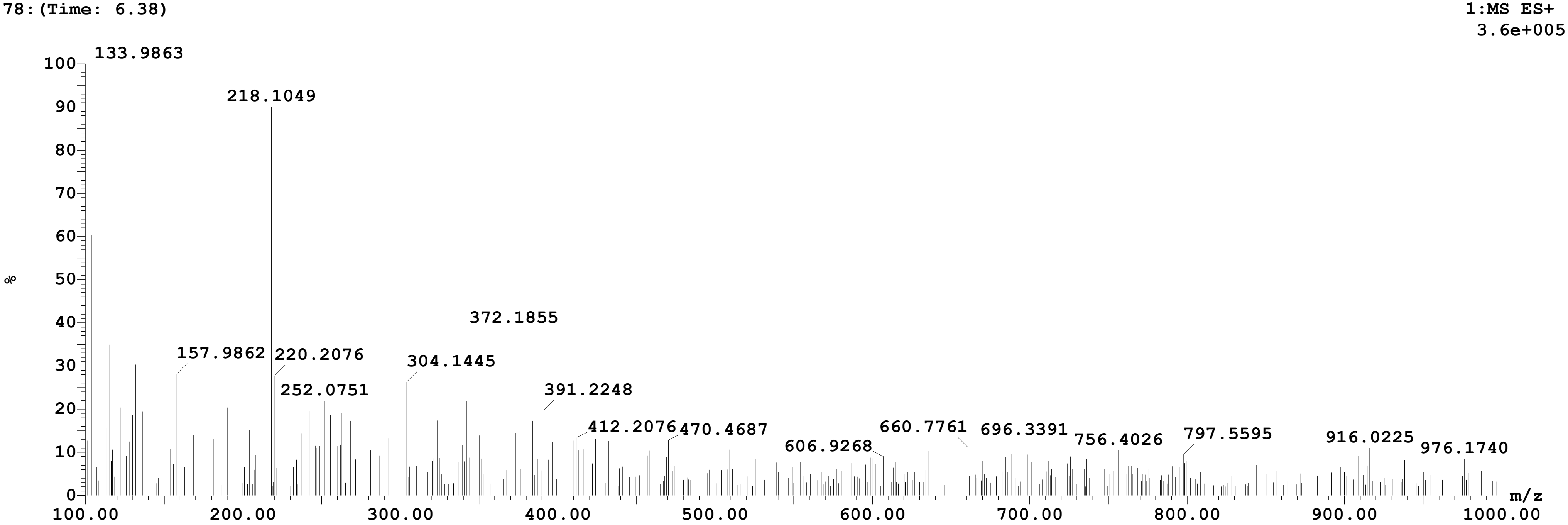

Center for DRUG DISCOVERY RESEARCH and DEVELOPMENT
Openlynx Report -

Sample: 122 | Vial:1:A,2 | ID:
File:E2479R | Date:06-Nov-2024 | Time:23:37:05
Description:Eth.AC (2)

Printed: Sun Nov 24 12:56:41 2024

Peak ID	Time	Error PPM
79	11.15	

79: (Time: 11.15)

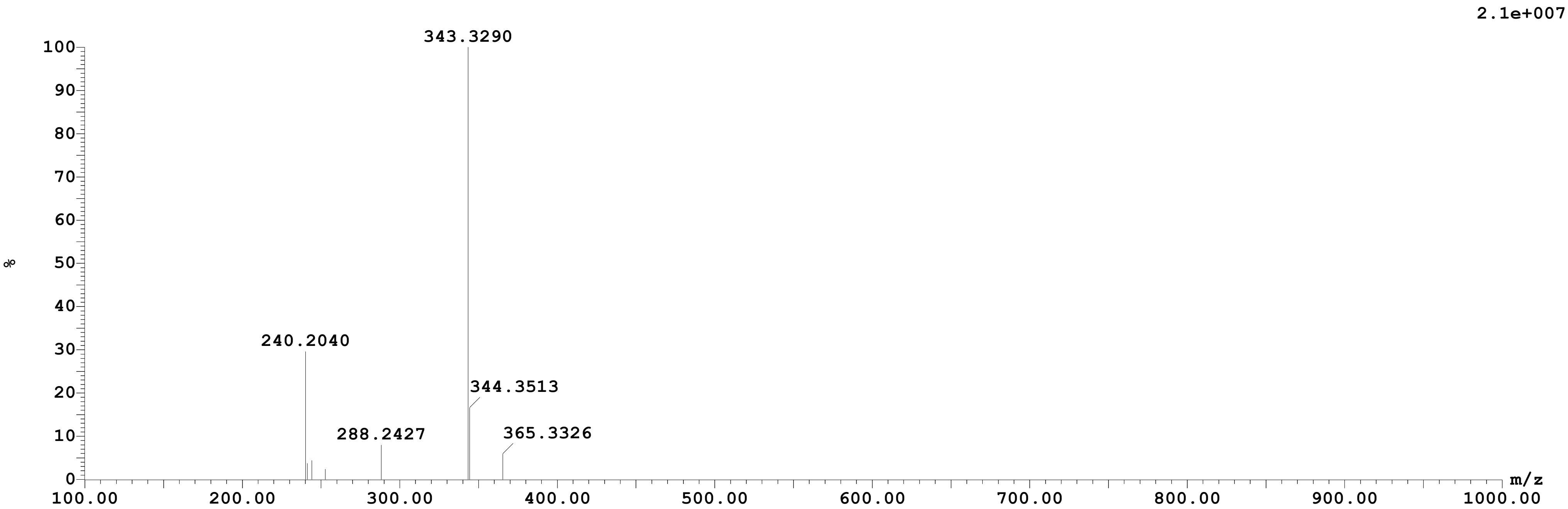

Openlynx Report -

Sample: 122 Vial:1:A,2 ID:
File:E2479R Date:06-Nov-2024 Time:23:37:05
Description:Eth.AC (2)

Printed: Sun Nov 24 12:56:41 2024

Peak ID	Time	Error PPM
80	11.20	

80:(Time: 11.20)

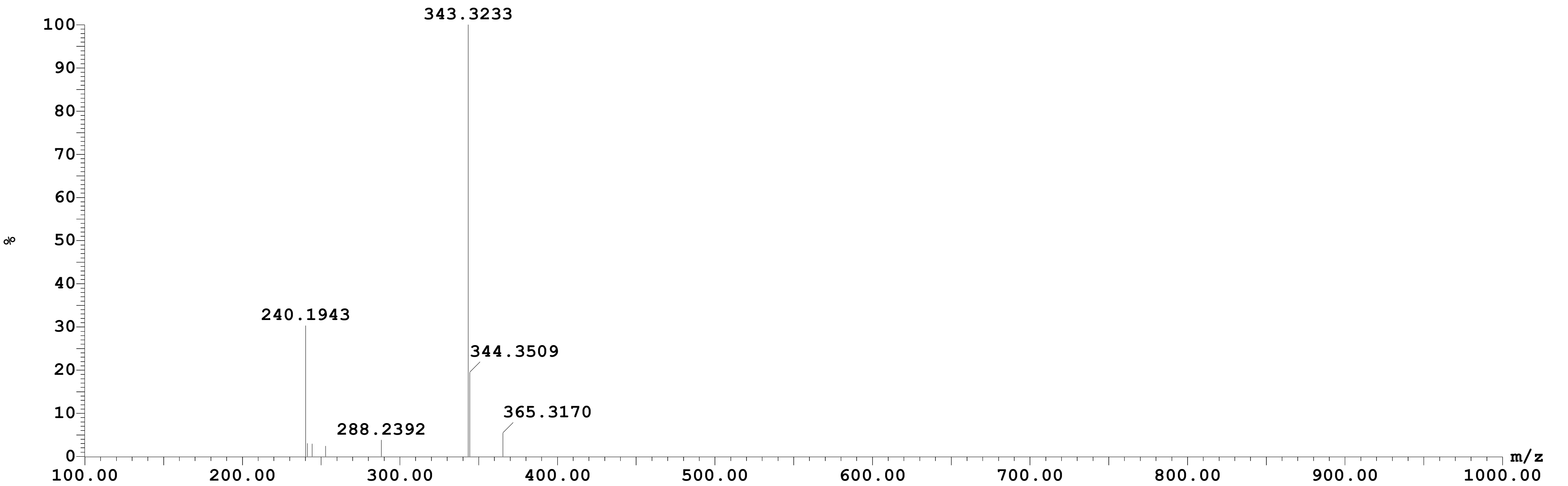

Center for DRUG DISCOVERY RESEARCH and DEVELOPMENT
Openlynx Report -

Sample: 122 Vial:1:A,2 ID:
File:E2479R Date:06-Nov-2024 Time:23:37:05
Description:Eth.AC (2)

Printed: Sun Nov 24 12:56:41 2024

Peak ID	Time	Error PPM
81	13.28	

81:(Time: 13.28)

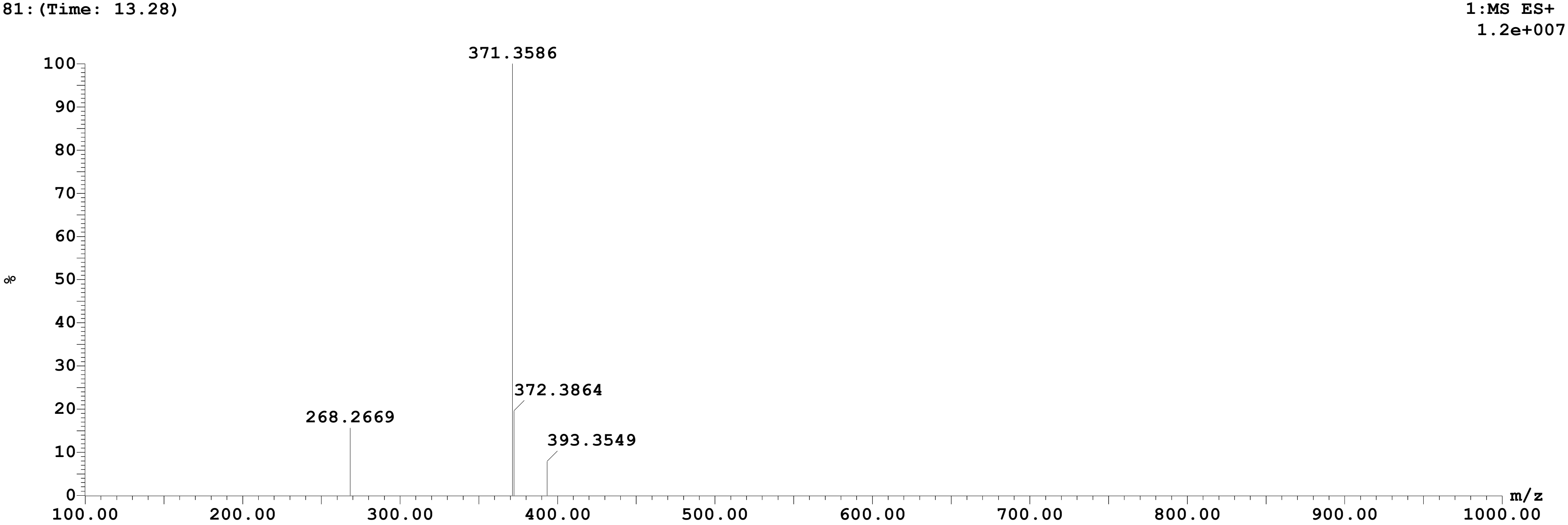

Openlynx Report -

Sample: 122 Vial:1:A,2 ID:
File:E2479R Date:06-Nov-2024 Time:23:37:05
Description:Eth.AC (2)

Printed: Sun Nov 24 12:56:41 2024

Peak ID	Time	Error PPM
82	13.32	

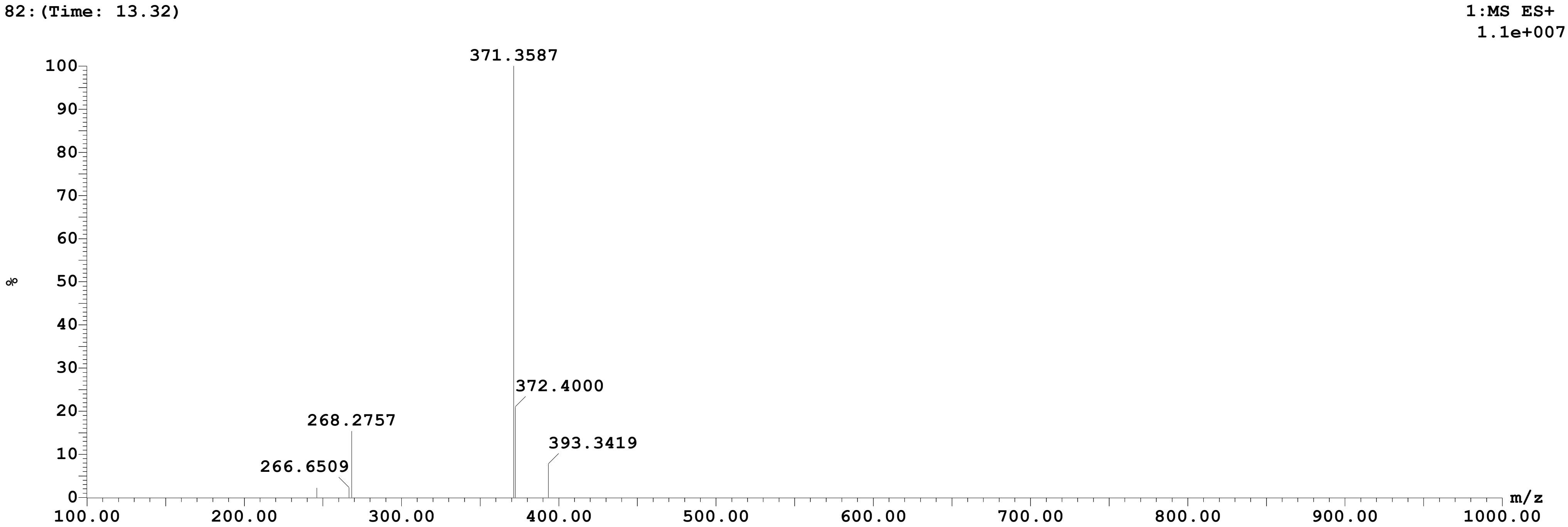

Center for DRUG DISCOVERY RESEARCH and DEVELOPMENT
Openlynx Report -

Sample: 122 Vial:1:A,2 ID:
File:E2479R Date:06-Nov-2024 Time:23:37:05
Description:Eth.AC (2)

Printed: Sun Nov 24 12:56:41 2024

Peak ID	Time	Error PPM
83	20.94	

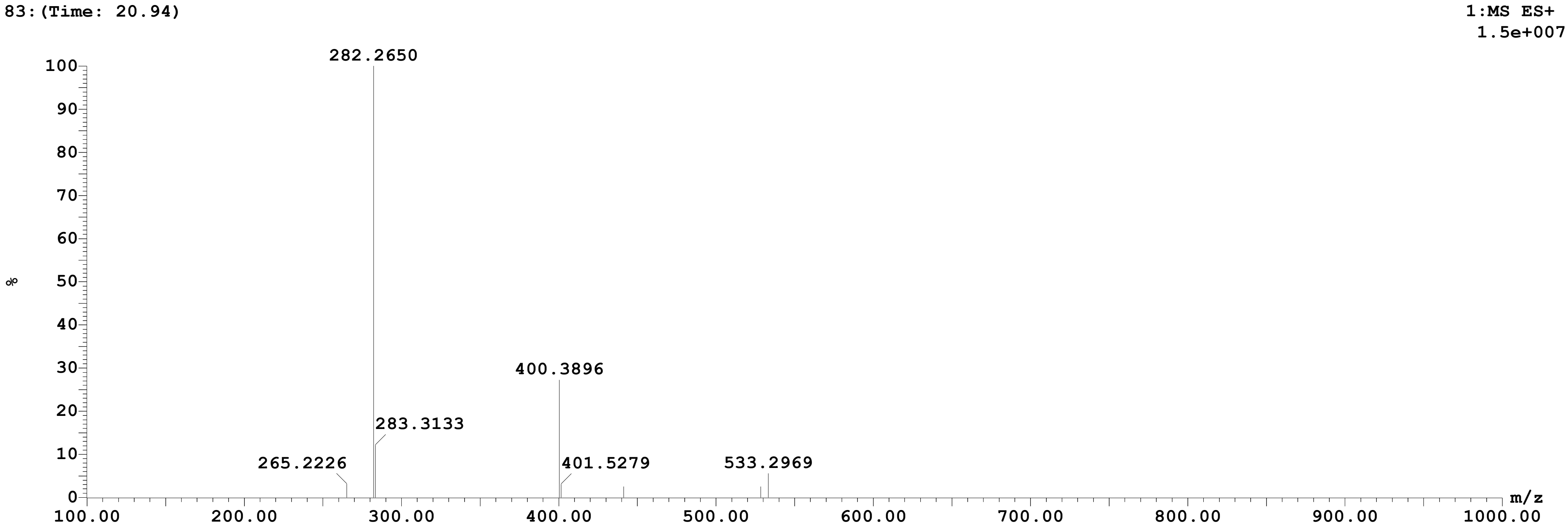

Openlynx Report -

Sample: 122 | Vial:1:A,2 | ID:
File:E2479R | Date:06-Nov-2024 | Time:23:37:05
Description:Eth.AC (2)

Printed: Sun Nov 24 12:56:41 2024

Peak ID	Time	Error PPM
84	23.51	

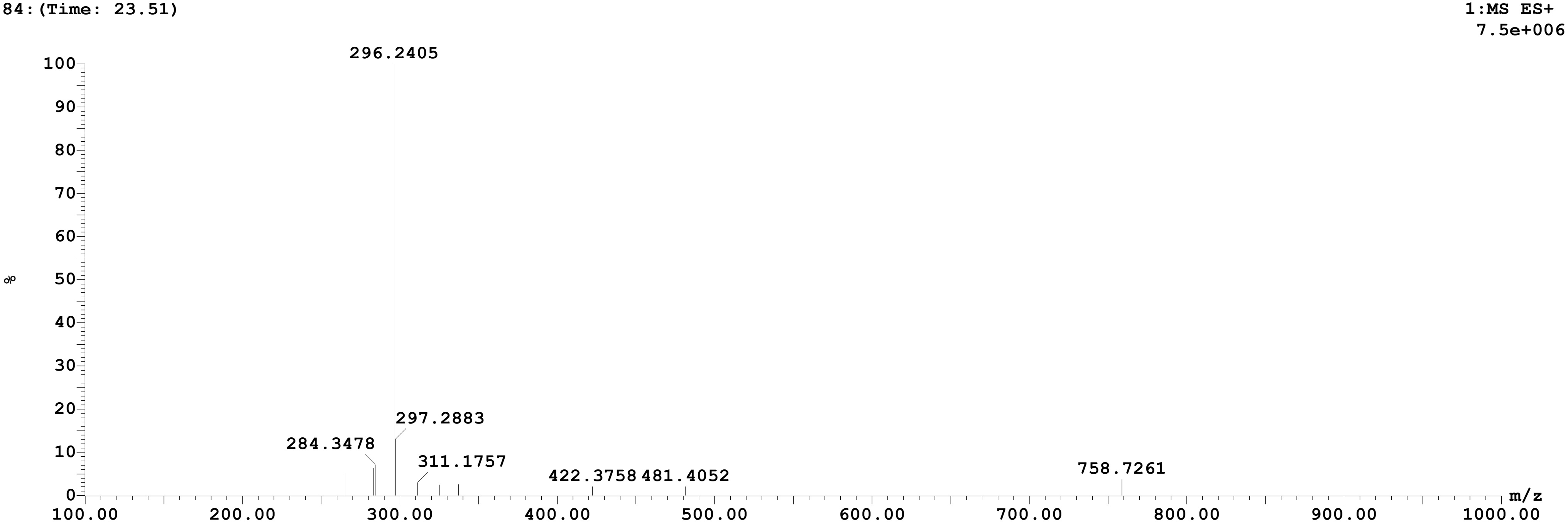

Openlynx Report -

Sample: 122 Vial:1:A,2 ID:
File:E2479R Date:06-Nov-2024 Time:23:37:05
Description:Eth.AC (2)

Printed: Sun Nov 24 12:56:41 2024

Peak ID	Time	Error PPM
85	23.67	

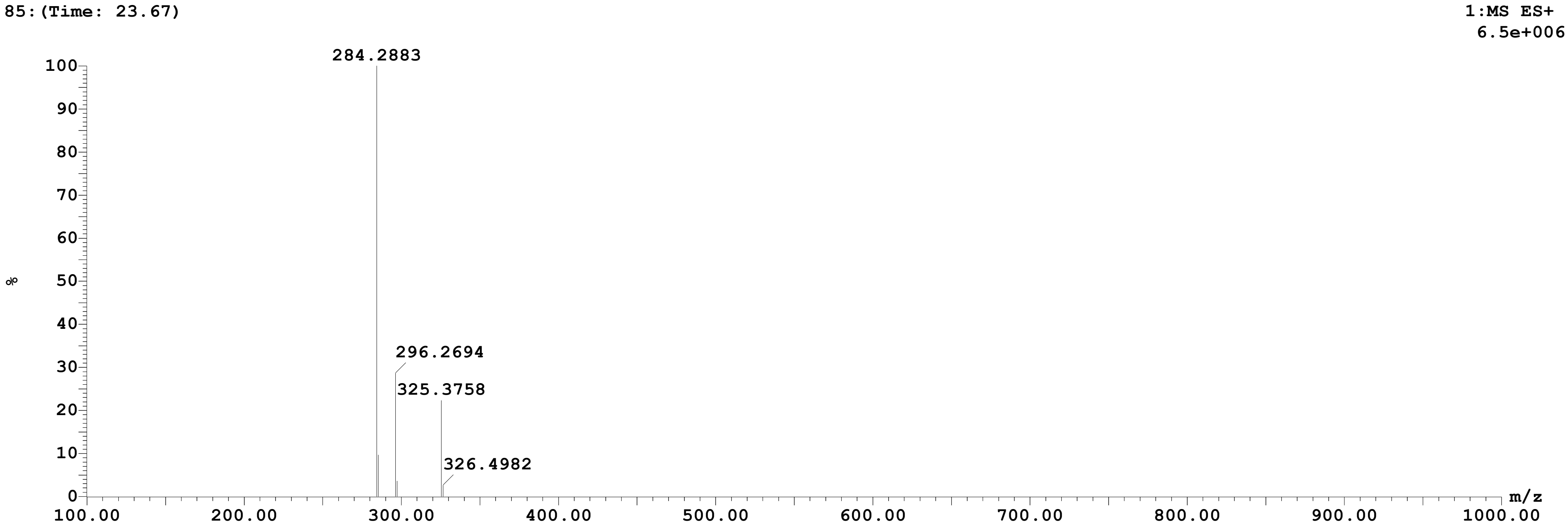

Center for DRUG DISCOVERY RESEARCH and DEVELOPMENT
Openlynx Report -

Sample: 122 | Vial:1:A,2 | ID:
File:E2479R | Date:06-Nov-2024 | Time:23:37:05
Description:Eth.AC (2)

Printed: Sun Nov 24 12:56:41 2024

Peak ID	Time	Error PPM
86	26.16	

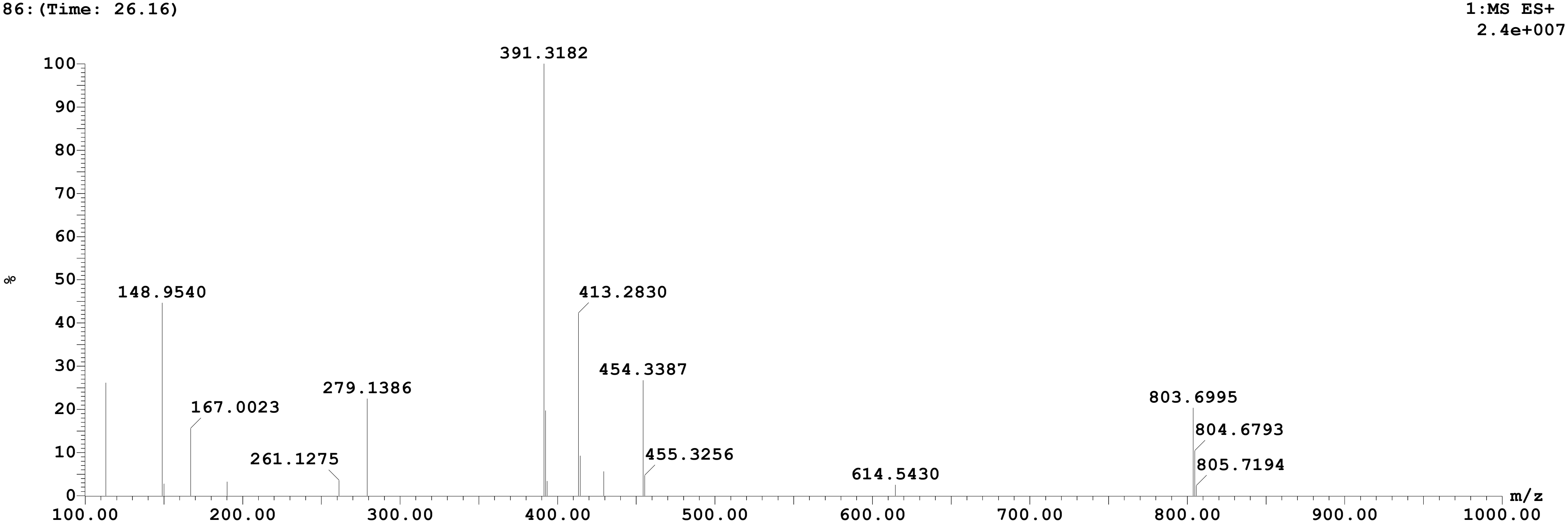

Center for DRUG DISCOVERY RESEARCH and DEVELOPMENT
Openlynx Report -

Sample: 122 Vial:1:A,2 ID:
File:E2479R Date:06-Nov-2024 Time:23:37:05
Description:Eth.AC (2)

Printed: Sun Nov 24 12:56:41 2024

Peak ID	Time	Error PPM
87	26.19	

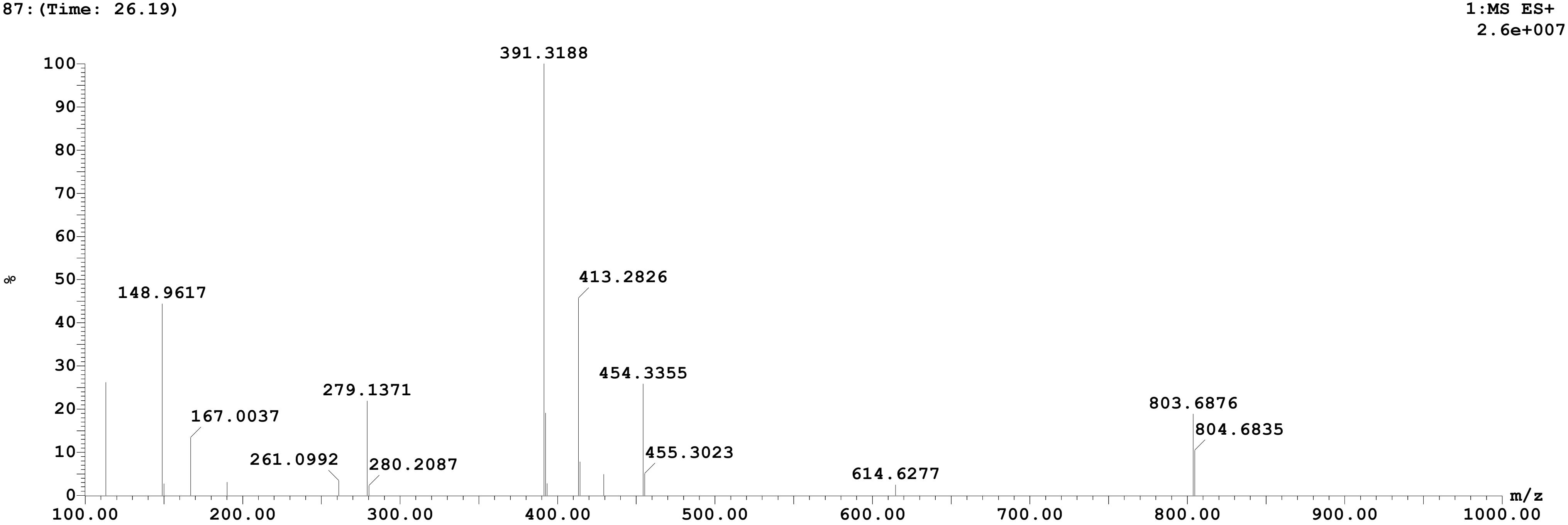

Center for DRUG DISCOVERY RESEARCH and DEVELOPMENT
Openlynx Report -

Sample: 122 | Vial:1:A,2 | ID:
File:E2479R | Date:06-Nov-2024 | Time:23:37:05
Description:Eth.AC (2)

Printed: Sun Nov 24 12:56:41 2024

Peak ID	Time	Error PPM
88	26.24	

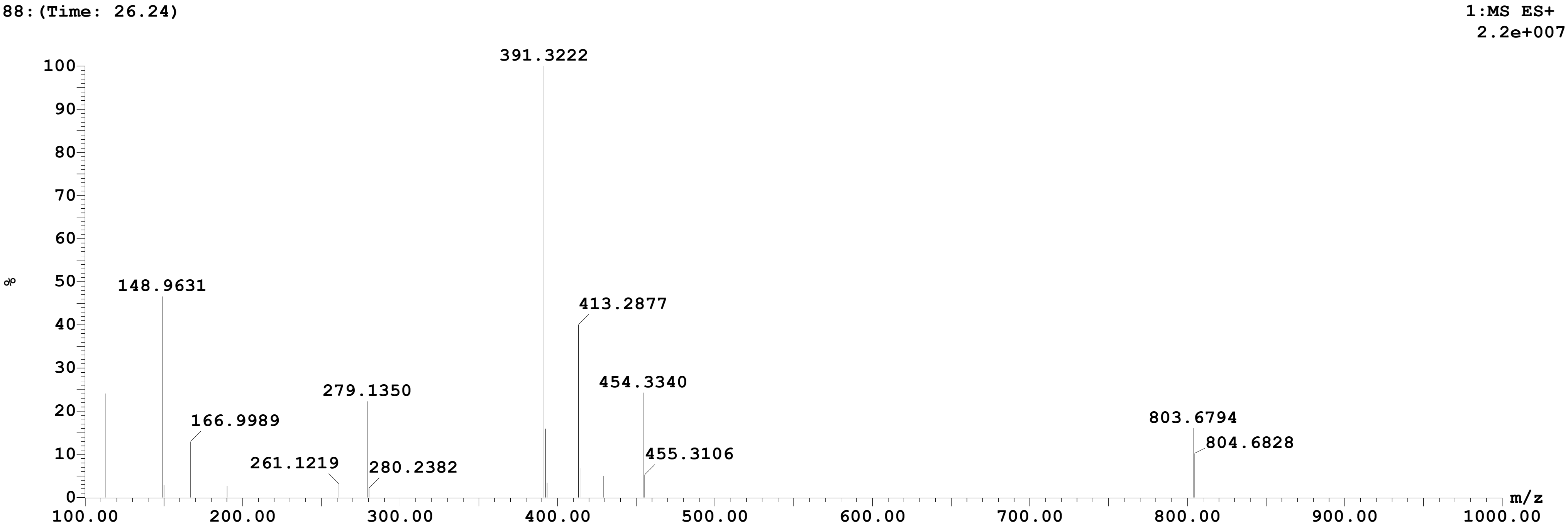

Center for DRUG DISCOVERY RESEARCH and DEVELOPMENT
Openlynx Report -

Sample: 122
File:E2479R
Description:Eth.AC (2)

Vial:1:A,2
Date:06-Nov-2024

ID:
Time:23:37:05

Printed: Sun Nov 24 12:56:41 2024

Peak ID	Time	Error PPM
89	28.37	

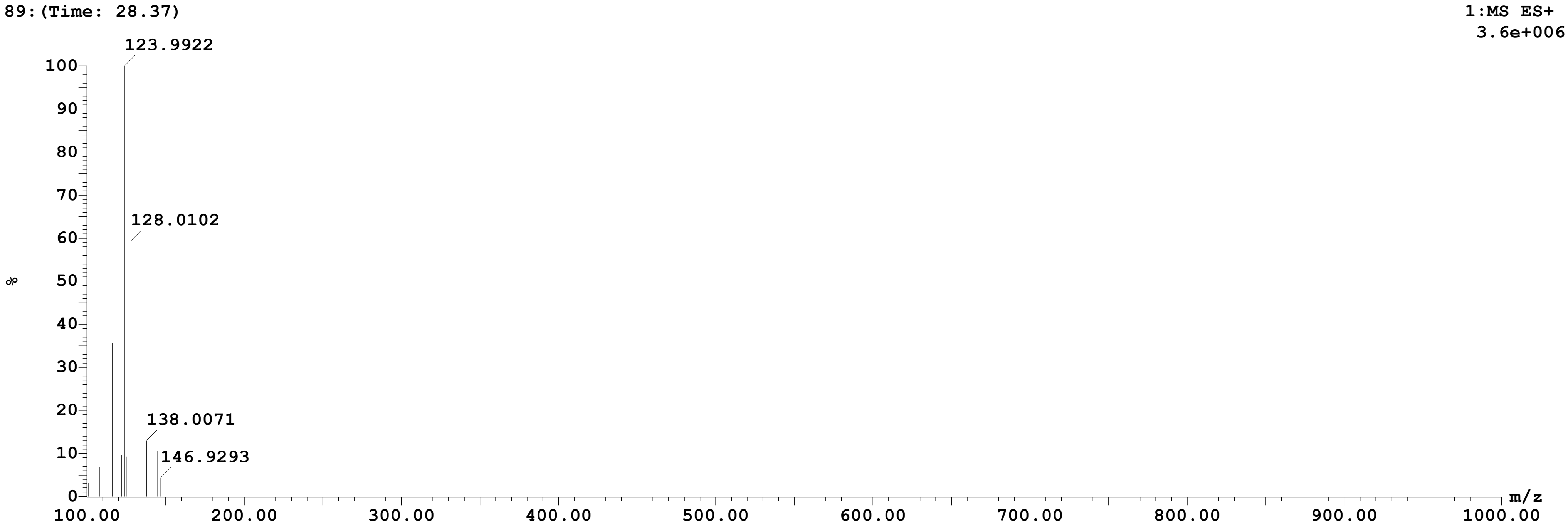

Openlynx Report -

Sample: 122 Vial:1:A,2 ID:
File:E2479R Date:06-Nov-2024 Time:23:37:05
Description:Eth.AC (2)

Printed: Sun Nov 24 12:56:41 2024

Peak ID	Time	Error PPM
90	30.89	

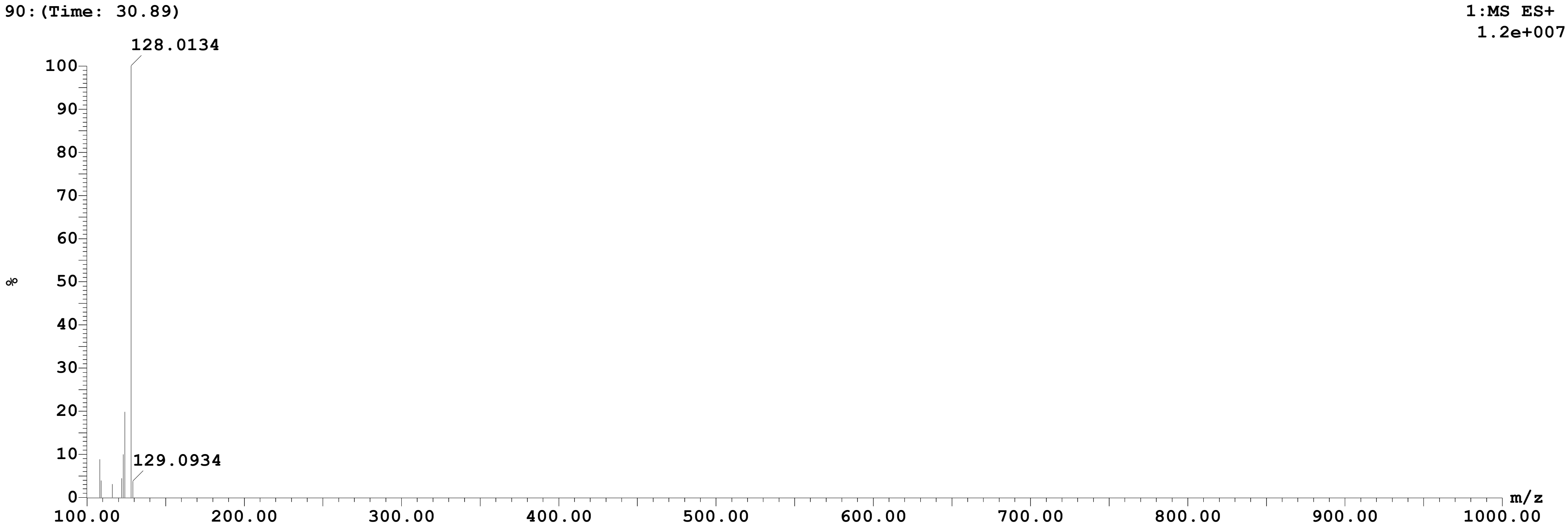

Openlynx Report -

Sample: 122
Vial:1:A,2
ID:
File:E2479R
Date:06-Nov-2024
Time:23:37:05
Description:Eth.AC (2)

Printed: Sun Nov 24 12:56:41 2024

Peak ID	Time	Error PPM
91	30.96	

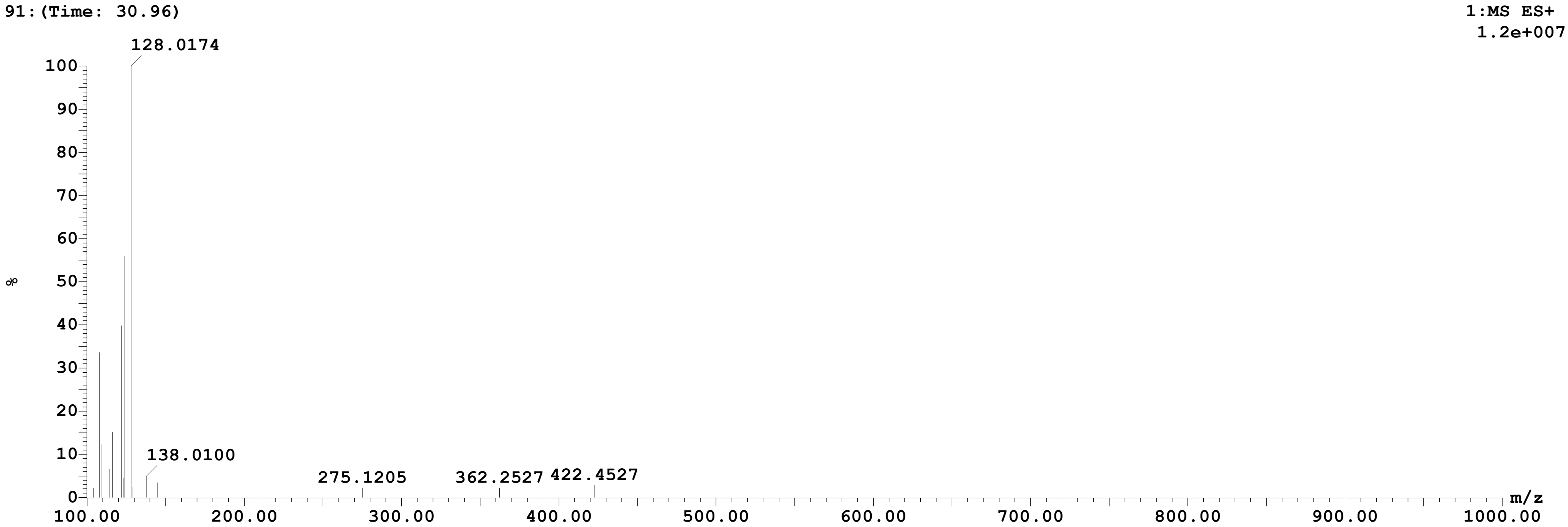

Center for DRUG DISCOVERY RESEARCH and DEVELOPMENT
Openlynx Report -

Sample: 122
File:E2479R
Description:Eth.AC (2)

Vial:1:A,2
Date:06-Nov-2024

ID:
Time:23:37:05

Printed: Sun Nov 24 12:56:41 2024

Peak ID	Time	Error PPM
92	31.19	

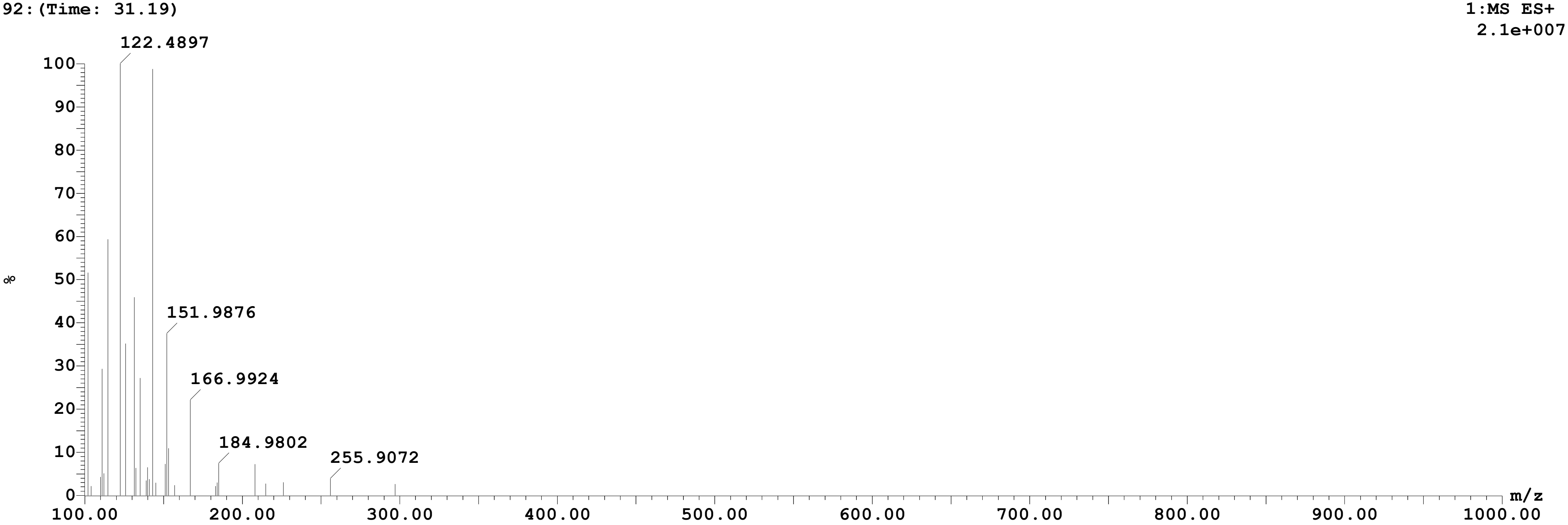

Center for DRUG DISCOVERY RESEARCH and DEVELOPMENT
Openlynx Report -

Sample: 122 | Vial:1:A,2 | ID:
File:E2479R | Date:06-Nov-2024 | Time:23:37:05
Description:Eth.AC (2)

Printed: Sun Nov 24 12:56:41 2024

Peak ID	Time	Error PPM
93	31.23	

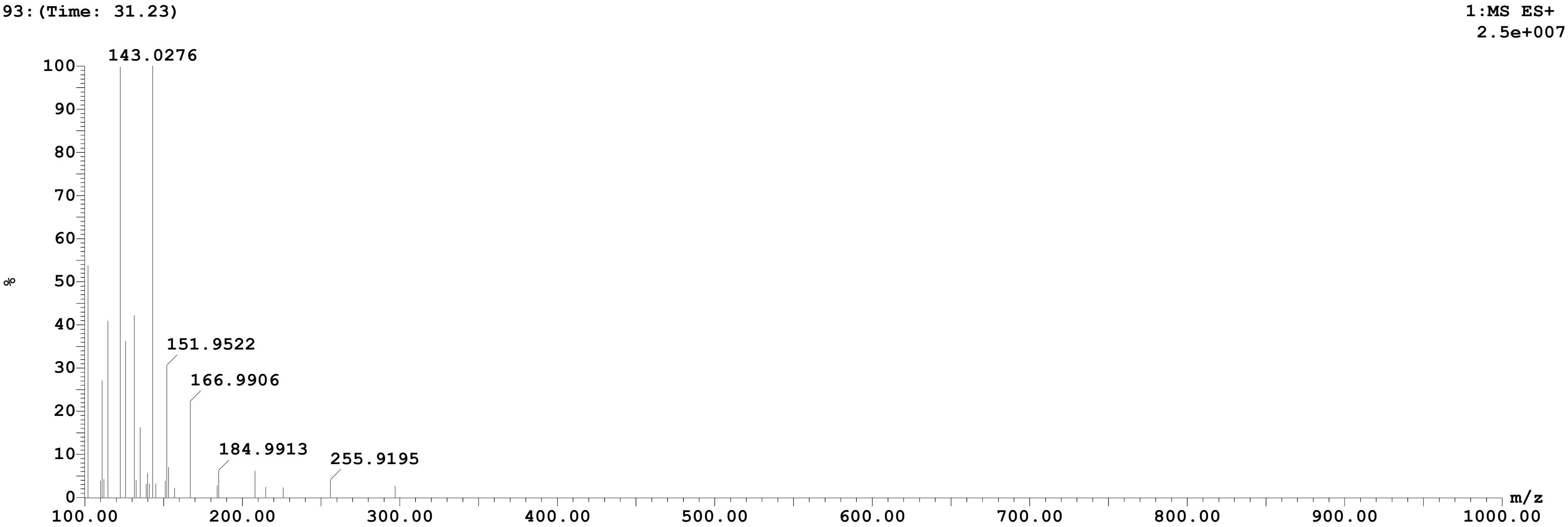

Center for DRUG DISCOVERY RESEARCH and DEVELOPMENT
Openlynx Report -

Sample: 122 | Vial:1:A,2 | ID:
File:E2479R | Date:06-Nov-2024 | Time:23:37:05
Description:Eth.AC (2)

Printed: Sun Nov 24 12:56:41 2024

Peak ID	Time	Error PPM
94	31.26	

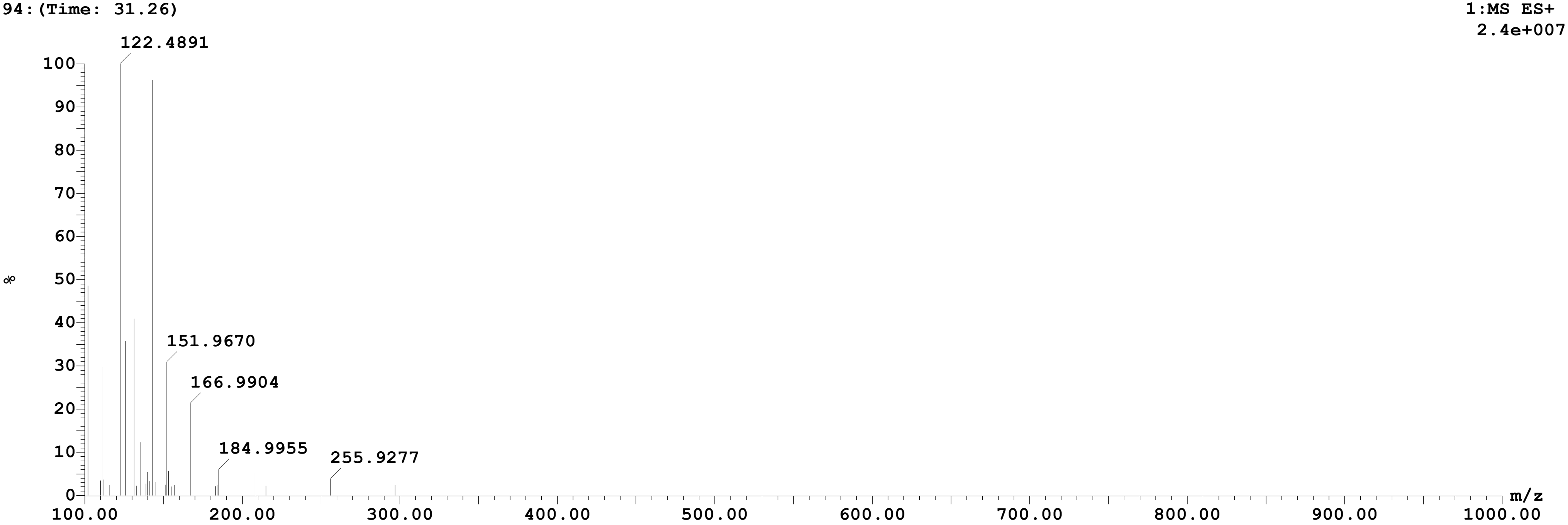

Center for DRUG DISCOVERY RESEARCH and DEVELOPMENT
Openlynx Report -

Sample: 122 | Vial:1:A,2 | ID:
File:E2479R | Date:06-Nov-2024 | Time:23:37:05
Description:Eth.AC (2)

Printed: Sun Nov 24 12:56:41 2024

Peak ID	Time	Error PPM
95	31.29	

95: (Time: 31.29)

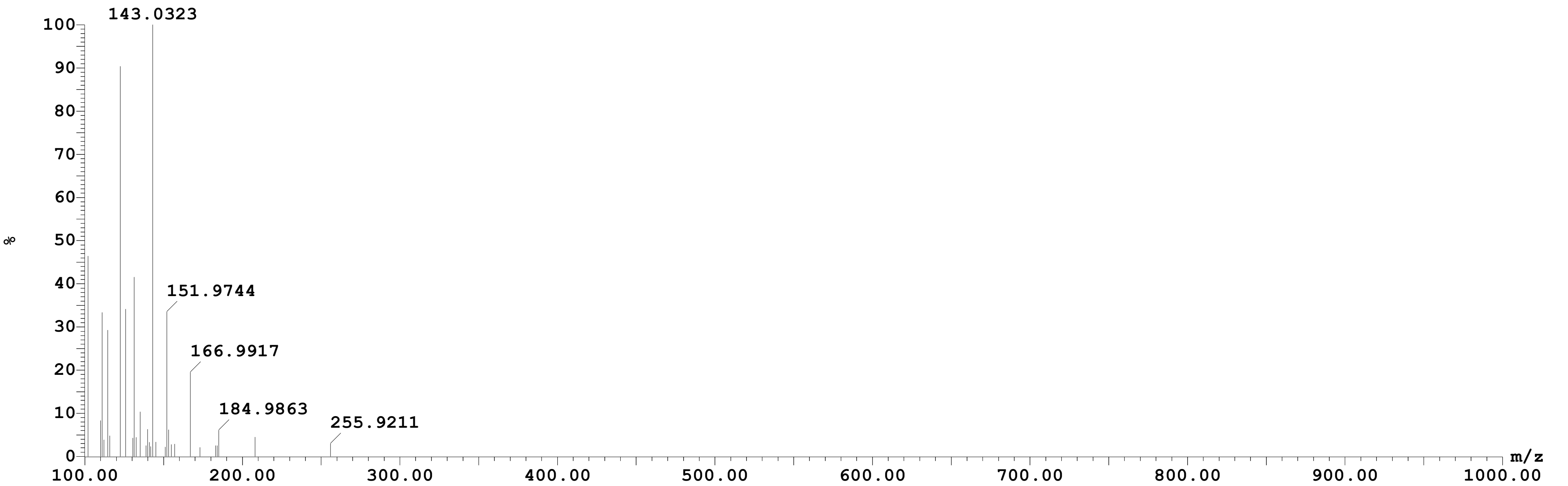

Sample: 122 Vial:1:A,2 ID:
File:E2479R Date:06-Nov-2024 Time:23:37:05
Description:Eth.AC (2)

Printed: Sun Nov 24 12:56:41 2024

HEADER

Header
Acquired File Name: E2479R
Acquired Date: 06-Nov-2024
Acquired Time: 23:37:05
Job Code: 2024
Task Code:
User Name:
Laboratory Name:
Instrument: XEVO-TQD#QCA423
Conditions:
Submitter:
SampleID:
Bottle Number: 1:A,2
Description: Eth.AC (2)

Sample: 122 Vial:1:A,2 ID:
File:E2479R Date:06-Nov-2024 Time:23:37:05
Description:Eth.AC (2)

Printed: Sun Nov 24 12:56:41 2024

CALIBRATION

Instrument Calibration:	
Calibration File:	C:\MassLynx\IntelliStart\Results\Y1\Calibration_20240911.cal
Parameters	
MS1 Static:	
Mass:	20 Da to 775 Da.
Resolution:	12.0/14.5
Ion Energy:	0.1
Reference File:	Naics1
Acquisition File:	STATMS1
MS1 Scanning:	
Mass:	10 Da to 2048 Da.
Resolution:	12.0/14.5
Ion Energy:	0.1
Reference File:	Naics1
Acquisition File:	SCNMS1
MS1 Scan Speed Compensation:	
Scan:	100 to 2000 amu/sec.
Resolution:	12.0/14.5
Ion Energy:	0.1
Reference File:	Naics1
Acquisition File:	FASTMS1
MS2 Static:	None
MS2 Scanning:	None
MS2 Scan Speed Compensation:	None
Calibration Time:	13:05
Calibration Date:	09/11/24
Coefficients	
MS1 Static:	0.000000000000*x^4 + -0.000000002321*x^3 + 0.000002745679*x^2 + 0.999021759284*x +0.133202347144
MS2 Static:	0.000000000000*x^4 + -0.000000001951*x^3 + 0.000001990911*x^2 + 0.999659890623*x +0.086980594036
Function 1:	0.000000000002*x^4 + -0.000000004845*x^3 + 0.000004793418*x^2 + 0.997906830084*x +0.025576511231
Parameters for C:	\LC-MS samples\2022-2023.PRO\ACQUDB\MS +ve 32 min.EXP

TUNE

MS

Data Processing:	
SIR / MRM Chromatogram Spike Removal:	ON
SIR / MRM Smoothing:	OFF
Smoothing window size (scans):	3
Number of smooths:	2
Prescan Statistics:	
Initial Average Intensity:	1.3475
Initial Average Std Dev:	1.1341
Bunch Zero Level:	0.0084
Bunch Std Dev:	0.0112
Bunch Threshold:	0.0898
Spike Removal Std Dev:	1.0568
Method Events:	
Initial Stop Flow:	No Change
Initial Switch 2:	No Change
Initial Switch 3:	No Change
Initial Switch 4:	No Change
Initial Infusion:	No Change
Initial Flow State:	LC
Initial Flow Rate:	5
Initial Reservoir:	No Action
API Probe Delay Temp:	20
Initial Refill:	No Action
Timed Events Enabled	
Event:	Time(Sec)NameAction
Source Information:	
Type - ESI	
Status - Option Connector Disconnected	
Ion Count Threshold:	30.0000
Instrument Parameters - Function 1:	

Sample: 122 Vial:1:A,2 ID:
File:E2479R Date:06-Nov-2024 Time:23:37:05
Description:Eth.AC (2)

Printed: Sun Nov 24 12:56:41 2024

Parameter File - C:	\LC-MS samples\2022-2023.PRO\ACQUDB\Y1.ipr
Polarity:	ES+
Calibration:	Dynamic 1
Capillary (kV):	3.00
Cone (V):	30.0033.94
RF (V):	2.50
Extractor (V):	3.00
Source Temperature (°C):	150
Desolvation Temperature (°C):	400
Cone Gas Flow (L/Hr):	50
Desolvation Gas Flow (L/Hr):	600
Collision Gas Flow (mL/Min):	OFF
LM 1 Resolution:	12.00
HM 1 Resolution:	14.53
Ion Energy 1:	0.10
MS Mode Entrance:	1.00
MS Mode Collision Energy:	2.00
MS Mode Exit:	1.00
MSMS Mode Entrance:	1.00
MSMS Mode Collision Energy:	2.00
MSMS Mode Exit:	1.00
LM 2 Resolution:	12.45
HM 2 Resolution:	15.63
Ion Energy 2:	0.10
Gas On MS Mode Entrance:	30.00
Gas On MS Mode Exit:	30.00
Gas On MSMS Mode Entrance:	1.00
Gas On MSMS Mode Exit:	1.00
Gas Off MS Mode Entrance:	30.00
Gas Off MS Mode Exit:	30.00
Gas Off MSMS Mode Entrance:	30.00
Gas Off MSMS Mode Exit:	30.00
Gain:	1.00
Active Reservoir:	Unknown
Cone Energy Ramp:	Disabled
Probe Temperature Ramp:	Disabled
Collision Energy Ramp:	Disabled
Engineers Settings:	

MS1 Low Mass Position:	519
MS1 High Mass Position:	197
MS1 Low Mass Resolution:	510
MS1 High Mass Resolution:	1635
MS1 Resolution Linearity:	929
MS1 High Mass DC Balance:	0.10
MS1 DC Polarity:	Positive
MS2 Low Mass Position:	519
MS2 High Mass Position:	241
MS2 Low Mass Resolution:	510
MS2 High Mass Resolution:	1660
MS2 Resolution Linearity:	900
MS2 High Mass DC Balance:	-0.15
MS2 DC Polarity:	Positive
HM RF Lens Correction +:	0.00
HM RF Lens Correction -:	0.00
Instrument Readbacks	
Capillary (kV):	3.03
Cone (V):	33.94
Extractor (V):	5.62
Source Temperature (°C):	148
Desolvation Temperature (°C):	399
Cone Gas Flow (L/Hr):	50
Desolvation Gas Flow (L/Hr):	592
Collision Gas Flow (mL/Min):	OFF

Inter-scan delays:
Automatic Mode
MS 1 Delay Table:

R		delay
<=	2.000	0.003
<=	3.000	0.005
<=	5.000	0.007
<=	7.000	0.009
>	7.000	0.010

MS 2 Delay Table:

R		delay
<=	30.000	0.003
>	30.000	0.005

Sample: 122 Vial:1:A,2 ID:
File:E2479R Date:06-Nov-2024 Time:23:37:05
Description:Eth.AC (2)

Printed: Sun Nov 24 12:56:41 2024

INLET

ACE Experimental Record
Inlet Method File: c:\lc-ms samples\2022-2023.pro\acqudb\extract acn
--------------------- Run method parameters ----------------

Sample: 122 Vial:1:A,2 ID:
File:E2479R Date:06-Nov-2024 Time:23:37:05
Description:Eth.AC (2)

Printed: Sun Nov 24 12:56:41 2024

PUMP

Waters ACQUITY QSM
Solvent A Name: Water + 0.1 % Formic acid
Solvent B Name: Methanol
Solvent C Name: 50%H2O+50%MeOH
Solvent D Name: Acetonitrile + 0.1 % Formic acid
Low Pressure Limit: 0 psi
High Pressure Limit: 14000 psi
Seal Wash Period: 30.00 min

Gradient

Time (min)	Flow Rate	A%	B%	Curve
Initial	0.200	90.0	0.0	0.0
2.00	0.200	90.0	0.0	0.0
5.00	0.200	70.0	0.0	0.0
15.00	0.200	30.0	0.0	0.0
22.00	0.200	10.0	0.0	0.0
25.00	0.200	10.0	0.0	0.0
26.00	0.200	0.0	0.0	0.0
29.00	0.200	0.0	0.0	0.0
32.00	0.200	90.0	0.0	0.0

Comment:

Flow Ramp Rate:	0.45 min
D Solvent Selection (if supported):	No Change
System Pressure Data Channel:	No
Flow Rate Data Channel:	No
%A Data Channel:	Yes
%B Data Channel:	No
%C Data Channel:	No
%D Data Channel:	Yes
Primary Data Channel:	No
Accumulator Data Channel:	No
Degasser Data Channel:	No
Gradient Start:	At Injection
Gradient Start Volume:	0 uL
Gradient Start Time:	0.00 min
Participate in pre-analysis:	No

Sample: 122	Vial:1:A,2	ID:
File:E2479R	Date:06-Nov-2024	Time:23:37:05
Description:Eth.AC (2)		

Printed: Sun Nov 24 12:56:41 2024

AUTOSAMPLER

Waters ACQUITY FTN AutoSampler	
Run Time:	32.00 min
Comment:	
Load Ahead:	Disabled
Loop Offline:	Automatic min
Wash Solvent Name:	Water
Pre-Inject Wash Time:	0.0 sec
Post-Inject Wash Time:	15.0 sec
Purge Solvent Name:	Water
Dilution:	Disabled
Dilution Volume:	0 uL
Delay Time:	0 min
Dilution Needle Placement:	4 mm
Target Column Temperature:	Off C
Target Sample Temperature:	Off C
Syringe Draw Rate:	Automatic
Needle Placement:	Automatic
Pre-Aspirate Air Gap:	Automatic
Post-Aspirate Air Gap:	Automatic
Column Temperature Data Channel:	No
Room Temperature Data Channel:	No
Sample Temperature Data Channel:	No
Sample Organizer Temperature Data Channel:	No
Sample Pressure Data Channel:	No
Preheater Temperature Data Channel:	No
Seal Force Data Channel:	No
No Injection Mode Enabled:	No
Autoaddition Mix Stroke Cycles:	Automatic
Autoaddition Mix Stroke Volume:	Automatic uL
Run Events:	No
Active Preheater:	Use Console Configuration

Sample Run Injection Parameter
Injection Volume (ul) - 10.00
---------------------------- oOo -----------------------------
End of experimental record.
------------------- Waters ACQUITY QSM Postrun Report ---------------

Openlynx Report -

Sample: 122	Vial:1:A,2	ID:
File:E2479R	Date:06-Nov-2024	Time:23:37:05
Description:Eth.AC (2)		

Printed: Sun Nov 24 12:56:41 2024

Firmware Version:	1.71.381 (Feb 14 2018)
Software Version:	1.71.2797
Checksum:	0x94ae01d3
Serial Number:	Serial Number Uninitialized
Minimum System Pressure:	1484.0 psi
Maximum System Pressure:	5245.0 psi
Average System Pressure:	3213.0 psi

--------------------------- oOo ----------------------------

------------------ Waters ACQUITY FTN Postrun Report --------------

Software Version:	1.71.2686
Firmware Version:	1.71.395 (Feb 14 2018)
Checksum:	0x285858d7
Serial Number:	F13SDI420G
Sample Syringe Size:	100.0
Extension Loop Size:	0.0
Needle Size:	15.0
Minimum Sample Temperature:	25.9 C
Maximum Sample Temperature:	26.2 C
Average Sample Temperature:	26.0 C
Minimum Column Temperature:	0.0 C
Maximum Column Temperature:	0.0 C
Average Column Temperature:	0.0 C

--------------------------- oOo ----------------------------

FUNCTION

Function 1

Scans in function:	3759
Cycle time (secs):	Automatic
Scan duration (secs):	0.500
Inter Scan Delay (secs):	Automatic
Start and End Time(mins):	0.000 to 32.000
Ionization mode:	ES+
Data type:	Enhanced Mass
Function type:	Scan
Mass range:	100 to 1000
